# Crystallographic fragment screening-based study of a novel FAD-dependent oxidoreductase from *Chaetomium thermophilum*

**DOI:** 10.1107/S2059798321003533

**Published:** 2021-05-14

**Authors:** Leona Švecová, Lars Henrik Østergaard, Tereza Skálová, Kirk Matthew Schnorr, Tomáš Koval’, Petr Kolenko, Jan Stránský, David Sedlák, Jarmila Dušková, Mária Trundová, Jindřich Hašek, Jan Dohnálek

**Affiliations:** a Institute of Biotechnology of the Czech Academy of Sciences, v.v.i., Průmyslová 595, 252 50 Vestec, Czech Republic; b Faculty of Nuclear Sciences and Physical Engineering, Czech Technical University in Prague, Břehová 7, 115 19 Prague 1, Czech Republic; c Novozymes A/S, Biologiens Vej 2, 2800 Kgs. Lyngby, Denmark; d CZ-OPENSCREEN: National Infrastructure for Chemical Biology, Institute of Molecular Genetics of the Czech Academy of Sciences, v.v.i., Vídeňská 1083, 142 20 Prague, Czech Republic

**Keywords:** FAD-dependent oxidoreductases, GMC oxidoreductases, *Chaetomium thermophilum*, crystallographic fragment screening

## Abstract

The FAD-dependent oxidoreductase from *Chaetomium thermophilum* (*Ct*FDO) is a novel thermostable glycoprotein from the glucose–methanol–choline (GMC) oxidoreductase superfamily. However, *Ct*FDO shows no activity toward the typical substrates of the family and high-throughput screening with around 1000 compounds did not yield any strongly reacting substrate. Therefore, protein crystallography, including crystallographic fragment screening, with 42 fragments and 37 other compounds was used to describe the ligand-binding sites of *Ct*FDO and to characterize the nature of its substrate. The structure of *Ct*FDO reveals an unusually wide-open solvent-accessible active-site pocket with a unique His–Ser amino-acid pair putatively involved in enzyme catalysis. A series of six crystal structures of *Ct*FDO complexes revealed five different subsites for the binding of aryl moieties inside the active-site pocket and conformational flexibility of the interacting amino acids when adapting to a particular ligand. The protein is capable of binding complex polyaromatic substrates of molecular weight greater than 500 Da.

## Abstract 2

The high-resolution crystal structure of a novel FAD-dependent oxidoreductase from the GMC oxidoreductase superfamily reveals a novel His–Ser active-site pair located in an extensive active-site pocket. Crystallographic fragment screening led to the identification of subsites inside the active-site pocket, indicating a preference for polyaromatic substrates.

## 1. Introduction   

The thermophilic filamentous fungus *Chaetomium thermophilum* (*Ct*) found in soil, dung and compost heaps significantly participates in cellulose decomposition. The optimal temperature for its growth ranges between 45°C and 55°C, temperatures that are appropriate for lignin-degradation processes in compost. *Ct* has become a potential source of thermostable proteins that are of interest for various commercial and industrial high-temperature processes. *Ct* has also been widely used in academic studies, enabling, for instance, structural studies of many proteins (Amlacher *et al.*, 2011 ▸; Tuomela *et al.*, 2000 ▸; Zhou *et al.*, 2017 ▸; Maheshwari *et al.*, 2000 ▸).

Based on its sequence and fold, the novel FAD-dependent oxidoreductase from *Chaetomium thermophilum* var. *thermo­philum* (*Ct*FDO) belongs to the glucose–methanol–choline (GMC) oxidoreductase superfamily, which includes aryl-alcohol oxidases, glucose oxidases, glucose dehydrogenases, choline oxidases, cholesterol oxidases, pyranose 2-oxidases, pyridoxine 4-oxidases and others. Although the GMC oxidoreductases catalyse the oxidation of different types of substrates, they share core structural elements, a two-domain character, a conserved N-terminal Gly-*X*-Gly-*X-X*-Gly sequence motif characteristic of the initial βαβ segment of the Rossmann fold that binds the adenosine diphosphate moiety of FAD, and a conserved active-site histidine. The conserved histidine plays the role of the catalytic base in the majority of GMC oxidoreductases (Romero & Gadda, 2014 ▸; Wohlfahrt *et al.*, 1999 ▸; Hernández-Ortega *et al.*, 2012 ▸; Yoshida *et al.*, 2015 ▸; Wongnate & Chaiyen, 2013 ▸; Mugo *et al.*, 2013 ▸; Graf *et al.*, 2015 ▸; Smitherman *et al.*, 2015 ▸; Dijkman *et al.*, 2015 ▸) and is usually present in combination with another histidine or an aspara­gine residue, which can form a hydrogen bond to an alcohol substrate. Both residues are situated on the *re* face of the FAD isoalloxazine ring, creating a His–His or His–Asn pair (Sützl *et al.*, 2019 ▸).

The catalytic reaction in GMC oxidoreductases generally consists of reductive and oxidative half-reactions. Most of the members of the GMC superfamily act on the hydroxyl groups of non-activated alcohols, carbohydrates and sterols. The reductive half-reaction involves direct hydroxyl-proton transfer from the substrate to the active-site base and the extraction of two electrons and a proton by FAD, yielding a carbonyl on the substrate, a protonated base (His) and a reduced FAD cofactor (an anionic hydroquinone). During the oxidative half-reaction, the FAD cofactor is re-oxidized by the transfer of two electrons and a proton to a suitable electron acceptor such as oxygen or a quinone. A second proton is transferred from the base. The varying substrate specificities arise from differences in the active site (Romero & Gadda, 2014 ▸; Carro *et al.*, 2017 ▸).

Biotechnology and white industry are constantly searching for novel enzymes, which often have new functionalities and are able to withstand conditions relevant to their industrial application. However, such a search can lead to the substrates for a given enzyme being hard to identify. Crystallographic screening of low-molecular-weight fragments is a powerful method for mapping the binding sites in the target protein and for the identification of chemical groups that specifically interact with the target. The usual aim of the method is the utilization of the group of binding fragments as a starting point for new drug design. Nevertheless, it also has potential for the characterization of binding sites for unknown substrates and for the prediction of enzymatic function.

Here, we report the process of *Ct*FDO production together with biophysical and structural studies. A large activity screening showed *Ct*FDO to be inactive with the tested compounds, including common substrates of GMC oxido­reductases. X-ray structural analysis of *Ct*FDO revealed novel features of the active-site pocket that are likely to indicate a different type of substrate to those common for GMC oxidoreductases. Here, we present the utilization of crystallographic screening of fragments and compounds for the localization of binding sites, identification of chemical groups of putative substrates and prediction of the substrate specificity of *Ct*FDO.

## 2. Materials and methods   

### 2.1. Protein expression and purification   

The gene encoding the oxidoreductase (the complete sequence; Supplementary Fig. S1) was cloned using genomic DNA prepared from *C. thermophilum* var. *thermophilum* (strain CBS144.50/DSM 1495). The resulting gDNA was used as the template in a PCR reaction to amplify the gene of interest with the forward primer 5′-ACACAACTGGGGATCCACCATGAGGACATCAAGTTTCCAGC-3′ and the reverse primer 5′-CGGCCAAACAATCGGAGTAATAAGCTTCTCGAGATCT-3′. The PCR product obtained was cloned into an expression vector essentially as described by Christensen *et al.* (1988 ▸) using Clontech InFusion cloning. The DNA construct was verified by sequencing and subsequently transformed into *Aspergillis oryzae* protoplasts for expression.

The enzyme was produced by submerged fermentation in shake flasks each containing 150 ml DAP4C-1 medium supplemented with 5 ml of 20% lactic acid and 3.5 ml of 50% diammonium phosphate. DAP4C-1 medium was composed of 0.5 g yeast extract, 10 g maltose, 20 g dextrose, 11 g magnes­ium sulfate heptahydrate, 1 g dipotassium phosphate, 2 g citric acid monohydrate, 5.2 g potassium phosphate tribasic monohydrate, 1 ml Dowfax 63N10 (an antifoaming agent) and 2.5 g calcium carbonate supplemented with 1 ml KU6 metal solution and deionized water to 1000 ml. The KU6 metal solution consisted of 6.8 g zinc chloride, 2.5 g cupric sulfate penta­hydrate, 0.13 g nickel(II) chloride, 13.9 g iron(II) sulfate heptahydrate, 8.45 g manganese sulfate monohydrate, 3 g citric acid and deionized water to 1000 ml.

The flasks were inoculated with spores from the *A. oryzae* strain expressing the recombinant enzyme and cultivated in shake flasks for four days at a temperature of 30°C with agitation at 180 rev min^−1^. The total volume of 2.7 l culture broth was harvested by filtration using a 0.2 µm filter device, which resulted in 2.5 l sterile broth that was used as a starting material for purification.

The sterile-filtered fermentation broth was cross-flow filtered using a suspended screen channel filtration unit from Pall Corporation (OMEGA 10K 34041102BL) to remove salts and to concentrate the sample to a final volume of 150 ml. The pH of the solution was subsequently adjusted to 7.5 by titration with a solution of 1 *M* Tris pH 7.5.

The enzyme was purified by cation-exchange chromatography using a 25 ml XK16 column containing S-Source that was connected to an ÄKTA FPLC system. The column was pre-equilibrated with 25 m*M* Tris–HCl pH 7.5 before loading the column with the prepared starting material. Unbound proteins were removed by washing with 25 m*M* Tris–HCl until a baseline level was obtained at 280 nm. Bound proteins were then eluted over 300 ml using a linear gradient of 0–0.5 *M* NaCl in 25 m*M* Tris–HCl pH 7.5. The flow rate during chromatography was 10 ml min^−1^ and 10 ml fractions were collected during elution. The enzyme eluted at approximately 200 m*M* NaCl. The purity of the collected fractions was analysed by SDS–PAGE and an absorbance scan. The pure enzyme fractions were pooled and concentrated using Amicon spin filters with a 10 kDa cutoff. All steps of the purification were performed at room temperature.

### 2.2. Sample deglycosylation   

The deglycosylation procedure was performed using endoglycosidase F1 according to the assay stated on the Sigma–Aldrich website (https://www.sigmaaldrich.com/). The reaction mixture was incubated at 37°C for 2 h and then at 4°C overnight. The effect of deglycosylation was examined by SDS–PAGE. The deglycosylated sample (*Ct*FDO_degl_) was concentrated to 8 mg ml^−1^ in the storage buffer (25 m*M* Tris–HCl pH 7.5 with 100 m*M* NaCl) using a Nanosep 10K centrifugal device with molecular-weight cutoff 10 kDa (Pall Corporation).

#### 2.2.1. Thermal stability measurement   

The change in the thermal stability of glycosylated and deglycosylated *Ct*FDO (*Ct*FDO_degl_) was measured using nano differential scanning fluorimetry (nanoDSF). The nanoDSF profiles were measured in the temperature range 20–95°C with a temperature slope of 2.5°C min^−1^ and an excitation power of 45% using a Prometheus NT.48 (NanoTemper). The samples were diluted in storage buffer to a final concentration of 0.7 mg ml^−1^. Evaluation of the data was performed using the *PR.ThermControl* v.2.1.1 software.

#### 2.2.2. UV–Vis spectrophotometry of *Ct*FDO in solution   

UV–Vis absorption spectra of *Ct*FDO and *Ct*FDO_degl_ samples were collected using a DeNovix DS-11 microvolume spectrophotometer and 1.2 µl samples in storage buffer at a protein concentration of 7.2 mg ml^−1^ at room temperature. The spectra were buffer-subtracted.

### 2.3. Sequence analysis by mass spectrometry   

#### 2.3.1. Liquid-chromatography tandem mass spectrometry (LC-MS/MS)   

The *Ct*FDO_degl_ sample was diluted in 0.5 *M* glycine buffer pH 2.3 and 300 pmol was injected onto a pepsin column for online protein digestion. After 3 min of digestion and trapping, the peptides were separated by a 1290 series UHPLC system (Agilent Technologies) on a reverse-phase C18 column linked to the electrospray ion source of a 15T SolariX XR FT-ICR mass spectrometer (Bruker Daltonics). The mass spectrometer was operated in positive data-dependent mode. Data were processed using the *DataAnalysis* 4.2 software and exported to mgf format. *ProteinScape* (Bruker Daltonics) with the *Mascot* search engine was used for the identification of peptides.

#### 2.3.2. MALDI-TOF peptide mass fingerprinting   

SDS gel bands with 4 µg *Ct*FDO_degl_ were cut out, chopped into small pieces and dehydrated using acetonitrile. Dithiothreitol at a concentration of 50 m*M* was added to the gel pieces. After 30 min of incubation at 60°C, iodoacetamide at a concentration of 100 m*M* was added to the gel pieces and left to incubate in the dark at room temperature for 30 min. The gel pieces were further washed with water and dehydrated using acetonitrile. Trypsin solution was added to the gel pieces and left to incubate at 37°C overnight. 1 µl of tryptic peptide mixture was applied onto the stainless-steel MALDI target, covered with α-cyano-4-hydroxycinnamic acid as a matrix and analysed using a 15T SolariX XR FT-ICR mass spectrometer (Bruker Daltonics) operating in positive mode. Data were processed using the *DataAnalysis* 4.2 (Bruker Daltonics) and *mMass* software.

### 2.4. Molecular-mass determination   

#### 2.4.1. MALDI-TOF mass spectrometry   

1 µl protein sample (*Ct*FDO and *Ct*FDO_degl_) at a concentration of 10 nmol ml^−1^ was applied onto a stainless-steel MALDI target and left to dry. The sample was overlaid with 1 µl sinapinic acid (Sigma–Aldrich) and left to dry at room temperature. The intact protein was analysed using an Autoflex Speed MALDI-TOF mass spectrometer (Bruker Daltonics) operated in linear positive mode. Data were processed using the *FlexAnalysis* 3.3 software (Bruker Daltonics).

#### 2.4.2. Mass photometry   

Data were collected on a Refeyn OneMP instrument using the *DiscoverMP* software (version 2.2.1). The measurements were performed using clean cover slips (High Precision cover slips, No. 1.5, 24 × 50 mm, Marienfeld) mounted with silicon gaskets (CultureWell Reusable Gaskets, Grace Biolabs). Samples were diluted in 25 m*M* Tris–HCl, 75 m*M* NaCl pH 7.5 to final concentrations of 1.7 µg ml^−1^ (20 n*M*) and 1.6 µg ml^−1^ (24 n*M*) for *Ct*FDO and *Ct*FDO_degl_, respectively. Data were acquired and analysed using *DiscoverMP* (version 2.3.dev12) using default settings.

#### 2.4.3. Small-angle X-ray scattering   

The small-angle X-ray scattering (SAXS) experiment was performed with the deglycosylated form of *Ct*FDO (*Ct*FDO_degl_). The SAXS experiment was performed in batch mode on the EMBL P12 beamline at the PETRA III synchrotron-radiation source, DESY, Hamburg using a PILATUS 6M detector (Dectris, Baden-Daettwil, Switzerland). The measurements were performed at 20°C with a sample-to-detector distance of 3.0 m, a wavelength of 1.24 Å and an exposure time per image of 0.045 s. Data were collected for *Ct*FDO_degl_ (4.2 mg ml^−1^) dissolved in 25 m*M* Tris–HCl pH 7.5 with 25 m*M* NaCl. Data processing was performed with the *ATSAS* 3.0.0 package (Manalastas-Cantos *et al.*, 2021 ▸). As the data had fair quality according to the shape of the scattering curve, Guinier analysis and Kratky plot, only the molecular weight was estimated. The molecular envelope was not calculated.

### 2.5. Crystallization   

Initial crystallization condition screening with commercially available kits was performed using the sitting-drop vapour-diffusion technique with drops composed of 0.3 µl *Ct*FDO solution at a concentration of 8 mg ml^−1^ in 25 m*M* Tris–HCl pH 7.5, 100 m*M* NaCl and 0.3 µl reservoir solution. The screening did not lead to any crystalline material, probably as a consequence of a large number of glycans on *Ct*FDO (seven possible glycosylation sites according to the *NetNGlyc* 1.0 server; http://www.cbs.dtu.dk/services/NetNGlyc/). Therefore, crystallization screening was repeated with the same setup using deglycosylated *Ct*FDO (*Ct*FDO_degl_ at a concentration of 8 mg ml^−1^ in 25 m*M* Tris–HCl pH 7.5, 100 m*M* NaCl). Crystalline material appeared using condition No. 30 of the PEGRx 2 screen [0.1 *M* sodium acetate pH 4.0, 18%(*w*/*v*) polyethylene glycol 5000, 0.2 *M* magnesium formate; Hampton Research] in 14 days. The optimized condition consisted of 0.1 *M* sodium acetate pH 5.5, 17%(*w*/*v*) polyethylene glycol 5000, 0.16 *M* magnesium formate. Crystals of ligand-free *Ct*FDO_degl_ were prepared with reservoir solution enriched with 20 m*M* cystamine. Cystamine was initially used for complex preparation, but the structure lacked electron density for cystamine. As this was the highest resolution structure of the nonliganded *Ct*FDO_degl_ form, it is presented as the ligand-free structure of *Ct*FDO_degl_ (*Ct*FDO:free). Single crystals of rectangular shape grew at 20°C in 1 µl hanging drops with a 1:1 protein:reservoir ratio in 7–14 days.

#### 2.5.1. Preparation of complexes with ligands   

42 fragments from the Frag Xtal Screen (Jena Bioscience; Huschmann *et al.*, 2016 ▸) and 37 other compounds (Supplementary Table S1) were selected for soaking into *Ct*FDO_degl_ crystals or for co-crystallization with *Ct*FDO_degl_. The fragments were selected according to the expected stereochemistry of the putative substrate/product of lignin degradation (excluding compounds with exotic chemistry). The other 37 compounds are substrates of GMC oxidoreductases, compounds similar to aryl alcohols and commercially available compounds found in compost. The screening with the fragments (50 nmol each) was performed according to the protocol from Jena Bioscience (https://www.jenabioscience.com/) with a soaking time from 1 to 70 h and a temperature of 20°C. Crystals of the complexes of *Ct*FDO_degl_ with the fragments methyl-4-(aminomethyl)benzoate (MAMB) and 4-oxo-*N*-[1-(3-pyridinyl)ethyl]-2-thiophene­butanamide (PESB) were obtained after soaking for 2.5 and 3 h, respectively. The crystals used for the preparation of the complexes with 2-(1*H*-indol-3-yl)-*N*-[(1-methyl-1*H*-pyrrol-2-yl)methyl]ethanamine (IPEA) and 4-nitrocatechol (4NC) were prepared using a crystallization condition enriched with 20 m*M* MgCl_2_ and with soaking times of 70 and 20 h, respectively. Crystals of *Ct*FDO_degl_ complexed with 4-nitrophenol (4NP) and 2,2′-azino-bis(3-ethylbenzthiazoline-6-sulfonic acid) (ABTS) were prepared by co-crystallization (Supplementary Table S1): the reservoir solution contained an additional 20 m*M* 4NP or 8.5 m*M* ABTS and 20 m*M* MgCl_2_, respectively.

### 2.6. Diffraction data collection and processing   

A crystal of ligand-free *Ct*FDO_degl_ was cryoprotected with reservoir solution containing 5%(*v*/*v*) polyethylene glycol 200 and 5% glycerol for 22 min and was subequently vitrified and stored in liquid nitrogen. The crystals of the complexes were cryoprotected using perfluoropolyether cryo oil (Hampton Research). Diffraction data were collected either on beamline P13 of the PETRA III synchrotron-radiation source, DESY, Hamburg, Germany or on beamlines 14.1 and 14.2 of the BESSY II synchrotron-radiation source, Helmholz-Zentrum, Berlin, Germany. All diffraction data were recorded at 100 K using a PILATUS 6M or a PILATUS 2M detector (Dectris). The statistics from the data processing as well as a detailed description of the experimental setup are summarized in Table 1 ▸.

All diffraction data were auto-indexed, integrated and scaled using *XDSgui* (Kabsch, 2010 ▸). Merging was performed in *AIMLESS* (Evans & Murshudov, 2013 ▸). The phase problem of the ligand-free *Ct*FDO_degl_ structure (*Ct*FDO:free) was solved by molecular replacement in *MoRDa* (Vagin & Lebedev, 2015 ▸) with the structure of *A. flavus* FAD glucose dehydrogenase (*Af*GDH; PDB entry 4ynt; Yoshida *et al.*, 2015 ▸; sequence identity 30%) as a model. Missing residues were built in the electron-density map using the *Phenix Autobuild* wizard (Terwilliger, Grosse-Kunstleve, Afonine, Moriarty, Zwart *et al.*, 2008 ▸). The phase problems of the complexed structures, except for that with IPEA (*Ct*FDO:IPEA), were solved by molecular replacement with *MOLREP* (Vagin & Teplyakov, 2010 ▸) [complexes with MAMB (*Ct*FDO:MAMB) and PESB (*Ct*FDO:PESB)] or *Phaser* (McCoy *et al.*, 2007 ▸) [complexes with 4NC (*Ct*FDO:4NC), 4NP (*Ct*FDO:4NP) and ABTS (*Ct*FDO:ABTS)] using the structure of *Ct*FDO:free as a model. All structures were manually edited in *Coot* (Emsley *et al.*, 2010 ▸). The *Ct*FDO:free, *Ct*FDO:MAMB, *Ct*FDO:PESB, *Ct*FDO:4NC, *Ct*FDO:4NP and *Ct*FDO:ABTS structures were refined in *REFMAC*5 (version 5. 8. 0258; Murshudov *et al.*, 2011 ▸) and the structure of *Ct*FDO:IPEA in *phenix.refine* (version 1.18.1-3865; Afonine *et al.*, 2012 ▸). *R*_free_ calculation was used as the cross-validation method. For high-resolution structures with a large number of measured reflections (*Ct*FDO:free and *Ct*FDO:4NC), 2% of the reflections were used as a test set. For the rest of the structures with lower resolution, 4.8–5.1% of the reflections were used as a test set (Table 2 ▸). H atoms in riding positions were used in all refinements. All *Ct*FDO_degl_ structures were refined with the reduced form of the flavin adenine di­nucleotide (FADH_2_) cofactor. The geometrical restraints for the cofactor as well as for the ligands MAMB, PESB, IPEA, 4NC, 4NP and ABTS were generated using *AceDRG* (Long *et al.*, 2017 ▸) and were manually checked and edited. Problematic water molecules in the solvation layers at the protein surface were restrained. The structures of *Ct*FDO:free, *Ct*FDO:4NC and *Ct*FDO:ABTS were refined with anisotropic ADPs. For all structures, the last refinement cycle in *REFMAC*5 and *phenix.refine* was performed with all measured reflections. The structure quality was assessed by the validation tools implemented in *Coot*, *MolProbity* (Chen *et al.*, 2010 ▸) and the wwPDB Validation Service (Berman *et al.*, 2003 ▸). Carbo­hydrate residues were checked by the *pdb-care* program (Lütteke & Lieth, 2004 ▸). Quality indicators from structure refinement are summarized in Table 2 ▸.

#### 2.6.1. PDB accession codes   

The coordinates and structure factors of the *Ct*FDO:free, *Ct*FDO:MAMB, *Ct*FDO:PESB, *Ct*FDO:IPEA, *Ct*FDO:4NC, *Ct*FDO:4NP and *Ct*FDO:ABTS structures have been deposited in the PDB as entries 6ze2, 6ze3, 6ze4, 6ze5, 6ze6, 6ze7 and 7aa2, respectively. The X-ray diffraction image data have been deposited in the Structural Biology Data Grid (https://data.sbgrid.org/) at https://doi.org/10.15785/SBGRID/804 for *Ct*FDO:free, https://doi.org/10.15785/SBGRID/805 for *Ct*FDO:MAMB, https://doi.org/10.15785/SBGRID/806 for *Ct*FDO:PESB, https://doi.org/10.15785/SBGRID/807 for *Ct*FDO:IPEA, https://doi.org/10.15785/SBGRID/808 for *Ct*FDO:4NC, https://doi.org/10.15785/SBGRID/809 for *Ct*FDO:4NP and https://doi.org/10.15785/SBGRID/810 for *Ct*FDO:ABTS.

### 2.7. Quality and content of structures   

All *Ct*FDO_degl_ crystal structures except for *Ct*FDO:MAMB contain two molecules of *Ct*FDO_degl_ in the asymmetric unit, referred to as chains *A* and *B*. All structures bind an Mg^2+^ ion coordinated by six water molecules at the contact of the chains between proline-rich loops (Pro172–Pro175) in both chains. Each molecule, except that of *Ct*FDO:MAMB, binds a formic acid molecule in the β-turn consisting of residues Pro413–Ser415. The fragments (MAMB, PESB, IPEA and 4NC), 4NP and ABTS were modelled in the active-site pocket of *Ct*FDO_degl_. All structures reveal at least two carbohydrate units modifying Asn197, indicating unsuccessful cleavage of the glycans at this site, probably due to a deeper immersion of the glycosylation site in the enzyme.

For the *Ct*FDO:free crystal structure at 1.31 Å resolution, Ramachandran analysis in *MolProbity* (Chen *et al.*, 2010 ▸) showed all residues to be in allowed regions. One *N*-acetyl-d-glucosamine (GlcNAc) moiety was modelled linked to Asn114, Asn182 and Asn374 of both chains and to Asn543 of chain *A*. Two GlcNAc moieties were modelled linked to Asn197 of both chains.

The *Ct*FDO:MAMB structure at a resolution of 2.2 Å has one molecule of *Ct*FDO_degl_ in the asymmetric unit that binds one molecule of MAMB and a molecule of formic acid in the active-site pocket. Ramachandran analysis shows two outliers: Thr469, which is in good agreement with 2*m* *F*_o_ − *DF*_c_ density, and Asn295, with a glycan with ambiguous electron density. One GlcNAc moiety was modelled linked to Asn114, Asn182, Asn295 and Asn374, and two GlcNAc units linked to Asn197.

The *Ct*FDO:PESB structure at a resolution of 1.6 Å has a lower data completeness (93.5%; Table 1 ▸) as a consequence of omitting regions on the detector due to ice rings. Two outliers according to the Ramachandran plot (Thr469 of both chains) are in good agreement with electron density. A GlcNAc unit was modelled linked to Asn114, Asn182, Asn295 and Asn374 of both chains. Asn197 in both chains was modified by two units of GlcNAc and one additional unit of mannose in chain *B*. The PESB fragment (0.9 occupancy) and one formic acid molecule were modelled in the active-site pocket.

The *Ct*FDO:IPEA complex solved at a resolution of 1.82 Å has two outliers in the Ramachandran plot (alternative *B* of Gly293 of chain *B* and Thr469 of chain *B*) that fit well into electron density. Both *Ct*FDO_degl_ molecules bind one formic acid moiety and one IPEA fragment in the active-site pocket. One additional IPEA fragment was modelled at the crystal contact between chain *A* and chain *A* of a symmetry-related molecule. One GlcNAc unit was modelled modifying Asn114, Asn182 and Asn374 and two units modifying Asn197 of both chains.

The *Ct*FDO:4NC complex solved at a resolution of 1.26 Å was refined with anisotropic ADPs except for 452 water molecules. A GlcNAc moiety was modelled linked to Asn114, Asn182, Asn295 and Asn374 of both chains and to Asn543 of chain *A*. Two GlcNAc units were modelled modifying Asn197. One *Ct*FDO_degl_ binds two 4NC molecules (occupancies of 0.7 and 0.8) and one molecule of formic acid in the active-site pocket. A strong peak (6σ) in the *mF*_o_ − *DF*_c_ difference electron-density map was found at the contact of two chains between residues Trp97 of chain *A* and Lys625 of the symmetry-related chain *B*. This peak was left unmodelled.

The *Ct*FDO:4NP complex at 1.5 Å resolution has three Ramachandran outliers: Thr469 of both chains, with good agreement with electron density, and Arg628 of chain *A*, which is located at the disordered C-terminus of the chain. Asparagines Asn114, Asn182, Asn197 and Asn374 were modelled modified in the same way as those of *Ct*FDO:IPEA. Chain *A* binds two molecules of 4NP (both with occupancy 0.8) and one of formic acid, and chain *B* binds one 4NP (0.8 occupancy) and one formic acid molecule in the active-site pocket. Besides these ligands and a formic acid binding to the β-turn (above), the protein binds four additional formic acid molecules, two acetic acid molecules and four polyethylene glycol moieties (two tetraethylene glycol and two triethylene glycol) at the crystal contacts.

*Ct*FDO:ABTS solved at 1.4 Å resolution was refined with anisotropic ADPs except for the water molecules and a GlcNAc moiety modelled linked to Asn197 of both chains. The ABTS molecule was modelled in the active-site pocket. The occupancy of ABTS was decreased (0.6 for one benzothiazoline moiety and 0.9 for the second) as the electron density for ABTS indicates its displacement in a direction away from the active site and the alternative conformation of Trp97 excludes full occupancy of the whole ABTS molecule. Trp97 and ABTS were modelled with complementary occupancies. Asparagine residues Asn114, Asn182, Asn197, Asn295, Asn374 and Asn543 have the same glycans built as those of *Ct*FDO:4NC. A strong peak (4.2σ) in difference electron density was found between Trp97 of chain *B* and Lys625 of symmetry-related chain *A*, probably corresponding to missing residues at the C-terminus of the neighbouring chain. It was left unmodelled.

### 2.8. UV–Vis spectrophotometry of *Ct*FDO crystals   

UV–Vis spectrophotometry data were measured on a *Ct*FDO_degl_ crystal of dimensions 150 × 150 × 50 µm without any ligand or potential substrate that was vitrified without cryoprotection. During the measurements, the crystal was cooled in a stream of nitrogen gas (100 K). For data collection, an HR2000+ES UV–Vis spectrophotometer (OceanOptics) at the MX-SpectroLab at BESSY II, Helmholz Zentrum, Berlin, Germany was used. The absorption spectra (200–1100 nm) were recorded by the *OceanView* spectroscopy software and were normalized (at two wavelengths: 290 and 900 nm) with *GraphPad Prism* version 7.02 for Windows (GraphPad Software, La Jolla, California, USA). The dark background was evaluated and subtracted at the beginning of data collection. The absorption spectra were collected before and after crystal irradiation by X-rays and compared. The crystal was irradiated with an X-ray beam with photon energy 13.4 keV and photon flux 5.7 × 10^11^ photons per second (beamline 14.2 at BESSY II) for 3 min, which corresponds, according to the available data, to a dose of the order of 1 MGy.

### 2.9. *Ct*FDO activity assay   

#### 2.9.1. Verification of *Ct*FDO oxidoreductase activity   

*Ct*FDO (the fully glycosylated form) at a concentration of 4 mg ml^−1^ (70 µl total volume) was reduced with 0.25 m*M* sodium dithionite (DTN) in 25 m*M* Tris–HCl pH 7.5, 100 m*M* NaCl. The reaction ran under aerobic conditions at 23°C for 16 min. The solution was stirred after 3 and 9 min. The re-oxidation effect was monitored in the wavelength range 280–600 nm with a Libra S22 UV–Vis spectrophotometer (Biochrom) in combination with the *Resolution Spectrophotometer* PC software (Biochrom). The spectra were buffer-subtracted.

#### 2.9.2. Activity assay based on a coupled reaction with horseradish peroxidase   

All of the activity tests below were performed with the fully glycosylated form of *Ct*FDO. The colorimetric assay for *Ct*FDO activity measurement was based on a coupled reaction with horseradish peroxidase (HRP; Sigma–Aldrich catalog No P8250; Nordkvist *et al.*, 2007 ▸). The reaction ran either in 40 m*M* Britton–Robinson buffer (boric acid, acetic acid and phosphoric acid adjusted with NaOH to pH 3.5–9.8) with 0.25 µg of *Ct*FDO at 45°C for 20 min or in 100 m*M* phosphate buffer with 30 m*M* NaCl pH 4.5–9 with 2 µg of *Ct*FDO at 37°C or 45°C for 30 min. The reactions were then cooled to room temperature for 3 min and the reaction mixture (11 m*M N*-ethyl-*N*-sulfopropyl-*m*-toluidine, 4 m*M* 4-aminoantipyrine and ∼6 U ml^−1^ HRP) was added in a 1:1 ratio.

The reaction of *Ct*FDO with 5 m*M* ABTS ran in 40 m*M* Britton–Robinson buffer (pH 4.5–8.9) with 0.5 µg of *Ct*FDO at 45°C for 20 min and at room temperature for 3 min. The absorbance of the product was measured at 420 nm using a CLARIOstar Monochromator Microplate Reader (BMG Labtech, Ortenberg, Germany). The substrates used in the reactions together with the details of particular reactions are summarized in Supplementary Table S2.

In addition, a liquid compost extract was prepared as a mixture of putative substrates. About 200 g of one-year-old compost was blended with 500 ml distilled water and incubated at 60°C for 30 min. The infusion was filtered via filter paper, a sterile syringe-filter unit (33 mm diameter) with a 0.22 µm pore-size hydrophilic polyethersulfone membrane (Millex) and a Nanosep 10K centrifugal device with molecular-weight cutoff 10 kDa (Pall Corporation). The reaction of 20 µl of the liquid extract with 2 µg of *Ct*FDO was run in Britton–Robinson buffer (pH 4.5, 6, 7.5 and 8) at 45°C for 30 min. The reactions were then cooled to room temperature for 3 min. The reaction mixture with HRP was then added in a 1:1 ratio. The absorbance of the resulting product was measured at 540 nm in a CLARIOstar Monochromator Microplate Reader (BMG Labtech).

#### 2.9.3. Activity assay based on coupled reaction with luciferase   

The high-throughput activity screening (HTS) of 990 selected compounds (molecular weights of 101–1550 g mol^−1^) was performed using the ROS-Glo H_2_O_2_ Assay (Promega). HTS was performed using a fully automated robotic cell::explorer HTS (Perkin Elmer) with an ECHO 550 integrated acoustic noncontact liquid handler (Labcyte, USA). The *Ct*FDO sample was dispensed into white solid polystyrene 1536-well microplates by a Multidrop Combi (Thermo Fisher Scientific). The luminescence signal was recorded on an Envision multimode plate reader (Perkin Elmer) equipped with an enhanced luminescence module. Data were collected, processed and normalized using the proprietary LIMS system ScreenX. A detailed description of the experiment is summarized in Supporting Information 2, including the list of tested compounds.

#### 2.9.4. Bioinformatic analysis and electrostatics calculations   

Sequence analysis and alignments were performed using the following software tools and servers: *BLAST* (Boratyn *et al.*, 2012 ▸), *PDBeFold* (Krissinel & Henrick, 2005 ▸) and *ESPript* 3.0 (Robert & Gouet, 2014 ▸). The protonation states were assigned using *PROPKA* (Søndergaard *et al.*, 2011 ▸) at pH 7 and pH 5.5 (the pH of the crystallization condition). The PQR parameter files were generated with the *PDB*2*PQR* pipeline using the AMBER force field (Dolinsky *et al.*, 2004 ▸) and reduced FAD (FADH^−^) parameters. The electrostatic potential distribution was calculated using the *Adaptive Poisson–Boltzman Solver* (*APBS*; Baker *et al.*, 2001 ▸) and visualized using the *APBS* plugin in *PyMOL* (Schrödinger). The van der Waals interactions between the ligands and *Ct*FDO_degl_ were analysed with *LIGPLOT* (Wallace *et al.*, 1995 ▸).

## 3. Results   

### 3.1. Expression and purification   

The enzyme was successfully expressed in *A. oryzae* and the resulting product was purified to homogeneity. The theoretical isoelectric point of 9.5 pointed to cation-exchange chromatography as ideal for purification, which indeed provided high purity after just a single purification step. The calculated molecular weight of 68 kDa was smaller than that observed by SDS–PAGE, where a smear at 80 kDa was observed, indicating protein glycosylation.

### 3.2. Biophysical characteristics of *Ct*FDO   

The identity of *Ct*FDO was verified by LC-MS/MS spectrometry, which confirmed 595 amino-acid residues and revealed 20 missing residues at the N-terminus (21 residues of the signal peptide are also not present) and eight residues at the C-terminus compared with the expected mature enzyme sequence (Supplementary Fig. S2). *Ct*FDO contains the Gly-*X*-Gly-*X-X-*Gly sequence motif (Gly32–Gly37) and C-terminal active-site histidine (His564) signatures of the GMC oxido­reductase superfamily (Supplementary Fig. S2; Romero & Gadda, 2014 ▸). *Ct*FDO is a monomer in solution, as was shown by mass photometry (Supplementary Fig. S3), and its molecular weight is about 85 kDa, which was also confirmed by MALDI-TOF spectra (Supplementary Fig. S4). The wide peak around 85 kDa in the MALDI-TOF spectra shows possible heterogeneity of glycosylation. Comparison of the spectrum with that of the deglycosylated form of *Ct*FDO (*Ct*FDO_degl_) shows a loss of about 17 kDa in mass upon deglycosylation (Supplementary Fig. S4). By subtracting the theoretical molecular mass of nonglycosylated *Ct*FDO (65 kDa, sequence confirmed by LC-MS/MS) from that of fully glycosylated *Ct*FDO, it follows that molecular mass of the oligosaccharide moieties is approximately 20 kDa.

The *Ct*FDO deglycosylation had an impact on the melting temperature, which decreased by 3.5°C for *Ct*FDO_degl_ compared with that of C*t*FDO (*T*_m1_ = 70.8°C) and had a weaker second peak in the nanoDSF melting curve (Supplementary Fig. S5). The FAD oxidation state (peaks around 390 and 460 nm) remained the same upon deglycosylation (Supplementary Fig. S6).

### 3.3. Crystal structure of ligand-free *Ct*FDO   

#### 3.3.1. Overall *Ct*FDO X-ray structure   

**Figure 1:**
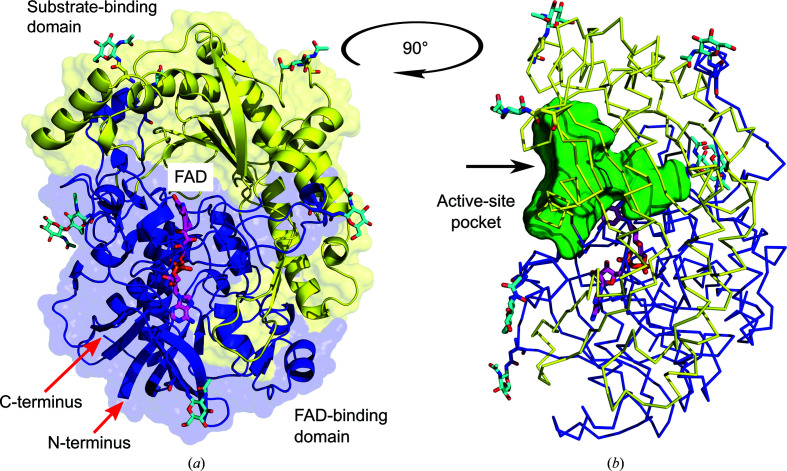
Crystal structure of *Ct*FDO_degl_. The cofactor and the *N*-acetyl-d-glucosamine units (occupied in at least one of the structures) are shown as sticks with magenta and cyan C atoms, respectively. (*a*) The yellow and blue secondary-structure elements represent the substrate-binding and the FAD-binding domains, respectively. (*b*) Side view of *Ct*FDO_degl_ shown as a C^α^ trace. The active-site pocket, as calculated with *HOLLOW* (Ho & Gruswitz, 2008 ▸), is displayed as a green surface. The black arrow marks the entrance to the pocket. The molecular graphics were created with *PyMOL* (Schrödinger).

The crystallized *Ct*FDO_degl_ is a monomer in solution with a molecular weight of around 68 kDa, as verified by several experimental methods (Supplementary Figs. S3, S4 and S7). The *Ct*FDO:free crystal structure has two *Ct*FDO_degl_ molecules (chains* A* and *B*) in the asymmetric unit, with an r.m.s.d. on C^α^ atoms of 0.12 Å. The amino-acid residue numbering corresponds to the complete expected mature enzyme sequence including the signal peptide (Supplementary Fig. S2). The N-terminal residues 42–45 and C-terminal residues 631–636 in both monomers lacked clear electron density and were not modelled. *Ct*FDO contains one disulfide bridge: a *trans* vicinal disulfide between two sequence-adjacent cysteine residues (Cys566 and Cys567; Richardson *et al.*, 2017 ▸). The *Ct*FDO_degl_ crystal structures confirmed the presence of six *N*-glycosylation sites in *Ct*FDO (at Asn114, Asn182, Asn197, Asn295, Asn374 and Asn543; Fig. 1 ▸). The seventh glycosylation site at Asn46 was structurally inconclusive, but was confirmed by MALDI-TOF peptide mass fingerprinting (Supplementary Fig. S8).

#### 3.3.2. The active site and FAD cofactor   

**Figure 2:**
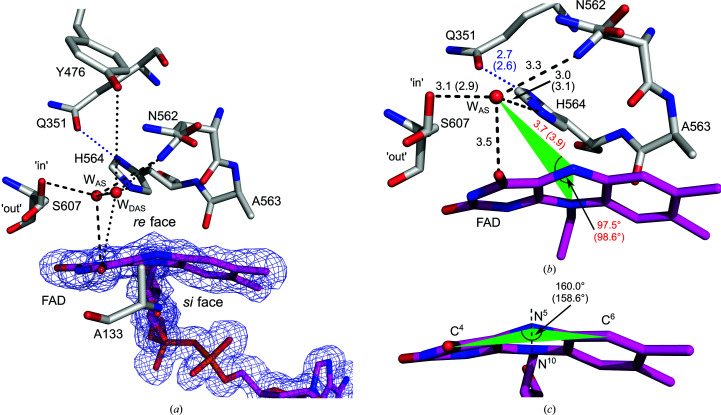
The catalytic site of *Ct*FDO:free (chain *A*). The cofactor, modelled in reduced form (magenta C atoms), is surrounded by the His564–Ser607 pair and additionally by Asn562, Ala563 and Tyr476 on its *re* face and by Ala133 on its *si* face. The orientation of the His564 imidazole ring is stabilized by a hydrogen bond to Gln351 (His564 N^δ1^–Gln351 O^ɛ1^, blue dotted line). Asn562 and Ser607 have two alternative conformations. The ‘in’ (nearest standard rotamer χ_1_ = −65°) and ‘out’ (χ_1_ = 64°) conformations of Ser607 are labelled. The active-site water molecules (W_AS_ and W_DAS_) binding in the catalytic site (black dashed and dotted lines, respectively) are labelled. W_AS_ binds between His564 N^ɛ2^, Ser607 O^γ^, Asn562 N^δ2^, FAD O^4^ and FAD N^3^ in the putative binding site of the catalytically modified group of the substrate. (*a*) A simulated-annealing 2*mF*_o_ − *DF*_c_ composite OMIT map (calculated in *Phenix*; Terwilliger, Grosse-Kunstleve, Afonine, Moriarty, Adams *et al.*, 2008 ▸) shown for the cofactor contoured at the 1σ level. (*b*) Labelled distances (differing values for chain *B* are given in parentheses) for W_AS_, which is at a distance of 3.7 Å (3.9 Å in chain *B*) from FAD N^5^ and makes an angle of 97.5° (98.6° in chain *B*) with the FAD N^10^–FAD N^5^ atoms, *i.e.* it occupies the site of oxidative attack in *Ct*FDO. (*c*) The isoalloxazine bend of 20.0° (21.4° in chain *B*) around the FAD N^5^ and FAD N^10^ axis (dashed line). The angle between the C^4^, N^5^ and C^6^ atoms is labelled (the value for chain *B* is given in parentheses). The molecular graphics were created with *PyMOL* (Schrödinger).

*Ct*FDO contains a wide-open active-site pocket with a funnel-like shape, with the FAD isoalloxazine ring reaching into the pocket from one side (Fig. 1 ▸ *b*). The cofactor is noncovalently bound to the apo­enzyme via hydrogen bonds to 13 surrounding residues (Ile56, Ser57, Glu77, Ala78, Val124, Gly128, Asn132, Ala133, Val135, Val273, Ile597, Ser607 and Met609; Supplementary Fig. S9) and indirectly via 12 water molecules. A composite OMIT map of the isoalloxazine ring confirms the nonplanar conformation of the ring with a 20.0° (21.4° in chain *B*) bend around the N^5^–N^10^ axis (measured for atoms C^4^—N^5^—C^6^), which indicates the reduced state of the cofactor (Fig. 2 ▸; Kao *et al.*, 2008 ▸). Absorption spectra of an unliganded *Ct*FDO_degl_ crystal measured before and after X-ray exposure (Supplementary Fig. S10) showed that FAD was oxidized before and reduced after exposure and that the reduction occurred during X-ray exposure. We expect that the form observed in the structures is the anionic fully reduced (FADH^−^) form of the cofactor.

*Ct*FDO contains four residues on the *re* face of the iso­alloxazine ring, namely Asn562, Ala563, His564 and Ser607 (Fig. 2 ▸). The position of His564 in *Ct*FDO corresponds to the conserved active-site histidine residue that plays the role of a catalytic base during the reductive half-reaction in the majority of GMC oxidoreductases (Hernández-Ortega *et al.*, 2012 ▸; Wongnate & Chaiyen, 2013 ▸; Smitherman *et al.*, 2015 ▸; Leskovac *et al.*, 2005 ▸; Graf *et al.*, 2015 ▸; Mugo *et al.*, 2013 ▸; Sygmund *et al.*, 2013 ▸). The orientation of the His564 imidazole ring is stabilized by a hydrogen bond to Gln351 (His564 N^δ1^–Gln351 O^ɛ1^). His564 is complemented by Ser607 located close to the pyrimidine moiety of the FAD isoalloxazine ring, thus creating a His–Ser pair. The His–Ser pair, Asn562 and the isoalloxazine ring bind water molecules in the *Ct*FDO:free structure (Fig. 2 ▸).

### 3.4. *Ct*FDO oxidoreductase activity and substrate specificity   

The observed spontaneous re-oxidation of *Ct*FDO (Supplementary Fig. S11) after chemical reduction by DTN indicated that *Ct*FDO is likely to use oxygen as an electron and proton acceptor during the oxidative half-reaction. Two types of activity assays were used to uncover the substrate of *Ct*FDO. Nevertheless, none of the tested compounds from various classes (carbohydrates, aliphatic and aromatic alcohols, aldehydes and vitamins) and neither lignin components nor compost extract showed significant activity with *Ct*FDO. Some compounds (glucose, cellobiose, pyridoxine, choline, benzyl alcohol, coniferyl alcohol, 4-methoxybenzyl alcohol and 4-hydroxy-3-methoxybenzyl alcohol) were also tested with *Ct*FDO_degl_ under similar conditions as for *Ct*FDO, with negative results.

### 3.5. Crystal structures of *Ct*FDO–ligand complexes   

A total of six structures of *Ct*FDO_degl_ complexes with ligands (Supplementary Fig. S12) were determined (Table 1 ▸). Despite marginal differences in the crystallization conditions used for the preparation of the complexes, polymorphism can be observed, as demonstrated by three different space groups: *P*2_1_2_1_2_1_ for *Ct*FDO:PESB, *Ct*FDO:4NC, *Ct*FDO:4NP and *Ct*FDO:ABTS, *P*2_1_2_1_2 for *Ct*FDO:MAMB and *P*2_1_ for *Ct*FDO:IPEA and *Ct*FDO:free. However, alignments of *Ct*FDO:free with the *Ct*FDO–ligand complexes show almost negligible differences in the overall structure (the C^α^ r. m. s. d. is 0.10–0.19 Å). The r.m.s.d. of C^α^ atoms between chains *A* and *B* within one asymmetric unit ranges between 0.12 and 0.19 Å (*PDBeFold*; Krissinel & Henrick, 2005 ▸).

#### 3.5.1. The binding subsites of the active-site pocket   

**Figure 3:**
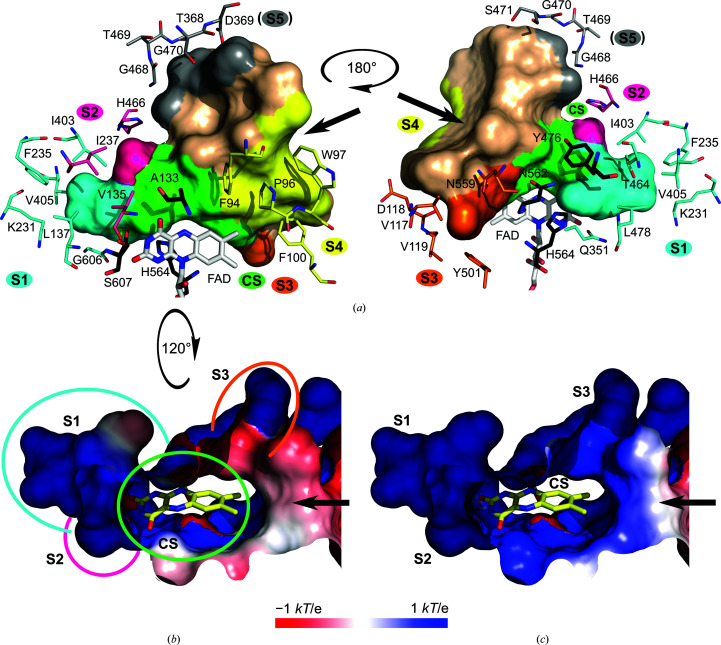
The active-site pocket of *Ct*FDO. The black arrows show the entrance to the pocket. (*a*) The view from two sides of the pocket in surface representation with a highlighted catalytic site (CS; green) and five subsites S1 (cyan), S2 (hot pink), S3 (orange), S4 (yellow) and the hypothetical S5 (grey). Selected surrounding residues are shown as sticks with C atoms in colours corresponding to the subsites. The residues shown with C atoms in black (Ala133, Tyr476, Asn562, His564 and Ser607) form the catalytic site. The FAD cofactor is shown with C atoms in white. The tunnel calculation was performed with *HOLLOW* (Ho & Gruswitz, 2008 ▸). The molecular graphics were created using *PyMOL* (Schrödinger). (*b*, *c*) Electrostatic potential distribution represented on the solvent-accessible surface of the active-site pocket of *Ct*FDO at neutral pH 7 (*b*) and pH 5.5, corresponding to the crystallization condition (*c*). The catalytic site and subsites in (*b*) are marked by circles in colours corresponding to (*a*). The flavin has yellow C atoms. The electrostatic potential distribution was calculated using the *Adaptive Poisson–Boltzman Solver* (*APBS*; Baker *et al.*, 2001 ▸) and visualized using the *APBS* plugin in *PyMOL* (Schrödinger).

The complexes suggest that the individual compounds utilize five binding subsites: the catalytic site (CS) and four other subsites marked S1–S4 (Fig. 3 ▸). The CS is placed in the narrow passage of the pocket created by the FAD isoalloxazine ring, Ala133, Tyr476, Asn562, Ala563, His564 and Ser607 (Fig. 2 ▸). The residues of the CS occasionally occur in two alternative conformations adapting to the bound compound. Of these, Ser607 was modelled in two conformations, a conformer near to the standard rotamer χ_1_ = −65° (the ‘in’ conformer) and a conformer near to χ_1_ = 64° (the ‘out’ conformer), in the majority of the *Ct*FDO_degl_ structures (apart from *Ct*FDO:4NC and *Ct*FDO:ABTS). One exception is His564, which is always modelled in one conformation enforced by the stabilizing hydrogen bond with Gln351. The active-site pocket of *Ct*FDO:free is occupied by water molecules, two of which are in the CS (Fig. 2 ▸). The first one, the active-site water molecule (W_AS_), was found bridging four atoms: His564 N^ɛ2^ (3.0 Å; 3.1 Å in chain *B*), Ser607 O^γ^ (‘in’ conformer, 3.1 Å; 2.9 Å in chain *B*), Asn562 N^δ2^ (3.3 Å) and FAD O^4^ (3.5 Å). The second water molecule, which is more distant from the isoalloxazine ring (W_DAS_), is bound between W_AS_ (2.4 Å; 2.6 Å in chain *B*), Asn562 N^δ2^ (3.5 Å; 3.4 Å in chain *B*), FAD O^4^ (3.6 Å; 3.8 Å in chain *B*) and Tyr OH (3.5 Å in chain *B*, 4.7 Å in chain *A*) (Fig. 2 ▸ *a*). In the *Ct*FDO–ligand complexes, W_DAS_ is always replaced by a ligand.

Subsite 1 (S1) is the deepest part of the active-site pocket; it is partially separated by the residues of the CS, creating a cavity (Fig. 3 ▸ *a*). S1 is formed mainly by hydrophobic residues (Val135, Leu137, Lys231, Ile237, Gln351, Ile403, Val405, Thr464, His466, Leu474, Leu478 and Gly606) and has significantly positive electrostatic potential at neutral pH (Fig. 3 ▸ *b*). Subsite 2 (S2) is placed nearby and is formed by Val135, Ile237 and His466. Two remaining subsites are located at the edge of the tunnel. Subsite 3 (S3) is a shallow cavity established by Val117–Ile120, Tyr501 and Asn559–Ser561; subsite 4 (S4) is lined by the aromatic residues Phe94, Trp97 and Phe100 and by Pro96. In addition to S1–S4, another subsite (S5) potentially available for substrate binding can be identified. It is located at the entrance to the active-site pocket, formed by Thr368, Asp369 and the Gly468–Ser471 peptide, offering a number of hydrophilic contacts for interaction. In the current structures it is filled by water.

#### 3.5.2. Specific features of *Ct*FDO–ligand complexes   

**Figure 4:**
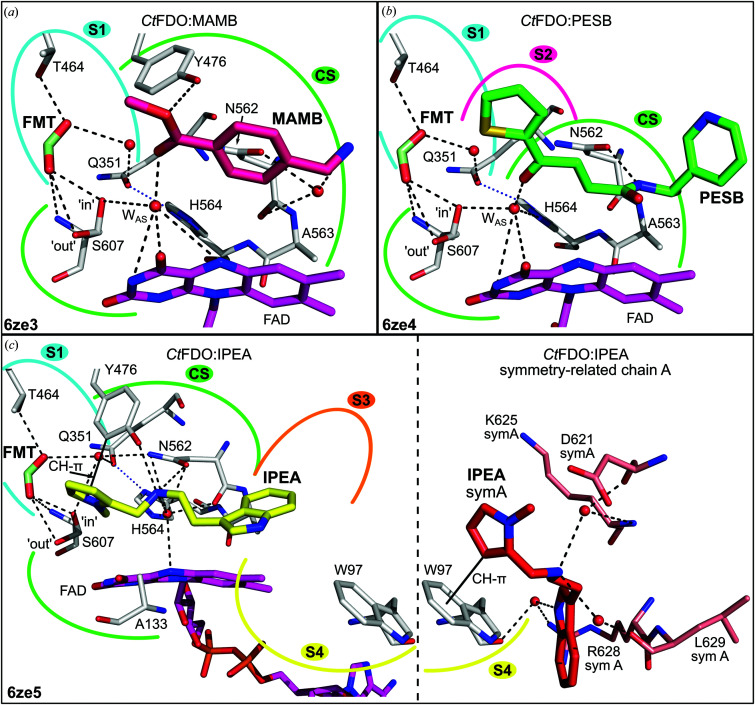
The active-site pockets of the *Ct*FDO:MAMB, *Ct*FDO:PESB and *Ct*FDO:IPEA complexes (chains *A*). The PDB code for each structure is shown in the bottom left corner. Residues involved in ligand interaction and the FAD cofactor are shown as sticks (C atoms in light grey and magenta, respectively). Water molecules mediating ligand–*Ct*FDO contacts are shown as red spheres. The active-site water molecule is labelled W_AS_. Selected interactions are shown as black dashed or full lines. The blue dotted line indicates the hydrogen bond His564 N^δ1^–Gln351 O^ɛ1^ stabilizing the position of the His564 indole ring. The green, cyan, hot pink, orange and yellow curves represent the catalytic site (CS) and subsites S1, S2, S3 and S4, respectively. (*a*) Interactions of MAMB (hot pink C atoms) and formic acid (FMT; pale green C atoms) in the *Ct*FDO:MAMB complex. (*b*) Interactions of PESB (green C atoms) and FMT (pale green C atoms) in the *Ct*FDO:PESB complex. (*c*) Interactions of the FMT (pale green C atoms) and IPEA (yellow C atoms) molecules binding in the active-site pocket of chain *A* in the *Ct*FDO:IPEA complex and the additional binding variant second IPEA molecule (red C atoms) at the interface of the symmetry-related chain *A* (symA, pink C atoms) with chain *A*. The figures were created with *PyMOL* (Schrödinger).

In the *Ct*FDO:MAMB complex (PDB entry 6ze3; Fig. 4 ▸ *a*, Supplementary Fig. S13), *Ct*FDO_degl_ binds one MAMB molecule in the CS and one molecule of formic acid (from the crystallization condition) in S1. The position of the active-site water W_AS_ (HOH_A836_) is similar to that in *C*tFDO:free (shifted by 0.7 Å). MAMB is hydrogen-bonded to Tyr476 and, via W_AS_, to flavin. One additional water mediates the contact of MAMB with Asn562 (Supplementary Table S3). MAMB is further stabilized by van der Waals (vdW) interactions with the flavin, Ala133, Leu399, Tyr476 and Asn562. The formic acid molecule interacts via hydrogen bonds to Thr464, Ser607 in both conformers and, via a water molecule, to Gln351.

In the *Ct*FDO:PESB complex (PDB entry 6ze4; Fig. 4 ▸ *b*, Supplementary Fig. S14), each protein chain binds one PESB molecule (with the thiophene moiety in subsite S2 and the aliphatic moiety in the CS), one formic acid (subsite S1) and the active-site water W_AS_ in the active-site pocket. The formic acid molecule makes the same interactions in S1 as in *Ct*FDO:MAMB, and W_AS_ (HOH_A804_ in chain *A*, HOH_B817_ in chain *B*) adopts an almost identical position as W_AS_ in *Ct*FDO:free (shifted by 0.2 and 0.5 Å for chains *A* and *B*, respectively). PESB interacts via a hydrogen bond with Asn562 and via W_AS_ with flavin, His564 and Ser607 (‘in’ conformer; Supplementary Table S3). PESB is further stabilized by vdW interactions with FAD, Ala133, Val135, Leu399, His466, Leu474, Tyr476 and Asn562.

In the *Ct*FDO:IPEA complex (PDB entry 6ze5; Fig. 4 ▸ *c*, Supplementary Fig. S15), each protein chain binds one IPEA molecule (with the pyrrole and aliphatic moieties in the CS and the indole moiety in subsite S4) and one formic acid molecule (subsite S1) in the active-site pocket. Formic acid binds in S1 similarly as in *Ct*FDO:MAMB, with an additional water-mediated bond to Asn562. IPEA interacts with Asn562 and Tyr476 directly via hydrogen bonds and also with Asn562 via a water molecule. The pyrrole moiety of IPEA is stabilized by Tyr476 via a CH–π interaction. IPEA also makes vdW interactions with the flavin, Phe94, Pro96, Ile120, Ala133, Val135, Ile403, Tyr476, Asn562, His564 and Ser607 (Supplementary Table S3). The IPEA C atom replaces the active-site water in the CS.

An additional IPEA molecule is bound to the Asp621–Arg628 peptide of chain *A*. Due to the crystal contact with the symmetry-related chain *A*, a binding variant to the Trp97–Leu98 peptide of the symmetry-related chain *A* occurs. IPEA establishes a CH–π interaction with the indole moiety of Trp97, water-mediated hydrogen bonds with Trp97, Asp621, Lys625, Arg628 and Leu629, and vdW interactions with Trp97, Leu98, Asp621, Lys624, Lys625, Arg628 and Leu629.

**Figure 5:**
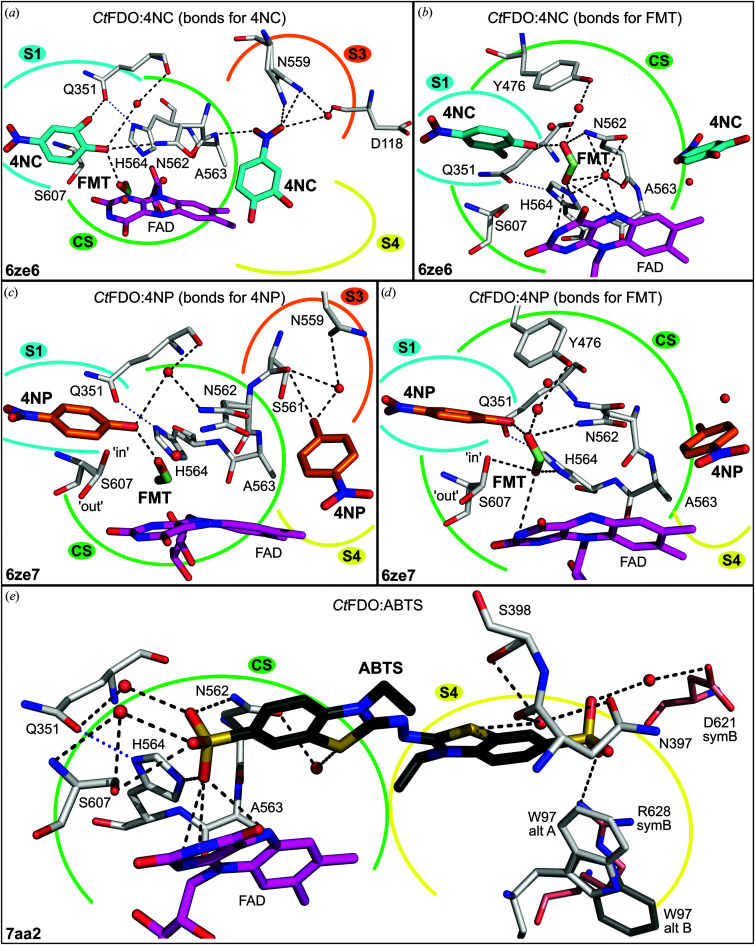
The active-site pockets of the *Ct*FDO:4NC, *Ct*FDO:4NP and *Ct*FDO:ABTS complexes (chains *A*) displayed in the same style as in Fig. 4 ▸. The figures were created in *PyMOL* (Schrödinger). (*a*, *b*) Interactions of two molecules of 4NC (cyan C atoms) and formic acid (FMT; pale green C atoms) in the *Ct*FDO:4NC complex, respectively. (*c*, *d*) Interactions of two molecules of 4NP (orange C atoms) and FMT (pale green C atoms) in the *Ct*FDO:4NP complex, respectively. (*e*) Interactions of ABTS (black C atoms) in the *Ct*FDO:ABTS complex. Asp621 and Arg628 belong to symmetry-related chain *B* (symB, pink C atoms). Residue Trp97 was modelled in two alternative conformations: alt A (light grey C atoms) and alt B (grey C atoms).

In the *Ct*FDO:4NC complex (PDB entry 6ze6; Figs. 5 ▸ *a* and 5 ▸ *b*, Supplementary Fig. S16), both *Ct*FDO_degl_ chains bind two molecules of 4NC, one in subsite S1 and another in subsite S3, and one molecule of formic acid in the CS, where it replaces the active-site waters. The former 4NC molecule is stabilized mainly via vdW interactions (Leu137, Lys231, Phe235, Gln351, Ile403, Val405, Thr464, Tyr476, Leu478, Asn562, His564, Gly606 and Ser607) and via a hydrogen bond to Gln351 and Asn562. The latter 4NC is hydrogen-bonded to Ser561, Ala563 and Asn559, and it interacts via a water molecule with Ser561, Gln565 and Ile120. Its position is also stabilized by vdW interactions with Phe100, Ile120, Ser561, Asn562, Ala563, Asn559 and flavin (Supplementary Table S3). The position of the aromatic ring of the latter 4NC corresponds to that of the indole moiety of IPEA in *Ct*FDO:IPEA. A formic acid molecule was modelled in the CS with hydrogen bonds to flavin, 4NC (in S1), Asn562 and His564.

In the *Ct*FDO:4NP complex (PDB entry 6ze7; Figs. 5 ▸ *c* and 5 ▸ *d*, Supplementary Fig. S17), both monomers of the *Ct*FDO:4NP structure bind one 4NP in subsite S1, where it adopts the same position as 4NC in *Ct*FDO:4NC and is stabilized by the same vdW interactions, except for that with Gln351. 4NP is further hydrogen-bonded to Asn562 and makes water-mediated interactions with Gln351. Two alternative conformations of Ser607 in *Ct*FDO:4NP are enabled by the decreased occupancy of 4NP (0.8). In chain *A*, the second 4NP molecule is bound in the entrance to the active-site pocket (its hydroxyl group is in S3 and its nitro group is in S4). The aromatic ring of 4NP corresponds to the same position as 4NC and the indole moiety of IPEA in *Ct*FDO:4NC and *Ct*FDO:IPEA, respectively. It establishes a hydrogen bond to Ser561 and, via a water molecule, further interacts with Asn559. It is also stabilized by vdW interactions with Phe94, Pro96, Ile120, the Ser561–Ala563 peptide and the flavin (Supplementary Table S3). In a similar position as in chain *A* of *Ct*FDO:4NP, a weak peak of electron density, likely for the same ligand, was found in chain *B* but was left uninterpreted. Also, as in *Ct*FDO:4NC, a molecule of formic acid was modelled in the CS with hydrogen bonds to Ser607 (the ‘in’ conformer), 4NP (in S1), His564 and flavin, and interacting via a water molecule with Tyr476.

In the *Ct*FDO:ABTS complex (PDB entry 7aa2; Fig. 5 ▸ *e*, Supplementary Fig. S18), each monomer binds one ABTS molecule in the active-site pocket. One ABTS sulfate moiety binds in the CS, where it replaces the active-site water. Considering the FADH^−^ state of the cofactor, the SO_3_ group in CS is likely to be protonated at O^47^. The other sulfate of ABTS, together with the benzothiazoline moiety, uses subsite S4 for binding. ABTS is hydrogen-bonded to flavin, Asn562, His564, Ser607 and to Arg628 of a symmetry-related chain *B*. It further interacts via a water molecule with Gln351, Asn397, Ser398, Asn562, Ser607 and with Asp621 of a symmetry-related chain *B*. ABTS is also stabilized by vdW interactions with Phe94, Pro96, Trp97, Ala133, Val135, Ser398, Leu399, Tyr476, Asn562, His564, Ser607 and flavin (Supplementary Table S3).

## 4. Discussion   

### 4.1. *Ct*FDO and other GMC oxidoreductases   

Three-dimensional analysis performed on the *Ct*FDO:free structure (secondary-structure matching in *PDBeFold*; Krissinel & Henrick, 2005 ▸) revealed a number of oxidoreductases with similar structures (C^α^ r.m.s.d.s of around 1.7 Å and sequence identities of around 30%). The following five structures with similar features were selected as representatives for a detailed comparison: (i) aryl-alcohol oxidase from *Thermothelomyces thermophilus* (*Mt*AAO; PDB entry 6o9n; Kadowaki *et al.*, 2020 ▸; r.m.s.d. of 1.79 Å for 559 aligned C^α^ atoms; sequence identity 37%), (ii) glucose dehydrogenase from *Aspergillus flavus* (*Af*GDH; PDB entry 4ynt; Yoshida *et al.*, 2015 ▸; r.m.s.d. of 1.58 Å on 523 C^α^ atoms; sequence identity 29%), (iii) glucose oxidase from *A. niger* (*An*GOX; PDB entry 1cf3; Wohlfahrt *et al.*, 1999 ▸; r.m.s.d. of 1.61 Å on 521 C^α^ atoms; sequence identity 30%), (iv) glucose oxidase from *Penicillium amagasakiense* (*Pa*GOX; PDB entry 1gpe; Wohlfahrt *et al.*, 1999 ▸; r.m.s.d. of 1.72 Å on 523 C^α^ atoms; sequence identity 28%) and (v) aryl-alcohol oxidase from *Pleurotus eryngii* (*Pe*AAO; PDB entry 3fim; Fernández *et al.*, 2009 ▸; r.m.s.d. of 1.67 Å on 508 C^α^ atoms, sequence identity 28%).

**Figure 6:**
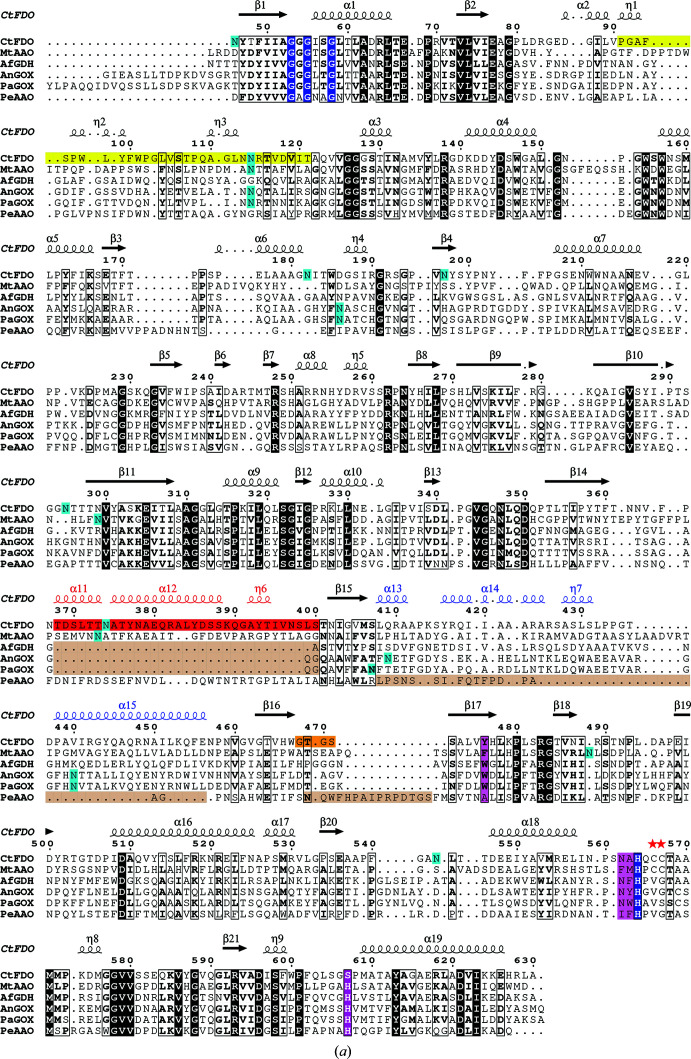

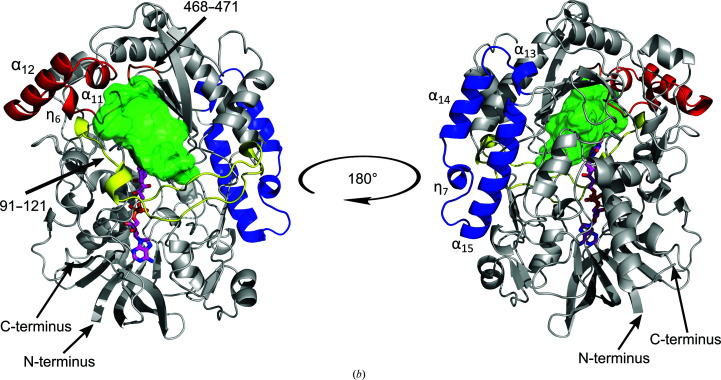
Structure-based sequence alignment and comparison of structural elements of *Ct*FDO with the most similar structures of GMC oxidoreductases. (*a*) Structure-based sequence alignment of *Ct*FDO (PDB entry 6ze2, chain *A*) with *Mt*AAO (PDB entry 6o9n), *Af*GDH (PDB entry 4ynt), *An*GOX (PDB entry 1cf3), *Pa*GOX (PDB entry 1gpe, chain *A*) and *Pe*AAO (PDB entry 3fim) according to *PDBeFold* (Krissinel & Henrick, 2005 ▸). Black and blue backgrounds show invariant residues of these six enzymes and the conserved motifs (Gly-*X*-Gly-*X*-*X*-Gly sequence motif and the conserved histidine residue) indicative of GMC oxidoreductases, respectively. The second residue of the His–His/His–Ser pair is shown in white letters on a magenta background. A pink background denotes other residues present in the active site. The parts of the sequence shown on a light brown background correspond to the main secondary-structure differences between the compared enzymes and *Ct*FDO. The extra secondary-structure elements of *Ct*FDO are coloured red and blue. The yellow, red and orange colours mark the secondary-structure elements of the wide-open access to the active site in *Ct*FDO. The confirmed *N*-glycosylation sites in *Ct*FDO and structurally confirmed *N*-glycosylation sites in *Mt*AAO, *Af*GDH and *An*GOX are marked by cyan boxes. The vicinal disulfide in *Ct*FDO is marked by red stars. The graphics were created in *ESPript* (Robert & Gouet, 2014 ▸). (*b*) Secondary-structure representation of *Ct*FDO:free with highlighted structural motifs as marked in the structure-based sequence alignment. The active-site pocket is shown in surface representation (green; calculated by *HOLLOW*; Ho & Gruswitz, 2008 ▸). The FAD cofactor is represented in sticks with C atoms coloured magenta. Molecular graphics were created with *PyMOL* (Schrödinger).

*Ct*FDO, *Mt*AAO, *Af*GDH, *An*GOX and *Pa*GOX share the short version of the loop between β_16_ and β_17_ in *Ct*FDO (residues 468–471) and the helical extension, composed of helices α_13_–α_15_, in *Ct*FDO (residues 407–457), in contrast to the majority of GMC oxidoreductases, including *Pe*AAO (Fig. 6 ▸). *Ct*FDO and *Mt*AAO also share the helical insertion in the substrate-binding domain (helices α_11_, α_12_ and η_6_) in *Ct*FDO (residues 367–400), which is also present in *Pe*AAO and missing in *Af*GDH, *An*GOX and *Pa*GOX (Fig. 6 ▸). The loop 468–471 and the helical insertion α_11_–α_12_–η_6_, together with the loop 91–121, form the entrance to the *Ct*FDO active-site pocket. *Ct*FDO, *Mt*AAO, *An*GOX and *Pa*GOX match with respect to the three-dimensional positions of the glycosylation sites at Asn114, Asn107, Asn89 and Asn93, respectively. Whereas the carbohydrates at Asn89 and Asn93 contribute to dimer stabilization in *An*GOX and *Pa*GOX, dimer formation by *Ct*FDO and *Mt*AAO was not observed (Yoshida *et al.*, 2015 ▸).

*Ct*FDO contains one disulfide bridge: a *trans* vicinal disulfide formed by two neighbouring cysteine residues (Cys566 and Cys567). It is located near the conserved His564 and probably stabilizes the local protein geometry and interactions (Richardson *et al.*, 2017 ▸). The same sequence motif containing a *trans* vicinal disulfide at the same position was found in *Mt*AAO (His579-*X*-Cys581-Cys582).

### 4.2. Character of the active-site pocket   

GMC oxidoreductases often feature a narrow tunnel or cleft leading to the active site (Mugo *et al.*, 2013 ▸; Fernández *et al.*, 2009 ▸; Dijkman *et al.*, 2015 ▸; Hallberg *et al.*, 2002 ▸; Salvi *et al.*, 2014 ▸). The active site of *Ct*FDO is much more open to the exterior (Fig. 1 ▸ *b*) and potentially can accommodate molecules larger than 500 Da, as confirmed by the *Ct*FDO:ABTS complex (the molecular weight of ABTS is 514.6 Da). Such a feature has so far only been described for *Mt*AAO, where the wide-open entrance to the catalytic site is associated with a similar enzymatic activity for small and bulky substrates (Kadowaki *et al.*, 2020 ▸). The entrance to the active site in *Ct*FDO is partially formed by the abovementioned short loop 468–471 between the two antiparallel β-strands (β_16_ and β_17_) in the large six-stranded antiparallel β-sheet of the substrate-binding domain (Fig. 6 ▸). In the majority of GMC oxido­reductases the loop is usually much longer and covers the active site. Moreover, it can adopt different conformations during the catalytic cycle, as described for *Trametes multicolor* pyranose 2-oxidase (P2O; Spadiut *et al.*, 2010 ▸). The *B*-factor distribution in the *Ct*FDO_degl_ structures does not indicate any significantly flexible loops, except for the N- and C-termini. Therefore, we do not suggest any motion on the level of secondary-structure elements in *Ct*FDO, such as loop rearrangement, related to substrate entry and/or product release.

As *Ct*FDO originates from a cellulose-degrading fungus, and given its structural similarity to GMC homologues, we had expected that *Ct*FDO would act on lignocellulosic components. Nevertheless, this initial assumption could not be confirmed by activity tests with several of these components (Supplementary Table S2). A subsequent extended activity test, together with our high-throughput activity screening with various types of compounds of molecular masses from 70 to 1550 Da (Supplementary Table S2, Supporting Information 2), yielded no conclusive substrate. Therefore, we performed crystallo­graphic fragment screening (using about 80 compounds and fragments) to analyse the binding sites in *Ct*FDO and to shed light on the composition of a putative substrate. Over 130 diffraction data sets with diffraction limits of 1.2–3.3 Å were processed and analyzed. 10% of the solved structures were complexes binding a ligand in the active site of *Ct*FDO (six unique *Ct*FDO–ligand complexes), while the remaining 90% of the structures were without any bound ligand (except for formic acid). Cystamine, which was used for *Ct*FDO complex preparation by co-crystallization (Supplementary Table S1), was found to improve the diffraction quality of the *Ct*FDO_degl_ crystal, but without any electron density being found for the compound. The structure is presented as the ligand-free structure of *Ct*FDO_degl_ and is referred to as the *Ct*FDO:free structure.

The *Ct*FDO–ligand complexes revealed five binding sub­sites in the *Ct*FDO active-site pocket: the catalytic site (CS) and subsites S1–S4. A fifth hypothetical binding subsite (S5) at the entrance to the active-site pocket is postulated, created mainly by the hydrophilic residues Thr368 and Asp369 and the Gly468–Ser472 peptide.

Different moieties of the bound compounds use the individual subsites differently. The CS, which is located in the narrow passage of the pocket, binds both aromatic (MAMB, the pyrrole of IPEA and the benzothiazoline of ABTS) and aliphatic (formic acid, PESB and IPEA) moieties (Figs. 4 ▸ *a*, 4 ▸ *b*, 4 ▸ *c* and 5 ▸ *e*). Subsite S1, with the shape of a small cavity created mainly by hydrophobic residues, binds small inorganic molecules (such as formic acid) and water with Ser607 in the ‘in’ conformation, and small aromatic molecules or moieties (such as 4NC) with the ‘out’ conformation of Ser607. Such a subsite had not previously been described in GMC oxidoreductases. Nevertheless, detailed analysis of their structures shows a similar cavity in 5-hydroxymethylfurfural oxidase from *Methylovorus* sp. (HMFO; PDB entry 4udp) located in a similar position as in *Ct*FDO, without any notes by the authors (Dijkman *et al.*, 2015 ▸).

Subsite S2 of *Ct*FDO was found to bind only aromatic moieties (the thiophene moiety of PESB). S3 binds the indole part of IPEA, the nitro group of 4NC and the hydroxyl of 4NP. Subsite S4 binds the benzothiazoline of ABTS, the nitro group of 4NP and the indole part of IPEA (from the symmetry-related chain *A*). Residue Trp97 belonging to S4 has slightly higher *B*-factor values compared with the neighbouring residues and the overall structure averages (Table 2 ▸) among all *Ct*FDO structures. The increased mobility of Trp97 was confirmed by the *Ct*FDO:ABTS structure, in which it is present in two alternative conformations, the first (nearest standard rotamer χ_1_ = 58°) accommodating the bound benzothiazoline moiety of ABTS in subsite S4 and the second (χ_1_ = 60°) observed in all *Ct*FDO structures. Trp97 is involved in the creation of crystal contacts to a symmetry-related *Ct*FDO_degl_ molecule and participates in the binding of the polyethylene unit (triethylene glycol) in *Ct*FDO:4NP. Probably due to crystal contacts, Trp97 also participates in binding the IPEA molecule from the symmetry-related chain in *Ct*FDO:IPEA via CH–π interactions. In *Ct*FDO:4NC, a cloud of electron density (*mF*_o_ − *DF*_c_, not interpreted) was observed at the interface of the Trp97 indole moiety (chain *A*). This peak indicates possible ligand binding via stacking interactions, but could be also interpreted as localization of the C-terminus of the neighbouring chain. It is likely that Trp97 could serve in the binding of the aromatic moieties of larger substrates.

### 4.3. Catalytic site and the active-site water molecule   

The unliganded structures of GMC oxidoreductases usually bind a water molecule in the catalytic site, located in the active-site pocket between the flavin and the active-site residues (Fernández *et al.*, 2009 ▸; Kadowaki *et al.*, 2020 ▸; Wohlfahrt *et al.*, 1999 ▸; Koch *et al.*, 2016 ▸; Dreveny *et al.*, 2009 ▸; Doubayashi *et al.*, 2011 ▸; Bannwarth *et al.*, 2004 ▸). Its position corresponds to the site of oxidative attack, which in flavoenzymes is typically located at a distance of about 3.5–3.8 Å from FAD N^5^ on the FAD *re* face, making an angle of about 96–117° with the FAD N^10^–FAD N^5^ atoms (Fraaije & Mattevi, 2000 ▸). The alignment of the unliganded structures and complexes with products and substrate analogues shows that the active-site water molecule binds in close proximity to the expected position of the reactive part of the corresponding substrate in *Pe*AAO (PDB entries 3fim and 5oc1; Carro *et al.*, 2017 ▸), pyridoxine 4-oxidase (PNOX; PDB entries 3t37 and 4ha6; Mugo *et al.*, 2013 ▸), *Af*GDH (PDB entries 4ynt and 4ynu; Yoshida *et al.*, 2015 ▸) and choline oxidase (PDB entries 2jbv and 4mjw; Quaye *et al.*, 2008 ▸; Salvi *et al.*, 2014 ▸). Similarly, in *Pichia pastoris* alcohol oxidase, FAD-dependent hydroxynitrile lyase from *Prunus amygdalus* and *Aspergillus oryzae* formate oxidase (FOD) the active-site water molecule is likely to mimic the binding position of the formaldehyde O atom, cyanide and formate, respectively (Koch *et al.*, 2016 ▸; Dreveny *et al.*, 2009 ▸; Doubayashi *et al.*, 2011 ▸).

Similarly to GMC oxidoreductases, the active site of *Ct*FDO:free is occupied by an active-site water molecule (W_AS_) coordinated by the atoms His564 N^ɛ2^, Ser607 O^γ^, Asn562 N^δ2^ and FAD O^4^ (Fig. 2 ▸). Its distance to FAD N^5^ is 3.7 Å (3.8 Å in chain *B*) and it makes an angle with the FAD N^10^–FAD N^5^ atoms of about 97.5° (98.6° in chain *B*), which corresponds to the site of oxidative attack as defined for flavoenzymes. In the *Ct*FDO complexes, W_AS_ is usually displaced by a ligand [by formic acid in *Ct*FDO:4NC and *Ct*FDO:4NP (Figs. 5 ▸ *a*–5 ▸ *d*), by the IPEA C atom in *Ct*FDO:IPEA (Fig. 4 ▸ *c*) and by the sulfate moiety in *Ct*FDO:ABTS (Fig. 5 ▸ *e*)] or mediates the contact of the ligand carbonyl group (MAMB O^1^ in *Ct*FDO:MAMB and PESB O^1^ in *Ct*FDO:PESB) with flavin and the active-site His564–Ser607 pair (Figs. 4 ▸ *a* and 4 ▸ *b*). Based on this, we expect that the position of W_AS_ represents the catalytic centre of *Ct*FDO.

### 4.4. FAD cofactor   

The FAD cofactor was found to be bent in all *Ct*FDO crystal structures. Absorption spectra measured with an un­liganded *Ct*FDO_degl_ crystal showed that the FAD cofactor was reduced during X-ray exposure (Supplementary Fig. S10). Therefore, we expect the FADH^−^ state of the cofactor in all of our *Ct*FDO_degl_ structures. A significant experimental effort was made to obtain both the oxidized state (by decreasing the X-ray dose in combination with soaking crystals in a solution with a higher pH) and the purposefully reduced state of FAD (by chemical reduction of the *Ct*FDO_degl_ crystals) in the *Ct*FDO structure to observe flavin structural changes, but without any success. The FAD isoalloxazine ring in *Ct*FDO is anchored via direct hydrogen bonds between the FAD pyrimidine moiety and the main chains of Ala133, Val135, Met609 and the ‘out’ conformer of Ser607 and via water-mediated hydrogen bonds to the main chains of Ala610, Leu137 and Ser607 (Supplementary Fig. S19). The remaining dimethylbenzene moiety of the isoalloxazine lacks specific interactions with the protein. From the current data, however, it is not clear how changes in the FAD oxidation state are manifested structurally.

### 4.5. Residues of the catalytic site   

The structural, mechanistic and computational studies show that the conserved active-site histidine is likely to play the role of the catalytic base deprotonating the hydroxyl group of a substrate in *An*GOX, *Pe*AAO, HMFO and other GMC enzymes (Hernández-Ortega *et al.*, 2012 ▸; Wongnate & Chaiyen, 2013 ▸; Smitherman *et al.*, 2015 ▸; Leskovac *et al.*, 2005 ▸; Mugo *et al.*, 2013 ▸; Graf *et al.*, 2015 ▸; Sygmund *et al.*, 2013 ▸; Dijkman *et al.*, 2015 ▸). In *Ct*FDO, the position of the active-site histidine is occupied by His564 (Fig. 2 ▸ *a*). Other semi-conserved active-site residues in the GMC oxidoreductase superfamily are histidine or asparagine residues, which together with the conserved His create a His–His or His–Asn pair at the catalytic site. Two related enzymes with different pairs again have been described to date: PNOX (PDB entry 3t37) and FOD (PDB entry 3q9t), with His–Pro and His–Arg pairs, respectively (Mugo *et al.*, 2013 ▸; Doubayashi *et al.*, 2011 ▸). The second residue in the pair is likely to play the minor role of a residue that hydrogen-bonds the alcohol group of the substrate in the correct position in *Pe*AAO, P2O, PNOX and others (Mugo *et al.*, 2013 ▸; Hernández-Ortega *et al.*, 2012 ▸; Wongnate *et al.*, 2011 ▸; Rotsaert *et al.*, 2003 ▸; Graf *et al.*, 2015 ▸). Unconventionally, *Ct*FDO contains a His–Ser (His564–Ser607) pair in the catalytic site, which is present at the same position as the His–His, His–Asn, His–Pro or His–Arg pair. A sequence-similarity search in *BLAST* (Boratyn *et al.*, 2012 ▸) revealed three other sequences of uncharacterized GMC proteins from fungal species (UniProt IDs A0A175W6J3, B2AMU4 and A0A447BYY0; sequence identity of ∼50–70% with *Ct*FDO) that contain a His–Ser pair in the active site.

Ser607 is in two conformations in the majority of the presented *Ct*FDO structures: ‘in’ and ‘out’. Ser607 in the ‘in’ conformation participates in binding the active-site water molecule (*Ct*FDO:free and *Ct*FDO:PESB), formic acid in the S1 subsite (*Ct*FDO:MAMB, *Ct*FDO:PESB and *Ct*FDO:IPEA) and in the catalytic site (*Ct*FDO:4NP). The ‘in’ conformation of Ser607 excludes the binding of small aromatic moieties to subsite S1 as in *Ct*FDO:4NC. The ‘out’ conformation only was also modelled in the case of *Ct*FDO:ABTS. In *Ct*FDO:4NP, both conformations are observed due to the reduced occupancy of 4NP in subsite S1. To the best of our knowledge, Ser607 is the first residue type at this position that was observed with the side chain in two significantly different conformations. We assume that the ‘in’ conformation of Ser607 may specifically hydrogen-bond to substrate, similarly as observed for His and Asn in this position in other enzymes. It is also possible that the ‘out’ conformer could participate in binding of the first substrate (donor of electrons and protons) to the CS with concurrent binding to subsite S1, and the ‘in’ conformer would be then involved in binding of the second substrate, an oxygen molecule.

On the FAD *re* face, the His–Ser pair is complemented by Asn562 and Tyr476. An asparagine residue at the same spatial position as Asn562 is also present in *Af*GDH (Asn503), *An*GOX (Asn514) and *Pa*GOX (Asn518), where it is or is likely to be involved in ligand binding (Yoshida *et al.*, 2015 ▸; Wohlfahrt *et al.*, 1999 ▸). A tyrosine residue is located at the same position as Tyr476 in cellobiose dehydrogenase from *Phanerochaete chrysosporium* and *Myricoccum thermophilum* (PDB entries 1naa and 4qi4, respectively), where it probably stabilizes the transition state during ligand oxidation (Hallberg *et al.*, 2003 ▸; Tan *et al.*, 2015 ▸). In the *Ct*FDO–ligand complexes, Asn562 and Tyr476 participate in ligand binding, and we suggest the same role for these residues in the case of the *Ct*FDO substrate, *i.e.* ligand-binding and/or transition-state stabilization.

### 4.6. Substrates of *Ct*FDO   

**Figure 7:**
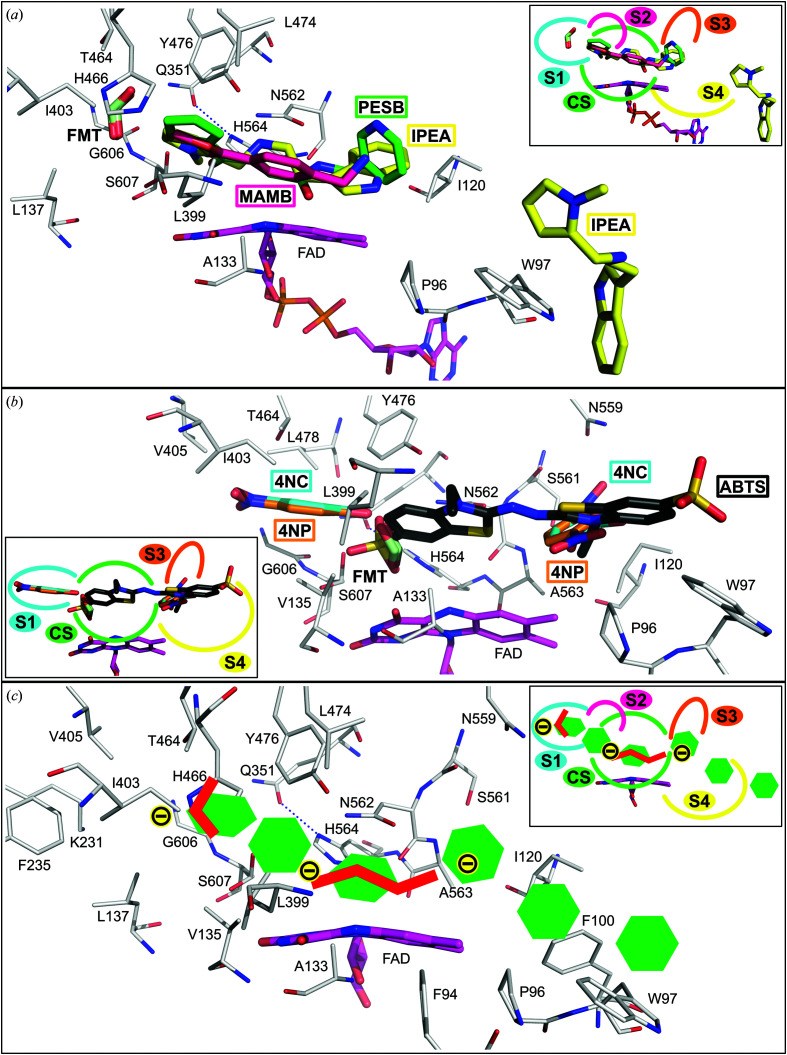
Three-dimensional superposition of the active sites of the *Ct*FDO complexes. FAD and formic acid (FMT) molecules and selected residues at distances of up to 3.8 Å from the ligands are shown with C atoms coloured magenta, pale green and light grey, respectively. The insets show the binding of the moieties of the ligands with regard to individual subsites. (*a*) Alignment of *Ct*FDO:MAMB (PDB entry 6ze3), *Ct*FDO:PESB (PDB entry 6ze4) and *Ct*FDO:IPEA (PDB entry 6ze5). The ligands MAMB, PESB and IPEA are shown with C atoms coloured hot pink, green and yellow, respectively. (*b*) Superposition of *Ct*FDO:4NC (PDB entry 6ze6), *Ct*FDO:4NP (PDB entry 6ze7) and *Ct*FDO:ABTS (PDB entry 7aa2). The ligands 4NC, 4NP and ABTS are shown with C atoms coloured cyan, orange and black, respectively. (*c*) Schematic pattern of ligand composition. Of all of the compounds used for *Ct*FDO–ligand complex preparation, only those containing an aromatic group (except for formic acid) were found binding in the internal pocket. Hexagons, red lines and black circles with a minus sign on a yellow background represent the binding of aromatic rings, aliphatic moieties and negatively charged moieties in the active-site pocket, respectively. The pattern indicates a polyaromatic composition of the putative substrate. Molecular graphics were created with *PyMOL* (Schrödinger).

The volume of the active-site pocket in *Ct*FDO was established to be approximately 2400 Å^3^ using *HOLLOW* (Ho & Gruswitz, 2008 ▸) and the 3*V* server (Voss & Gerstein, 2010 ▸). In terms of the density of the tested compounds for *Ct*FDO activity (alcohols, carbohydrates and lignin components), the volume corresponds roughly to a molecule with a molecular weight of between 1200 and 2500 Da, assuming that the entire pocket is filled. The crystallographic screening, together with several structurally identified binding subsites inside the active-site pocket that bind predominantly aromatic compounds, indicates that the *Ct*FDO substrate is likely to be a complex polyaromatic compound (Fig. 7 ▸, Supplementary Fig. S20). This is consistent with the abovementioned expectation of a substrate of high molecular weight based on the large volume of the active-site pocket. The substrate could possibly be of lignin-like character, although some lignin components (both small and bulky) failed to show *Ct*FDO activity in our experiments.

The spectrophotometric observation of the re-oxidation of *Ct*FDO after its reduction by sodium dithionite shows that *Ct*FDO is likely to utilize O_2_ as an acceptor of electrons and protons during the oxidative half-reaction. Our activity tests with typical substrates of GMC oxidoreductases revealed that *Ct*FDO had no activity against these compounds. Interestingly, a lack of enzymatic activity against a similar group of potential substrates was previously also described for another protein from the GMC oxidoreductase superfamily: allergen 12 from *Malassezia sympodialis* (Zargari *et al.*, 2007 ▸). The additional high-throughput screening of *Ct*FDO activity yielded no convincing substrate either. The ROS-Glo H_2_O_2_ Assay (Promega) that was used is based on several steps of the production of luciferin and its reaction with luciferase. Intermediates in the process contain the benzothiazole moiety. Considering the ability of *Ct*FDO to bind aromatics, including the benzothiazoline moieties of ABTS, it cannot be excluded that *Ct*FDO inhibits luciferin production and that its reaction with luciferase by binding the intermediates and, *vice versa*, luciferin products may inhibit the reaction of *Ct*FDO with the tested compounds. It is also possible that the N- and C-terminal residues that are missing in the recombinantly produced *Ct*FDO are necessary for the enzyme to be catalytically active. Also, we cannot exclude the possibility that *Ct*FDO is naturally enzymatically inactive, as shown previously, for example, for human dipeptidyl peptidase 10 (Bezerra *et al.*, 2015 ▸) and some members of the aldehyde dehydrogenase superfamily (Jackson *et al.*, 2015 ▸).

### 4.7. Glycosylation of *Ct*FDO   

Comparison of the *Ct*FDO structure with those of *An*GOX, *Pa*GOX and *Mt*AAO showed identical glycosylation sites at Asn114, Asn89, Asn93 and Asn107. The oligosaccharide units linked to Asn89 and Asn93 assist in the stabilization of the homodimers of *An*GOX and *Pa*GOX, *i.e.* the catalytically active forms of *An*GOX and *Pa*GOX. Monomeric forms of *An*GOX and *Pa*GOX do not possess catalytic activity (Ye & Combes, 1989 ▸; Witt *et al.*, 1998 ▸). Both glycosylated and deglycosylated forms of *Ct*FDO were found to be monomers in solution (Supplementary Figs. S3 and S7). Given the missing terminal residues of the enzyme, it cannot be excluded that the complete mature *Ct*FDO forms dimers. On the other hand, *Ct*FDO is more structurally similar to *Mt*AAO than to *An*GOX and *Pa*GOX in terms of its secondary-structure elements and the large active-site pocket with a wide-open entrance. *Mt*AAO is catalytically active as a monomer (Kadowaki *et al.*, 2020 ▸), although it also contains the abovementioned glycosylation site.

The oligosaccharide moieties of heterologously expressed fully glycosylated *Ct*FDO form almost one quarter (20 kDa) of the molecular weight of the whole *Ct*FDO molecule. It cannot be excluded that the extensive glycosylation of recombinant *Ct*FDO may lead to a decrease in or a loss of enzymatic activity, as reported previously for glucose oxidase (Romanos *et al.*, 1992 ▸).

If any of these effects cause any changes to *Ct*FDO activity, the actual catalytic site of *Ct*FDO does not seem to be affected: oxidation and reduction of the cofactor was observed as well as the role of molecular oxygen as a probable second substrate. As for the questionable identity of the first substrate, it is possible that the actual substrate was not in the test group of compounds or that the experiment design or reaction conditions were not optimal, but there is also a probability that this particular recombinant form of *Ct*FDO is not capable of the binding and/or the oxidation of the first substrate.

## 5. Conclusion   

Crystal structure analysis of *Ct*FDO showed that this novel enzyme with a currently unknown substrate preserves the typical fold of the GMC oxidoreductase superfamily, with the addition of several extra helices and with an atypically large and wide-open active-site tunnel. The active site contains a novel His–Ser pair (His564 and Ser607). His564 putatively acts as a general base and Ser607, together with Asn562 and Tyr476 on the FAD *re* face, participates in substrate binding. The active-site tunnel is extended beyond the pyrimidine moiety of FAD by a small cavity with positive electrostatic potential. The results of crystallographic fragment screening revealed the binding of small inorganic and aromatic moieties in several subsites of the tunnel and suggested a complex polyaromatic nature of the enzyme substrate corresponding to larger lignin components.

## 6. Related literature   

The following references are cited in the supporting information for this article: Hajizadeh *et al.* (2018 ▸).

## Supplementary Material

PDB reference: *Ct*FDO, 6ze2

PDB reference: complex with (4-methoxycarbonylphenyl)methylazanium, 6ze3

PDB reference: complex with 4-oxo-*N*-[(1*S*)-1-(pyridin-3-yl)ethyl]-4-(thiophen-2-yl)butanamide, 6ze4

PDB reference: complex with 2-(1*H*-indol-3-yl)-*N*-[(1-methyl-1*H*-pyrrol-2-yl)methyl]ethanamine, 6ze5

PDB reference: complex with 4-nitrocatechol, 6ze6

PDB reference: complex with 4-nitrophenol, 6ze7

PDB reference: complex with ABTS, 7aa2

Supporting Information 1. Supplementary Figures and Tables. DOI: 10.1107/S2059798321003533/jv5004sup1.pdf

Supporting Information 2. Information about the high-throughput activity screening, including Supplementary Fig. 21 and Tables S4 and S5. DOI: 10.1107/S2059798321003533/jv5004sup2.pdf

X-ray diffraction data for the structure of unliganded CtFDO (PDB entry 6ze2).: https://doi.org/10.15785/SBGRID/804

X-ray diffraction data for the structure of CtFDO in complex with (4-methoxycarbonylphenyl)methylazanium (PDB entry 6ze3).: https://doi.org/10.15785/SBGRID/805

X-ray diffraction data for the structure of CtFDO in complex with 4-oxo-N-[(1S)-1-(pyridin-3-yl)ethyl]-4-(thiophen-2-yl)butanamide (PDB entry 6ze4).: https://doi.org/10.15785/SBGRID/806

X-ray diffraction data for the structure of CtFDO in complex with 2-(1H-indol-3-yl)-N-[(1-methyl-1H-pyrrol-2-yl)methyl]ethanamine (PDB entry 6ze5).: https://doi.org/10.15785/SBGRID/807

X-ray diffraction data for the structure of CtFDO in complex with 4-nitrocatechol (PDB entry 6ze6).: https://doi.org/10.15785/SBGRID/808

X-ray diffraction data for the structure of CtFDO in complex with ABTS (PDB entry 7aa2).: https://doi.org/10.15785/SBGRID/810

X-ray diffraction data to the structure of CtFDO in complex with 4-nitrophenol (PDB entry 6ze7).: https://doi.org/10.15785/SBGRID/809

## Figures and Tables

**Table 1:** Data-collection and processing statistics Values in parentheses are for the outer shell. The resolution cutoff was chosen so that CC_1/2_ was greater than 0.5 and the mean *I*/σ(*I*) in the outer shell was around 1.5.

	*Ct*FDO:free	*Ct*FDO:MAMB	*Ct*FDO:PESB	*Ct*FDO:IPEA	*Ct*FDO:4NC	*Ct*FDO:4NP	*Ct*FDO:ABTS
Method of complex preparation	—	Soaking	Soaking	Soaking	Soaking	Co-crystallization	Co-crystallization
PDB code	6ze2	6ze3	6ze4	6ze5	6ze6	6ze7	7aa2
Diffraction source	P13, PETRA III	14.2, BESSY II	14.1, BESSY II	P13, PETRA III	14.2, BESSY II	14.1, BESSY II	P13, PETRA III
Wavelength (Å)	1.0332	0.9184	0.9184	0.9763	0.9184	0.9184	0.9763
Temperature (K)	100	100	100	100	100	100	100
Detector	PILATUS 6M	PILATUS 2M	PILATUS 6M	PILATUS 6M	PILATUS 2M	PILATUS 6M	PILATUS 6M
Crystal-to-detector distance (mm)	141.74	222.69	266.58	343.72	159.95	293.45	243.41
Rotation range per image (°)	0.1	0.1	0.1	0.05	0.1	0.1	0.05
Total No. of images	1699	1996	1498	3579	2000	1400	2000
Exposure time per image (s)	0.04	0.1	0.2	0.04	0.2	0.1	0.04
Space group	*P*2_1_	*P*2_1_2_1_2	*P*2_1_2_1_2_1_	*P*2_1_	*P*2_1_2_1_2_1_	*P*2_1_2_1_2_1_	*P*2_1_2_1_2_1_
*a*, *b*, *c* (Å)	46.7, 116.8, 109.0	109.7, 115.6, 46.6	93.6, 109.9, 116.1	47.0, 117.0, 109.9	93.6, 109.6, 116.1	92.6, 109.7, 115.6	93.6, 109.8, 116.0
α, β, γ (°)	90.0, 90.8, 90.0	90.0, 90.0, 90.0	90.0, 90.0, 90.0	90.0, 90.7, 90.0	90.0, 90.0, 90.0	90.0, 90.0, 90.0	90.0, 90.0, 90.0
Mosaicity (°)	0.06	0.38	0.19	0.18	0.11	0.08	0.09
Resolution range (Å)	46.69–1.31 (1.33–1.31)	43.26–2.22 (2.29–2.22)	49.38–1.60 (1.63–1.60)	43.40–1.82 (1.85–1.82)	29.06–1.26 (1.28–1.26)	47.23–1.50 (1.53–1.50)	49.34–1.40 (1.42–1.40)
Total No. of reflections	856212 (38307)	218724 (20580)	811144 (43706)	358504 (18223)	2316159 (81149)	976283 (48771)	825189 (43026)
No. of unique reflections	268173 (13444)	30083 (2690)	147085 (7605)	105562 (5247)	314691 (14281)	186872 (9171)	223121 (11468)
Completeness (%)	95.4 (96.8)	99.9 (100.0)	93.5 (98.5)	99.4 (99.7)	98.3 (91.0)	99.5 (99.2)	95.5 (99.8)
Multiplicity	3.2 (2.8)	7.3 (7.7)	5.5 (5.7)	3.4 (3.5)	7.4 (5.7)	5.2 (5.3)	3.7 (3.8)
〈*I*/σ(*I*)〉	11.2 (2.0)	8.3 (1.8)	8.7 (1.6)	9.6 (1.7)	16.5 (1.7)	10.6 (1.7)	9.2 (1.4)
Solvent content (%)	48.1	47.5	48.0	48.6	47.9	47.1	47.9
Matthews coefficient (Å^3^ Da^−1^)	2.37	2.34	2.36	2.39	2.36	2.32	2.36
*R*_merge_	0.045 (0.489)	0.167 (1.147)	0.088 (1.009)	0.079 (0.752)	0.060 (0.978)	0.086 (0.908)	0.058 (0.925)
*R*_meas_	0.053 (0.599)	0.182 (1.236)	0.097 (1.108)	0.094 (0.888)	0.065 (1.076)	0.096 (1.008)	0.067 (1.079)
*R*_p.i.m._	0.029 (0.340)	0.066 (0.441)	0.039 (0.441)	0.051 (0.469)	0.024 (0.439)	0.042 (0.429)	0.033 (0.542)
CC_1/2_	0.999 (0.795)	0.996 (0.646)	0.998 (0.659)	0.997 (0.673)	1.000 (0.660)	0.997 (0.668)	0.999 (0.589)
Overall *B* factor from Wilson plot (Å^2^)	12.7	33.5	16.8	22.0	10.0	11.1	16.2

**Table 2:** Structure solution and refinement AU, asymmetric unit; GlcNAc, *N*-acetyl-D-glucosamine.

	*Ct*FDO:free	*Ct*FDO:MAMB	*Ct*FDO:PESB	*Ct*FDO:IPEA	*Ct*FDO:4NC	*Ct*FDO:4NP	*Ct*FDO:ABTS
PDB entry	6ze2	6ze3	6ze4	6ze5	6ze6	6ze7	7aa2
PDB ligand ID	—	8G2	479	4AQ	4NC	NPO	EBS
Molecular-replacement program	*MoRDa*	*MOLREP*	*MOLREP*	—	*Phaser*	*Phaser*	Phaser
Refinement program	*REFMAC*5	*REFMAC*5	*REFMAC*5	*phenix.refine*	*REFMAC*5	*REFMAC*5	*REFMAC*5
*R*_work_	0.106	0.178	0.169	0.167	0.113	0.155	0.128
*R*_free_	0.137	0.255	0.203	0.202	0.147	0.182	0.172
Reflections used for *R*_free_ (%)	2.0	5.0	5.0	4.8	2.0	5.1	5.0
Average *B* factor (Å^2^)	17.4	41.3	19.6	26.5	14.3	16.0	19.1
R.m.s. deviations from ideal							
Bond lengths (Å)	0.013	0.010	0.010	0.011	0.014	0.012	0.013
Angles (°)	1.825	1.738	1.658	1.127	1.839	1.776	1.751
Ramachandran plot†							
Favoured (%)	96.1	94.0	95.7	95.6	96.2	96.1	96.2
Outliers (%)	0.0	0.3	0.2	0.2	0.0	0.2	0.0
No. of outliers	0	2	2	2	0	3	0
No. of protein subunits per AU	2	1	2	2	2	2	2
Localized ligands in AU (occupancy)	—	1 MAMB (1.0)	2 PESB (0.9)	3 IPEA (1.0)	4 4NC (0.7-0.8)	3 4NP (0.8)	2 ABTS (0.6–0.9)
No. of localized water molecules	1664	310	1611	1272	1884	1489	1653
No. of other localized moieties							
FAD	2	1	2	2	2	2	2
GlcNAc	11	6	12	10	13	10	13
Mannose	—	—	1	—	—	—	—
Formic acid	2	1	4	4	4	8	2
Mg^2+^	1	1	1	2	3	5	3
Na^+^	—	1	2	—	1	1	—
Cl^−^	—	—	—	—	1	1	1
Tetraethylene glycol/triethylene glycol	—	—	—	—	—	2/2	—
Acetate ion	—	—	—	—	—	2	—

† As calculated by *MolProbity*.
