# Crystallographic fragment screening-based study of a novel FAD-dependent oxidoreductase from *Chaetomium thermophilum*. Corrigendum

**DOI:** 10.1107/S2059798321006100

**Published:** 2021-06-18

**Authors:** Leona Švecová, Lars Henrik Østergaard, Tereza Skálová, Kirk Matthew Schnorr, Tomáš Koval’, Petr Kolenko, Jan Stránský, David Sedlák, Jarmila Dušková, Mária Trundová, Jindřich Hašek, Jan Dohnálek

**Affiliations:** a Institute of Biotechnology of the Czech Academy of Sciences, v.v.i., Průmyslová 595, 252 50 Vestec, Czech Republic; b Faculty of Nuclear Sciences and Physical Engineering, Czech Technical University in Prague, Břehová 7, 115 19 Prague 1, Czech Republic; c Novozymes A/S, Biologiens Vej 2, 2800 Kgs. Lyngby, Denmark; d CZ-OPENSCREEN: National Infrastructure for Chemical Biology, Institute of Molecular Genetics of the Czech Academy of Sciences, v.v.i., Vídeňská 1083, 142 20 Prague, Czech Republic

**Keywords:** FAD-dependent oxidoreductases, GMC oxidoreductases, *Chaetomium thermophilum*, crystallographic fragment screening, corrigendum, synchrotron facilities

## Abstract

The synchrotron facilities used in collecting the data for the article by Švecová *et al.* [(2021), *Acta Cryst.* D**77**, 755–775] are acknowledged.

## Abstract 2

The article by Švecová *et al.* [(2021), *Acta Cryst.* D**77**, 755–775] is updated.

In the article by Švecová *et al.* (2021 ▸) proper acknow­ledgement was not made to the two synchrotron sources used to collect the data. Here we update the acknowledgements section and add two references.

In Section 2.6 of the article the third sentence should include citations as follows: Diffraction data were collected either on beamline P13 of the PETRA III synchrotron-radiation source, DESY, Hamburg, Germany (Cianci *et al.*, 2017 ▸) or on beamlines 14.1 and 14.2 of the BESSY II synchrotron-radiation source, Helmholz-Zentrum, Berlin, Germany (Mueller *et al.*, 2015 ▸).

In Section 2.8 of the article the third sentence should include a citation as follows: For data collection, an HR2000+ES UV–Vis spectrophotometer (OceanOptics) at the MX-SpectroLab at BESSY II, Helmholz Zentrum, Berlin, Germany (Mueller *et al.*, 2015) was used.

An updated ackowledgements section is given below.
