# Supplementary material for: Crystallographic fragment screening-based study of a novel FAD-dependent oxidoreductase from Chaetomium thermophilum
Source: Acta Crystallogr D Struct Biol. 2021 May 14;77(Pt 6):755–75. doi: 10.1107/S2059798321003533 (PMC8171062; doi:10.1107/S2059798321003533)
Supplement: Supplementary file 1 [file d-77-00755-sup1.pdf]

STRUCTURAL
BIOLOGY

Volume 77 (2021)

Supporting information for article:

Crystallographic fragment screening-based study of novel FAD-dependent oxidoreductase from *Chaetomium thermophilum*

Leona Švecová, Lars Henrik Øestergaard, Tereza Skálová, Kirk Matthew Schnorr, Tomáš Koval', Petr Kolenko, Jan Stránský, David Sedlák, Jarmila Dušková, Mária Trundová, Jindřich Hašek and Jan Dohnálek

Supporting information 1

Crystallographic fragment screening-based study of novel FAD-dependent oxidoreductase from *Chaetomium thermophilum*

Authors

Leona Švecová[ab]*, Lars H. Østergaard[c], Tereza Skálová[a], Kirk Schnorr[c], Tomáš Koval'[a], Petr Kolenko[ab], Jan Stránský[a], David Sedlák[d], Jarmila Dušková[a], Mária Trundová[a], Jindřich Hašek[a] and Jan Dohnálek[a]*

[a]Institute of Biotechnology of the Czech Academy of Sciences v.v.i., Průmyslová 595, Vestec, 25250, Czech Republic

[b]Faculty of Nuclear Sciences and Physical Engineering, Czech Technical University in Prague, Břehová 7, Prague 1, 11519, Czech Republic

[c]Novozymes A/S, Biologiens Vej 2, Kgs. Lyngby, 2800, Denmark

[d]CZ-OPENSCREEN: National Infrastructure for Chemical Biology, Institute of Molecular Genetics of the Czech Academy of Sciences v.v.i., Vídeňská 1083, Prague, 14220, Czech Republic

Correspondence email: leona.svecova@ibt.cas.cz; jan.dohnalek@ibt.cas.cz

IMPORTANT: this document contains embedded data - to preserve data integrity, please ensure where possible that the IUCr Word tools (available from http://journals.iucr.org/services/docxtemplate/) are installed when editing this document.

```
ATGAGGACATCAAGTTTCCAGCTTCTGGGAGCGGTTTTGCCCCTCCTGTCTCTGTCGGCAGCGAGTCCAA 70
TCGATATCGACTCTGCAAAAGTCCCAAGACATGCCACAATTGTTCACCCAAGGAACGTTCACTCCAATTA 140
TACATTCATCATTGCTGGCGGCGGCATATCCGGGCTCACCCTCGCCGACCGTCTAACCGAAGATCCTCGA 210
GGTACAAACGACTCCCCTAGAGAACTATGTATGTAGGAGACTGGCTGGTTACTAATGTCGAATTTTCTCT 280
TTTTTGCTATAGTAACCGTCCTTGTGATTGAGGCCGGTCCTTTGGATCGGGGTGAAGATGGCATCCTCGT 350
CCCGGGAGCCTTCTCTCCTTGGCTCTACTTCTGGCCCGGTCTTGTTAGCACACCTCAAGCAGGGCTTAAT 420
AACAGGACCGTTGATGTTATCACAGCGCAGGTTGTCGGTGGCGGAAGCACTATCAACGCTATGGTGTACT 490
TGAGGTACGCTGAGCAGGCGACAGAATACGCCTTCCTGCCATCAGCCAAGCTGCATGCTGACGGCGTCTA 560
TTCAGGGGTGACAAGGATGATTATGATTCATGGGGTGCCCTCGGAAATCCGGGCTGGAGCTGGAATTCCA 630
TGCTTCCCTATTTCATCAAGGTAAAGCTATGGCTAGAAAGCTCCAGACAGCACCAACTGGCCCCGTGCTA 700
ACACAGCGCAGAGTGAAACTTTCACGCCCCCTAGCCCTGAACTTGCTGCGGCTGGCAACATCACTTGGGA 770
TGGTTCAATTCGGGGCCGTTCTGGTCCCGTGAACTACTCATATCCCAACTATTTCTTCCCGGGGAGCGGT 840
GAGTGACAGTCTGCGGAGAGATTGTTGGCATATGAACTGAATGCTGACAATGTATCCTAAAGAAAACTGG 910
TGGAACGCCGCAAATGAAGTCGGCCTTCCTCCTGTTAAGGACCCAATGGCGGGGTCGAAGCAAGGTGTTT 980
TCTGGATCCCATCTGCCATTGATGCCAGGACTATGACAAGAAGCCATGCTCGGCGCAATCATTACGACCG 1050
CGTCAGCTCCCGGCCAAACTACCACATCCTGCCATCTCACCTGGTGTCTAAGATTTTATTCAGGGGCAAG 1120
CAAGCCATTGGCGTGAGCTACATCCCGACTTCTGGCGGCAATACTACGACCAATGTCTACGCAAGCAAGG 1190
AGATCACCCTGGCTGCTGGTGGCCTGGGAACCCCGAAGATCCTGCAACTCTCGGGTATTGGCCCCCGGAA 1260
GCTTCTCAACGAGCTCGGCATCCCTGTCATTTCCGACCTCCCCGGCGTCGGGCAGAACCTGCAGGACCAG 1330
CCCACGCTGACAATCCCGTATACTTGTGCGTTTTTCCTTTTCATTCGTCCTTTCGAGTATGTTCTGAGTC 1400
AAGAGCGGTTGACTAACAATTTGCTTCTTCCCATGTGAAGTCACAAACAACGTGTTTCCCAACACTGATA 1470
GCCTCACCACGAACGCTACTTACAACGCCGAGCAGCGGGCATTGTATGACAGCTCGAAGCAGGGTGCTTA 1540
CACCATCGTTAACTCCTTGAGCACGAACATTGGTGTGATGTCCCTGCAGCGGGCGGCACCCAAAAGCTAC 1610
CGGCAGATCCAACGCCGAGCAGCGGGCATTGTATGACAGCTCGAAGCAGGGTGCTTACACCATCGTTAAC 1680
TCCTTGAGCACGAACATTGGTGTGATGTCCCTGCAGCGGGCGGCACCCAAAAGCTACCGGCAGATCATCG 1750
CCGCTGCCAGGGCGCGCAGTGCCTCGCTTTCTCTGCCTCCTGGCACCGACCCAGCCGTCATCCGTGGCTA 1820
CCAGGCACAGCGCAATGCCATCCTGAAGCAGTTTGAGAACCCCAACGTCGGTGTTGGCACAGTGCACTGG 1890
GGCACGGGTAGCTCGGCGTTGGTGTACCATCTGAAGCCCCTGAGCCGCGGCACGGTCAACATTCGCTCAA 1960
CCAATCCTCTGGACGCCCCAGAAATCGACTACCGGACGGGCACCGATCCCATCGACGCGCAGGTTTACAC 2030
GTCTTTGTTCCGCAAGAACCGCGAAATTTTCAACGCGCCGTCGATGCGCGTGCTGGGTCCCAGTGAGGCA 2100
GCACCCTTCGGCGCCAACCTGACCACCGATGAGGAGATTTATGCTGTCATGCGCGAGCTGATCAACCCGT 2170
CGAACGCGCACCAGTGCTGCACGGCTGCCATGATGCCCAAGGATATGGGTGGTGTTGTATCAAGTGAACA 2240
GAAGGTGTATGGTGTTCAGGGCCTCCGTGTCGCGGATATCAGCTTCTGGCCCTTCCAGCTCTCAGGGTCA 2310
CCCATGGCCACAGCGTACGCGGGCGCTGAGAGGGTACGTTCAAAGTCTCGAGTCTTCTGACAAACGTGCT 2380
AACACGCATTTGTAGCTCGCCGATGTCATCAAGAAGGAGCACCGCCTGGCTGGCGCACCTAAGAGTCTAG 2450
TAGGGCGCTGCATGCAATGAGAATCGCACGTCAAAACGAAAAATAATACATATAATAACTAATTTAAACA 2520
TCAGCCATCGAGGAATAACCTCAAATCGGCCAAACAATCGGAGTAA ••• 2566
```

Figure S1 DNA sequence encoding the *Ct*FDO. The exons are highlighted in rose colour and these are spliced together into mRNA. For a recombinant production, the complete sequence.

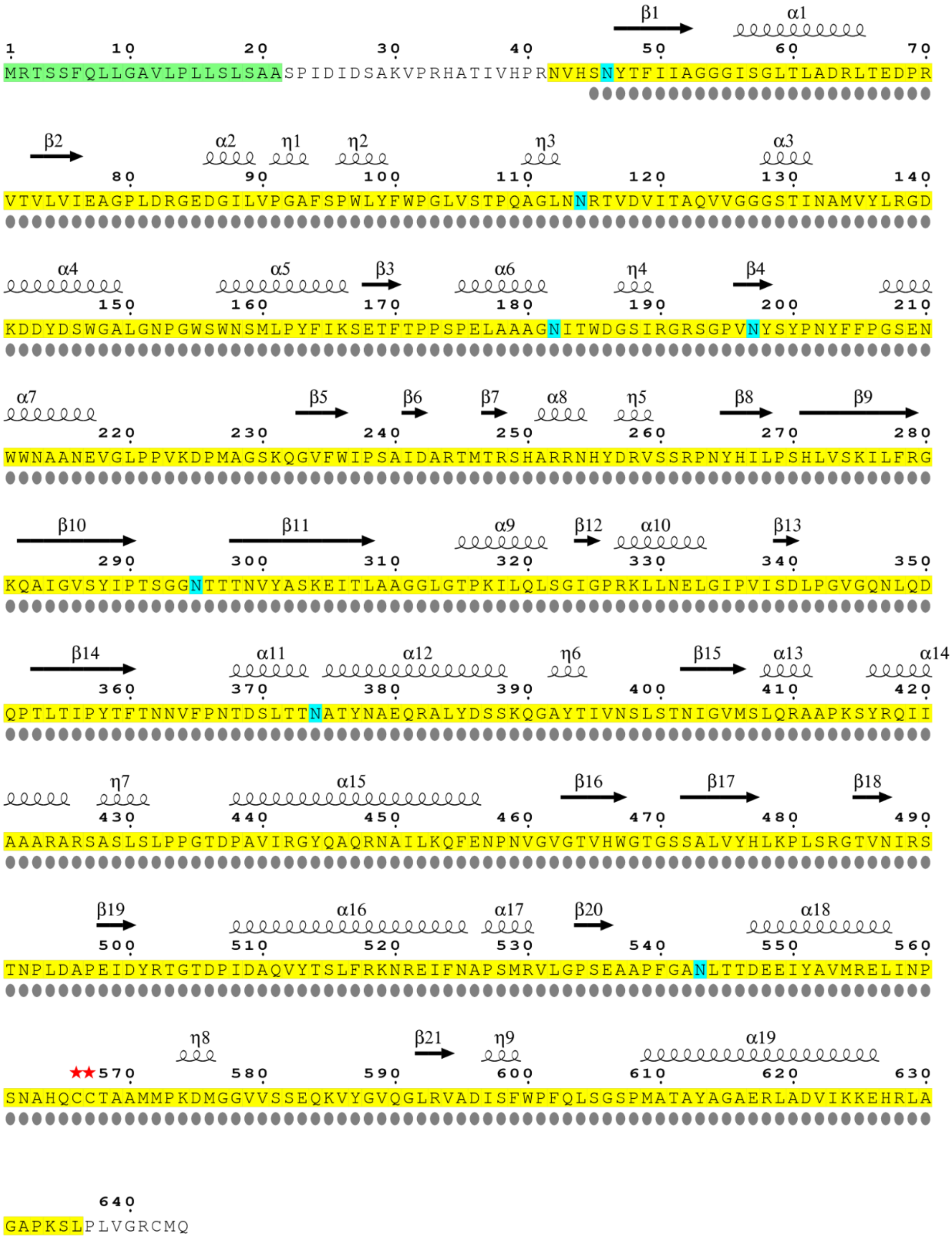

Figure S2 Complete expected mature sequence of *Ct*FDO with highlighted sequence parts confirmed with liquid chromatography-mass spectrometry (LC-MS/MS, yellow background) and with *Ct*FDO crystal structures (grey dots). The sequence part with green background correspond to the signal peptide. Residues highlighted by cyan background mark the N-glycosylation sites in *Ct*FDO. The red stars mark the only disulphide bridge. For the LC-MS/MS, the deglycosylated sample of *Ct*FDO was digested by pepsin. Fragments were analysed by LC-MS/MS using an electrospray ion source of a 15T solariX XR FT-ICR mass spectrometer (Bruker Daltonics). Mass spectrometer was operated in positive, data dependent mode. Data were processed using the DataAnalysis 4.2 software and exported to mgf format. The fragments were identified by ProteinScape (Bruker Daltonics) with the Mascot search engine.

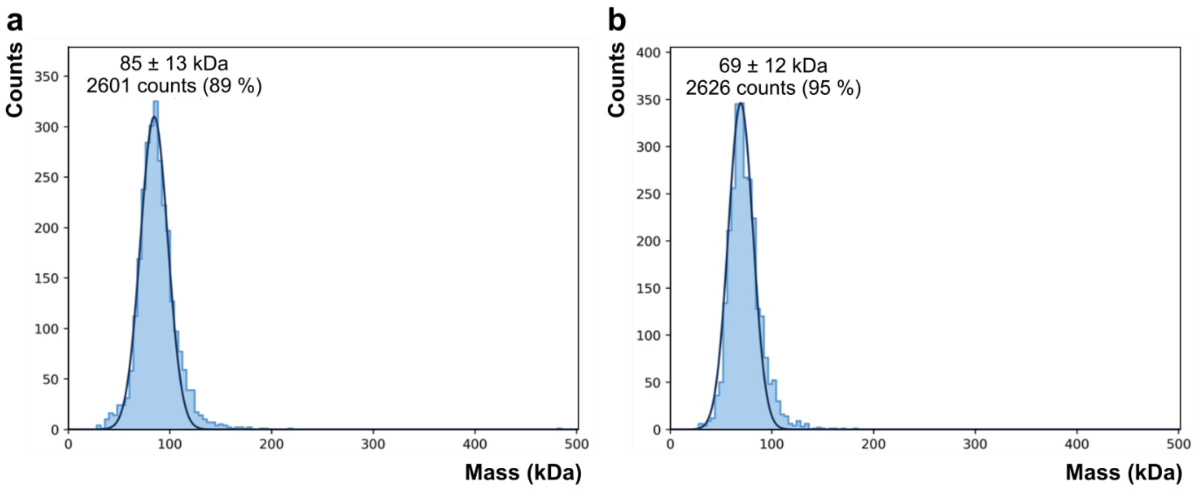

Figure S3 Mass distribution analysis done using mass photometry with (a) glycosylated and (b) deglycosylated form of *Ct*FDO (*Ct*FDO_{degl}). Graphs show a single peak for *Ct*FDO and *Ct*FDO_{degl} corresponding to 85 and 69 kDa containing more than 89 and 95 % of the population, respectively. No other oligomeric form was found. Data were collected on a Refeyn OneMP instrument using the DiscoverMP (v 2.2.1) software. Samples were diluted in 25 mM Tris-HCl with 75 mM NaCl, pH 7.5 to the concentration of 20 nM and 24 nM for *Ct*FDO and *Ct*FDO_{degl}, respectively. Data was analysed using DiscoverMP (version v. 2.3.dev12) using default settings.

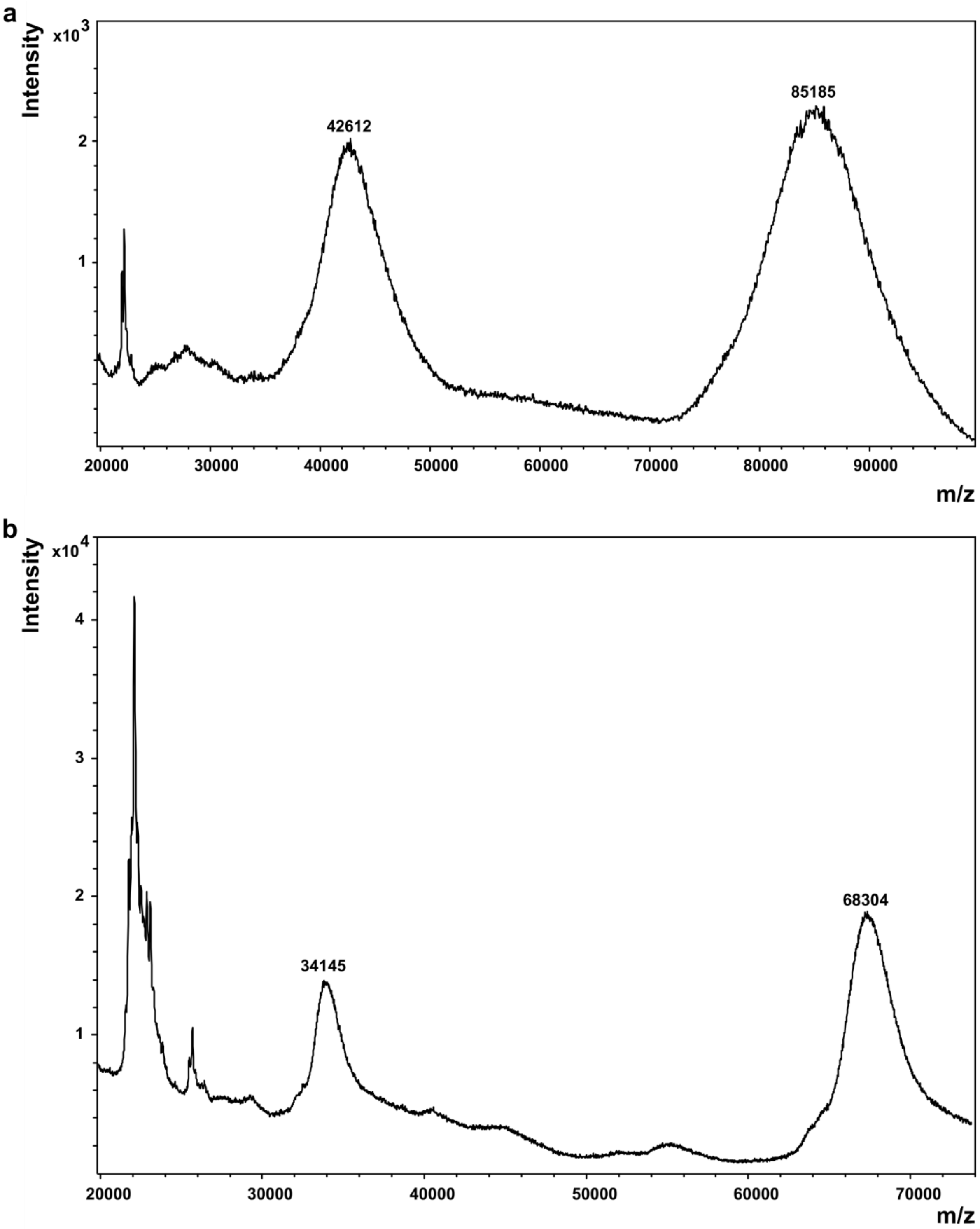

Figure S4 MALDI-TOF spectra of *Ct*FDO in (a) fully glycosylated and (b) deglycosylated form. One µl of sample at a concentration of 10 pmol·µl^{-1} was applied on stainless-steel MALDI target and let dry. The sample was overlaid with 1 µl of sinapinic acid (Sigma-Aldrich) and let dry at room temperature. Intact protein was analysed by a MALDI-TOF mass spectrometer Autoflex Speed (Bruker Daltonics) operated in linear positive mode. Data were processed by the FlexAnalysis 3.3 software (Bruker Daltonics).

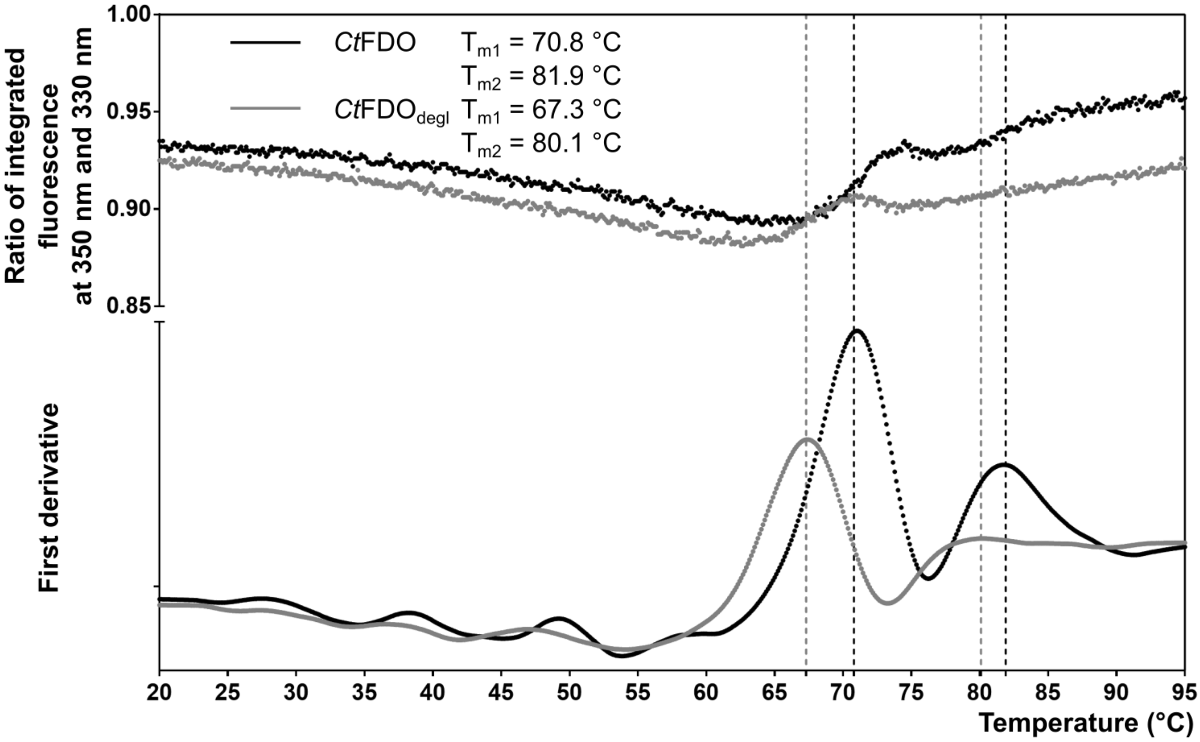

Figure S5 Thermal stability of *Ct*FDO fully glycosylated (black curve) and deglycosylated (*Ct*FDO$_{degl}$) by endoglycosidase F1 (grey curve) measured by nano differential scanning fluorimetry using a Prometheus NT.48 instrument (NanoTemper). The samples of concentration approximately 0.7 mg·ml^{-1} were diluted in 25 mM Tris-HCl, pH 7.5 with 100 mM NaCl. The measurements were done in temperature range 20-95°C with slope 2.5 °C per min. Difference in the height of peaks is given by a slightly different concentration of the samples. The data were evaluated with the PR.ThermControl v2.1.1 software and plotted using GraphPad Prism 7.02 (GraphPad Software).

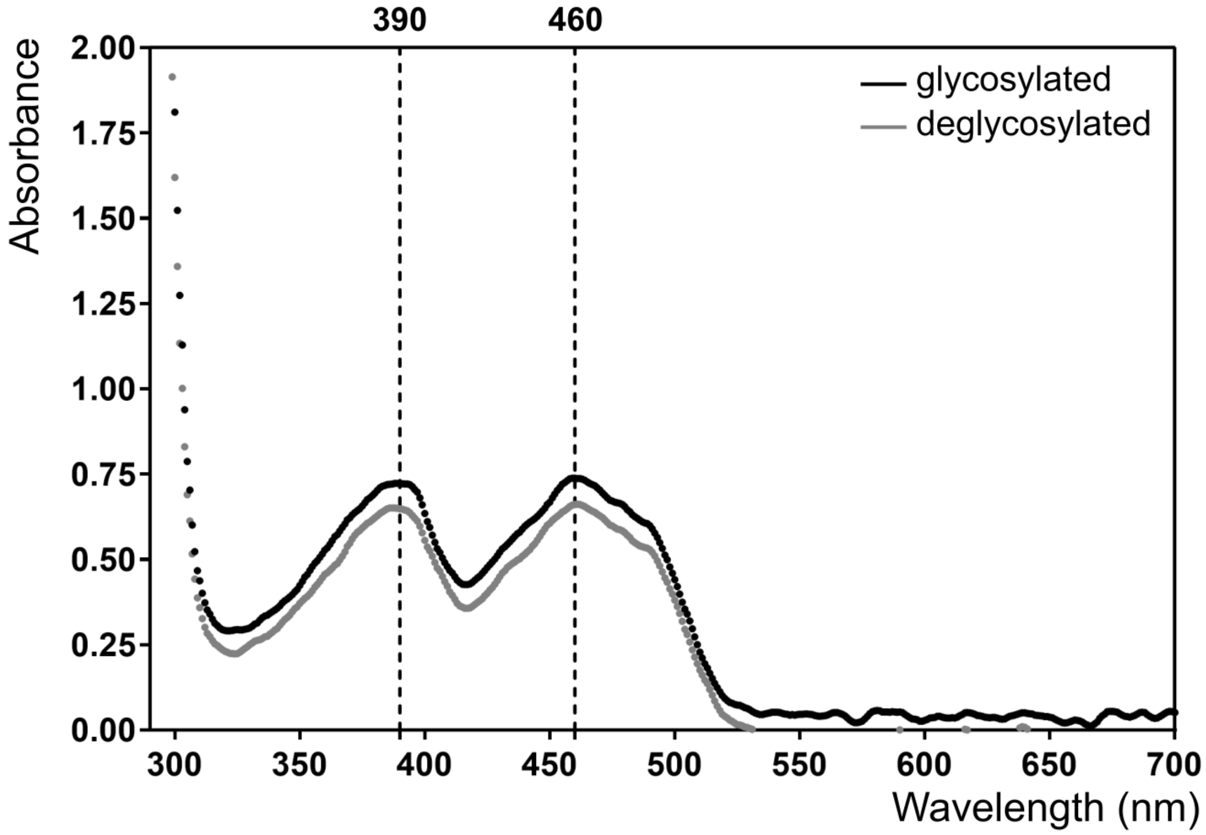

Figure S6 The UV-VIS absorption spectra for fully glycosylated (in black) and deglycosylated (in grey) *Ct*FDO in solution showing two maxima at 390 nm and 460 nm. Both profiles show an oxidatized state of FAD in solution (25 mM Tris-HCl with 100 mM NaCl, pH 7.5). The UV-VIS absorption spectra were measured using a DeNovix DS-11 microvolume spectrophotometer with protein concentration of approximately 7.2 mg ml^{-1}. The different height of peaks is given by varied concentration of the samples. The spectra were plotted with GraphPad Prism version 7.02 for Windows (GraphPad Software, La Jolla California USA, www.graphpad.com).

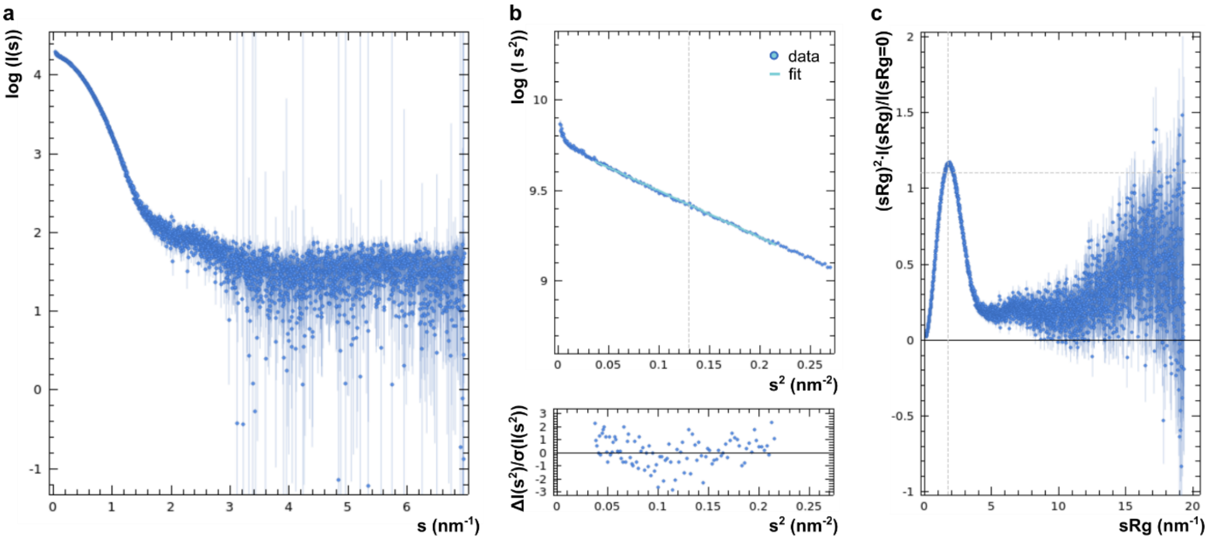

Figure S7 The small-angle X-ray scattering (SAXS) plots obtained for deglycosylated form of *Ct*FDO (*Ct*FDO$_{degl}$). (a) The scattering plot (b) Guinier plot, and (c) dimensionless Kratky plot with *s* normalized by *R*g (2.77 ± 0.08) and *I*(sRg) normalized to sRg = 0. The estimated molecular weight for *Ct*FDO$_{degl}$ ranges between 56 and 68 kDa depending on the calculation method (Hajizadeh *et al.*, 2018) (59 kDa based on Q_P –protein volume from the Porod invariant, 68 kDa based on M_0W, 56 kDa based on V_C – empirical Volume of Correlation). The SAXS experiment was performed in batch mode at the EMBL P12 beamline at the Petra III synchrotron radiation source (DESY, Hamburg) using a detector Pilatus 6M (Dectris, Baden-Daettwil, Switzerland). The measurement was done at 20 °C with sample-to-detector distance 3.0 m, wavelength 1.24 Å, and exposure time per image 0.045 s. The data were measured for *Ct*FDO$_{degl}$ (4.2 mg·ml^{-1}), dissolved in 25 mM Tris-HCl, pH 7.5 with 25 mM NaCl. The data were processed using the ATSAS 3.0.0 package (Manalastas-Cantos *et al.*, 2021). The data had fair quality according to the shape of the scattering curve, Guinier analysis and Kratky plot. Molecular envelope was not calculated.

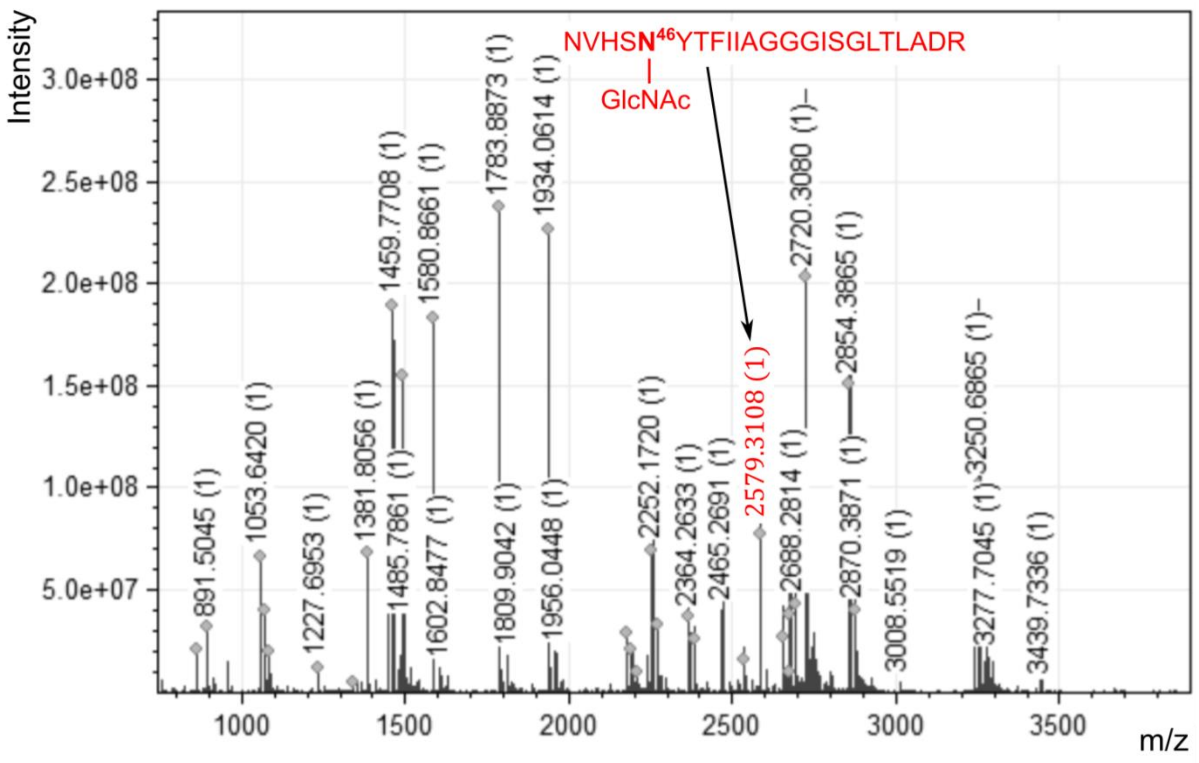

Figure S8 Confirmation of *Ct*FDO identity and presence of glycosylation site at Asn46 with MALDI-TOF peptide mass fingerprinting (PMF). The observed ion at m/z 2579.3108 corresponds to peptide N^{42}VHSNYTFIISGGGISGLTLADR^{64} modified by one *N*-acetyl-D-glucosamine unit linked to Asn46. PMF was performed using a sample of *Ct*FDO$_{degl}$ (amount of 4 µg) loaded onto SDS gel. SDS gel bands were cut-out, chopped into small pieces, and dehydrated by acetonitrile. Dithiothreitol at a concentration of 50 mM was added to the gel pieces and let incubate at 60 °C for 30 min. Then iodoacetamide at a concentration of 100 mM was added to the gel pieces. After 30 min in dark and at room temperature, the gel pieces were washed by water and dehydrated by acetonitrile. Trypsin solution was added to the gel pieces and let incubate at 37 °C overnight. One microliter of tryptic peptide mixture was applied on the stain-less steel MALDI target, covered with α-Cyano-4-hydroxycinnamic acid as a matrix and analysed by a 15T solariX XR FT-ICR mass spectrometer (Bruker Daltonics) operating in positive mode. Data were processed by the DataAnalysis 4.2 software (Bruker Daltonics) and the mMass software.

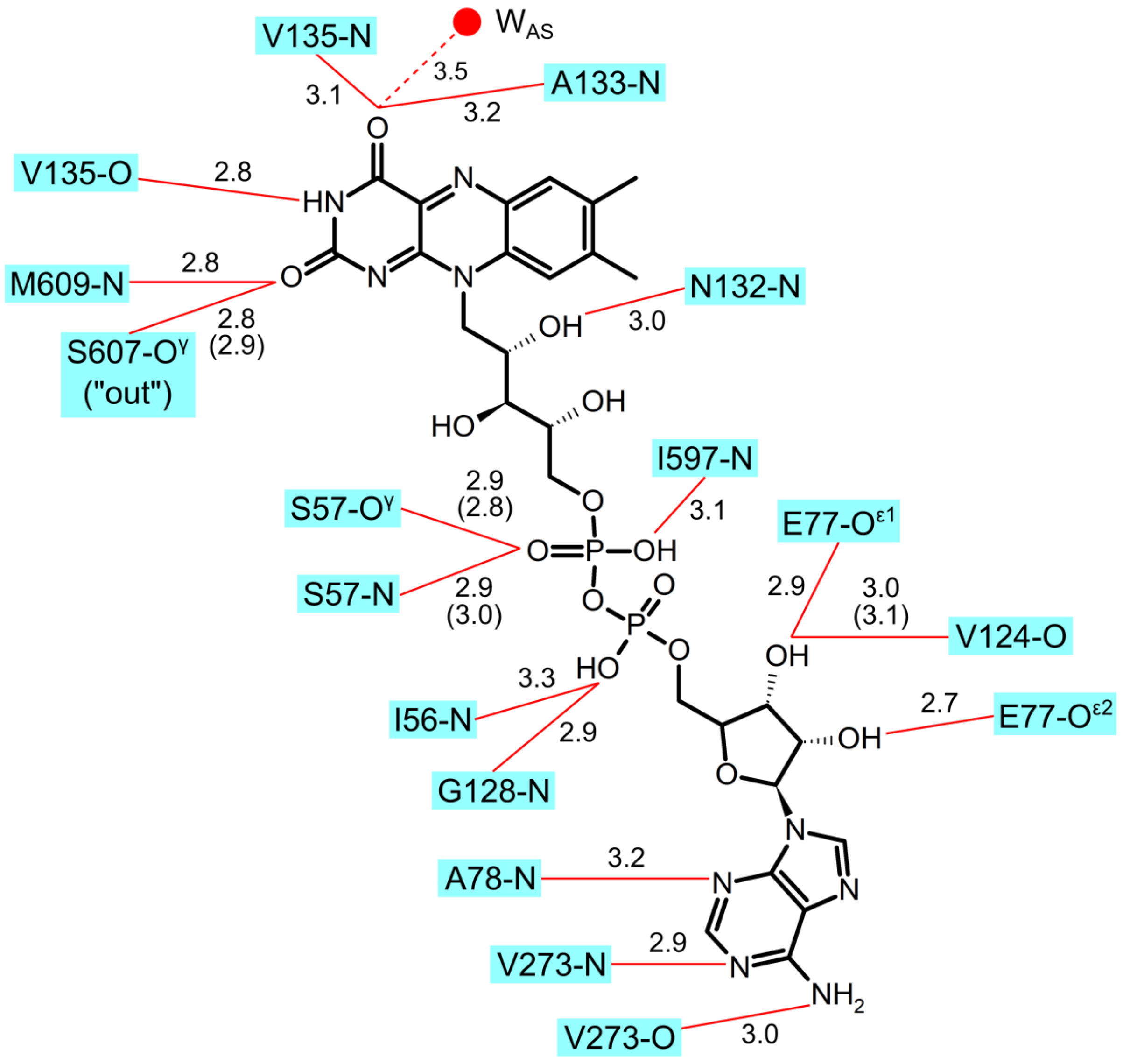

Figure S9 Schematic diagram of the FAD interactions with *Ct*FDO. Hydrogen bonds are indicated as red lines with donor-acceptor distances in Å (different values for chain B are given in parentheses). Besides the residues of *Ct*FDO, the interaction with the active-site water (W_{AS}) is indicated (red sphere).

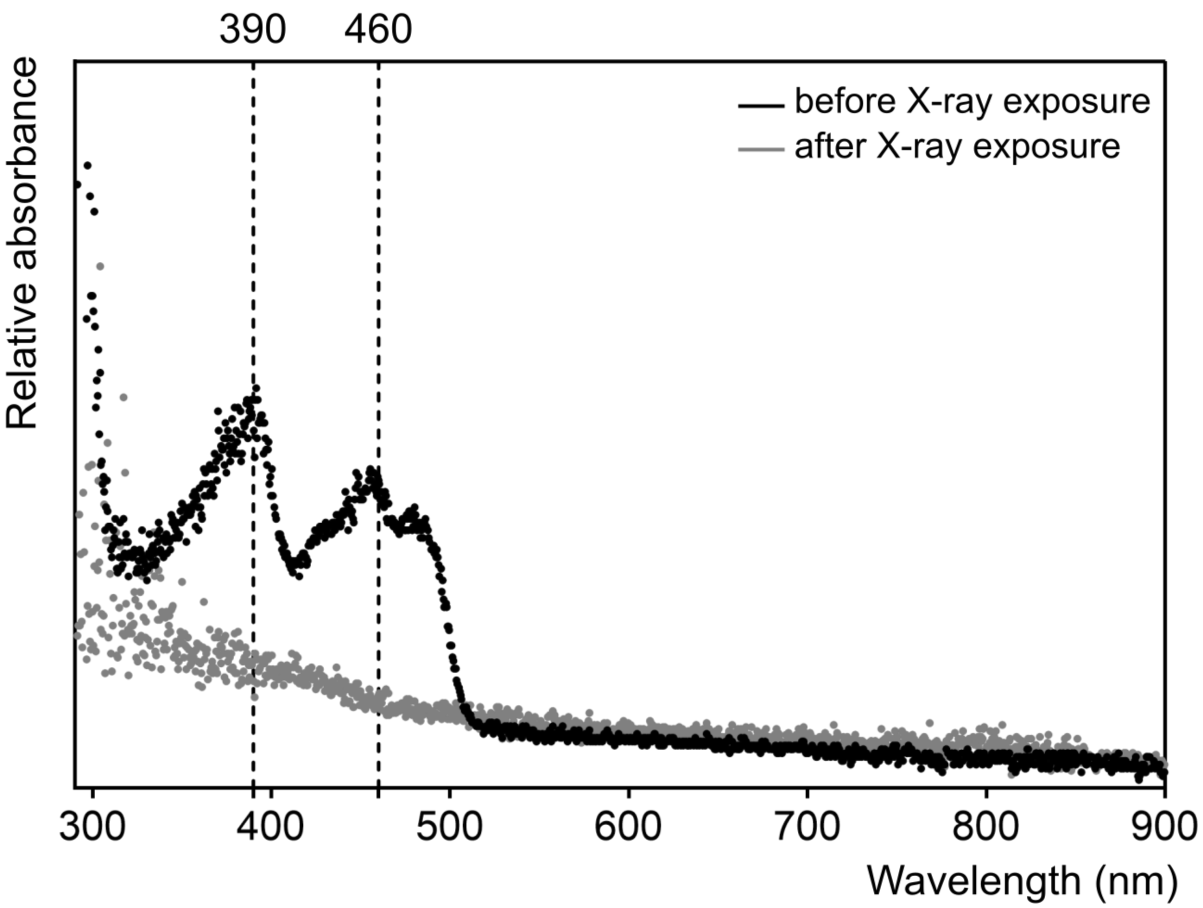

Figure S10 The UV-VIS absorption spectra measured for *Ct*FDO$_{degl}$ crystal before (in black) and after (in grey) exposure to X-ray beam show the oxidized state and reduced state of FAD, respectively. The data prove reduction of FAD during exposure to X-rays. The absorption spectra were recorded with the OceanView spectroscopy software and normalized at 290 and 900 nm in GraphPad Prism version 7.02 for Windows (GraphPad Software, La Jolla California USA, www.graphpad.com).

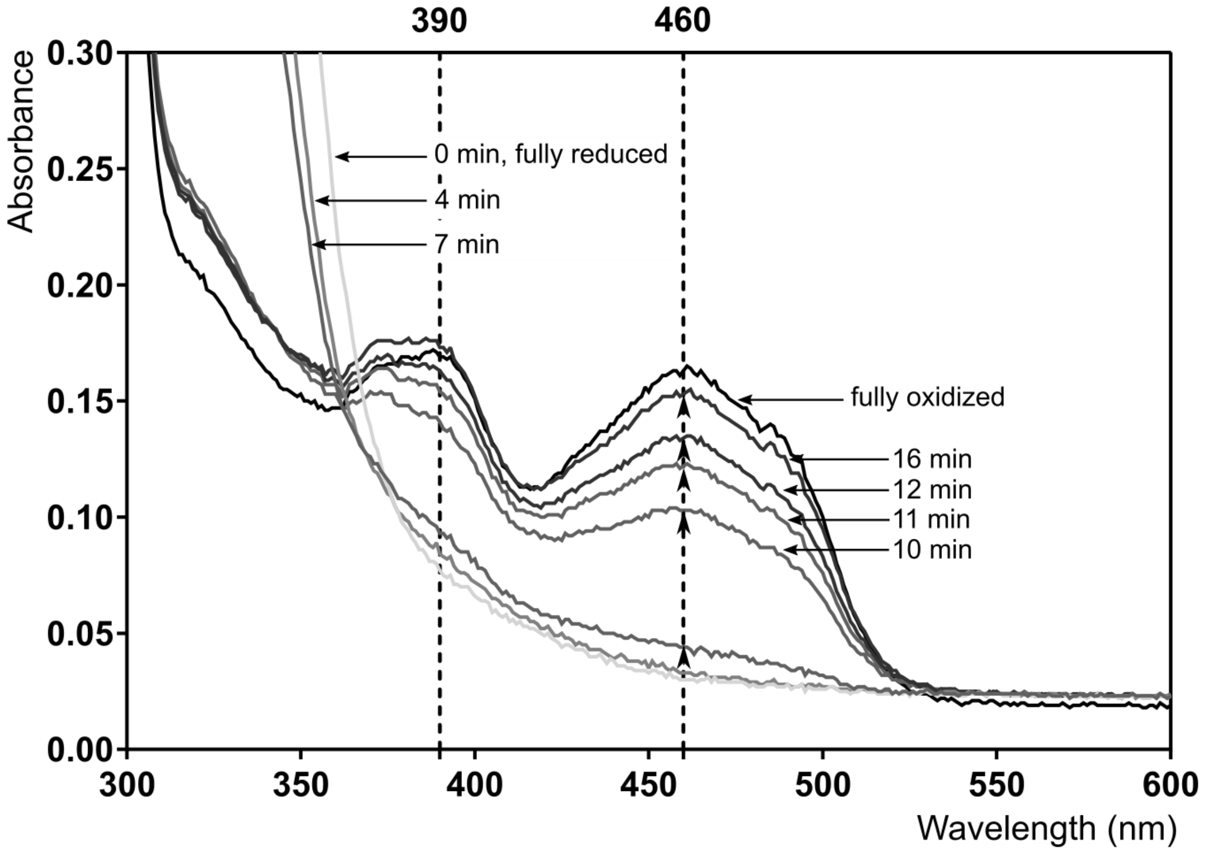

Figure S11 UV-VIS absorption spectra of *Ct*FDO reduction by dithionite (DTN) and re-oxidation by atmospheric molecular oxygen in solution. Reduced *Ct*FDO lacks the flavin absorption peaks around 390 and 460 nm, typical for the oxidized state of FAD. The increased absorbance in the range of 300-360 nm corresponds to absorbance of DTN and its consumption in time. The solution was stirred by a pipette after 3 and 9 min. The re-oxidation effect was monitored with a UV/VIS spectrophotometer (Libra S22, Biochrom Ltd.) and the Resolution Spectrophotometer PC Software (Biochrom Ltd.). The spectra were buffer-subtracted. The spectra were plotted with GraphPad Prism version 7.02 for Windows (GraphPad Software, La Jolla California USA, www.graphpad.com).

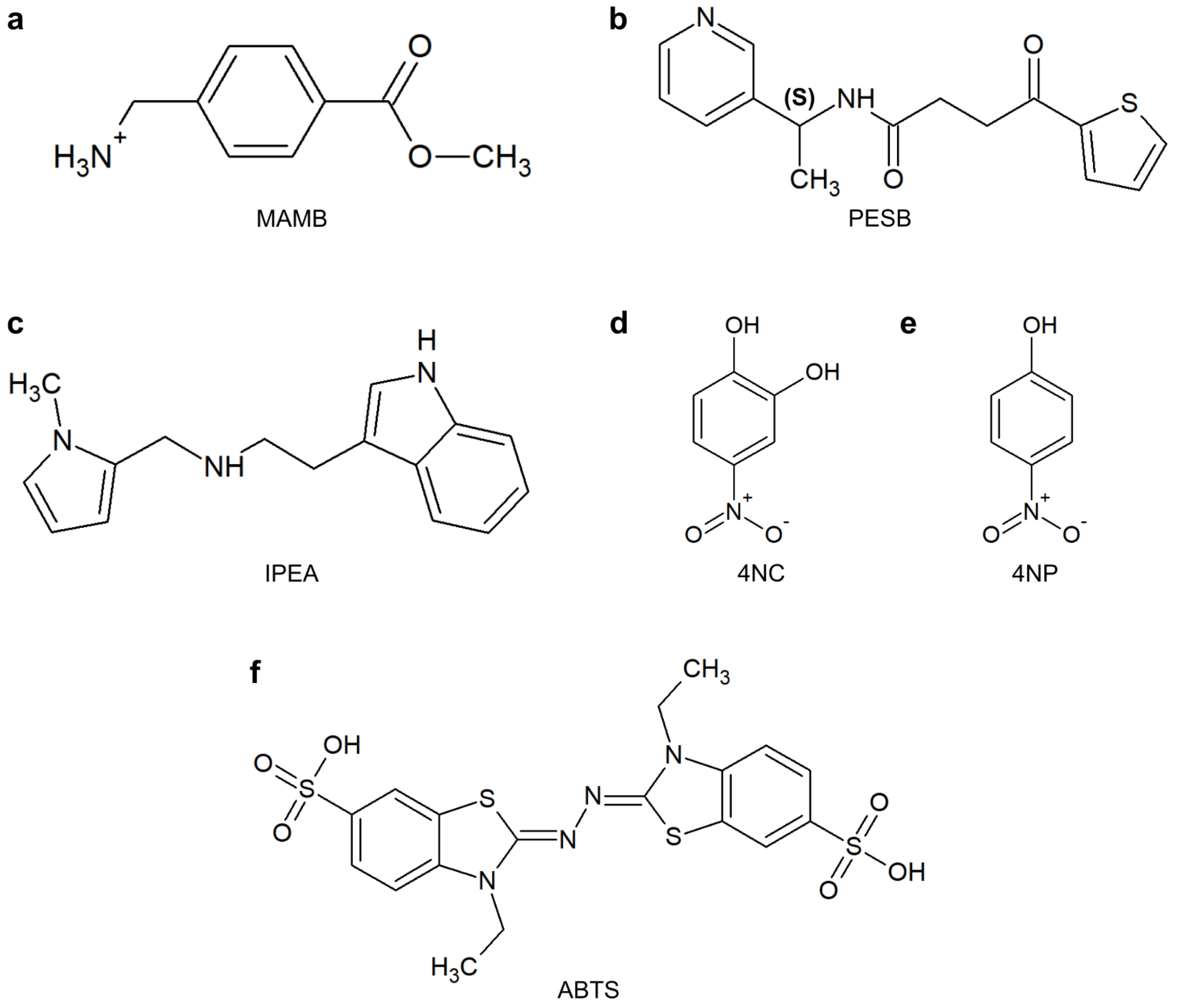

Figure S12 Structural formulae of ligands binding in the *Ct*FDO active-site pocket. (a) methyl 4-(aminomethyl)benzoate (MAMB), (b) 4-oxo-*N*-[1-(3pyridinyl)ethyl]-2-thiophenebutanamide (PESB), (c) 2-(1*H*-indol-3-yl)-*N*[(1-methyl-1*H*-pyrrol-2-yl)-methyl]-ethanamine (IPEA), (d) 4-nitrocatechol (4NC), (e) 4-nitrophenol (4NP), and (f) 2,2'-azino-bis(3-ethylbenzothiazoline-6-sulfonic acid) (ABTS).

Table S1 Summary of ligands and fragments utilized for preparation of crystals of *Ct*FDO complexes using soaking or co-crystallization method.

For co-crystallization, the ligand concentration is given for reservoir solution and for soaking, it is given for crystallization drop. The concentration of fragments from Frag Xtal Screen is given as amount in crystallization drop, since most of them were not completely soluble in reservoir solution. ★marks solved structure of *Ct*FDO:ligand complex.

Ligand	Concentration	Method	Result
Carbohydrates			
Cellobiose	6-22 mM	co-crystallization	deteriorated diffraction
D-(+)-galactose	10-15 mM	co-crystallization	ligand binding not detected
Glucose	10 mM	co-crystallization	ligand binding not detected
Lactose	15 mM	co-crystallization	ligand binding not detected
Rhamnose	6-10 mM	co-crystallization	deteriorated diffraction
Raffinose	5 mM	co-crystallization	ligand binding not detected
Sucrose	10 mM	co-crystallization	ligand binding not detected
Maltose	15 mM	co-crystallization	ligand binding not detected
Na-L-ascorbic acid	20 mM	co-crystallization	ligand binding not detected
D-sorbitol	20 mM	co-crystallization	deteriorated diffraction
Lignin constituents			
Guaiacylglycerol-beta-guaiacyl ether	10 mM	soaking (1 h)	ligand binding not detected
Caffeic acid	18 mM	co-crystallization	ligand binding not detected
Cinnamyl alcohol	22 mM in 0.6 % ethanol	co-crystallization	ligand binding not detected
p-coumaric acid	20 mM	co-crystallization	ligand binding not detected
4-methoxybenzyl alcohol	25 mM in 10 % ethanol	co-crystallization	ligand binding not detected
4-hydroxy-3-methoxybenzyl alcohol	15 mM	co-crystallization	ligand binding not detected
Aliphatic alcohols			
Crotyl alcohol	20 mM	co-crystallization	ligand binding not detected
Choline	20 mM	co-crystallization	ligand binding not detected
Aromatic alcohols			
o-phenylenediamine	10 mM	soaking (4–6 min)	ligand binding not detected
Aminophenol	10 mM	soaking (5 min)	ligand binding not detected
4-nitrophenol	20 mM	co-crystallization	★*Ct*FDO:4NP (PDB entry: 6ze7)
2-phenylethanol	20 mM	co-crystallization	ligand binding not detected
3-phenyl-1-propanol	5 mM	soaking (3 s)	crystal damage, ligand binding not detected
Aromatic acids			
Gallic acid	3.5 mM	soaking (3 s)	crystal damage, ligand binding not detected

Ellagic acid	5 mM	co-crystallization	deteriorated diffraction
Dyes			
2,6-dichloroindophenol	0.12 mM	co-crystallization	ligand binding not detected
Reducing agents			
Potassium ferrocyanide	20 mM	co-cryst., soaking (2–4 min)	ligand binding not detected
Dithionite	10 mM	soaking (10 s)	crystal damage, deteriorated diffraction
AMP containing ligand			
Adenosine monophosphate	20 mM	co-crystallization	deteriorated diffraction
Nicotinamide adenine dinucleotide	60 mM	co-crystallization	ligand binding not detected
Others			
5-hydroxymethyl furfural	10 mM	soaking (2–4 min)	ligand binding not detected
Cystamine	20 mM	co-crystallization	★*Ct*FDO:free (PDB entry: 6ze2)
2,2’-azino-bis(3-ethylbenzothiazoline-6-sulfonic acid	8.5 mM	co-crystallization	★*Ct*FDO:ABTS (PDB entry 7aa2)
Fluvastatin	1.3 mM	soaking (1–2 min)	ligand binding not detected
Eltrombopag	0.5 mM	soaking (1 min)	ligand binding not detected
AZD-8055	0.5 mM	soaking (1 min)	ligand binding not detected
Triamcinolone	1.3 mM	soaking (2 min)	ligand binding not detected
Frag Xtal Screen (*Jena Bioscience*) *order* (*place in the 96-well plate*)	Amount in drop		
FRS-3 (A3)	50 µmol	soaking (70 h)	ligand binding not detected
FRS-6 (A6)	50 µmol	soaking (3.5–24 h)	ligand binding not detected
FRS-7 (A7)	50 µmol	soaking (3–7 h)	ligand binding not detected
FRS-9 (A9)	50 µmol	soaking (20 h)	ligand binding not detected
FRS-10 (A10)	50 µmol	soaking (17 h)	ligand binding not detected
FRS-12 (A12)	50 µmol	soaking (70 h)	deteriorated diffraction
FRS-16 (B4)	50 µmol	soaking (2.5–70 h)	ligand binding not detected
FRS-18 (B6)	50 µmol	soaking (70 h)	ligand binding not detected
FRS-24 (B12)	50 µmol	soaking (3–17 h)	★*Ct*FDO:PESB (PDB entry: 6ze4)
FRS-28 (C4)	50 µmol	soaking (3– 21 h)	ligand binding not detected
FRS-29 (C5)	50 µmol	soaking (70 h)	ligand binding not detected
FRS-32 (C8)	50 µmol	soaking (2.5 h)	★*Ct*FDO:8G2 (PDB entry: 6ze3)
FRS-35 (C11)	50 µmol	soaking (70 h)	★*Ct*FDO:IPEA (PDB entry: 6ze5)
FRS-36 (C12)	50 µmol	soaking (3–22 h)	deteriorated diffraction

FRS-38 (D2)	50 µmol	soaking (2.5–24 h)	deteriorated diffraction
FRS-39 (D3)	50 µmol	soaking (21 h)	ligand binding not detected
FRS-41 (D5)	50 µmol	soaking (2.5–24 h)	deteriorated diffraction
FRS-42 (D6)	50 µmol	soaking (3 h)	ligand binding not detected
FRS-43 (D7)	50 µmol	soaking (2.5–24 h)	deteriorated diffraction
FRS-47 (D11)	50 µmol	soaking (21 h)	deteriorated diffraction
FRS-48 (D12)	50 µmol	soaking (17 h)	ligand binding not detected
FRS-49 (E1)	50 µmol	soaking (21 h)	ligand binding not detected
FRS-52 (E4)	50 µmol	soaking (21 h)	ligand binding not detected
FRS-57 (E9)	50 µmol	soaking (21 h)	ligand binding not detected
FRS-59 (E11)	50 µmol	soaking (21 h)	deteriorated diffraction
FRS-62 (F2)	50 µmol	soaking (21 h)	ligand binding not detected
FRS-65 (F5)	50 µmol	soaking (3.5 h)	ligand binding not detected
FRS-67 (F7)	50 µmol	soaking (3.5–24 h)	ligand binding not detected
FRS-69 (F9)	50 µmol	soaking (21 h)	ligand binding not detected
FRS-70 (F10)	50 µmol	soaking (3.5–24 h)	ligand binding not detected
FRS-72 (F12)	50 µmol	soaking (3.5–24 h)	ligand binding not detected
FRS-74 (G2)	50 µmol	soaking (16.5–17 h)	ligand binding not detected
FRS-77 (G5)	50 µmol	soaking (3.5–24 h)	ligand binding not detected
FRS-78 (G6)	50 µmol	soaking (20 h)	ligand binding not detected
FRS-80 (G8)	50 µmol	soaking (17 h)	ligand binding not detected
FRS-81 (G9)	50 µmol	soaking (21 h)	deteriorated diffraction
FRS-82 (G10)	50 µmol	soaking (17 h, 20 h)	★*Ct*FDO:4NC (PDB entry: 6ze6)
FRS-88 (H4)	50 µmol	soaking (21 h)	deteriorated diffraction
FRS-90 (H6)	50 µmol	soaking (17 h)	ligand binding not detected
FRS-91 (H7)	50 µmol	soaking (3.5–24 h)	ligand binding not detected
FRS-92 (H8)	50 µmol	soaking (17 h)	ligand binding not detected
FRS-94 (H10)	50 µmol	soaking (20 h)	ligand binding not detected

Table S2 Summary of substrates tested for *Ct*FDO activity.

[a] The reaction was run in Britton-Robinson buffer pH 3.5-9.8 with 0.25 µg of *Ct*FDO for at 45 °C for 20 min. [b] The reaction was run in 100 mM phosphate buffer with 30 mM NaCl pH 4.5-9 with 2 µg of *Ct*FDO at 37 °C for 20 min. [c] The reaction as in [a] was run with 0.5 µg of *Ct*FDO. [d] The reaction as in [b] was run at 45 °C. For compounds included in high-throughput activity screening see Supporting information 2.

Substrate	[Substrate] in solution	pH
Carbohydrates		
Glucose[b]	5 mM	4.5, 6.0, 8.0
L-Rhamnose[b]	5 mM	4.5, 6.0, 8.0
Cellobiose[b]	5 mM	4.5, 6.0, 8.0
Lactose[b]	5 mM	4.5, 6.0, 8.0
Aliphatic alcohols - saturated		
1,4-butanediol[a]	5 mM	3.5, 5.4, 7.1, 8.0
Aliphatic alcohols - unsaturated		
Crotyl alcohol[b]	100 mM	5.0, 6.0, 8.0
Aromatic alcohols		
Phenol[b]	5 mM	5.0, 6.0, 8.0
Catechol[a]	5 mM	3.5, 5.8, 6.7, 7.7, 8.9
Hydroquinone[b]	5 mM	5, 6, 8
4-nitrophenol[b]	3.8 mM	5.0, 6.0, 8.0
Benzyl alcohol[a]	5 mM	3.5, 5.4, 7.1, 8.0
4-methoxybenzyl alcohol[a]	5 mM	3.5, 5.4, 7.1, 8.0
4-methoxybenzyl alcohol[b]	50 mM	4.5, 5.0, 6.0, 7.0, 8.0, 9.0
4-hydroxy-3-methoxybenzyl alcohol[a]	5 mM	3.5, 5.4, 7.1, 8.0
Cinnamyl alcohol[b]	5 mM	5.0, 6.0, 8.0
p-coumaryl alcohol[b]	9.5 mM	4.5, 6, 8
Coniferyl alcohol[a]	5 mM	3.5, 5.4, 7.1, 8.0
2-phenyl ethanol[b]	4.4 mM	5.0, 6.0, 8.0
3-phenyl-1-propanol[a]	5 mM	3.5, 5.4, 7.1, 8.0
2,6-dimethoxyphenol[a]	5 mM	3.5, 5.8, 6.7, 7.7, 8.9
Aldehydes		
4-anisaldehyd[a]	4.3 mM	3.5, 5.4, 7.1, 8.0
Vitamins		
Pyridoxine[a]	5 mM	3.7, 5.4, 7.1, 7.4, 8.0
Others		
Choline[a]	5 mM	3.5, 5.4, 7.1, 8.0
5-(hydroxymethyl)furfural[a]	5 mM	3.5, 5.8, 6.7, 7.7, 8.9
Guiacyl-glycerol-guiacyl ether[a]	2.4 mM	3.5, 5.4, 7.1, 8.0
Tannic acid[b]	5 mM	5.0, 7.0
ABTS[c]	5 mM	4.5, 5.4, 6.1, 7.1, 8.0, 8.9
Tetracycline[d]	27 mM	4.5, 5.0, 6.0, 7.0, 8.0
Bilirubin[b]	200 uM	8.0
Dithiotreitol[b]	5 mM	5.0, 8.0

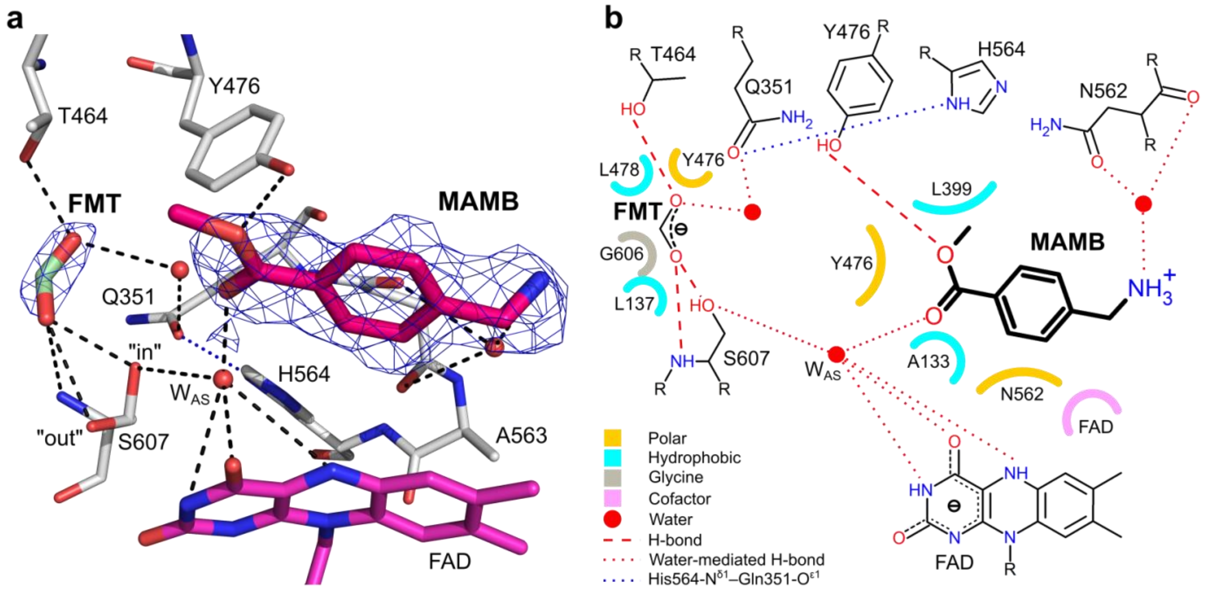

Figure S13 The active-site pocket of *Ct*FDO:MAMB (PDB entry: 6ZE3). (a) Simulated-annealing $2mF_O$-DF_C composite omit map shown for the formic acid (FMT) and MAMB molecules contoured at 1σ level (calculated in Phenix Terwilliger *et al.*, 2008). The amino acid residues, MAMB, flavin, and FMT are shown as sticks with carbon coloured in grey, hotpink, magenta, and palegreen, respectively. The cofactor is shown as $FADH^-$ form, but it cannot be excluded that it is $FADH_2$. The water molecules (red spheres) are shown with up to four most probable hydrogen bonds. The active-site water is labelled W_{AS}. The dashed lines mark hydrogen bonds and the blue dotted line marks the hydrogen bond stabilizing the position of the His564 imidazole ring. The graphics was created in Pymol Molecular Graphics System (Schrödinger). (b) 2D schematic interaction diagram of the active site of *Ct*FDO:MAMB. Hydrogen bonds are indicated as dashed or dotted lines. Residues with vdW contacts are marked with semicircles.

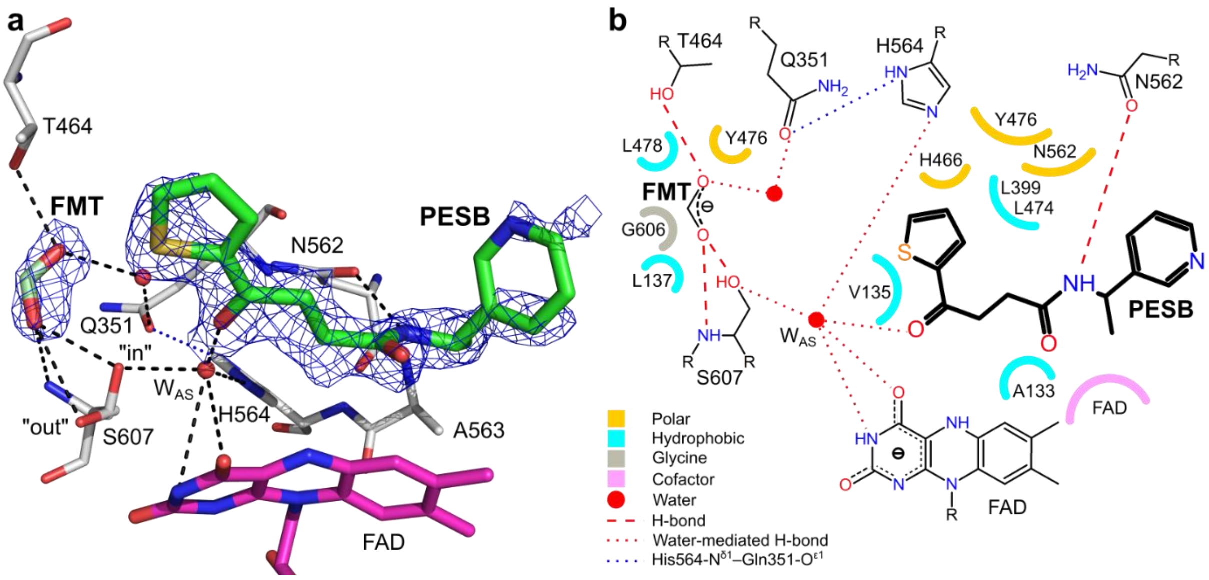

Figure S14 The active-site pocket of *Ct*FDO:PESB (PDB entry: 6ZE4, chain A). (a) Simulated-annealing 2mF_O-DF_C composite omit map shown for the PESB and formic acid (FMT) molecules contoured at 0.8σ level (calculated in Phenix, Terwilliger *et al.*, 2008). The amino acid residues, flavin, PESB, and FMT are shown as sticks with carbons coloured in grey, magenta, green, and palegreen, respectively. The water molecules are shown as red spheres, the active-site water molecule is labeled W_{AS} and is shown with all hydrophilic interactions. The dashed lines mark hydrogen bonds and the blue dotted line marks the hydrogen bond stabilizing the position of the His564 imidazole ring. The graphics was created with the Pymol Molecular Graphics System (Schrödinger). (b) 2D schematic interaction diagram of the active site of *Ct*FDO:PESB. The hydrogen bonds are indicated as dashed or dotted lines. Residues with vdW contacts are marked with semicircles.

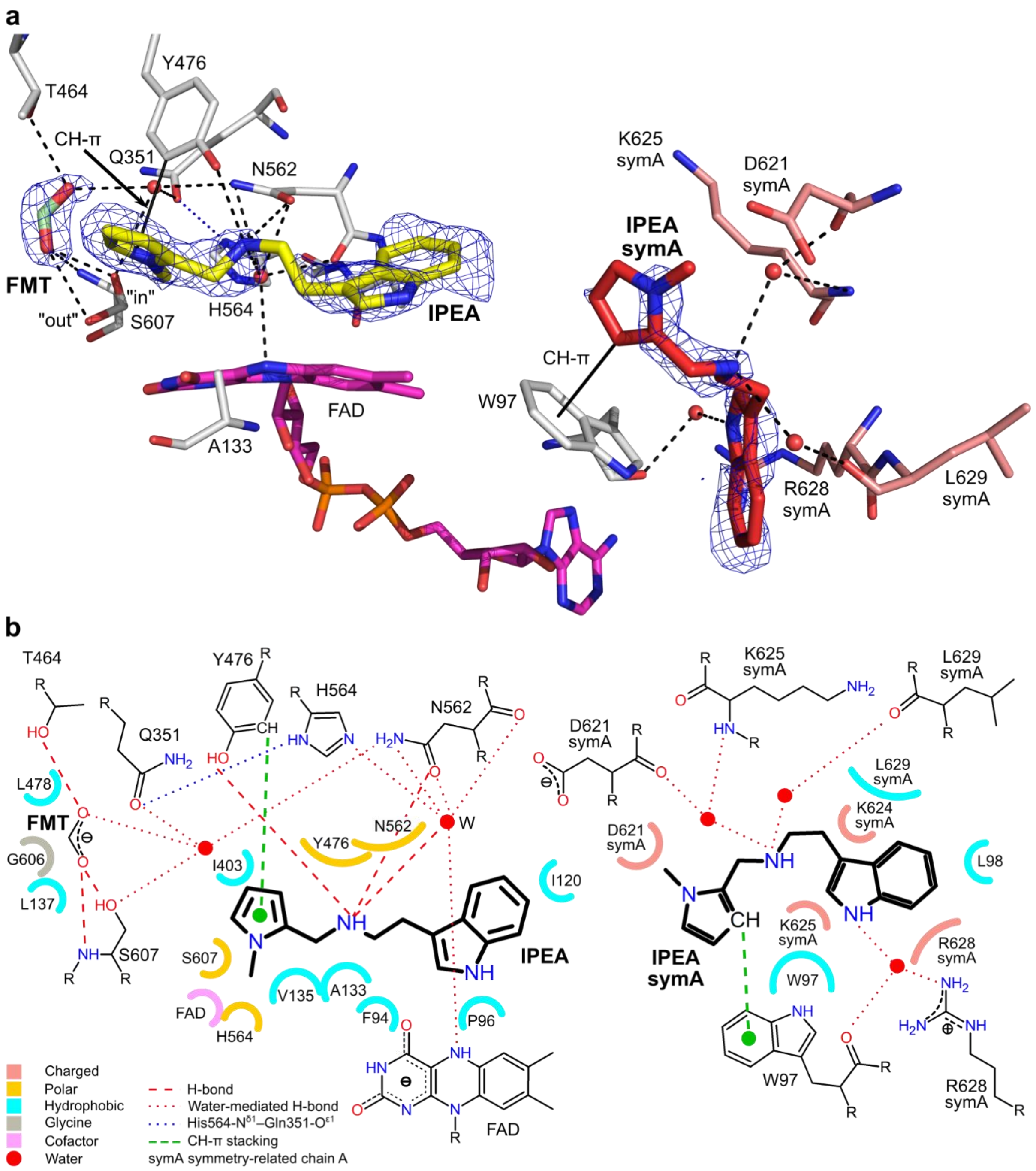

Figure S15 The active-site pocket of *Ct*FDO:IPEA (PDB entry: 6ZE5, merge of chain A and symmetry-related chain A). (a) Simulated-annealing $2mF_{\mathrm{O}}$-DF_{C} composite omit map shown for the formic acid (FMT) and IPEA molecules contoured at 1σ level (calculated in Phenix, Terwilliger *et al.*, 2008). The *Ct*FDO:IPEA (residues with carbon grey) binds FMT (carbon palegreen) and IPEA (carbon yellow) in the active-site pocket and second IPEA molecule (carbon red) on the interface of symmetry-related chain A (carbon salmon) and chain A. The flavin is shown with carbon colored in magenta. The water molecules are shown as red spheres. The dashed lines, full lines, and blue dotted line mark hydrogen bonds, CH-π interactions, and the hydrogen bond stabilizing the position of the His564 imidazole ring, respectively. The graphics was created with the Pymol Molecular Graphics System (Schrödinger). (b) 2D schematic interaction diagram of the active site of *Ct*FDO:IPEA. Hydrogen bonds are indicated as dashed or dotted lines and the CH-π interactions as green dashed lines. Residues with vdW contacts are marked with semicircles. The water molecule labelled W is shown with all hydrophilic interactions.

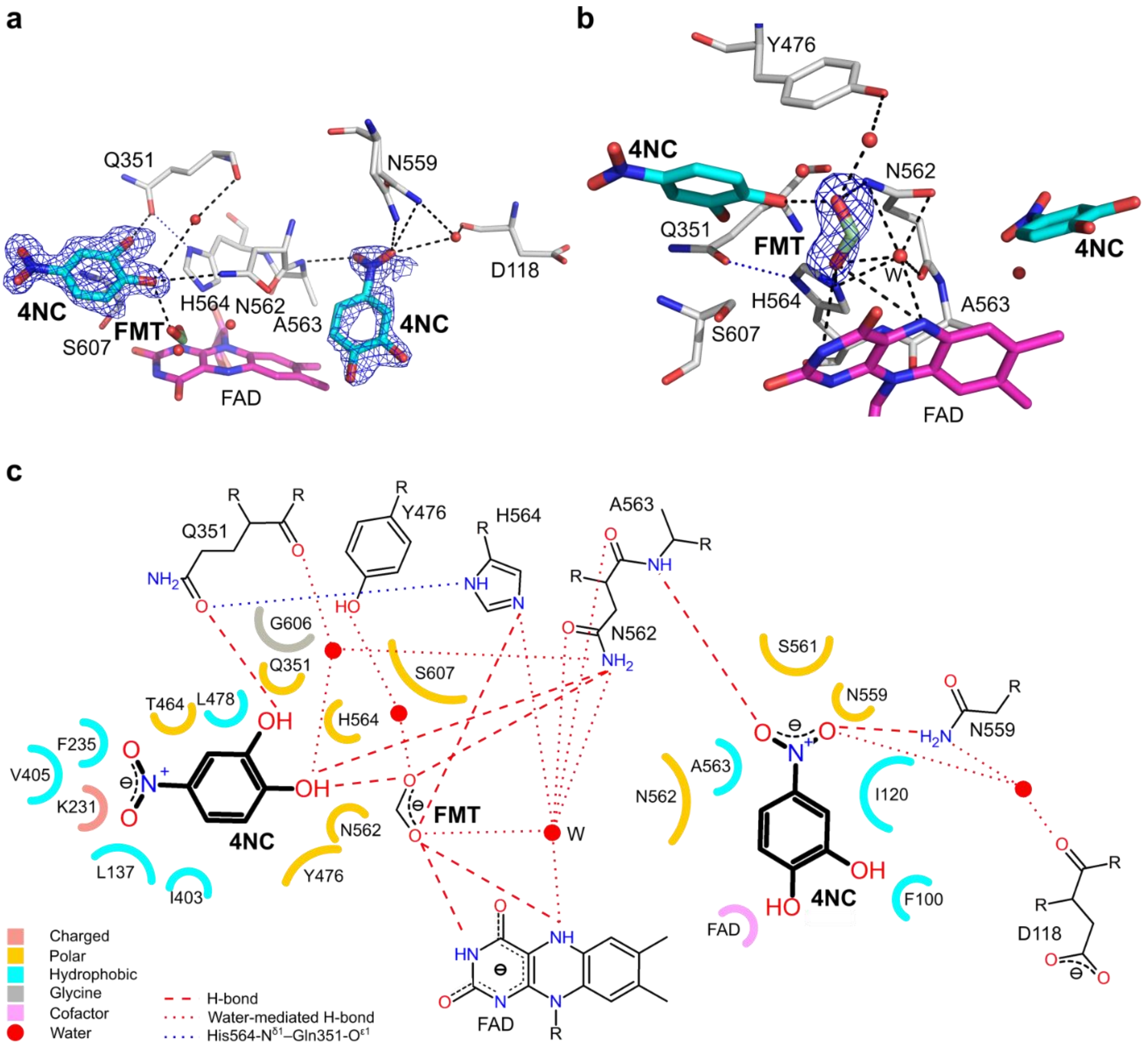

Figure S16 The active-site pocket of *Ct*FDO:4NC (PDB entry: 6ZE6, chain A). Simulated-annealing 2mF_O-DF_C composite omit map shown for (a) the 4NC molecules and (b) the formic acid (FMT) molecule contoured at 1σ level (calculated in Phenix, Terwilliger *et al.*, 2008). The complex (residues with gray carbon atoms) binds FMT (carbon palegreen) and two 4NC molecules (carbon cyan) in the active-site pocket. The cofactor is shown with carbon coloured in magenta. The water molecules (red spheres) and shown with the most probable hydrogen bonds, the water molecule labelled W is shown with all hydrophilic contacts. The dashed lines and blue dotted line mark hydrogen bonds and the hydrogen bond stabilizing the position of the His564 imidazole ring, respectively. The graphics was created with the Pymol Molecular Graphics System (Schrödinger). (c) The 2D schematic interaction diagram of the active site of *Ct*FDO:4NC. Hydrogen bonds are indicated as dashed or dotted lines. Residues with vdW contacts are marked with semicircles.

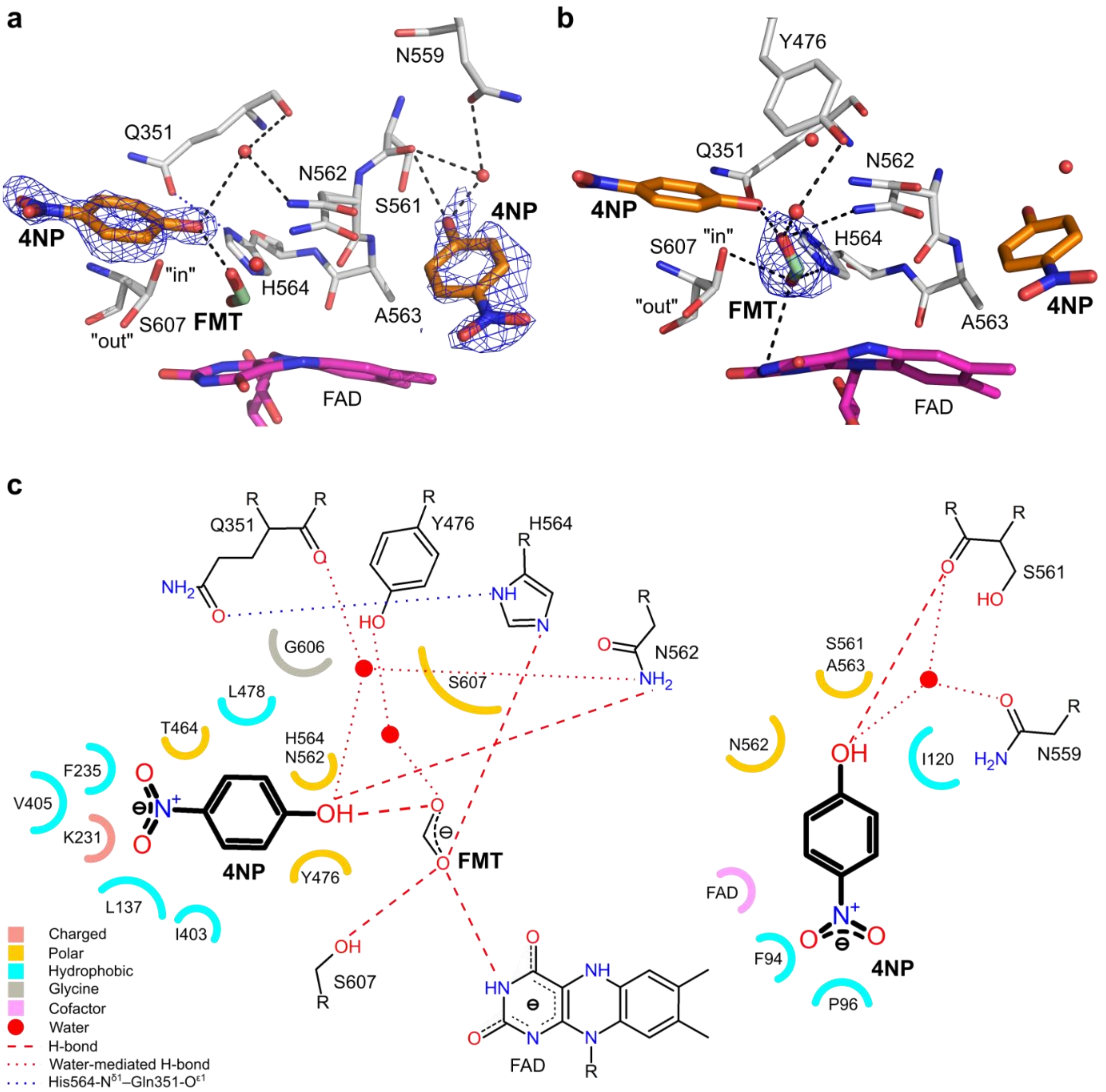

Figure S17 The active-site pocket of *Ct*FDO:4NP (PDB entry: 6ZE7, chain A). Simulated-annealing $2mF_O$-DF_C composite omit map shown for (a) the 4NP molecules and for (b) the FMT molecule contoured at 0.8σ level (calculated in Phenix, Terwilliger *et al.*, 2008). The complex (chain A, residues with gray carbon atoms) binds FMT (carbon palegreen) and two 4NP molecules (carbon orange) in the active-site pocket. The cofactor is shown with magenta carbon atoms. The water molecules are shown as red spheres. The dashed lines and blue dotted line mark hydrogen bonds and the hydrogen bond stabilizing the position of the His564 imidazole ring, respectively. The graphics was created in the Pymol Molecular Graphics System (Schrödinger). (c) 2D schematic interaction diagram of the *Ct*FDO:4NP. Hydrogen bonds are indicated as dashed or dotted lines. Residues with vdW contacts are marked with semicircles.

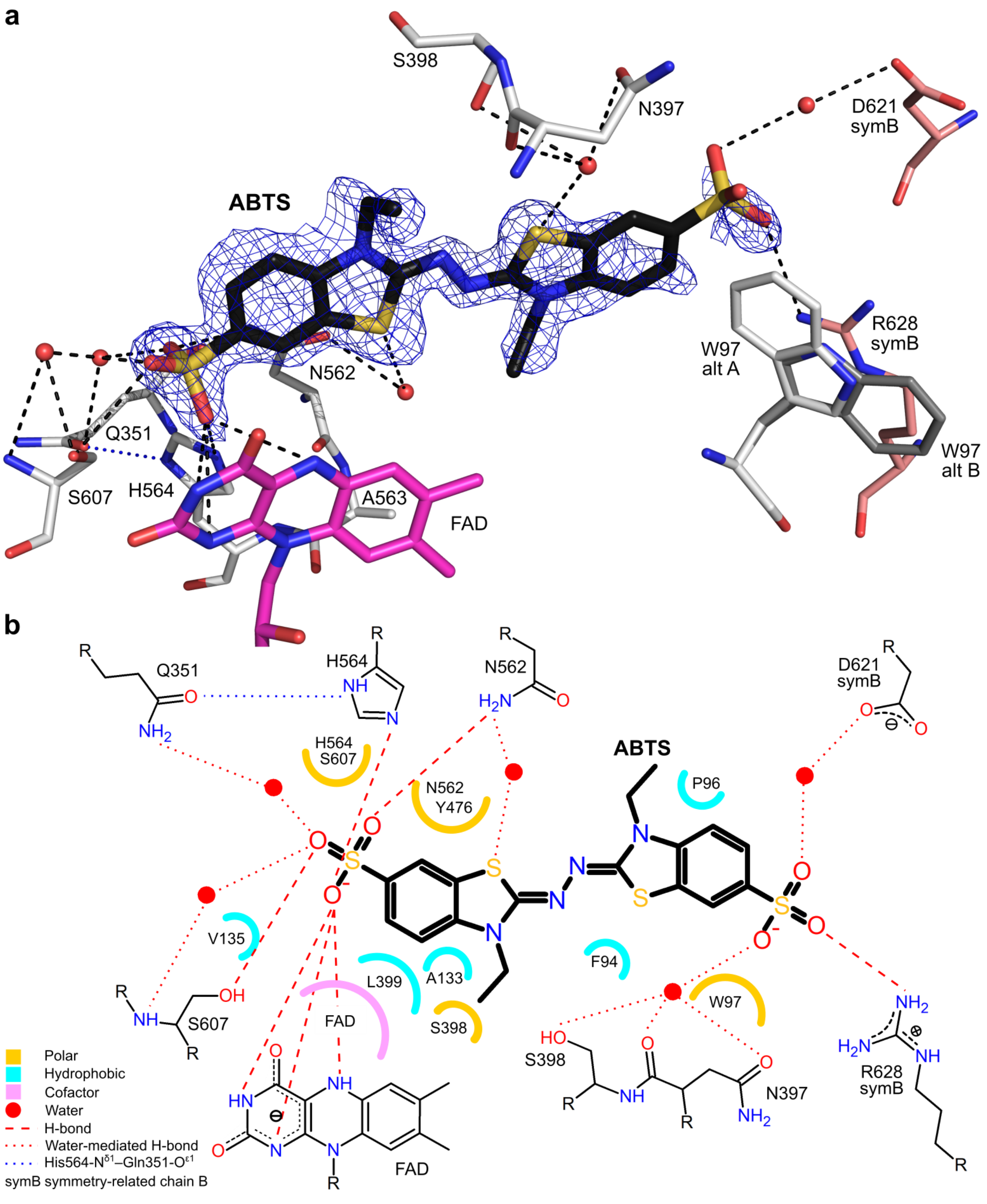

Figure S18 The active-site pocket of *Ct*FDO:ABTS (PDB entry: 7AA2, chain A). (a) Simulated-annealing 2mF_O-DF_C composite omit map shown for ABTS contoured at 1σ level (calculated in Phenix, Terwilliger *et al.*, 2008). The residues are shown with light gray carbon atoms, ABTS with carbon in black, and the cofactor in magenta. The water molecules are shown as red spheres. The dashed lines and blue dotted line mark hydrogen bonds and the hydrogen bond stabilizing the position of His564 imidazole ring, respectively. Residue Trp97 was modelled in two alternative conformations alt A (carbon light grey) and alt B (carbon gray). The graphics was created in the Pymol Molecular Graphics System (Schrödinger). (b) 2D schematic interaction diagram of the active site of *Ct*FDO:ABTS. Hydrogen bonds are indicated as dashed or dotted lines. Residues with vdW contacts are marked with semicircles.

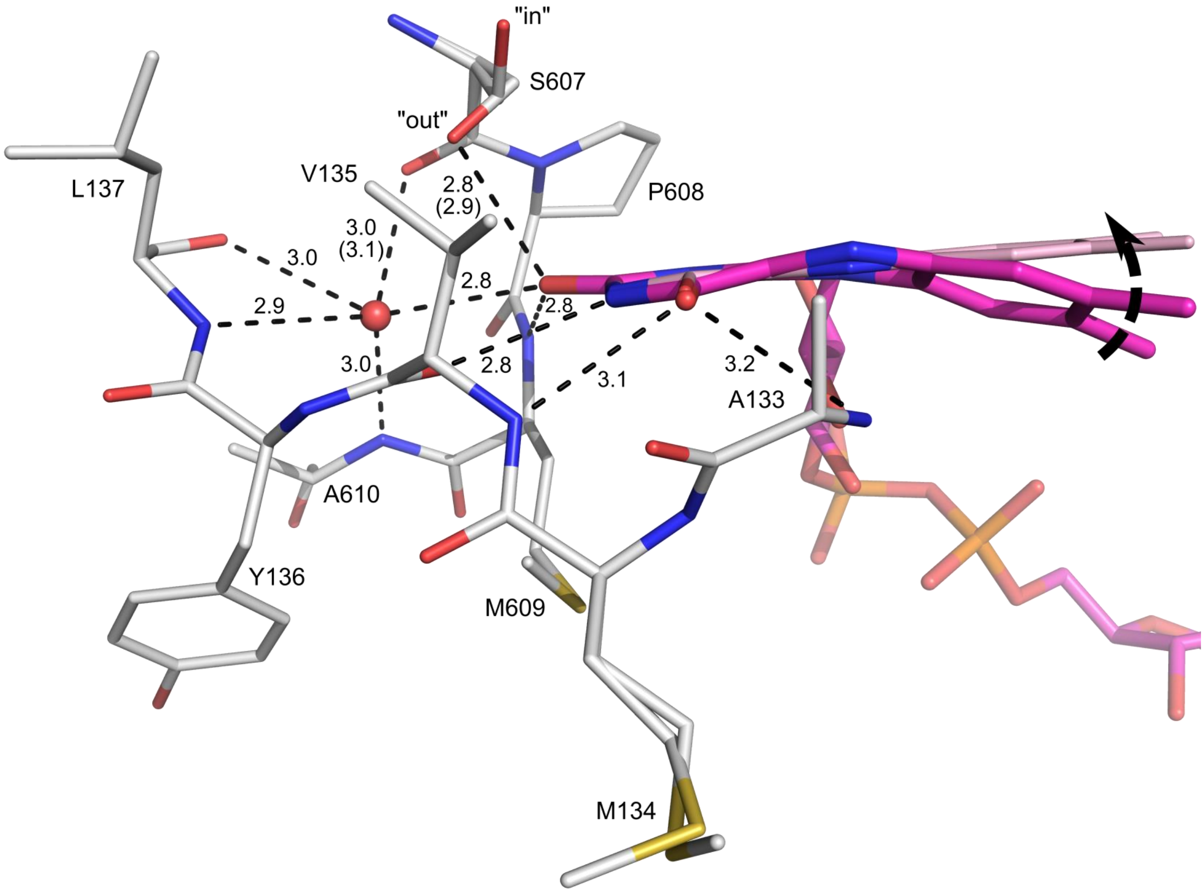

Figure S19 The FAD pyrimidine moiety anchoring in *Ct*FDO via hydrogen bonds with the main chain of the Ala133-Leu137 and Ser607-Ala610 peptides (carbon light grey) and the side chain of the "out" conformer of Ser607 (*Ct*FDO:free, PDB entry: 6ZE2). The cofactor is shown with magenta carbons. The accomplishment of structural changes between the oxidized and reduced forms of the FAD isoalloxazine ring is not clear. One of the possibilities, movement of the dimethylbenzene moiety of FAD (accompanied by structural changes of the surrounding protein residues), is indicated by black arrow and theoretical conformation of planar isoalloxazine ring (carbon pink). The water molecule mediating hydrogen bonds between the FAD pyrimidine moiety and the peptides is shown as red sphere and is shown with all hydrophilic contacts. Hydrogen bonds of other atoms are indicated as dashed lines with labelled distances in Å (different values for chain B are given in parentheses). The graphics was created in Pymol Molecular Graphics System (Schrödinger).

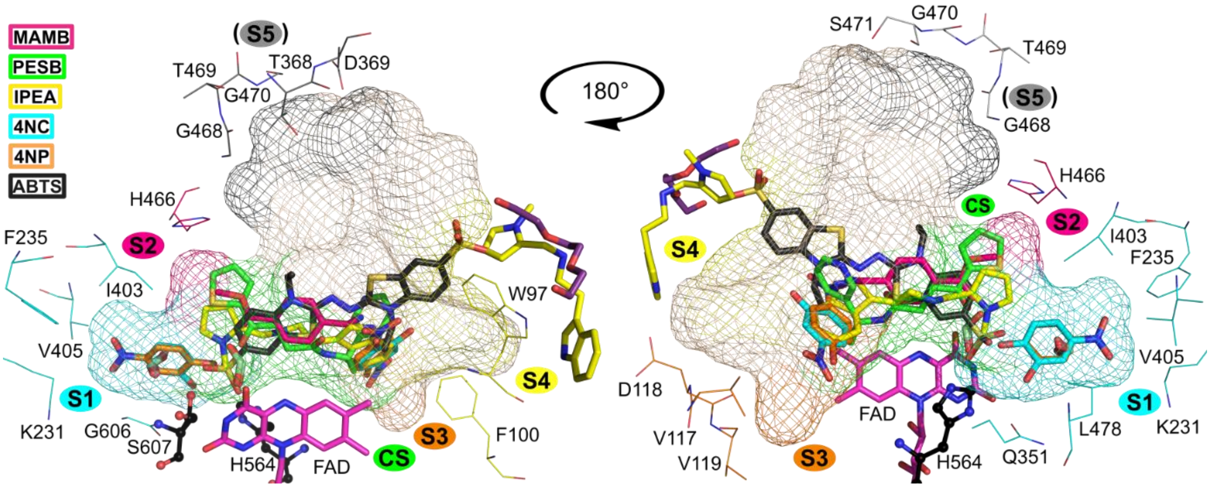

Figure S20 Three-dimensional superposition of the active-sites of all *Ct*FDO complexes (*Ct*FDO:MAMB, *Ct*FDO:PESB, *Ct*FDO:IPEA, *Ct*FDO:4NC, *Ct*FDO:4NP, and *Ct*FDO:ABTS). The superposed ligands are shown in the active-site pocket of *Ct*FDO:free. The pocket (calculated with HOLLOW, Ho & Gruswitz, 2008) is depicted in mesh representation with highlighted catalytic site CS (green) and five sub-sites S1 (cyan), S2 (hot pink), S3 (orange), S4 (yellow), and hypothetical S5 (grey). The selected surrounding residues are shown as lines with carbon coloured by colour corresponding to the sub-site. The His-Ser pair (His564 and Ser607) is shown in ball and stick representation (carbon black). The FAD cofactor (carbon magenta), MAMB (carbon hotpink), PESB (carbon green), IPEA (carbon yellow), 4NC (carbon cyan), 4NP (carbon orange), ABTS (carbon dark grey), formic acid (carbon palegreen), and triethylene glycol (carbon purple) are shown as sticks. Molecular graphics was created using PyMOL (Schrödinger).

Table S3 Detailed ligand interactions with the *Ct*FDO in *Ct*FDO:ligand complexes.

IPEA$_1$ and IPEA$_2$ mark IPEA binding in the active-site pocket and on the interface between two molecules of *Ct*FDO, respectively. 4NC$_1$ and 4NC$_2$ mark 4NC binding in S1 subsite and in the entrance to the pocket, respectively. 4NP$_1$ and 4NP$_2$ mark 4NC binding in S1 subsite, respectively. * and ⋆ mark CH–π interaction and interaction with a residue of a symmetry related chain, respectively. The bond lengths in parentheses are given for chain B. In the case of two conformations of a residue, a range of bond lengths is given.

hydrogen bond	Distance (Å)	Water-mediated bond	Distance (Å)	Van der Waals
MAMB				
MAMB-O–Tyr476-OH	3.2	MAMB-O^1–W$_{AS}$– FAD-N^5	2.5– W$_{AS}$ –3.5	flavin, Ala133, Leu399, Tyr476, Asn562
		MAMB-O^1–W$_{AS}$–FAD-O^4	2.5– W$_{AS}$ –2.6	
		MAMB-O^1–W$_{AS}$–FAD-N^3	2.5– W$_{AS}$ –3.2	
		MAMB-O^1–W$_{AS}$– Ser607-O$^\gamma$ (χ1=–65°)	2.5– W$_{AS}$ –3.5	
		MAMB-N–HOH–Asn562-O	3.6–HOH–3.4	
		MAMB-N–HOH– Asn562-O$^{\delta1}$	3.6–HOH–3.0	
PESB				
PESB-N–Asn562-O$^{\delta1}$	2.8	PESB-O^1– W$_{AS}$–FAD-N^3	2.3 (2.5)–W$_{AS}$–3.5 (3.3)	flavin, Ala133, Val135, Leu399, His466, Leu474, Tyr476, Asn562
		PESB-O^1– W$_{AS}$– His564-N$^{\varepsilon2}$	2.3 (2.5)–W$_{AS}$–3.0 (2.9)	
		PESB-O^1– W$_{AS}$–Ser607-O$^\gamma$ (χ1=–65°)	2.3 (2.5)–W$_{AS}$–2.9	
		PESB-O^1–W$_{AS}$–FAD-O^4	2.3 (2.5)–W$_{AS}$–3.3	
IPEA				
IPEA$_1$-N^1–Asn562-O$^{\delta1}$	3.0 (3.2)	IPEA$_1$-N^1–HOH–Asn562-O	2.9 (2.7)–HOH–3.0	flavin, Phe94, Pro96, Ile120, Ala133, Val135, Ile403, Tyr476, Asn562, His564, Ser607
IPEA$_1$-N^1– Tyr476-OH	2.9 (2.8)	IPEA$_1$-N^1–HOH–Asn562-O$^{\delta1}$	2.9 (2.7)–HOH–2.8 (3.1)	
IPEA$_1$-pyrrole–Tyr476*	3.3 (3.4)	IPEA$_1$-N^1–HOH–Asn562-N$^{\delta2}$	2.9 (2.7)–HOH–3.4 (3.5)	
		IPEA$_1$-N^1–HOH–FAD-N^5	2.9 (2.7)–HOH–3.1 (2.9)	
		IPEA$_1$-N^1–HOH–His564-N$^{\varepsilon2}$	2.9 (2.7)–HOH–2.7 (3.0)	
IPEA$_2$-pyrrole–Trp97*,⋆	3.3	IPEA$_2$-N^1–HOH–Leu629-O	3.0–HOH–2.6	Leu98⋆, Trp97⋆, Asp621, Lys624, Lys625, Arg628, Leu629
		IPEA$_2$-N^1–HOH–Glu621-O	3.1–HOH–2.3	
		IPEA$_2$-N^2–HOH–Lys625-N	3.1–HOH–3.5	
		IPEA$_2$-N^2–HOH–Trp97-O⋆	2.9–HOH–2.8	
4NC				
4NC$_1$-O^8–Gln351-O$^{\varepsilon1}$	2.9	4NC$_1$-O^7–HOH–Gln351-O	3.5 (3.6)–HOH–2.9 (3.0)	Leu137, Lys231, Phe235, Gln351, Ile403, Val405, Thr464, Tyr476, Leu478, Asn562, His564, Gly606, Ser607
4NC$_1$-O^7– Asn562-N$^{\delta2}$	3.3 (3.4)	4NC$_1$-O^7–HOH–Asn562-N$^{\delta2}$	3.5 (3.6)–HOH–2.9 (2.8)	
4NC$_1$-O^7–FMT-O^1	2.6			
4NC$_2$-O^{11}–Ala563-N	3.1 (3.5)	4NC$_2$-O^{10}–HOH–Asn559-N$^{\varepsilon2}$	3.1 (2.9)–HOH–3.1-3.8 (3.0)	flavin, Phe100, Ile120, Ser561, Asn562, Ala563, Asn559
4NC$_2$-O^{10}–Asn559-N$^{\delta2}$	3.0-3.4 (3.3)	4NC$_2$-O^{10}–HOH–Asp118-O	3.1 (2.9)–HOH–2.7	

4NP				
4NP$_1$-OH–FMT-O^{1}	2.6	4NP$_1$-OH–HOH–Gln351-O	3.6 (3.5)–HOH–3.0	Leu137, Lys231, Phe235, Ile403, Val405, Thr464, Tyr476, Leu478, Asn562, His564, Gly606, Ser607
4NP$_1$-OH–Asn562-N$^{\delta2}$	3.3 (3.4)	4NP$_1$-OH–HOH–Asn562-N$^{\delta2}$	3.6 (3.5)–HOH–2.8-3.5 (2.7-3.6)	
4NP$_2$-OH–Ser561-O	3.1	4NP$_2$-OH–HOH–Asn559-O$^{\delta1}$	2.9	flavin, Phe94, Pro96, Ile120, Ser561-Ala563 peptide
		4NP$_2$-OH–HOH–Ser561-O	2.9	
ABTS				
ABTS-O^{47}–FAD-N^{1}	3.5 (3.6)	ABTS-O^{46}–HOH–Gln351-O$^{\varepsilon1}$	2.9–HOH–3.2 (3.3)	
ABTS-O^{47}–FAD-N^{3}	3.1	ABTS-S^{18}–HOH–Asn397-O$^{\delta1}$	3.3 (3.6)–HOH–3.5 (3.4)	
ABTS-O^{47}–FAD-N^{5}	3.4	ABTS-S^{18}–HOH–Asn397-O	3.3 (3.6)–HOH–2.9 (3.0)	flavin, Phe94, Pro96, Trp97, Ala133, Val135, Ser398, Leu399, Tyr476, Asn562, His564, Ser607
ABTS-O^{47}–His564-N$^{\varepsilon2}$	2.6 (2.7)	ABTS-S^{18}–HOH–Ser398-O$^{\gamma}$	3.3 (3.6)–HOH–3.4 (3.7)	
ABTS-O^{45}–Ser607-O$^{\gamma}$	3.3 (3.4)	ABTS-S^{9}–HOH–Asn562-O$^{\delta1}$	3.5 (3.6)–HOH–3.0 (3.2)	
ABTS-O^{46}–Asn562-N$^{\delta2}$	3.0	ABTS-S^{9}–HOH–Asn562-O	3.5 (3.6)–HOH–3.2 (3.3)	
ABTS-O^{39}–Arg628-N$^{\eta2\star}$	3.4 (-)	ABTS-O^{45}–HOH–Ser607-O$^{\gamma}$	3.0–HOH–3.0 (2.8)	
		ABTS-O^{45}–HOH– Ser607-N	3.0–HOH–3.6 (3.4)	
		ABTS-O^{48}–HOH–Asp621-O$^{\delta2\star}$	3.3 (-)–HOH–3.1 (-)	

References

Hajizadeh, N. R., Franke, D., Jeffries, C. M. & Svergun, D. I. (2018). *Sci. Rep.* **8**, 7204.

Ho, B. K. & Gruswitz, F. (2008). *BMC Struct. Biol.* **8**, 49.

Terwilliger, T. C., Grosse-Kunstleve, R. W., Afonine, P. V., Moriarty, N. W., Adams, P. D., Read, R. J., Zwart, P. H. & Hung, L. W. (2008). *Acta Crystallogr. Sect. D Biol. Crystallogr.* **64**, 515–524.
