# Supplementary material for: Crystallographic fragment screening-based study of a novel FAD-dependent oxidoreductase from Chaetomium thermophilum
Source: Acta Crystallogr D Struct Biol. 2021 May 14;77(Pt 6):755–75. doi: 10.1107/S2059798321003533 (PMC8171062; doi:10.1107/S2059798321003533)
Supplement: Supplementary file 2 [file d-77-00755-sup2.pdf]

STRUCTURAL
BIOLOGY

Volume 77 (2021)

Supporting information for article:

Crystallographic fragment screening-based study of novel FAD-dependent oxidoreductase from *Chaetomium thermophilum*

Leona Švecová, Lars Henrik Øestergaard, Tereza Skálová, Kirk Matthew Schnorr, Tomáš Koval', Petr Kolenko, Jan Stránský, David Sedlák, Jarmila Dušková, Mária Trundová, Jindřich Hašek and Jan Dohnálek

Supporting information 2

Crystallographic fragment screening-based study of novel FAD-dependent oxidoreductase from *Chaetomium thermophilum*

Authors

Leona Švecová[ab]*, Lars H. Østergaard[c], Tereza Skálová[a], Kirk Schnorr[c], Tomáš Koval'[a], Petr Kolenko[ab], Jan Stránský[a], David Sedlák[d], Jarmila Dušková[a], Mária Trundová[a], Jindřich Hašek[a] and Jan Dohnálek[a]*

[a]Institute of Biotechnology of the Czech Academy of Sciences v.v.i., Průmyslová 595, Vestec, 25250, Czech Republic

[b]Faculty of Nuclear Sciences and Physical Engineering, Czech Technical University in Prague, Břehová 7, Prague 1, 11519, Czech Republic

[c]Novozymes A/S, Biologiens Vej 2, Kgs. Lyngby, 2800, Denmark

[d]CZ-OPENSCREEN: National Infrastructure for Chemical Biology, Institute of Molecular Genetics of the Czech Academy of Sciences v.v.i., Vídeňská 1083, Prague, 14220, Czech Republic

Correspondence email: leona.svecova@ibt.cas.cz; jan.dohnalek@ibt.cas.cz

High-throughput screening with oxidase activity assay coupled to luciferase signal detection

The high-throughput activity screening of 990 selected compounds (Support. Information 2 Table S2) was performed using the ROS-Glo^{TM} H_2O_2 Assay (Promega). Compounds of molecular weight of 101-1550 g·mol^{-1} containing at least one oxidizable OH group were selected. The reactions (3.5 µl total volume) ran with two concentrations of test compounds (10 µM and 50 µM) and 0.5 µM *Ct*FDO (in 25 mM Tris-HCl, pH 7.5 with 100 mM NaCl) in two different buffers (50 mM Tris-HCl, pH 7.5 and 50 mM Bis-Tris, pH 6) at 37 °C. First, 3.5 µl of *Ct*FDO diluted in the reaction buffer was dispensed by Multidrop Combi (Thermo Fisher Scientific) to white solid polystyrene 1536-well microplates with non-binding surface modification (cat. n. 3729, Corning Costar). 17.5 nl or 2.5 nl of 10 mM screened compounds prediluted in DMSO to 10 mM were transferred to assay plates using contact-free acoustic transfer by ECHO 550 (Labcyte, Inc., USA) integrated in the fully automated robotic HTS station cell::explorer (Perkin Elmer). At least 22 wells on each assay plate were dedicated to different assay controls permitting to control the specificity of detected activities. These controls were 1) reaction buffer without enzyme and test compounds and 2) enzyme diluted in the reaction buffer without test compounds. Plates were vigorously shaken, spun down, and incubated for 16 hours at 37 °C. At the end of the incubation period, plates were taken out of the incubator and left for 15 min at room temperature. H_2O_2 was dispensed to wells containing reaction buffer only to the final concentration of 10 µM, thus creating a positive control for the ROS-Glo^{TM} H_2O_2 Assay. Next, 1 µl of H_2O_2 substrate was dispensed to all wells, plates were shaken and incubated for another 60 min at laboratory temperature. Finally, 4 µl of ROS-Glo™ Detection Solution supplemented

with fresh L-Cysteine just prior to use, was dispensed to plates and 20 min later, luminescence was recorded on the multimode plate reader Envision (Perkin Elmer) equipped with an enhanced luminescence module. Data were collected, processed and normalized using the proprietary LIMS system ScreenX.

The screening yielded 20 compounds with molecular weight between 170 and 479 g·mol^{-1} and signal in the range between 10 % and 130 % of the positive control represented by 10 µM H_2O_2 added to reaction buffer without enzyme (Support. Information 2 Fig. S1). Positive signal occurred for all 20 compounds at pH 7.5 while only for 8 compounds at pH 6.0.

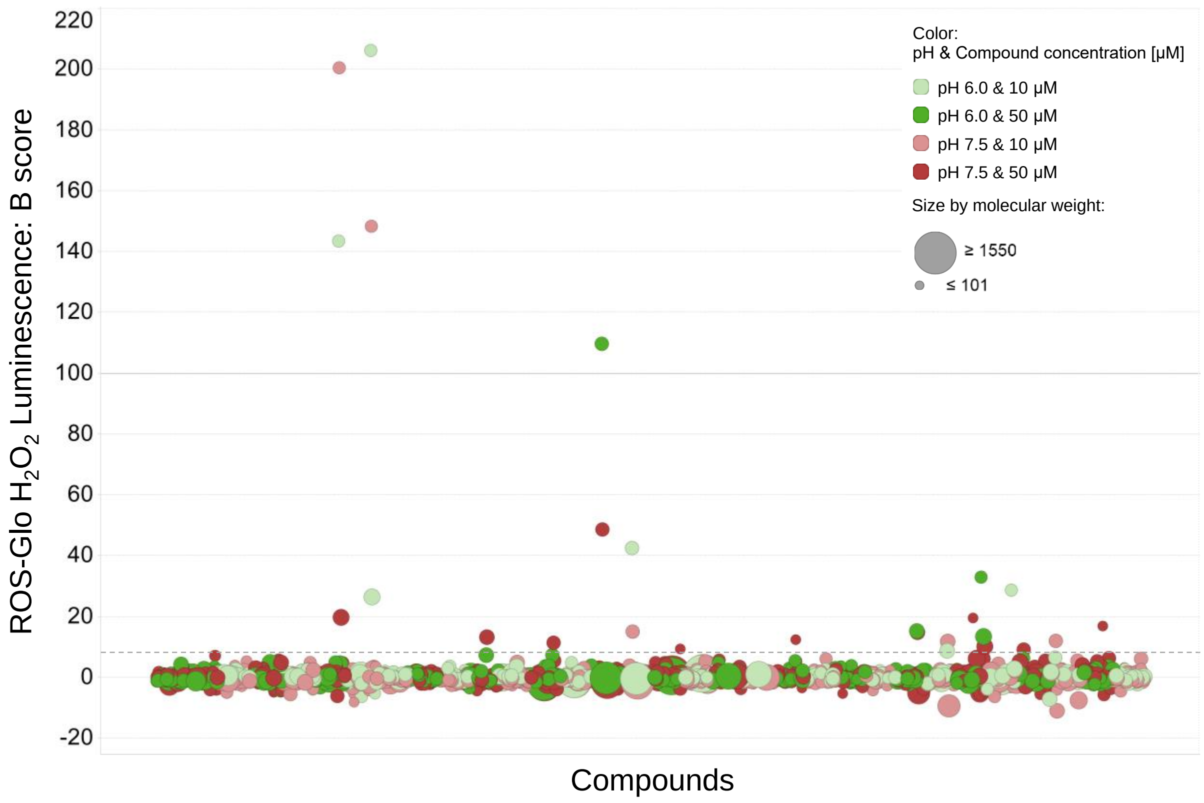

Figure S1 HTS screening of 1070 small molecules as potential substrates for *Ct*FDO oxidoreductase activity. 10 µM (light colour) and 50 µM (dark colour) compounds were incubated with 0.5 µM *Ct*FDO at pH 6.0 (green) or pH 7.5 (red) for 16 h and ROS species were detected by luminescent ROS-Glo™ H_2O_2 Assay. Data were processed and normalized with the B score algorithm. Circle size corresponds to the molecular weight of individual compounds.

Validation of these 20 hits was carried out exactly as the primary screening with the following modifications. The reaction was run in white solid, low volume, polystyrene 384-well microplates with non-binding surface modification (cat. n. 4513, Corning Costar) in a total volume of 6.5 µl of a reaction buffer containing 50 mM Tris-HCl, pH 7.5. All compounds were tested in triplicates at 50 µM with 0.5 µM *Ct*FDO and without enzyme. The reaction ran for 3, 6, 11 and 24 hours.

The validation experiment revealed that 17 compounds (Support. Information 2 Table S1) induced luciferase signal in both, enzyme-containing and enzyme-lacking, reactions to similar levels. The remaining 3 compounds did not show any significant assay signal in all reaction times and for these compounds the results observed in the primary screening could not be reproduced.

Table S1 List of hits from the primary screening for substrates of *Ct*FDO oxidoreductase activity.

*compounds not showing any significant assay signal in the validation experiment

Traditional name	IUPAC name	Mw [g·mol^{-1}]
Noradrenaline	4-(2-amino-1-hydroxyethyl)benzene-1,2-diol	169.2
Gallic acid	3,4,5-trihydroxybenzoic acid	170.1
α-Methyl-norepinephrine	4-[(1*R*)-2-amino-1-hydroxypropyl]benzene-1,2-diol	183.2
Racepinephrine hydrochloride	4-[1-hydroxy-2-(methylamino)ethyl]benzene-1,2-diol; hydrochloride	219.7
6-fluoro-noradrenaline hydrochloride	4-(2-amino-1-hydroxyethyl)-5-fluorobenzene-1,2-diol; hydrochloride	223.6
Isoproterenol hydrochloride	4-1-[hydroxy-2-[(propan-2-yl)amino]ethyl]benzene-1,2-diol; hydrochloride	247.7
Retinol	3,7-dimethyl-9-(2,6,6-trimethylcyclohex-1-en-1-yl)nona-2,4,6,8-tetraen-1-ol	286.5
Benserazide hydrochloride	2-amino-3-hydroxy-*N*'-[(2,3,4-trihydroxyphenyl)-methyl]-propanehydrazide; hydrochloride	293.7
Noradrenaline tartrate	4-[(1*R*)-2-amino-1-hydroxyethyl]benzene-1,2-diol; 2,3-dihydroxybutanedioic acid	319.3
Norepinephrine bitartrate	4-(2-amino-1-hydroxyethyl)benzene-1,2-diol; (2*R*,3*R*)-2,3-dihydroxybutanedioic acid	319.3
Fluorescein	2-(6-hydroxy-3-oxo-3*H*-xanthen-9-yl)benzoic acid	332.3
Isoetharine mesylate	4-[1-hydroxy-2-[(propan-2-yl)amino]butyl]benzene-1,2-diol; methanesulfonic acid	335.4
Isoproterenol tartrate	4-[(1*R*)-1-hydroxy-2-[(propan-2-yl)amino]ethyl]benzene-1,2-diol; (2*S*,3*S*)-2,3-dihydroxybutanedioic acid	361.3
Riboflavin	7,8-dimethyl-10-[(2*S*,3*S*,4*R*)-2,3,4,5-tetrahydroxypentyl]-2*H*,3*H*,4*H*,10*H*-benzo[g]pteridine-2,4-dione	376.4
*Triamcinolone	(8*S*,9*R*,10*S*,11*S*,13*S*,14*S*,16*R*,17*S*)-9-fluoro-11,16,17-trihydroxy-17-(2-hydroxyacetyl)-10,13-dimethyl-6,7,8,11,12,14,15,16-octahydrocyclopenta[a]phenanthren-3-one	394.4
*Fluvastatin sodium	(*E*,3*S*,5*R*)-7-[3-(4-fluorophenyl)-1-propan-2-ylindol-2-yl]-3,5-dihydroxyhept-6-enoate; sodium	433.4
Eltrombopag	3'-[2-[1-(3,4-dimethylphenyl)-3-methyl-5-oxo-4,5-dihydro-1*H*-pyrazol-4-ylidene]hydrazin-1-yl]-2'-hydroxy-[1,1'-biphenyl]-3-carboxylic acid	442.5
AZD8055	[5-[2,4-bis(3-methylmorpholin-4-yl)pyrido[2,3-d]pyrimidin-7-yl]-2-methoxyphenyl]methanol	465.5
CID-2858522	1-(3,5-di-*tert*-butyl-4-hydroxyphenyl)-2-[[2-[(3-hydroxypropyl)amino]-5,6-dimethyl-1,3-benzodiazol-1-yl]ethanone	465.6
*LY411575	2-[[2-(3,5-difluorophenyl)-2-hydroxyacetyl]amino]-*N*-(5-methyl-6-oxo-7*H*-benzo[d][1]benzazepin-7-yl)propanamide	479.5

Table S2 List of compounds selected for the high throughput activity screening with *Ct*FDO.

IUPAC name	Chemical formula	Mw [g·mol^{-1}]
2-amino-3-hydroxy-propanoic acid	$C_3H_7NO_3$	105.1
(pyridin-3-yl)-methanol	C_6H_7NO	109.1
(1*H*-imidazol-5-yl)-methanol; hydrochloride	$C_4H_7ClN_2O$	134.6
1,3-benzodioxol-5-ylmethanol	$C_8H_8O_3$	152.2
3-(2,5-dimethyl-pyrrol-1-yl)-propan-1-ol	$C_9H_{15}NO$	153.1
(2-methylsulfanyl-phenyl)-methanol	$C_8H_{10}OS$	154.2
4-(2-hydroxy-ethoxy)-phenol	$C_8H_{10}O_3$	154.2
2-(aminomethyl)-3-(furan-2-yl)-propan-1-ol	$C_8H_{13}NO_2$	155.2
2-(hydroxy-methyl)-5-methoxy-pyran-4-one	$C_7H_8O_4$	156.2
2-[cyclohexyl-(methyl)-amino]-ethanol	$C_9H_{19}NO$	157.1
N-cyclohexyl-2-hydroxyacetamide	$C_8H_{15}NO_2$	157.2
1-(azepan-1-yl)-2-hydroxy-ethanone	$C_8H_{15}NO_2$	157.2
2-hydroxy-1-(4-methyl-piperazin-1-yl)-ethanone	$C_7H_{14}N_2O_2$	158.2
2-(piperazin-1-yl)-butan-1-ol	$C_8H_{18}N_2O$	158.2
3-(piperidin-4-yloxy)-propan-1-ol	$C_8H_{17}NO_2$	159.2
2-indazol-1-ylethanol	$C_9H_{10}N_2O$	162.2
4-(2-hydroxy-ethylamino)-benzonitrile	$C_9H_{10}N_2O$	162.2
(1-methylbenzotriazol-5-yl)methanol	$C_8H_9N_3O$	163.2
(2-amino-1,3-dihydroinden-2-yl)methanol	$C_{10}H_{13}NO$	163.2
4-(C-ethyl-*N*-hydroxycarbonimidoyl)phenol	$C_9H_{11}NO_2$	165.2
2-amino-3-phenyl-butan-1-ol	$C_{10}H_{15}NO$	165.2
N-(2-hydroxyethyl)pyridine-4-caboxamide	$C_8H_{10}N_2O_2$	166.2
methyl 6-(hydroxy-methyl)-pyridine-3-carboxylate	$C_8H_9NO_3$	167.2
4-[1-hydroxy-2-(methylamino)-ethyl]-phenol	$C_9H_{13}NO_2$	167.2
4-(2-amino-1-hydroxy-ethyl)-benzene-1,2-diol	$C_8H_{11}NO_3$	169.2
2-(hydroxy-methyl)-pyrrolidine-3,4-diol; hydrochloride	$C_5H_{12}ClNO_3$	169.6
3,4,5-trihydroxy-benzoic acid	$C_7H_6O_5$	170.1
2-(2-methyl-5-nitro-1*H*-imidazol-1-yl)-ethanol	$C_6H_9N_3O_3$	171.2
2-(3-chloro-phenoxy)-ethanol	$C_8H_9ClO_2$	172.6
(2-methylquinolin-4-yl)methanol	$C_{11}H_{11}NO$	173.2
(1-phenyl-1*H*-pyrazol-4-yl)-methanol	$C_{10}H_{10}N_2O$	174.2
(2-phenyl-1,3-oxazol-4-yl)-methanol	$C_{10}H_9NO_2$	175.2
3-(hydroxymethyl)-1*H*-quinolin-2-one	$C_{10}H_9NO_2$	175.2
2-(1,2-dihydroxy-ethyl)-3,4-dihydroxy-2*H*-furan-5-one	$C_6H_8O_6$	176.1
2-(hydroxy-methyl)-1-methyl-piperidine-3,4,5-triol	$C_7H_{15}NO_4$	177.2
2-[4-(2-hydroxy-ethoxy)-phenyl]-acetonitrile	$C_{10}H_{11}NO_2$	177.2
2-(3,4-dihydro-1*H*-isoquinolin-2-yl)ethanol	$C_{11}H_{15}NO$	177.2

3-([1,2,4]triazolo[1,5-a]-pyrimidin-2-yl)-propan-1-ol	$C_8H_{10}N_4O$	178.2
(2-methyl-1,3-benzothiazol-5-yl)methanol	C_9H_9NOS	179.2
1-(4-(2-hydroxy-ethyl)-phenylamino)-acetamide	$C_{10}H_{13}NO_2$	179.2
(2-thiophen-2-yl-1,3-oxazol-5-yl)-methanol	$C_8H_7NO_2S$	181.2
4-(2-hydroxy-ethylamino)-benzoic acid	$C_9H_{11}NO_3$	181.2
3-(5-ethoxy-pyridin-3-yl)-propan-1-ol	$C_{10}H_{15}NO_2$	181.2
3-(2-methyl-phenoxy)-propane-1,2-diol	$C_{10}H_{14}O_3$	182.2
(2,6-dimethylimidazo[2,1-b][1,3]-thiazol-5-yl)meyhanol	$C_8H_{10}N_2OS$	182.3
4-[(1*R*)-2-amino-1-hydroxy-propyl]-benzene-1,2-diol	$C_9H_{13}NO_3$	183.2
2-hydroxyethyl(trimethyl)azanium; bromide	$C_5H_{14}BrNO$	184.1
(5-[(2-methylpropan-2-yl)-oxymethyl]-1,2-oxazol-3-yl)-methanol	$C_9H_{15}NO_3$	185.2
4-(2-amino-1-hydroxy-ethyl)-phenol; hydron; chlorid	$C_8H_{12}ClNO_2$	189.6
[5-(3-methylphenyl)-1,3,4-oxadiazol-2-yl]-methanol	$C_{10}H_{10}N_2O_2$	190.2
(4-phenyl-cyclohexyl)-methanol	$C_{13}H_{18}O$	190.3
N-(2-hydroxyethyl)-3-phenyl-prop-2-enamine	$C_{11}H_{13}NO_2$	191.2
4-(hydroxy-methyl)-1-phenyl-pyrrolidin-2-one	$C_{11}H_{13}NO_2$	191.2
(2-phenyl-1,3-thiazol-5-yl)-methanol	$C_{10}H_9NOS$	191.3
(4-phenyl-1,3-thiazol-2-yl)-methanol	$C_{10}H_9NOS$	191.3
2-[(4-propan-2-ylphenyl)methylideneamino]ethanol	$C_{12}H_{17}NO$	191.3
[2-(methylamino)-3,4-dihydro-1*H*-naphthalen-2-yl]methanol	$C_{12}H_{17}NO$	191.3
3-(1-hydroxybutan-2-yliminomethyl)phenol	$C_{11}H_{15}NO_2$	193.2
4-(1-hydroxybutan-2-yliminomethyl)phenol	$C_{11}H_{15}NO_2$	193.2
[2-(morpholin-4-yl)-phenyl]-methanol	$C_{11}H_{15}NO_2$	193.2
2-(2-methyl-1,3-benzothiazol-5-yl)ethanol	$C_{10}H_{11}NOS$	193.3
2-(benzylamino)-3-methylbutan-1-ol	$C_{12}H_{19}NO$	193.3
6-methylamino-hexane-1,2,3,4,5-pentol	$C_7H_{17}NO_5$	195.2
2-[4-(*N*-hydroxy-*C*-methylcarbonimidoyl)phenoxy]ethanol	$C_{10}H_{13}NO_3$	195.2
2-[benzyl(2-hydroxyethyl)amino]ethanol	$C_{11}H_{17}NO_2$	195.3
N-[(2-fluorophenyl)methylideneamino]-2-hydroxyacetamide	$C_9H_9FN_2O_2$	196.2
2-[2-(2-hydroxy-ethoxy)-phenoxy]-ethanol	$C_{10}H_{14}O_4$	198.1
2-hydroxy-2-(4-hydroxy-3-methoxy-phenyl)-acetic acid	$C_9H_{10}O_5$	198.2
3-(2-methoxy-phenoxy)-propane-1,2-diol	$C_{10}H_{14}O_4$	198.2
1-(2-hydroxyethyl)-5-propylpyrazole-4-carboxylic acid	$C_9H_{14}N_2O_3$	198.2
(4-benzylphenyl)-methanol	$C_{14}H_{14}O$	198.3
3-chloro-6-(hydroxymethyl)-oxane-2,4,5-triol	$C_6H_{11}ClO_5$	198.6
2-[2-(4-fluoro-phenoxy)-ethylamino]-ethanol	$C_{10}H_{14}FNO_2$	199.1
2-[2-(2-fluoro-phenoxy)-ethylamino]-ethanol	$C_{10}H_{14}FNO_2$	199.1
2-[(3-methylcyclohexyl)-propylamino]-ethanol	$C_{12}H_{25}NO$	199.3
2-(hydroxymethyl)-piperidine-3,4,5-triol; hydrochloride	$C_6H_{14}ClNO_4$	199.6

2-[(2-chlorophenyl)-methylamino]-propan-1-ol	$C_{10}H_{14}ClNO$	199.7
(4-pyridin-3-yloxyphenyl)-methanol	$C_{12}H_{11}NO_2$	201.2
[4-(hydroxymethyl)-phenyl]-methanesulfonamide	$C_8H_{11}NO_3S$	201.3
[4-(3,5-dimethylpyrazol-1-yl)-phenyl]-methanol	$C_{12}H_{14}N_2O$	202.3
3-(quinazolin-4-ylamino)propan-1-ol	$C_{11}H_{13}N_3O$	203.2
3-[1-hydroxy-2-(methylamino)-ethyl]-phenol; hydrochloride	$C_9H_{14}ClNO_2$	203.7
2-(2-propylbenzimidazol-1-yl)ethanol	$C_{12}H_{16}$N2O	204.3
[2-*tert*-butyl-5-(hydroxymethyl)-2-methyl-1,3-dioxolan-4-yl] methanol	$C_{10}H_{20}O_4$	204.3
2,4-dihydroxy-*N*-(3-hydroxypropyl)-3,3-dimethylbutanamide	$C_9H_{19}NO_4$	205.3
(4-methyl-2-phenyl-1,3-thiazol-5-yl)-methanol	$C_{11}H_{11}NOS$	205.3
[1-[(3-methylphenyl)methyl]-pyrrolidin-2-yl)-methanol	$C_{13}H_{19}NO$	205.3
3-(1,2,3,4-tetrahydronaphthalen-1-ylamino)propan-1-ol	$C_{13}H_{19}NO$	205.3
4,5-bis(hydroxymethyl)-2-methylpyridin-3-ol; hydron; chloride	$C_8H_{12}ClNO_3$	205.6
2-benzylidene-4-hydroxybutanehydrazide	$C_{11}H_{14}N_2O_2$	206.2
1-(2-hydroxyethyl)-2-(hydroxymethyl)piperidine-3,4,5-triol	$C_8H_{17}NO_5$	207.2
2-[4-(4,5-dihydro-1,3-oxazol-2-yl)-phenoxy)-ethanol	$C_{11}H_{13}NO_3$	207.2
2-hydroxy-*N*-[(2-hydroxyphenyl)methylideneamino]propanamide	$C_{10}H_{12}N_2O_3$	208.2
3-(1,3-benzothiazol-2-ylamino)propan-1-ol	$C_{10}H_{12}N_2OS$	208.3
5-fluoro-2-(2-hydroxyethyl)isoindole-1,3-dione	$C_{10}H_8FNO_3$	209.2
2-(5-amino-3-thiophen-2-ylpyrazol-1-yl)-ethanol	$C_9H_{11}N_3OS$	209.3
1-(2-hydroxyethyl)-3-(4-methylphenyl)thiourea	$C_{10}H_{14}N_2OS$	210.3
4-amino-1-[5-(hydroxymethyl)-oxolan-2-yl)-pyrimidin-2-one	$C_9H_{13}N_3O_3$	211.2
2-[2-(2-methoxyphenoxy)-ethylamino]-ethanol	$C_{11}H_{17}NO_3$	211.3
2-[(2,5-dimethoxyphenyl)-methylamino)-ethanol	$C_{11}H_{17}NO_3$	211.3
2-[(4-fluorophenyl)-methyl-propylamino]-ethanol	$C_{12}H_{18}FNO$	211.3
[5-ethyl-2-(furan-2-yl)-1,3-dioxan-5-yl]-methanol	$C_{11}H_{16}O_4$	212.2
4-(3-hydroxypropylsulfanyl)-benzoic acid	$C_{10}H_{12}O_3S$	212.3
tert-butyl 2-[4-(hydroxymethyl)triazol-1-yl]acetate	$C_9H_{15}N_3O_3$	213.2
4-chloro-*N*-(3-hydroxypropyl)-benzamide	$C_{10}H_{12}ClNO_2$	213.7
N-(2-hydroxyethyl)-2-(2-nitroimidazol-1-yl)acetamide	$C_7H_{10}N_4O_4$	214.2
4-(2-hydroxyethoxy)quinolone-3-carbonitrile	$C_{12}H_{10}N_2O_2$	214.2
N-(2-hydroxyethyl)-4-methylbenzenesulfonamide	$C_9H_{13}NO_3S$	215.3
N-benzyl-2-hydroxyethanesulfonamide	$C_9H_{13}NO_3S$	215.3
(1-benzyl-3,5-dimethylpyrazol-4-yl)-methanol	$C_{13}H_{16}N_2O$	216.3
ethyl-[2-hydroxy-2-(3-hydroxyphenyl)ethyl]azanium; chloride	$C_{10}H_{16}ClNO_2$	217.7
5-(hydroxy-methyl)-1,4-dimethyl-2-phenylpyrazol-3-one	$C_{12}H_{14}N_2O_2$	218.3
4-(2-amino-1-hydroxyethyl)-2-methoxyphenol; hydron; chloride	$C_9H_{14}ClNO_3$	219.1
[1-(2-phenylethylamino)-cyclopentyl]-methanol	$C_{14}H_{21}NO$	219.3
[1-(methylamino)-4-phenylcyclohexyl)-methanol	$C_{14}H_{21}NO$	219.3

4-[1-hydroxy-2-(methylamino)-ethyl]-benzene-1,2-diol; hydrochloride	$C_9H_{14}ClNO_3$	219.7
5-(difluoromethyl)-1-(2-hydroxyethyl)-3-methylpyrazole-4-carboxylic acid	$C_8H_{10}F_2N_2O_3$	220.2
[1-(4-fluorophenyl)-3,5-dimethylpyrazol-4-yl]-methanol	$C_{12}H_{13}FN_2O$	220.2
[2-(2-methylanilino)-1,3-thiazol-4-yl)-methanol	$C_{11}H_{12}N_2OS$	220.3
N-[[4-(dimethylamino)-phenyl]-methylideneamino]-2-hydroxyacetamide	$C_{11}H_{15}N_3O_2$	221.3
2-[2,6-di(propan-2-yl)-anilino]-ethanol	$C_{14}H_{23}NO$	221.3
N-(2-hydroxyethyl)-N'-(4-methylphenyl)-oxamide	$C_{11}H_{14}N_2O_3$	222.2
benzyl *N*-(1-hydroxy-2-methylpropan-2-yl)-carbamate	$C_{12}H_{17}NO_3$	223.3
[1-(4-fluoropheyl)-methyl]-piperidin-3-yl)-methanol	$C_{13}H_{18}FNO$	223.3
2-[3-(2,3-dimethylphenoxy)-propylamino]-ethanol	$C_{13}H_{21}NO_2$	223.3
4-(2-amino-1-hydroxyethyl)-5-fluorobenzene-1,2-diol; hydrochloride	$C_8H_{11}ClFNO_3$	223.6
1-[5-(hydroxymethyl)-2,5-dihydrofuran-2-yl)-5-methylpyrimidine-2,4-dione	$C_{10}H_{12}N_2O_4$	224.2
7-(2-hydroxyethyl)-1,3-dimethylpurine-2,6-dione	$C_9H_{12}N_4O_3$	224.2
3-(4-phenoxyphenyl)-prop-2-yn-1-ol	$C_{15}H_{12}O_2$	224.3
2-amino-9-(2-hydroxyethoxymethyl)-1*H*-purin-6-one	$C_8H_{11}N_5O_3$	225.2
3-hydroxy-2-(3-hydroxypropylaminomethyl)-5,5-dimethyl-cyclohex-2-en-1-one	$C_{12}H_{19}NO_3$	225.3
1-(4-fluorophenyl)-3-(1-hydroxybutan-2-yl)-urea	$C_{11}H_{15}FN_2O_2$	226.3
2-[(4-chlorophenyl)-methyl-propylamino)-ethanol	$C_{12}H_{18}ClNO$	227.7
2-hydroxy-*N*-(napthalen-1-ylmethylideneamino)-acetamide	$C_{13}H_{12}N_2O_2$	228.3
6-ethyl-4-(hydroxymethyl)-1,3-dimethyl-3a,6a-dihydroimidazo[4,5-d]imidazole-2,5-dione	$C_9H_{16}N_4O_3$	228.3
4-amino-1-[2-(hydroxymethyl)-1,3-oxathiolan-5-yl]-pyrimidin-2-one	$C_8H_{11}N_3O_3S$	229.3
2-[(4-chlorophenyl)-methyl-(2-hydroxyethyl)-amino]-ethanol	$C_{11}H_{16}ClNO_2$	229.7
4-(2,5-dimethylpyrrol-1-yl)-2-hydroxybenzoic acid	$C_{13}H_{13}NO_3$	231.3
[5-(1,3-bezothiazol-2-yl)-furan-2-yl]-methanol	$C_{12}H_9NO_2S$	231.3
(3-chlorocarbazol-9-yl)-methanol	$C_{13}H_{10}ClNO$	231.7
2-[(5-nitroquinolin-8-yl)-amino]-ethanol	$C_{11}H_{11}N_3O_3$	233.2
4-(2-hydroxyethyl)-4-azatetracyclo[5.3.2.0^{2,6}.0^{8,10}]-dodec-11-ene-3,5-dione	$C_{13}H_{15}NO_3$	233.3
1-(4-cyanophenyl)-3-(1-hydroxybutan-2-yl)urea	$C_{12}H_{15}N_3O_2$	233.3
4-[1-hydroxy-2-(methylamino)-ethyl]-2-methoxyphenol; hydrochloride	$C_{10}H_{16}ClNO_3$	233.7
2-hydroxy-*N*-[(4-prop-2-enoxyphenyl)-methylideneamino]-acetamide	$C_{12}H_{14}N2O_3$	234.3
3-[(2-hydroxy-1*H*-indol-3-yl)-methylideneamino]-propane-1,2-diol	$C_{12}H_{14}N_2O_3$	234.3
4-(4-ethyl-1-hydroxy-5,5-dimethyl-2*H*-imidazol-2-yl)-phenol	$C_{13}H_{18}N_2O_2$	234.3
(3-amino-phenyl)-(3-(hydroxy-methyl)-piperidin-1-yl)-methanone	$C_{13}H_{18}N_2O_2$	234.3
(3-amino-phenyl)-(4-(hydroxy-methyl)-piperidin-1-yl)-methanone	$C_{13}H_{18}N_2O_2$	234.3
2-(4-(3-methyl-benzyl)-piperazin-1-yl)-ethanol	$C_{14}H_{22}N_2O$	234.3

2-(4-phenethyl-piperazin-1-yl)-ethanol	$C_{14}H_{22}N_2O$	234.3
2-(2-hydroxyethyl)-1,3-dioxoisoindole-5-carboxylic acid	$C_{11}H_9NO_5$	235.2
[2-(4-ethoxyphenyl)-1,3-thiazol-4-yl]-methanol	$C_{12}H_{13}NO_2S$	235.3
3-[(6-ethoxy-1H-benzimidazol-2-yl)-amino]-propan-1-ol	$C_{12}H_{17}N_3O_2$	235.3
[2,4,6-triethyl-3-(hydroxyiminomethyl)-phenyl]-methanol	$C_{14}H_{21}NO_2$	235.3
2-[(4-propan-2-ylphenyl)-methyl-propylamino]-ethanol	$C_{15}H_{25}NO$	235.4
[6-(4-chlorophenoxy)-pyridin-3-yl]-methanol	$C_{12}H_{10}ClNO_2$	235.7
methyl 4-[[(2-hydroxyacetyl)-hydrazinylidene]-methyl]benzoate	$C_{11}H_{12}N_2O_4$	236.2
9-[5-(hydroxymethyl)-oxolan-2-yl)-1*H*-purin-6-one	$C_{10}H_{12}N_4O_3$	236.2
3-(4-phenylpiperazin-1-yl)-propane-1,2-diol	$C_{13}H_{20}N_2O_2$	236.3
2-[(4-dimethylaminophenyl)-methyl)-propylamino)-ethanol	$C_{14}H_{24}N_2O$	236.4
2-[4-(2-bicyclo[2.2.1]-hept-5-enylmethyl)-piperazin-1-yl]-ethanol	$C_{14}H_{24}N_2O$	236.4
[1-(5-nitro-pyridin-2-yl)-piperidin-3-yl]-methanol	$C_{11}H_{15}N_3O_3$	237.3
N-(2-hydroxyethyl)tricyclo[4.3.1.1^{3,8}]-undecane-1-carboxamide	$C_{14}H_{23}NO_2$	237.3
2-[(4-ethoxyphenyl)-methyl)-propylamino]-ethanol	$C_{14}H_{23}NO_2$	237.3
2-[2-hydroxyethyl-[(4-propan-2-ylphenyl)-methyl]-amino]-ethanol	$C_{14}H_{23}NO_2$	237.3
(1-benzylbenzimidazol-2-yl)-methanol	$C_{15}H_{14}N_2O$	238.3
3-(2-methoxy-4-prop-2-enylphenoxy)-propane-1,2-diol	$C_{13}H_{18}O_4$	238.3
2-[(4-nitrophenyl)-methyl-propylamino]-ethanol	$C_{12}H_{18}N_2O_3$	238.3
1-(1-hydroxybutan-2-yl)-3-(4-methoxyphenyl)-urea	$C_{12}H_{18}N_2O_3$	238.3
2-[4-[(4-fluorophenyl)-methyl]-piperazin-1-yl]-ethanol	$C_{13}H_{19}FN_2O$	238.3
2-[[4-(dimethylamino)-phenyl]-methyl-(2-hydroxyethyl)-amino]-ethanol	$C_{13}H_{22}N_2O_2$	238.3
[3-chloro-4-(difluoromethoxy)-5-methoxyphenyl]-methanol	$C_9H_9ClF_2O_3$	238.6
4-[2-(*tert*-butylamino)-1-hydroxyethyl]-2-(hydroxymethyl)-phenol	$C_{13}H_{21}NO_3$	239.3
2-[(4-methylsulfanylphenyl)-methyl-propylamino]-ethanol	$C_{13}H_{21}NOS$	239.4
[2-hydroxy-3-(2-methoxyphenoxy)-propyl] carbamate	$C_{11}H_{15}NO_5$	241.2
2-(2,3-dihydroxypropyliminomethyl)-3-hydroxy-5,5-dimethylcyclohex-2-en-1-one	$C_{12}H_{19}NO_4$	241.3
2-(dibenzylamino)-ethanol	$C_{16}H_{19}NO$	241.3
2-[2-hydroxyethyl-[(4-methylsulfanylphenyl)-methyl]-amino]-ethanol	$C_{12}H_{19}NO_2S$	241.4
[3-(3-phenylpropoxy)-phenyl]-methanol	$C_{16}H_{18}O_2$	242.3
1-(3-chloro-4-methylphenyl)-3-(1-hydroxypropan-2-yl)-urea	$C_{11}H_{15}ClN_2O_2$	242.7
4-amino-1-[3,4-dihydroxy-5-(hydroxymethyl)-oxolan-2-yl]-pyrimidin-2-one	$C_9H_{13}N_3O_5$	243.2
2-hydroxy-N-(4-phenoxyphenyl)-acetamide	$C_{14}H_{13}NO_3$	243.3
2-[2-(4-chloro-2-methylphenoxy)-ethyl-methylamino]-ethanol	$C_{12}H_{18}ClNO_2$	243.7
1-[3,4-dihydroxy-5-(hydroxymethyl)-oxolan-2-yl)-1,2,4-triazole-3-carboxamide	$C_8H_{12}N_4O_5$	244.2
4-amino-1-[3,4-dihydroxy-5-(hydroxymethyl)-oxolan-2-yl]-1,3,5-triazin-2-one	$C_8H_{12}N_4O_5$	244.2

2-[3,4-dihydroxy-5-(hydroxymethyl)-oxolan-2-yl]-1,2,4-triazine-3,5-dione	$C_8H_{11}N_3O_6$	245.2
3-[3-(4-fluorophenyl)-4-(hydroxymethyl)-pyrazol-1-yl]-propanenitrile	$C_{13}H_{12}FN_3O$	245.3
2-(3-chlorocarbazol-9-yl)-ethanol	$C_{14}H_{12}ClNO$	245.7
2-[(2-chloro-6-fluorophenyl)-methyl-propylamino]-ethanol	$C_{12}H_{17}ClFNO$	245.7
(1-heptylbenzimidazol-2-yl)-methanol	$C_{15}H_{22}N_2O$	246.4
2-[benzyl(thiophen-2-ylmethyl)-amino]-ethanol	$C_{14}H_{17}NOS$	247.4
2-[benzyl(cyclohexylmethyl)-amino]-ethanol	$C_{16}H_{25}NO$	247.4
4-[1-hydroxy-2-[(propan-2-yl)-amino)-ethyl]-benzene-1,2-diol; hydrochloride	$C_{11}H_{18}ClNO_3$	247.7
2-[4-[(2,4-dimethylphenyl)-methyl]-piperazin-1-yl]-ethanol	$C_{15}H_{24}N_2O$	248.4
2-[4-(3-phenylpropyl)-piperazin-1-yl]-ethanol	$C_{15}H_{24}N_2O$	248.4
2-[4-[(2,5-dimethylphenyl)-methyl]-piperazin-1-yl]-ethanol	$C_{15}H_{24}N_2O$	248.4
2-(anthracen-9-ylmethylideneamino)-ethanol	$C_{17}H_{15}NO$	249.3
2,4-di*tert*-butyl-6-(hydroxyiminomethyl)-phenol	$C_{15}H_{23}NO_2$	249.4
2-[3-(2-methoxyphenyl)-prop-2-enyl-propylamino]-ethanol	$C_{15}H_{23}NO_2$	249.4
2-(2-hydroxyethyl)-7,8-dimethoxyphthalazin-1-one	$C_{12}H_{14}N_2O_4$	250.3
[3-(1*H*-indol-3-ylmethylideneamino)-phenyl]-methanol	$C_{16}H_{14}N_2O$	250.3
2-[4-[(3-methoxyphenyl)-methyl]-piperazin-1-yl]-ethanol	$C_{14}H_{22}N_2O_2$	250.3
tert-butyl *N*-[3-(3-hydroxypropyl)-phenyl]-carbamate	$C_{14}H_{21}NO_3$	251.3
2-[(6-ethylthieno[2,3-d]-pyrimidin-4-yl)-amino]-butan-1-ol	$C_{12}H_{17}N_3OS$	251.4
2-(hydroxymethyl)-5-purin-9-yloxolane-3,4-diol	$C_{10}H_{12}N_4O_4$	252.2
2-amino-9-[4-hydroxy-3-(hydroxymethyl)-butyl]-1*H*-purin-6-one	$C_{10}H_{15}N_5O_3$	253.3
7-(2,3-dihydroxypropyl)-1,3-dimethylpurine-2,6-dione	$C_{10}H_{14}N_4O_4$	254.2
(7-methyl-6,6a,8,9-tetrahydro-4*H*-indolo[4,3-fg]-quinolin-9-yl)-methanol	$C_{16}H_{18}N_2O$	254.3
2-[4-[(4-chlorophenyl)methyl]-piperazin-1-yl]-ethanol	$C_{13}H_{19}ClN_2O$	254.8
2-amino-9-(1,3-dihydroxypropan-2-yloxymethyl)-1*H*-purin-6-one	$C_9H_{13}N_5O_4$	255.2
N-(2-hydroxyethyl)-2,2-diphenylacetamide	$C_{16}H_{17}NO_2$	255.3
5-(3,4-dimethylanilino)-pentane-1,2,3,4-tetrol	$C_{13}H_{21}NO_4$	255.3
2-[benzyl-[(2-methylphenyl)-methyl]-amino]-ethanol	$C_{17}H_{21}NO$	255.4
2-[benzyl(2-phenylethyl)-amino]-ethanol	$C_{17}H_{21}NO$	255.4
N-[(4-bromophenyl)-methylideneamino]-2-hydroxyacetamide	$C_9H_9BrN_2O_2$	257.1
N-[(3-bromophenyl)-methylideneamino]-2-hydroxyacetamide	$C_9H_9BrN_2O_2$	257.1
[3-[(2-nitrophenyl)-methylideneamino]-phenyl]-methanol	$C_{14}H_{12}N_2O_3$	256.3
[1-[(2-fluorophenyl)-methyl]-benzimidazol-2-yl]-methanol	$C_{15}H_{13}FN_2O$	256.3
2-(4-chlorophenoxy)-*N*-(1-hydroxy-2-methylpropan-2-yl)-acetamide	$C_{12}H_{16}ClNO_3$	257.7
2-(hydroxymethyl)-5-phenyl-3*H*-thieno[2,3-d]-pyrimidin-4-one	$C_{13}H_{10}N_2O_2S$	258.3
1-(3,4-dihydroxy-5-(hydroxymethyl)-oxolan-2-yl)-5-hydroxyimidazole-4-carboxamide	$C_9H_{13}N_3O_6$	259.2
bis(4-methylphenyl)-phosphorylmethanol	$C_{15}H_{17}O_2P$	260.3

3-acetyl-4-hydroxy-1-(2-hydroxyethyl)-2-phenyl-2*H*-pyrrol-5-one	$C_{14}H_{15}NO_4$	261.3
[3-(naphthalen-2-ylmethylideneamino)-phenyl]-methanol	$C_{18}H_{15}NO$	261.3
4-(hydroxymethyl)-10-imino-3,7-dioxa-1,9-diazatricyclo[6.4.0.0^{2,6}]dodeca-8,11-dien-5-ol; hydrochloride	$C_9H_{12}ClN_3O_4$	261.7
2-[(3,4-dichlorophenyl)-methyl-propylamino]-ethanol	$C_{12}H_{17}Cl_2NO$	262.2
2-[(2,3-dichlorophenyl)-methyl-propylamino]-ethanol	$C_{12}H_{17}Cl_2NO$	262.2
4-hydroxy-*N*-(2-hydroxyethyl)-1-methyl-2-oxoquinoline-3-carboxamide	$C_{13}H_{14}N_2O_4$	262.3
N-[1-(2-hydroxyethylamino)-3-methyl-1-oxobut-2-en-2-yl]-benzamide	$C_{14}H_{18}N_2O_3$	262.3
2-[(2,6-dichlorophenyl)-methyl-(2-hydroxyethyl)-amino]-ethanol	$C_{11}H_{15}Cl_2NO_2$	264.1
2-[(3,4-dichlorophenyl)-methyl-(2-hydroxyethyl)-amino]-ethanol	$C_{11}H_{15}Cl_2NO_2$	264.1
[3-ethoxy-2-[(5-methyl-1,2-oxazol-3-yl)-methoxy]-phenyl]-methanol	$C_{14}H_{17}NO_4$	263.3
2-(3,5-diphenylpyrazol-1-yl)-ethanol	$C_{17}H_{16}N_2O$	264.3
2-[4-[(4-ethoxyphenyl)-methyl]-piperazin-1-yl]-ethanol	$C_{15}H_{24}N_2O_2$	264.4
2-[4-[(4-prop-1-en-2-ylcyclohexen-1-yl)-methyl]-piperazin-1-yl]-ethanol	$C_{16}H_{28}N_2O$	264.4
1-methyl-1-nitroso-3-[2,4,5-trihydroxy-6-(hydroxymethyl)-oxan-3-yl]-urea	$C_8H_{15}N_3O_7$	265.2
2-[4-[(3-nitrophenyl)-methyl]-piperazin-1-yl]-ethanol	$C_{13}H_{19}N_3O_3$	265.3
2-[4-[(2-nitrophenyl)-methyl]-piperazin-1-yl]-ethanol	$C_{13}H_{19}N_3O_3$	265.3
2-[4-[(4-nitrophenyl)-methyl]-piperazin-1-yl]-ethanol	$C_{13}H_{19}N_3O_3$	265.3
3-(hydroxymethyl)-5-[(4-methylphenyl)-methylidene]-2-sulfanylidene-1,3-thiazolidin-4-one	$C_{12}H_{11}NO_2S_2$	265.4
4-hydroxy-*N*-[(4-hydroxy-3-methoxyphenyl)-methylideneamino]-pentanamide	$C_{13}H_{18}N_2O_4$	266.3
2-(2-nitro-5-piperazin-1-ylanilino)-ethanol	$C_{12}H_{18}N_4O_3$	266.3
2-[4-[(4-methylsulfanylphenyl)-methyl]-piperazin-1-yl]-ethanol	$C_{14}H_{22}N_2OS$	266.4
[1-[(4-bromophenyl)-methyl]-pyrazol-4-yl]-methanol	$C_{11}H_{11}BrN_2O$	267.1
2-(6-aminopurin-9-yl)-5-(hydroxymethyl)-oxolane-3,4-diol	$C_{10}H_{13}N_5O_4$	267.2
N-(4-chlorophenyl)-3-(hydroxymethyl)-piperidine-1-carboxamide	$C_{13}H_{17}ClN_2O_2$	268.7
1-[(2-hydroxyethylamino)-methyl]-benzo[f]-chromen-3-one	$C_{16}H_{15}NO_3$	269.3
2-(6-aminopurin-9-yl)-5-(hydroxymethyl)-oxolan-3-ol; hydrate	$C_{10}H_{15}N_5O_4$	269.3
2-[benzyl-[(2,5-dimethylphenyl)-methyl]-amino]-ethanol	$C_{18}H_{23}NO$	269.4
2-(3-butyl-2-iminobenzimidazol-1-yl)-ethanol; hydrochloride	$C_{13}H_{20}ClN_3O$	269.8
5-bromo-2-(2-hydroxyethyl)-isoindole-1,3-dione	$C_{10}H_8BrNO_3$	270.1
2-hydroxy-ethyl 4-benzoylbenzoate	$C_{16}H_{14}O_4$	270.3
2-[(3-bromophenyl)-methyl-propylamino]-ethanol	$C_{12}H_{18}BrNO$	272.2
2-[(4-bromophenyl)-methyl-propylamino]-ethanol	$C_{12}H_{18}BrNO$	272.2
2-(hydroxymethyl)-6-(4-hydroxyphenoxy)-oxane-3,4,5-triol	$C_{12}H_{16}O_7$	272.3
2-[[3-(hydroxymethyl)-phenyl]-iminomethyl]-4-nitrophenol	$C_{14}H_{12}N_2O_4$	272.3
2-[(4-bromophenyl)-methyl-(2-hydroxyethyl)-amino]-ethanol	$C_{11}H_{16}BrNO_2$	273.0

2-[4-[1-(furan-2-yl)-ethylamino]-pyrazolo[3,4-d]-pyrimidin-1-yl]-ethanol	$C_{13}H_{15}N_5O_2$	273.3
N-[(2,4-dichlorophenyl)-methylideneamino]-4-hydroxybutanamide	$C_{11}H_{12}Cl_2N_2O_2$	275.1
3-acetyl-4-hydroxy-1-(3-hydroxypropyl)-2-phenyl-2*H*-pyrrol-5-one	$C_{15}H_{17}NO_4$	275.3
2-[4-(2-hydroxyethyl)-pyrazol-1-yl]-*N*-(4-methoxyphenyl)-acetamide	$C_{14}H_{17}N_3O_3$	275.3
2-(4-methoxy-2,3-dihydrofuro[2,3-b]-quinolin-2-yl)-propane-1,2-diol	$C_{15}H_{17}NO_4$	275.3
2-[benzyl-[(2-chlorophenyl)-methyl]-amino]-ethanol	$C_{16}H_{18}ClNO$	275.8
hydron; 2-[2-(1-hydroxybutan-2-ylamino)-ethylamino]-butan-1-ol; dichloride	$C_{10}H_{26}Cl_2N_2O_2$	277.2
4-amino-1-[3,4-dihydroxy-5-(hydroxymethyl)-oxolan-2-yl]-pyrimidin-2-one; hydrochloride	C_9H14ClN3O5	279.7
[3-[(2,4-dichlorophenyl)-methylideneamino]-phenyl]-methanol	$C_{14}H_{11}Cl_2NO$	280.1
1-(2,3-dihydro-1,4-benzodioxin-6-yl)-3-(4-hydroxy-3-methylbutan-2-yl)-urea	$C_{14}H_{20}N_2O_4$	280.3
2-[5-(3-methylpiperazin-1-yl)-2-nitroanilino]-ethanol	$C_{13}H_{20}N_4O_3$	280.3
2-(hydroxymethyl)-5-[6-(methylamino)-purin-9-yl]-oxolane-3,4-diol	$C_{11}H_{15}N_5O_4$	281.3
[4-(hydroxymethyl)-piperidin-1-yl]-(5-methyl-4-propylthiophen-2-yl)-methanone	$C_{15}H_{23}NO_2S$	281.4
3-(1-hydroxybutan-2-ylimino)-2-(1-hydroxybutylidene)-5,5-dimethylcyclohexan-1-one	$C_{16}H_{27}NO_3$	281.4
(1-methyl-5-nitro-3-phenylindol-2-yl)-methanol	$C_{16}H_{14}N_2O_3$	282.3
[1-[2-(2-methylphenoxy)-ethyl]-benzimidazol-2-yl]-methanol	$C_{17}H_{18}N_2O_2$	282.3
2-[4-(2-pyridin-2-ylethylamino)-pyrazolo[3,4-d]-pyrimidin-1-yl]-ethanol	$C_{14}H_{16}N_6O$	284.3
N-ethyl-3-hydroxy-2-phenyl-*N*-(pyridine-4-ylmethyl)-propanamide	$C_{17}H_{20}N_2O_2$	284.4
2-[2-(2,4,6-trichlorophenoxy)-ethylamino]-ethanol	$C_{10}H_{12}Cl_3NO_2$	284.6
5-(6-amino-2-chloropurin-9-yl)-2-(hydroxymethyl)oxolan-3-ol	$C_{10}H_{12}ClN_5O_3$	285.7
2-[benzyl-[(3-nitrophenyl)-methyl]-amino]-ethanol	$C_{16}H_{18}N_2O_3$	286.3
3,7-dimethyl-9-(2,6,6-trimethylcyclohex-1-en-1-yl)-nona-2,4,6,8-tetraen-1-ol	$C_{20}H_{30}O$	286.5
2-[benzyl-[(4-methylsulfanylphenyl)-methyl]-amino]-ethanol	$C_{17}H_{21}NOS$	287.4
4-hydroxy-*N*-(2-hydroxyethyl)-2-oxo-1-prop-2-enylquinoline-3-carboxamide	$C_{15}H_{16}N_2O_4$	288.3
13-methyl-6,7,8,9,11,12,14,15,16,17-decahydrocyclopenta[a]-phenanthrene-3,16,17-triol	$C_{18}H_{24}O_3$	288.4
2-[4-[(2,3-dichlorophenyl)-methyl]-piperazin-1-yl]-ethanol	$C_{13}H_{18}Cl_2N_2O$	289.2
(8-methyl-8-azabicyclo[3.2.1]octan-3-yl 3-hydroxy-2-phenyl-propanoate	$C_{17}H_{23}NO_3$	289.2
2-(3,4-dihydroxyphenyl)-3,4-dihydro-2*H*-chromene-3,5,7-triol	$C_{15}H_{14}O_6$	290.3
2-[4-[[(6-methoxypyridin-3-yl)-methylamino]-methyl]-3,5-dimethylpyrazol-1-yl]-ethanol	$C_{15}H_{22}N_4O_2$	290.4
3-acetyl-4-hydroxy-1-(2-hydroxyethyl)-2-(4-methoxyphenyl)-2*H*-pyrrol-5-one	$C_{15}H_{17}NO_5$	291.3
2-[4-[3-(2-nitrophenyl)-prop-2-enyl]-piperazin-1-yl]-ethanol	$C_{15}H_{21}N_3O_3$	291.4

2,6-di*tert*-butyl-4-(3-hydroxy-2,2-dimethylpropyl)-phenol	$C_{19}H_{32}O_2$	292.5
3-bromo-2-hydroxy-5-phenyl-benzoic acid	$C_{13}H_9BrO_3$	293.1
2-[3-acetamido-2,5-dihydroxy-6-(hydroxymethyl)-oxan-4-yl]-oxypropanoic acid	$C_{11}H_{19}NO_8$	293.3
2-amino-3-hydroxy-*N'*-[(2,3,4-trihydroxyphenyl)-methyl]-propanehydrazide; hydrochloride	$C_{10}H_{16}ClN_3O_5$	293.7
N-[6-(hydroxyamino)-6-oxohexoxy]-3,5-dimethylbenzamide	$C_{15}H_{22}N_2O_4$	294.4
2-[2-(2-hydroxyethylamino)-benzimidazol-1-yl]-1-phenylethanone	$C_{17}H_{17}N_3O_2$	295.3
2-[2-(2-chloro-6-nitroanilino)-ethylamino]-ethanol; hydrochloride	$C_{10}H_{15}Cl_2N_3O_3$	296.2
[1-[2-(2,6-dimethylphenoxy)-ethyl]-benzimidazol-2-yl]-methanol	$C_{18}H_{20}N_2O_2$	296.4
17-ethynyl-13-methyl-7,8,9,11,12,14,15,16-octahydro-6*H*-cyclopenta[a]-phenanthrene-3,17-diol	$C_{20}H_{24}O_2$	296.4
2-[[6-(benzylamino)-9-methylpurin-2-yl]-amino]-ethanol	$C_{15}H_{18}N_6O$	298.3
2-[4-[(4-bromophenyl)-methyl]-piperazin-1-yl]-ethanol	$C_{13}H_{19}BrN_2O$	299.2
[5-bromo-2-(3-ethylpiperidin-1-yl)-pyridin-3-yl]-methanol	$C_{13}H_{19}BrN_2O$	299.2
2-[2-(3,4-dimethylphenoxy)-ethylamino]-ethanol; oxalic acid	$C_{14}H_{21}NO_6$	299.3
2-[2-(2,5-dimethylphenoxy)-ethylamino]-ethanol; oxalic acid	$C_{14}H_{21}NO_6$	299.3
2-[2-(2-ethylphenoxy)-ethylamino]-ethanol; oxalic acid	$C_{14}H_{21}NO_6$	299.3
2-amino-9-[3,4-dihydroxy-5-(hydroxymethyl)-oxolan-2-yl]-3*H*-purine-6-thione	$C_{10}H_{13}N_5O_4S$	299.3
N-(2-hydroxyethyl)-hexadecanamide	$C_{18}H_{37}NO_2$	299.5
1-(2-hydroxy-1-phenylethyl)-3-(5-methoxy-2-methylphenyl)-urea	$C_{17}H_{20}N_2O_3$	300.4
bis(4-chlorophenyl)-phosphorylmethanol	$C_{13}H_{11}Cl_2O_2P$	301.1
2-[1-(5-bromopyridin-2-yl)-piperidin-3-yl]-oxyethanol	$C_{12}H_{17}BrN_2O_2$	301.2
[3-[(3,4,5-trimethoxyphenyl)-methylideneamino]-phenyl]-methanol	$C_{17}H_{19}NO_4$	301.3
2-[4-[2-(3-fluorophenyl)-ethylamino]-pyrazolo[3,4-d]-pyrimidin-1-yl]-ethanol	$C_{15}H_{16}FN_5O$	301.3
2-[4-[4-(furan-2-yl)-butan-2-ylamino]-pyrazolo[3,4-d]-pyrimidin-1-yl]-ethanol	$C_{15}H_{19}N_5O_2$	301.3
2-aminooctadecane-1,3-diol	$C_{18}H_{39}NO_2$	301.5
2-(6-amino-2-chloropurin-9-yl)-5-(hydroxymethyl)-oxolane-3,4-diol	$C_{10}H_{12}ClN_5O_4$	301.7
potassium; [4-(1,2-dihydroxyethyl)-2-methoxyphenyl] sulfate	$C_9H_{11}KO_7S$	302.3
sodium; 1-[2-(hydroxymethyl)-4,5-dimethoxyphenyl]-cyclopentane-1-carboxylate	$C_{15}H_{19}NaO_5$	302.3
2-[5-[1-(2-amino-6-methylpyrimidin-4-yl)-piperidin-4-yl]-pyrazol-1-yl]-ethanol	$C_{15}H_{22}N_6O$	302.4
2-methoxy-13-methyl-6,7,8,9,11,12,14,15,16,17-decahydrocyclopenta[a]-phenanthrene-3,17-diol	$C_{19}H_{26}O_3$	302.4
2-[4-[methyl-[(3-methylthiophen-2-yl)-methyl]-amino]-pyrazolo[3,4-d]-pyrimidin-1-yl]-ethanol	$C_{14}H_{17}N_5OS$	303.4
2-[4-[[(4-fluoro-2,3-dihydro-1*H*-inden-1-yl)-amino]-methyl]-3,5-dimethylpyrazol-1-yl]-ethanol	$C_{17}H_{22}FN_3O$	303.4
2-(3,4-dihydroxyphenyl)-3,5,7-trihydroxy-2,3-dihydrochromen-4-one	$C_{15}H_{12}O_7$	304.3

N-[1-(2-hydroxyethyl)-pyrazol-4-yl]-5-thiophen-2-yl-1,2-oxazole-3-carboxamide	$C_{13}H_{12}N_4O_3S$	304.3
[5-(1-benzylindazol-3-yl)furan-2-yl]-methanol	$C_{19}H_{16}N_2O_2$	304.3
2-[5-[1-[(4,5-dimethyl-1,3-oxazol-2-yl)-methyl]-piperidin-4-yl]-pyrazol-1-yl]-ethanol	$C_{16}H_{24}N_4O_2$	304.4
1-(2-chloro-4-methylphenyl)-3-[[2-(hydroxymethyl)-phenyl]-methyl]-urea	$C_{16}H_{17}ClN_2O_2$	304.8
2-[4-[(5-bromothiophen-2-yl)methyl]-piperazin-1-yl]-ethanol	$C_{11}H_{17}BrN_2OS$	305.2
1-(2-hydroxyethyl)-1-[(2-methoxyphenyl)-methyl]-3-(3-methyl-1,2-oxazol-5-yl)-urea	$C_{15}H_{19}N_3O_4$	305.3
2-[4-[(imidazo[2,1-b][1,3]-thiazol-6-ylmethylamino)-methyl]-3,5-dimethylpyrazol-1-yl]-ethanol	$C_{14}H_{19}N_5OS$	305.4
N-[4-ethoxy-3-(hydroxymethyl)-phenyl]-2-ethyl-1,3-thiazole-5-carboxamide	$C_{15}H_{18}N_2O_3S$	306.1
2-[5-[1-[(2-methyl-1,3-thiazol-5-yl)-methyl]-piperidin-4-yl]-pyrazol-1-yl]-ethanol	$C_{15}H_{22}N_4OS$	306.4
5-bromo-1-[4-hydroxy-5-(hydroxymethyl)-oxolan-2-yl]-pyrimidine-2,4-dione	$C_9H_{11}BrN_2O_5$	307.1
2-amino-3-(4-hydroxy-3-iodophenyl)-propanoic acid	$C_9H_{10}INO_3$	307.1
2-[5-[(2-*tert*-butyl-1,3-thiazol-4-yl)-methylamino]-pyridin-2-yl]-oxyethanol	$C_{15}H_{21}N_3O_2S$	307.1
[1-[(2,4-dichlorophenyl)-methyl]-benzimidazol-2-yl]-methanol	$C_{15}H_{12}Cl_2N_2O$	307.2
2-(2-hydroxyethylamino)-3-methyl-5-thiophen-2-ylthieno[2,3-d]-pyrimidin-4-one	$C_{13}H_{13}N_3O_2S_2$	307.4
2-[5-[(2-*tert*-butyl-1,3-thiazol-5-yl)-methylamino]-pyridin-2-yl]-oxyethanol	$C_{15}H_{21}N_3O_2S$	307.4
[2-[[(2-methoxynaphthalen-1-yl)-methylamino]-methyl]-phenyl]-methanol	$C_{20}H_{21}NO_2$	307.4
2-amino-2-[2-(4-octylphenyl)-ethyl]-propane-1,3-diol	$C_{19}H_{33}NO_2$	307.5
5-[[[2-(4-chlorophenyl)-2-hydroxyethyl]-amino]-methyl]-2-methoxyphenol	$C_{16}H_{18}ClNO_3$	307.8
2-(3,4-dihydroxyphenyl)-3,4-dihydro-2*H*-chromene-3,5,7-triol; hydrate	$C_{15}H_{16}O_7$	308.3
N-[[2-(hydroxymethyl)-phenyl]-methyl]-4-oxo-1*H*-quinoline-3-carboxamide	$C_{18}H_{16}N_2O_3$	308.3
2-[5-[(5-methyl-[1,2,4]-triazolo[1,5-a]-pyrimidin-7-yl)-amino]-indol-1-yl]-ethanol	$C_{16}H_{16}N_6O$	308.3
7-[[4-(2-hydroxyethyl)-piperazin-1-yl]-methyl]-3-methyl-[1,3]-thiazolo[3,2-a]-pyrimidin-5-one	$C_{14}H_{20}N_4O_2S$	308.4
[1-[4-[(4-ethylphenyl)-methylamino]-phenyl]-triazol-4-yl]-methanol	$C_{18}H_{20}N_4O$	308.4
2-[4-fluoro-2-(2-hydroxyethoxy)-anilino]-*N*-(3-methyl-1,2-oxazol-5-yl)-acetamide	$C_{14}H_{16}FN_3O_4$	309.3
N-(2-hydroxy-1-phenylethyl)-1-propan-2-ylpyrazole-4-sulfonamide	$C_{14}H_{19}N_3O_3S$	309.4
2-[4-[2,3-dihydro-1*H*-inden-1-yl(methyl)-amino]-pyrazolo[3,4-d]-pyrimidin-1-yl]-ethanol	$C_{17}H_{19}N_5O$	309.4
2-[2-[4-[(4-nitrophenyl)-methyl]-piperazin-1-yl]-ethoxy]-ethanol	$C_{15}H_{23}N_3O_4$	309.4

4-chloro-*N*-(4-hydroxy-3-methylsulfanylbutan-2-yl)-benzenesulfonamide	$C_{11}H_{16}ClNO_3S_2$	309.8
N-[3-(2-hydroxyethylamino)-3-oxo-1-phenylprop-1-en-2-yl]-benzamide	$C_{18}H_{18}N_2O_3$	310.3
2,3-bis(2-hydroxyethylsulfanyl)-naphthalene-1,4-dione	$C_{14}H_{14}O_4S_2$	310.4
2-[(9-ethylcarbazol-3-yl)-methyl-propylamino]-ethanol	$C_{20}H_{26}N_2O$	310.4
2-[[4-[[(5-chloropyridin-2-yl)-amino]-methyl]-2-fluorophenyl]-methoxy]-ethanol	$C_{15}H_{16}ClFN_2O_2$	310.8
N-[2-[(2,5-difluorophenyl)-methyl]-3-hydroxypropyl]-thiophene-2-carboxamide	$C_{15}H_{15}F_2NO_2S$	311.3
2-[5-[(2-phenyltriazol-4-yl)-methylamino]-pyridin-2-yl]-oxyethanol	$C_{16}H_{17}N_5O_2$	311.3
[5-[[(3,5-dimethyl-1-phenylpyrazol-4-yl)-methylamino]-methyl]-furan-2-yl]-methanol	$C_{18}H_{21}N_3O_2$	311.4
[1-(4-ethylphenyl)-1-oxopropan-2-yl] 4-(hydroxymethyl)-benzoate	$C_{19}H_{20}O_4$	312.4
1-[5-[1-(hydroxymethyl)-3,4-dihydro-1*H*-isoquinoline-2-carbonyl]-1-methylpyrrol-3-yl]-ethanone	$C_{18}H_{20}N_2O_3$	312.4
1-(2,6-diethylphenyl)-3-[[2-(hydroxymethyl)-phenyl]-methyl]-urea	$C_{19}H_{24}N_2O_2$	312.4
1-(3-cyano-4-fluorophenyl)-3-(1-hydroxy-3-phenylpropan-2-yl)-urea	$C_{17}H_{16}FN_3O_2$	313.3
N-(3,4-difluorophenyl)-3-[4-(2-hydroxyethyl)-piperazin-1-yl]-propanamide	$C_{15}H_{21}F_2N_3O_2$	313.3
[2-[[(5-methoxy-2-methyl-2,3-dihydro-1-benzofuran-6-yl)-methylamino]-methyl]-phenyl]-methanol	$C_{19}H_{23}NO_3$	313.4
2-[4-[[1-(1-benzofuran-2-yl)-ethylamino]-methyl]-3,5-dimethylpyrazol-1-yl]-ethanol	$C_{18}H_{23}N_3O_2$	313.4
2-[[(5-chloropyrimidin-2-yl)-amino]-methyl]-3-(2,5-difluorophenyl)-propan-1-ol	$C_{14}H_{14}ClF_2N_3O$	313.7
[2-(2-cyanoanilino)-2-oxoethyl] 5-(hydroxymethyl)-2-methylfuran-3-carboxylate	$C_{16}H_{14}N_2O_5$	314.3
3-fluoro-4-[[[2-hydroxy-1-(4-methoxyphenyl)-ethyl]-methylamino]-methyl]-benzonitrile	$C_{18}H_{19}FN_2O_2$	314.4
3-[[2-[(4-fluorophenyl)-methyl]-3-hydroxypropyl]-amino]-5,6-dimethylpyridazine-4-carbonitrile	$C_{17}H_{19}FN_4O$	314.4
4-[[[2-hydroxy-1-(4-methoxyphenyl)-ethyl]-methylamino]-methyl]-benzamide	$C_{18}H_{22}N_2O_3$	314.4
2-[4-(2-thiophen-2-ylpyrrolidin-1-yl)-pyrazolo[3,4-d]-pyrimidin-1-yl]-ethanol	$C_{15}H_{17}N_5OS$	315.4
3-fluoro-*N*-[4-(hydroxymethyl)-cyclohexyl]-*N*,5-dimethylbenzenesulfonamide	$C_{15}H_{22}FNO_3S$	315.4
(5-thiophen-3-yl-1,2,4-oxadiazol-3-yl)-methyl 4-(hydroxymethyl)-benzoate	$C_{15}H_{12}N_2O_4S$	316.3
1-(3-chlorophenyl)-3-[2-(hydroxymethyl)-1,3-dihydroinden-2-yl]-urea	$C_{17}H_{17}ClN_2O_2$	316.8
N-[4-ethoxy-3-(hydroxymethyl)-phenyl]-1-methyl-5-propan-2-ylpyrazole-3-carboxamide	$C_{17}H_{23}N_3O_3$	317.4
4-[2-[2-(3,4-dimethoxy-phenyl)-ethylamino]-1-hydroxy-ethyl]-phenol	$C_{18}H_{23}NO_4$	317.4
2-[benzyl-[(4-phenylphenyl)-methyl]-amino]-ethanol	$C_{22}H_{23}NO$	317.4

2-[4-[(3,4-dihydro-2*H*-thiochromen-4-ylamino)-methyl]-3,5-dimethylpyrazol-1-yl]-ethanol	$C_{17}H_{23}N_3OS$	317.5
1-(3,5-difluorophenyl)-3-[2-(hydroxymethyl)-1,3-dihydroinden-2-yl]-urea	$C_{17}H_{16}F_2N_2O_2$	318.3
1-(4-fluoro-2-methylphenyl)-3-[[2-(2-hydroxyethoxy)-phenyl]-methyl]-urea	$C_{17}H_{19}FN_2O_3$	318.3
N-(1-hydroxybutan-2-yl)-2-[4-(thiophen-3-ylmethylamino)-phenyl]-acetamide	$C_{17}H_{22}N_2O_2S$	318.4
1-[4-[(2-hydroxy-1-phenylethyl)-amino]-piperidin-1-yl]-3,3-dimethylbutan-1-one	$C_{19}H_{30}N_2O_2$	318.5
(2*R*, 3*R*, 4*S*, 5*R*)-2-(5,6-dichlorobenzimidazol-1-yl)-5-(hydromethyl)-oxolane-3,4-diol	$C_{12}H_{12}Cl_2N_2O_4$	319.1
4-[(1*R*)-2-amino-1-hydroxyethyl]-benzene-1,2-diol; 2,3-dihydroxybutanedioic acid	$C_{12}H_{17}NO_9$	319.3
4-(-2-amino-1-hydroxyethyl)-benzene-1,2-diol; (2*R*,3*R*)-2,3-dihydroxybutanedioic acid	$C_{12}H_{17}NO_9$	319.3
1-(3-fluoro-4-methylsulfanylphenyl)-3-[[3-(hydroxymethyl)-phenyl]-methyl]-urea	$C_{16}H_{17}FN_2O_2S$	320.4
6-(hydroxymethyl)-*N*-[(2-hydroxynaphthalen-1-yl)-methylideneamino]-pyridine-3-carboxamide	$C_{18}H_{15}N_3O_3$	321.3
N-[(2,5-dimethoxyphenyl)-methyl]-N-(2-hydroxyethyl)-thiophene-3-carboxamide	$C_{16}H_{19}NO_4S$	321.4
2-[4-[[1-(3-fluoro-4-methoxyphenyl)-ethylamino]-methyl]-3,5-dimethylpyrazol-1-yl]-ethanol	$C_{17}H_{24}FN_3O_2$	321.4
8,16,18-trihydroxy-4-methyl-3-oxabicyclo[12.4.0]-octadeca-1(14),15,17-trien-2-one	$C_{18}H_{26}O_5$	322.2
2-[4-[2-(1*H*-indol-3-yl)-ethylamino]-pyrazolo[3,4-d]-pyrimidin-1-yl]-ethanol	$C_{17}H_{18}N_6O$	322.4
2-[4-[[3-(4-chlorophenyl)-1,2,4-oxadiazol-5-yl]-methyl]-piperazin-1-yl]-ethanol	$C_{15}H_{19}ClN_4O_2$	322.8
2,2-dichloro-*N*-[1,3-dihydroxy-1-(4-nitrophenyl)-propan-2-yl]-acetamide	$C_{11}H_{12}Cl_2N_2O_5$	323.1
N-benzyl-4-bromo-*N*-(2-hydroxyethyl)-1*H*-pyrrole-2-carboxamide	$C_{14}H_{15}BrN_2O_2$	323.2
methyl 4-[[2-(hydroxymethyl)-phenyl]-methylamino]-quinazoline-7-carboxylate	$C_{18}H_{17}N_3O_3$	323.3
2-[5-(1-quinazolin-4-ylpiperidin-4-yl)-pyrazol-1-yl]-ethanol	$C_{18}H_{21}N_5O$	323.4
1-[3-(2-hydroxyethyl)-pyrrolidin-1-yl]-3,3-diphenylpropan-1-one	$C_{21}H_{25}NO_2$	323.4
4-[2-[[1-hydroxy-1-(4-hydroxyphenyl)-propan-2-yl]-amino]-ethyl]-phenol; hydrochloride	$C_{17}H_{22}ClNO_3$	323.8
4-[2-[[(1*R*,2*S*)1-hydroxy-1-(4-hydroxyphenyl)-propan-2-yl]-amino]-ethyl]-phenol; hydrochloride	$C_{17}H_{22}ClNO_3$	323.8
2-[(5-bromopyrimidin-2-yl-)amino]-2-(4-methoxyphenyl)-ethanol	$C_{13}H_{14}BrN_3O_2$	324.2
2-[4-fluoro-2-(2-hydroxyethoxy)-anilino]-*N*-(thiophen-2-ylmethyl)-acetamide	$C_{15}H_{17}FN_2O_3S$	324.4
1-[[3-fluoro-4-(hydroxymethyl)-phenyl]-methyl]-3-(5-propan-2-yl-1,3,4-thiadiazol-2-yl)-urea	$C_{14}H_{17}FN_4O_2S$	324.4

[1-[2-(5-methyl-2-propan-2-ylphenoxy)-ethyl]-benzimidazol-2-yl]-methanol	$C_{20}H_{24}N_2O_2$	324.4
2-[3-(hydroxymethyl)-anilino]-2-phenyl-1-piperidin-1-ylethanone	$C_{20}H_{24}N_2O_2$	324.4
1-[2-(4-fluorophenyl)-2-methoxyethyl]-3-[2-(hydroxymethyl)-cyclohexyl]-urea	$C_{17}H_{25}FN_2O_3$	324.4
3-(2-chloro-4-methylphenyl)-1-(2-hydroxyethyl)-1-(thiophen-3-ylmethyl)-urea	$C_{15}H_{17}ClN_2O_2S$	324.8
N-(2-hydroxyethyl)-octadec-9-enamide	$C_{20}H_{39}NO_2$	325.5
2-[4-[(5-chlorothiophen-2-yl)-methoxy]-phenyl]-*N*-(2-hydroxyethyl)-acetamide	$C_{15}H_{16}ClNO_3S$	325.8
3-[2-(4-chlorophenyl)imidazo[1,2-a]-benzimidazol-3-yl]-propan-1-ol	$C_{18}H_{16}ClN_3O$	325.8
[1-phenyl-3-(4-phenylphenyl)-pyrazol-4-yl]-methanol	$C_{22}H_{18}N_2O$	326.4
2-(12-hydroxy-dodeca-5,10-diynyl)-3,5,6-trimethyl-cyclohexa-2,5-diene-1,4-dione	$C_{21}H_{26}O_3$	326.4
N-[2-(2-chloro-4-methoxyphenyl)-ethyl]-3-(hydroxymethyl)-piperidine-1-carboxamide	$C_{16}H_{23}ClN_2O_3$	326.8
2-(4-(4-hydroxy-3-isopropyl-benzyl)-3,5-dimethyl-phenoxy)-acetic acid	$C_{20}H_{24}O_4$	328.4
[3-[(9-ethylcarbazol-3-yl)-methylideneamino]-phenyl]-methanol	$C_{22}H_{20}N_2O$	328.4
4-fluoro-3-[[4-[2-(2-hydroxyethyl)-pyrazol-3-yl]-piperidin-1-yl]methyl]-benzonitrile	$C_{18}H_{21}FN_4O$	328.4
2-[4-[2-(2-hydroxyethyl)-pyrazol-3-yl]-piperidin-1-yl]-2-phenylacetamide	$C_{18}H_{24}N_4O_2$	328.4
3-[4-(3-imidazol-1-ylpropylamino)-5,6-dimethylpyrrolo[2,3-d]-pyrimidin-7-yl]-propan-1-ol	$C_{17}H_{24}N_6O$	328.4
[2-[4-(4-ethylpiperazin-1-yl)-butoxy]-phenyl]-methanol; hydrochloride	$C_{17}H_{29}ClN_2O_2$	328.9
[5-fluoro-3-(hydroxymethyl)-2-[(1-phenyl-1,2,4-triazol-3-yl)-methoxy]-phenyl]-methanol	$C_{17}H_{16}FN_3O_3$	329.3
3-[3-[bis(2-hydroxyethyl)-sulfamoyl]-4-methylphenyl]-prop-2-enoic	$C_{14}H_{19}NO_6S$	329.4
2-[5-(1-thieno[2,3-d]-pyrimidin-4-ylpiperidin-4-yl)-pyrazol-1-yl]-ethanol	$C_{16}H_{19}N_5OS$	329.4
2-(2,6-dimethylphenoxy)-ethyl 2-(2-hydroxyethylamino)-benzoate	$C_{19}H_{23}NO_4$	329.4
[1-(pyren-1-ylmethyl)-piperidin-3-yl]methanol	$C_{23}H_{23}NO$	329.4
N-benzyl-*N*-(2-hydroxyethyl)-5,6,7,8-tetrahydro-4*H*-cyclohepta[b]-thiophene-2-carboxamide	$C_{19}H_{23}NO_2S$	329.5
2-ethoxy-*N*-(1-hydroxybutan-2-yl)-4-methyl-5-propan-2-ylbenzenesulfonamide	$C_{16}H_{27}NO_4S$	329.5
4-chloro-*N*,*N*-bis(2-hydroxyethyl)-naphthalene-1-sulfonamide	$C_{14}H_{16}ClNO_4S$	329.8
[2-[2-[2-(4-methylpiperidin-1-yl)-ethoxy]-ethoxy]-phenyl]-methanol; hydrochloride	$C_{17}H_{28}ClNO_3$	329.9
3-[4-(4-methoxyphenyl)-sulfonylpiperazin-1-yl]-propane-1,2-diol	$C_{14}H_{22}N_2O_5S$	330.1
2-[[5-(3-chloro-2-methylphenyl)-furan-2-yl]-methylamino]-butan-1-ol; hydrochloride	$C_{16}H_{21}Cl_2NO_2$	330.2
3-[[2-(2,4-difluoroanilino)-quinazolin-4-yl]-amino]-propan-1-ol	$C_{17}H_{16}F_2N_4O$	330.3

5-(dimethylsulfamoyl)-*N*-(1-hydroxy-2-methylpropan-2-yl)-2-methoxybenzamide	$C_{14}H_{22}N_2O_5S$	330.4
1-(4,5-dimethoxy-2-methylphenyl)-3-[[2-(hydroxymethyl)-phenyl]-methyl]-urea	$C_{18}H_{22}N_2O_4$	330.4
1-[[2-(hydroxymethyl)phenyl]methyl]-3-[3-(2-methoxyethoxy)-phenyl]-urea	$C_{18}H_{22}N_2O_4$	330.4
17-(2-hydroxyacetyl)-10,13-dimethyl-1,2,6,7,8,9,11,12,14,15,16,17-dodecahydrocyclopenta[a]-phenanthren-3-one	$C_{21}H_{30}O_3$	330.5
2-[1-[3-(2-chlorophenoxy)-propyl]-benzimidazol-2-yl]-ethanol	$C_{18}H_{19}ClN_2O_2$	330.8
3-[1-[2-(4-chlorophenoxy)-ethyl]-benzimidazol-2-yl]-propan-1-ol	$C_{18}H_{19}ClN_2O_2$	330.8
2-[5-chloro-2-ethoxy-4-[(1-hydroxybutan-2-ylamino)-methyl]-phenoxy]-acetamide	$C_{15}H_{23}ClN_2O_4$	330.8
2-(2-bromophenyl)-imino-5,5-bis(hydroxymethyl)-1,3-thiazolidin-4-one	$C_{11}H_{11}BrN_2O_3S$	331.2
3-(3-hydroxypropyl)-2-[(3-methyl-1,2-oxazol-5-yl)-methylsulfanyl]-quinazolin-4-one	$C_{16}H_{17}N_3O_3S$	331.4
2-(6-hydroxy-3-oxo-3*H*-xanthen-9-yl)-benzoic acid	$C_{20}H_{12}O_5$	332.3
2-[4-(3-ethoxy-4-fluorophenyl)-sulfonylpiperazin-1-yl]-ethanol	$C_{14}H_{21}FN_2O_4S$	332.4
1-[2-(2-hydroxyethyl)-5-(2-methylphenyl)-pyrazol-3-yl]-3-(1-methoxypropan-2-yl)-urea	$C_{17}H_{24}N_4O_3$	332.4
3-[2-(2,3-dihydro-1-benzofuran-5-yl)ethyl]-1-[4-(hydroxymethyl)-cyclohexyl]-1-methylurea	$C_{19}H_{28}N_2O_3$	332.4
5-(2-bromoethyl)-1-[4-hydroxy-5-(hydroxymethyl)-oxolan-2-yl]-pyrimidine-2,4-dione	$C_{11}H_{13}BrN_2O_5$	333.1
[3,5-dichloro-4-(naphthalen-1-ylmethoxy)-phenyl]-methanol	$C_{18}H_{14}Cl_2O_2$	333.2
2,3-dihydroxybutanedioic acid; 4-[1-hydroxy-2-(methylamino)-ethyl]-benzene-1,2-diol	$C_{13}H_{19}NO_9$	333.3
2-[5-[1-[(5-*tert*-butyl-1,2,4-oxadiazol-3-yl)-methyl]-piperidin-4-yl]-pyrazol-1-yl]-ethanol	$C_{17}H_{27}N_5O_2$	333.4
2-[4-fluoro-2,6-bis(hydroxymethyl)-phenoxy]-*N*-(2-phenylethyl)-acetamide	$C_{18}H_{20}FNO_4$	333.4
2-[4-[[(6-chloroimidazo[1,2-a]pyridin-2-yl)-methylamino]-methyl]-3,5-dimethylpyrazol-1-yl]-ethanol	$C_{16}H_{20}ClN_5O$	333.8
2-[[5-(3-chloro-4-fluorophenyl)-furan-2-yl]-methylamino]-2-methylpropan-1-ol; hydrochloride	$C_{15}H_{18}Cl_2FNO_2$	334.2
N-(3-hydroxypropyl)-1,3-dioxobenzo[de]-isoquinoline-5-sulfonamide	$C_{15}H_{14}N_2O_5S$	334.3
1-(2-fluorophenyl)-3-[2-hydroxyethyl(thiophen-3-ylmethyl)-amino]-pyrrolidin-2-one	$C_{17}H_{19}FN_2O_2S$	334.4
2-[4-[(1E)-1-hydroxyimino-2,3-dihydroinden-5-yl]-3-pyridin-4-ylpyrazol-1-yl]-ethanol	$C_{19}H_{18}N_4O_2$	334.4
4-(diethylsulfamoyl)-*N*-(1-hydroxy-2-methylpropan-2-yl)thiophene-2-carboxamide	$C_{13}H_{22}N_2O_4S_2$	334.5
2-hydroxy-1-(3-hydroxy-10,13-dimethyl-2,3,4,5,6,7,8,9,11,12,14,15,16,17-tetradecahydro-1*H*-cyclopenta[a]-phenanthren-17-yl)-ethanone	$C_{21}H_{34}O_3$	334.5
2-[4-(5-chloro-2-methoxyphenyl)-sulfonylpiperazin-1-yl]-ethanol	$C_{13}H_{19}ClN_2O_4S$	334.8

1-(3-bromophenyl)-3-[[2-(hydroxymethyl)-phenyl]-methyl]-urea	$C_{15}H_{15}BrN_2O_2$	335.2
2-(4-chlorophenoxy)-1-[4-(2-hydroxyethyl)-piperazin-1-yl]-ethanone; hydrochloride	$C_{14}H_{20}Cl2N_2O_3$	335.2
2-[5-fluoro-2-[(3-thiophen-2-yl-1,2,4-oxadiazol-5-yl)-methylamino]-phenoxy]-ethanol	$C_{15}H_{14}FN_3O_3S$	335.4
4-[1-hydroxy-2-[(propan-2-yl)-amino)-butyl]-benzene-1,2-diol; methanesulfonic acid	$C_{14}H_{25}NO_6S$	335.4
2-[6-(cyclopentylamino)-purin-9-yl]-5-(hydroxymethyl)-oxolane-3,4-diol	$C_{15}H_{21}N_5O_4$	335.4
1-[4-(2-hydroxyethyl)-piperazin-1-yl]-3-methylpyrido[1,2-a]-benzimidazole-4-carbonitrile	$C_{19}H_{21}N_5O$	335.4
3-(4-benzyl-11,12-dimethyl-3,5,6,8,10-pentazatricyclo[7.3.0.0^{2,6}]-dodeca-1(9),2,4,7,11-pentaen-10-yl)-propan-1-ol	$C_{19}H_{21}N_5O$	335.4
4-[1-hydroxy-2-(4-phenylbutan-2-ylamino)-propyl]-phenol; hydrochloride	$C_{19}H_{26}ClNO_2$	335.9
2-[[4-[(4-methylphenyl)-methoxy]-phenyl]-methylamino]-butan-1-ol; hydrochloride	$C_{19}H_{26}ClNO_2$	335.9
4-bromo-*N*-(2-hydroxyethyl)-5-methyl-2-propan-2-ylbenzenesulfonamide	$C_{12}H_{18}BrNO_3S$	336.3
1-(4-chlorophenoxy)-3-[2-(hydroxymethyl)-piperidin-1-yl]-propan-2-ol; hydrochloride	$C_{15}H_{23}Cl_2NO_3$	336.3
5-bromo-*N*-(1-hydroxybutan-2-yl)-2,4-dimethylbenzenesulfonamide	$C_{12}H_{18}BrNO_3S$	336.3
3-(3,5-difluorophenyl)-1-[2-hydroxy-1-(4-methoxyphenyl)-ethyl]-1-methylurea	$C_{17}H_{18}F_2N_2O_3$	336.3
N,*N*-bis(2-hydroxyethyl)-2-oxo-1*H*-benzo[cd]-indole-6-sulfonamide	$C_{15}H_{16}N_2O_5S$	336.4
[3-(2-methylpropyl)-1,2,4-oxadiazol-5-yl]methyl 2-(2-hydroxyethylsulfanyl)-benzoate	$C_{16}H_{20}N_2O_4S$	336.4
2-hydroxyethyl 3-(2,4-dimethylphenyl)-1-phenylpyrazole-4-carboxylate	$C_{20}H_{20}N_2O_3$	336.4
N-(4-hydroxybutyl)-1,5-diphenyl-1,2,4-triazole-3-carboxamide	$C_{19}H_{20}N_4O_2$	336.4
3-[2-hydroxy-1-(1-methylpyrazol-4-yl)-ethyl]-1,1-diphenylurea	$C_{19}H_{20}N_4O_2$	336.4
4-[(2-chloro-4-fluorophenyl)-methylamino]-*N*-(1-hydroxypropan-2-yl)-benzamide	$C_{17}H_{18}ClFN_2O_2$	336.8
5-bromo-*N*-[3-(hydroxymethyl)-4-methoxyphenyl]-pyridine-2-carboxamide	$C_{14}H_{13}BrN_2O_3$	337.2
2-[3-[(4-amino-2-methylpyrimidin-5-yl)-methyl]-4-methyl-1,3-thiazol-3-ium-5-yl]-ethanol; chloride; hydrochloride	$C_{12}H_{18}Cl_2N_4OS$	337.3
4-[2-(*tert*-butylamino)-1-hydroxyethyl]-2-(hydroxymethyl)-phenol; sulfuric acid	$C_{13}H_{23}NO_7S$	337.4
N,*N*-bis(2-hydroxyethyl)-4-phenoxybenzenesulfonamide	$C_{16}H_{19}NO_5S$	337.4
4-[hydroxy(phenyl)-methylidene]-1-(3-hydroxypropyl)-5-phenylpyrrolidine-2,3-dione	$C_{20}H_{19}NO_4$	337.4
3-hydroxy-*N*-(2-hydroxy-3-phenoxypropyl)-naphthalene-2-carboxamide	$C_{20}H_{19}NO_4$	337.4
2-[3-(hydroxymethyl)-anilino]-*N*-(5-methyl-1,2-oxazol-3-yl)-2-phenylacetamide	$C_{19}H_{19}N_3O_3$	337.4

1-(furan-2-ylmethyl)-3-(2-hydroxy-1-phenylethyl)-1-pyridin-2-ylurea	$C_{19}H_{19}N_3O_3$	337.4
5-ethyl-2-(2-hydroxyethylamino)-6-methyl-3-(oxolan-2-ylmethyl)-thieno[2,3-d]-pyrimidin-4-one	$C_{16}H_{23}N_3O_3S$	337.4
4-[3-(hydroxymethyl)-quinolin-2-yl]-1-(1-methylpyrazol-4-yl)-piperazin-2-one	$C_{18}H_{19}N_5O_2$	337.4
1-(3,4-dihydro-1*H*-isoquinolin-2-yl)-3-[2-(hydroxymethyl)-benzimidazol-1-yl]-propan-2-ol	$C_{20}H_{23}N_3O_2$	337.4
2-[4-(5-methyl-3-phenylpyrazolo[1,5-a]pyrimidin-7-yl)-piperazin-1-yl]-ethanol	$C_{19}H_{23}N_5O$	337.4
N-ethyl-*N*-[1-(4-fluorophenyl)ethyl]-2-[4-(2-hydroxyethyl)-piperazin-1-yl]-acetamide	$C_{18}H_{28}FN_3O_2$	337.4
4-[1-hydroxy-2-(1-phenoxypropan-2-ylamino)-propyl]-phenol; hydrochloride	$C_{18}H_{24}ClNO_3$	337.8
2-(2-chloro-4-hydroxyphenyl)-*N*-[2-(4-fluorophenyl)-2-hydroxypropyl]-acetamide	$C_{17}H_{17}ClFNO_3$	337.8
2-[2-methoxy-4-[(2-phenylethylamino)-methyl]-phenoxy]-ethanol; hydrochloride	$C_{18}H_{24}ClNO_3$	337.8
5-bromo-*N*-(1-hydroxy-2-methylpropan-2-yl)-2-methoxybenzenesulfonamide	$C_{11}H_{16}BrNO_4S$	338.2
4-bromo-*N*,*N*-bis(2-hydroxyethyl)-3-methylbenzenesulfonamide	$C_{11}H_{16}BrNO_4S$	338.2
2-[4-[(5-bromopyrimidin-2-yl)-amino]-4,5,6,7-tetrahydroindazol-1-yl]-ethanol	$C_{13}H_{16}BrN_5O$	338.2
2-(3,4-dihydroxyphenyl)-3,5,7-trihydroxychromen-4-one; dihydrate	$C_{15}H_{14}O_9$	338.3
N-[2-(hydroxymethyl)-cyclohexyl]-1-oxo-3,4-dihydro-2*H*-isoquinoline-7-sulfonamide	$C_{16}H_{22}N_2O_4S$	338.4
4-[[4-(3-hydroxypropylamino)-quinazolin-2-yl]-amino]-benzoic acid	$C_{18}H_{18}N_4O_3$	338.4
2-[[2-(1,3-benzodioxol-5-ylmethylamino)-quinazolin-4-yl]-amino]-ethanol	$C_{18}H_{18}N_4O_3$	338.4
2-[[2-[2-(2-methoxyphenyl)-ethylamino]-quinazolin-4-yl]-amino]-ethanol	$C_{19}H_{22}N_4O_2$	338.4
[1-[3-(4-butan-2-ylphenoxy)-propyl]-benzimidazol-2-yl]-methanol	$C_{21}H_{26}N_2O_2$	338.4
2-(10-hydroxydecyl)-5,6-dimethoxy-3-methylcyclohexa-2,5-diene-1,4-dione	$C_{19}H_{30}O_5$	338.4
2-[(3-bromo-4-ethoxyphenyl)-methylamino]-butan-1-ol; hydrochloride	$C_{13}H_{21}BrClNO_2$	338.7
2-(2-chlorophenyl)-*N*-(1-hydroxy-3-methylbutan-2-yl)-4-methyl-1,3-thiazole-5-carboxamide	$C_{16}H_{19}ClN_2O_2S$	338.9
N-[4-(2-hydroxyethoxy)-phenyl]-2-(3-methyl-1,2,4-oxadiazol-5-yl)-benzamide	$C_{18}H_{17}N_3O_4$	339.3
[2-[2-(2-quinolin-8-yloxyethoxy)-ethoxy]-phenyl]-methanol	$C_{20}H_{21}NO_4$	339.4
2-cyclohexyl-8-(2-hydroxyethyl)-pyrido[4,3-b][1,6]-naphthyridine-1,9-dione	$C_{19}H_{21}N_3O_3$	339.4
2-[4-[[(2-methoxynaphthalen-1-yl)-methylamino]-methyl]-3,5-dimethylpyrazol-1-yl]-ethanol	$C_{20}H_{25}N_3O_2$	339.4
2-[3,5-dimethyl-4-[[[(1-methylimidazol-2-yl)-phenylmethyl]-amino]-methyl]-pyrazol-1-yl]-ethanol	$C_{19}H_{25}N_5O$	339.4
4-[3-(4-benzylpiperidin-1-yl)-1-hydroxy-2-methylpropyl]-phenol	$C_{22}H_{29}NO_2$	339.5

2-[2-chloro-4-(hydroxymethyl)-6-methoxyphenoxy]-*N*-(2-fluorophenyl)-acetamide	C16H15ClFNO4	339.7
(9-methyl-3-oxa-9-azatricyclo[3.3.1.0^{2,4}]-nonan-7-yl) 3-hydroxy-2-phenylpropanoate; hydrochloride	$C_{17}H_{22}ClNO_4$	339.8
2-[[1-[(2-thiophen-2-yl-1,3-oxazol-4-yl)-methyl]-benzimidazol-2-yl]-amino]-ethanol	$C_{17}H_{16}N_4O_2S$	340.4
3-(1-benzothiophen-2-ylmethylamino)-*N*-(2-hydroxyethyl)-2-methylbenzamide	$C_{19}H_{20}N_2O_2S$	340.4
1-[2-[2-hydroxy-3-[2-(hydroxymethyl)-benzimidazol-1-yl]-propoxy]-phenyl]-ethanone	$C_{19}H_{20}N_2O_4$	340.4
3-[1-[3-(hydroxymethyl)-phenyl]-ethylamino]-1-(2-methoxyphenyl)-pyrrolidin-2-one	$C_{20}H_{24}N_2O_3$	340.4
2-(hydroxymethyl)-*N*-[3-(2-methoxyphenyl)-phenyl]-2-methylpyrrolidine-1-carboxamide	$C_{20}H_{24}N_2O_3$	340.4
3-[[6-(benzylamino)-9-propan-2-ylpurin-2-yl]-amino]-propan-1-ol	$C_{18}H_{24}N_6O$	340.4
4-(7-hydroxyhept-2-enyl)-5-(3-hydroxyoct-1-enyl)-cyclopentane-1,3-diol	$C_{20}H_{36}O_4$	340.5
4-hydroxy-3-(7-hydroxyheptyl)-2-(3-hydroxyoct-1-enyl)-cyclopentan-1-one	$C_{20}H_{36}O_4$	340.5
4-hydroxy-2-(7-hydroxyheptyl)-3-(3-hydroxyoct-1-enyl)-cyclopentan-1-one	$C_{20}H_{36}O_4$	340.5
3-(3-chloro-2-methoxyphenyl)-1-(2-hydroxyethyl)-1-(thiophen-3-ylmethyl)-urea	$C_{15}H_{17}ClN_2O_3S$	340.8
[4-[[1-(3-chloro-4-fluorophenyl)-triazol-4-yl]-methyl]-5-methylmorpholin-2-yl]-methanol	$C_{15}H_{18}ClFN_4O_2$	340.8
3-(3-bromophenyl)-*N*-(1-hydroxy-2-methylpropan-2-yl)-4,5-dihydro-1,2-oxazole-5-carboxamide	$C_{14}H_{17}BrN_2O_3$	341.2
2-[2,3-dihydro-1*H*-inden-1-yl-[(3-thiophen-2-yl-1,2,4-oxadiazol-5-yl)-methyl]-amino]-ethanol	$C_{18}H_{19}N_3O_2S$	341.4
8-(2-hydroxyethyl)-2-(oxolan-2-ylmethyl)-pyrido[4,3-b][1,6]-naphthyridine-1,9-dione	$C_{18}H_{19}N_3O_4$	341.4
N-(3-hydroxypropyl)-2,4,6-tri(propan-2-yl)-benzenesulfonamide	$C_{18}H_{31}NO_3S$	341.5
5-(4-chlorophenyl)-11-(2-hydroxyethyl)-2,3,7,8,11-pentazatricyclo[7.4.0.0^{2,6}]-trideca-1(9),3,5,7,12-pentaen-10-one	$C_{16}H_{12}ClN_5O_2$	341.8
2-[4-[[(4-fluorophenyl)-methylamino]-methyl]-2-methoxyphenoxy]-ethanol; hydrochloride	$C_{17}H_{21}ClFNO_3$	341.8
2-[[2-[(2-fluorophenyl)-methoxy]-3-methoxyphenyl]-methylamino]-ethanol; hydrochloride	$C_{17}H_{21}ClFNO_3$	341.8
1-[6-[3-(dimethylamino)-propoxy]-2,2-dimethyl-3a,5,6,6a-tetrahydrofuro[2,3-d][1,3]-dioxol-5-yl]-ethane-1,2-diol; hydrochloride	$C_{14}H_{28}ClNO_6$	341.8
2-[4-[[1-(3,4-dichlorophenyl)-ethylamino]-methyl]-3,5-dimethylpyrazol-1-yl]-ethanol	$C_{16}H_{21}Cl_2N_3O$	342.3
N-[(3,4-dichlorophenyl)-methyl]-3-fluoro-*N*-(2-hydroxyethyl)-pyridine-4-carboxamide	$C_{15}H_{13}Cl_2FN_2O_2$	343.2
2-[(5-bromo-2-fluorophenyl)-methyl-[(5-methyl-1,2-oxazol-3-yl)methyl]-amino]-ethanol	$C_{14}H_{16}BrFN_2O_2$	343.2

3-(2,6-dichlorophenyl)-*N*-(1-hydroxy-2-methylpropan-2-yl)-5-methyl-1,2-oxazole-4-carboxamide	$C_{15}H_{16}Cl_2N_2O_3$	343.2
1-(3,5-dichloro-4-methylphenyl)-3-[2-hydroxy-1-(1-methylpyrazol-4-yl)-ethyl]-urea	$C_{14}H_{16}Cl_2N_4O_2$	343.2
7-fluoro-*N*-(1-hydroxy-3-phenylpropan-2-yl)-2-oxo-3,4-dihydro-1*H*-quinoline-6-carboxamide	$C_{19}H_{19}FN_2O_3$	342.4
3-(2-ethyl-6-methylphenyl)-1-(2-hydroxyethyl)-1-[(2-methoxyphenyl)-methyl]-urea	$C_{20}H_{26}N_2O_3$	342.4
N-[2-[2-(2-hydroxyethyl)-piperidine-1-carbonyl]-phenyl]-furan-2-carboxamide	$C_{19}H_{22}N_2O_4$	342.4
1-[3-[[[1-(2-hydroxyethyl)-3,5-dimethylpyrazol-4-yl]-methylamino]-methyl]-phenyl]-pyrrolidin-2-one	$C_{19}H_{26}N_4O_2$	342.4
2-[(8-propyl-11-thia-9,14,16-triazatetracyclo[8.7.0.0^{2,7}.0^{12,17}]-heptadeca-1,7,9,12,14,16-hexaen-13-yl)-amino]-ethanol	$C_{18}H_{22}N_4OS$	342.5
N-(1-hydroxy-2-methylpropan-2-yl)-4-methyl-3-[methyl-(propyl)-sulfamoyl]-benzamide	$C_{16}H_{26}N_2O_4S$	342.5
2-[4-(2-ethoxy-4,5-dimethylphenyl)sulfonylpiperazin-1-yl]-ethanol	$C_{16}H_{26}N_2O_4S$	342.5
2-[3,5-dimethyl-4-[[[1-(1-phenylethyl)-pyrrolidin-3-yl]amino]-methyl]-pyrazol-1-yl]-ethanol	$C_{20}H_{30}N_4O$	342.5
4-(7-hydroxyheptyl)-5-(3-hydroxyoct-1-enyl)cyclopentane-1,3-diol	$C_{20}H_{38}O_4$	342.5
2-(6-anilinopurin-9-yl)-5-(hydroxymethyl)-oxolane-3,4-diol	$C_{16}H_{17}N_5O_4$	343.3
2-[5-fluoro-2-[[3-(2-methylphenyl)-1,2,4-oxadiazol-5-yl]methylamino]-phenoxy]-ethanol	$C_{18}H_{18}FN_3O_3$	343.4
2-(2-hydroxyethylamino)-1-[5-(4-methylphenyl)-3-thiophen-2-yl-3,4-dihydropyrazol-2-yl]ethanone	$C_{18}H_{21}N_3O_2S$	343.4
1-(4-fluoro-3-pyrrol-1-ylphenyl)-3-[2-hydroxy-1-(1-methylpyrazol-4-yl)-ethyl]-urea	$C_{17}H_{18}FN_5O_2$	343.4
2-[4-[(6-ethylthieno[2,3-d]pyrimidin-4-yl)-amino]-4,5,6,7-tetrahydroindazol-1-yl]-ethanol	$C_{17}H_{21}N_5OS$	343.4
1-[3-[[[2-hydroxy-1-(4-methoxyphenyl)ethyl]-methylamino]-methyl]-4-methoxyphenyl]-ethanone	$C_{20}H_{25}NO_4$	343.4
1-cyclohexyl-1-(2-hydroxyethyl)-3-[2-methyl-5-(1,3-oxazol-2-yl)-phenyl]-urea	$C_{19}H_{25}N_3O_3$	343.4
[5-(4-chlorophenyl)-1,3-oxazol-2-yl]methyl 4-(hydroxymethyl)-benzoate	$C_{18}H_{14}ClNO_4$	343.8
4-bromo-*N*-(3-hydroxypropyl)-naphthalene-1-sulfonamide	$C_{13}H_{14}BrNO_3S$	344.2
2-[(6-bromo-4-phenylquinazolin-2-yl)-amino]-ethanol	$C_{16}H_{14}BrN_3O$	344.2
[2-(2-methoxyanilino)-2-oxoethyl] 2-(2-hydroxyethylamino)-benzoate	$C_{18}H_{20}N_2O_5$	344.4
[4-(4-fluorophenoxy)-phenyl]-[4-(2-hydroxyethyl)-piperazin-1-yl]-methanone	$C_{19}H_{21}FN_2O_3$	344.4
1-(3-cyclopentyloxy-2-methylphenyl)-3-[1-(furan-2-yl)-2-hydroxyethyl]-urea	$C_{19}H_{24}N_2O_4$	344.4
4-[[1-hydroxy-3-(1*H*-indol-3-yl)propan-2-yl]-amino]-*N*,*N*-dimethylpiperidine-1-carboxamide	$C_{19}H_{28}N_4O_2$	344.5
2-[[2-(3-chloro-4-methoxyanilino)-quinazolin-4-yl]-amino]-ethanol	$C_{17}H_{17}ClN_4O_2$	344.8

N-[4-chloro-3-[5-(hydroxymethyl)-furan-2-yl]-phenyl]-4-fluorobenzamide	$C_{18}H_{13}ClFNO_3$	345.7
1-(2-hydroxyethyl)-1-[(2-methoxyphenyl)methyl]-3-(3-methylsulfanylphenyl)-urea	$C_{18}H_{22}N_2O_3S$	346.1
[2-bromo-6-[(1-methylbenzimidazol-2-yl)-methylamino]-phenyl]-methanol	$C_{16}H_{16}BrN_3O$	346.2
2-[[5-(3-chloro-4-methoxyphenyl)-furan-2-yl]methylamino]-butan-1-ol; hydrochloride	$C_{16}H_{21}Cl_2NO_3$	346.2
3,4,8,10-tetrahydroxy-2-(hydroxymethyl)-9-methoxy-3,4,4a,10b-tetrahydro-2*H*-pyrano[3,2-c]-isochromen-6-one; hydrate	$C_{14}H_{18}O_{10}$	346.3
N-(1,1-dioxothiolan-3-yl)-2-[4-fluoro-2-(2-hydroxyethoxy)-anilino]-acetamide	$C_{14}H_{19}FN_2O_5S$	346.4
2-[4-[[phenyl(pyridin-2-yl)-methyl]-amino]-pyrazolo[3,4-d]-pyrimidin-1-yl]-ethanol	$C_{19}H_{18}N_6O$	346.4
4-[[(1-benzylpyrazol-4-yl)-methyl-(2-hydroxyethyl)-amino]-methyl]-benzonitrile	$C_{21}H_{22}N_4O$	346.4
2-(3-methoxyphenyl)-2-[(2-thiophen-3-yl-1,3-thiazol-5-yl)-methylamino]-ethanol	$C_{17}H_{18}N_2O_2S_2$	346.5
11-hydroxy-17-(2-hydroxyacetyl)-10,13-dimethyl-1,2,6,7,8,9,11,12,14,15,16,17-dodecahydrocyclopenta[a]-phenanthren-3-one	$C_{21}H_{30}O_4$	346.5
17-hydroxy-17-(2-hydroxyacetyl)-10,13-dimethyl-2,6,7,8,9,11,12,14,15,16-decahydro-1H-cyclopenta[a]-phenanthren-3-one	$C_{21}H_{30}O_4$	346.5
N-benzyl-1-(2,2-dimethylpropanoyl)-*N*-(2-hydroxyethyl)-piperidine-4-carboxamide	$C_{20}H_{30}N_2O_3$	346.5
2-[[5-(4-bromo-3-methylphenyl)-furan-2-yl]-methylamino]-ethanol; hydrochloride	$C_{14}H_{17}BrClNO_2$	346.7
2-[4-(2-hydroxyethyl)-phenoxy]-*N*-(2-phenylphenyl)-acetamide	$C_{22}H_{21}NO_3$	347.4
1-(4-ethoxyphenyl)-3-[4-(2-hydroxyethyl)-piperazin-1-yl]-pyrrolidine-2,5-dione	$C_{18}H_{25}N_3O_4$	347.4
N-(1-acetyl-2,3-dihydroindol-5-yl)-2-[(1-hydroxy-3,3-dimethylbutan-2-yl)-methylamino]-acetamide	$C_{19}H_{29}N_3O_3$	347.5
3-(4-chlorophenyl)-6-(2-hydroxyethyl)-2-methyl-7-oxo-1*H*-pyrazolo[1,5-a]-pyrimidine-5-carboxylic acid	$C_{16}H_{14}ClN_3O_4$	347.8
1-(4-chlorophenyl)-2-[4-[(4-fluorophenyl)-methyl]-piperidin-1-yl]-ethanol	$C_{20}H_{23}ClFNO$	347.9
2-[[2-bromo-4-(hydroxymethyl)-6-methoxyphenoxy]-methyl]-benzonitrile	$C_{16}H_{14}BrNO_3$	348.2
6-(2,4-dinitrophenoxy)-5-fluoro-2-(hydroxymethyl)-oxane-3,4-diol	$C_{12}H_{13}FN_2O_9$	348.2
2-[[2-[(4-fluorophenyl)methyl]-3-hydroxypropyl]-amino]-7-methyl-[1,3,4]-thiadiazolo[3,2-a]-pyrimidin-5-one	$C_{16}H_{17}FN_4O_2S$	348.4
4-[(1-hydroxy-2-methylpropan-2-yl)-sulfamoyl]-*N*-phenylbenzamide	$C_{17}H_{20}N_2O_4S$	348.4
4-(benzenesulfonamido)-*N*-(1-hydroxybutan-2-yl)-benzamide	$C_{17}H_{20}N_2O_4S$	348.4
N-(2-cyclopentylpyrazol-3-yl)-2-[2-hydroxyethyl(thiophen-3-ylmethyl)-amino]-acetamide	$C_{17}H_{24}N_4O_2S$	348.5
2-hydroxy-6-pentadecyl-benzoic acid	$C_{22}H_{36}O_3$	348.5

2,6-ditert-butyl-4-[[4-(2-hydroxyethyl)-piperazin-1-yl]-methyl]-phenol	$C_{21}H_{36}N_2O_2$	348.5
4-[2-(3-chloroanilino)-2-oxoethoxy]-*N*-(2-hydroxyethyl)-benzamide	$C_{17}H_{17}ClN_2O_4$	348.8
N-[3-[(2-bromo-4-methylphenyl)-methylamino]-phenyl]-2-hydroxyacetamide	$C_{16}H_{17}BrN_2O_2$	349.2
[3-(hydroxymethyl)-piperidin-1-yl]-[4-(2-(4-methoxyphenyl)-ethynyl)-phenyl]-methanone	$C_{22}H_{23}NO_3$	349.4
2-[6-(czclohexylamino)-purin-9-yl]-5-(hydroxymethyl)-oxolane-3,4-diol	$C_{16}H_{23}N_5O_4$	349.4
2-[6-[(2-hydroxycyclopentyl)-amino]-purin-9-yl]-5-(hydroxymethyl)-oxolane-3,4-diol	$C_{15}H_{21}N_5O_5$	351.4
4-ethylidene-7-hydroxy-7-(hydroxymethyl)-6-methyl-2,9-dioxa-14-azatricyclo[9.5.1.0^{14,17}]-heptadec-11-ene-3,8-dione	$C_{18}H_{25}NO_6$	351.4
2-[2-(3-hydroxypropyl)-benzimidazol-1-yl]-*N*-phenyl-*N*-propan-2-ylacetamide	$C_{21}H_{25}N_3O_2$	351.4
5,6-dihydrobenzo[b][1]-benzazepin-11-yl-[4-(2-hydroxyethyl)-piperazin-1-yl]-methanone	$C_{21}H_{25}N_3O_2$	351.4
3-[2-[(3-bromo-4-methoxyphenyl)-methylamino]-1-hydroxyethyl]-phenol	$C_{16}H_{18}BrNO_3$	352.2
5-bromo-2-ethoxy-*N*-(1-hydroxybutan-2-yl)-benzenesulfonamide	$C_{12}H_{18}BrNO_4S$	352.3
2-[(1-benzyl-4-hydroxy-2-oxoquinoline-3-carbonyl)-amino]-acetic acid	$C_{19}H_{16}N_2O_5$	352.3
2-[[5-(dimethylamino)-naphthalen-1-yl]-sulfonylamino]-4-hydroxybutanoic acid	$C_{16}H_{20}N_2O_5S$	352.4
N-(2-hydroxyethyl)-1-(4-methoxyphenyl)-*N*-methyl-5-phenyl-1,2,4-triazole-3-carboxamide	$C_{19}H_{20}N_4O_3$	352.4
3-[[2-(2,3-dihydro-1,4-benzodioxin-6-ylamino)-quinazolin-4-yl]amino]-propan-1-ol	$C_{19}H_{20}N_4O_3$	352.4
2-[4-(4-cyclohexylphenyl)-sulfonylpiperazin-1-yl]-ethanol	$C_{18}H_{28}N_2O_3S$	352.5
[1-[4-(5-methyl-2-propan-2-ylphenoxy)-butyl]-benzimidazol-2-yl]methanol	$C_{22}H_{28}N_2O_2$	352.5
1-(4-bromophenyl)-*N*-(2-hydroxyethyl)-5-propan-2-yltriazole-4-carboxamide	$C_{14}H_{17}BrN_4O_2$	353.2
[2-fluoro-4-[2-(4-methoxyphenyl)-ethynyl]-phenyl]-(3-hydroxypiperidin-1-yl)-methanone	$C_{21}H_{20}FNO_3$	353.4
1-(2-hydroxyethyl)-4-[hydroxy(phenyl)-methylidene]-5-(4-methoxyphenyl)-pyrrolidine-2,3-dione	$C_{20}H_{19}NO_5$	353.4
[2-oxo-2-(1,2,3,4-tetrahydronaphthalen-1-ylmethylamino)-ethyl] 4-(hydroxymethyl)-benzoate	$C_{21}H_{23}NO_4$	353.4
1-[4-hydroxy-5-(hydroxymethyl)-oxolan-2-yl]-5-iodopyrimidine-2,4-dione	$C_9H_{11}IN_2O_5$	354.1
3-acetyl-2-(4-bromophenyl)-4-hydroxy-1-(3-hydroxypropyl)-2H-pyrrol-5-one	$C_{15}H_{16}BrNO_4$	354.2
3-[3-(3,4-dihydroxyphenyl)-prop-2-enoyloxy]-1,4,5-trihydroxycyclohexane-1-carboxylic acid	$C_{16}H_{18}O_9$	354.3
4-[(1-hydroxy-2-methylpropan-2-yl)-sulfamoyl]-*N*-phenylthiophene-2-carboxamide	$C_{15}H_{18}N_2O_4S_2$	354.4
2-[[6-(benzylamino)-9-propan-2-ylpurin-2-yl]-amino]-butan-1-ol	$C_{19}H_{26}N_6O$	354.4

2-[5-bromo-2-methoxy-4-[(propan-2-ylamino)-methyl]-phenoxy]-ethanol; hydrochloride	$C_{13}H_{21}BrClNO_3$	354.7
(5-bromo-2,3,4-trimethylphenyl)-[4-(2-hydroxyethyl)-piperazin-1-yl]-methanone	$C_{16}H_{23}BrN_2O_2$	355.3
1-benzyl-3-(3-cyano-4,5,6,7-tetrahydro-1-benzothiophen-2-yl)-1-(2-hydroxyethyl)-urea	$C_{19}H_{21}N_3O_2S$	355.5
3-[[4-[2-(3-chloroanilino)-pyrimidin-4-yl]-pyridin-2-yl]-amino]-propan-1-ol	$C_{18}H_{18}ClN_5O$	355.8
2-bromo-*N*-(2-hydroxyethyl)-*N*-phenylbenzenesulfonamide	$C_{14}H_{14}BrNO_3S$	356.2
2-[2-(4-bromophenyl)-imidazo[1,2-a]-benzimidazol-3-yl]-ethanol	$C_{17}H_{14}BrN_3O$	356.2
2,2-dichloro-*N*-[1,3-dihydroxy-1-(4-methylsulfonylphenyl)-propan-2-yl]-acetamide	$C_{12}H_{15}Cl_2NO_5S$	356.2
N-(3,4-dichlorophenyl)-2-[4-(hydroxymethyl)-2-methoxyphenoxy]-acetamide	$C_{16}H_{15}Cl_2NO_4$	356.2
2-(2-hydroxyethylsulfanylmethyl)-spiro[3,6-dihydrobenzo[h]-quinazoline-5,1’-cyclohexane]-4-one	$C_{20}H_{24}N_2O_2S$	356.2
1-[2-(2-hydroxyethylamino)-benzimidazol-1-yl]-3,3-dimethylbutan-2-one; hydrobromide	$C_{15}H_{22}BrN_3O_2$	356.3
2-[4-[3-(benzylamino)-4-nitrophenyl]-piperazin-1-yl]-ethanol	$C_{19}H_{24}N_4O_3$	356.4
1-[2-(diethylamino)ethylamino]-4-(hydroxymethyl)-thioxanthen-9-one	$C_{20}H_{24}N_2O_2S$	356.5
3-amino-*N*-[3-(2-hydroxyethylsulfonyl)-phenyl]-benzamide; hydrochloride	$C_{15}H_{17}ClN_2O_4S$	356.8
1-benzyl-*N*-[3-(hydroxymethyl)-phenyl]-*N*-methylpyrazole-4-sulfonamide	$C_{18}H_{19}N_3O_3S$	357.1
2-[[3-methoxy-4-(thiophen-2-ylmethoxy)-phenyl]-methylamino]-2-methylpropan-1-ol; hydrochloride	$C_{17}H_{24}ClNO_3S$	357.9
(*Z*)-2-cyano-*N*-(2,5-dibromophenyl)-3-hydroxybut-2-enamide	$C_{11}H_8Br_2N_2O_2$	357.9
2-[[4-[(3-chlorophenyl)-methoxy]-3-methoxyphenyl]-methylamino]-ethanol; hydrochloride	$C_{17}H_{21}Cl_2NO_3$	358.3
7-hydroxy-6-[3,4,5-trihydroxy-6-(hydroxymethyl)-oxan-2-yl]-oxychromen-2-one; hydrate	$C_{15}H_{18}O_{10}$	358.3
2,3,5,6-tetrahydroxy-4-[3,4,5-trihydroxy-6-(hydroxymethyl)-oxan-2-yl]-oxyhexanoic acid	$C_{12}H_{22}O_{12}$	358.3
2-(6-amino-2-anilinopurin-9-yl)-5-(hydroxymethyl)-oxolane-3,4-diol	$C_{16}H_{18}N_6O_4$	358.4
17-hydroxy-17-(2-hydroxyacetyl)-10,13-dimethyl-6,7,8,9,12,14,15,16-octahydrocyclopenta[a]-phenanthrene-3,11-dione	$C_{21}H_{26}O_5$	358.4
17-hydroxy-17-(2-hydroxyacetyl)-10,13-dimethyl-1,2,6,7,8,9,12,14,15,16-decahydrocyclopenta[a]-phenanthrene-3,11-dione	$C_{21}H_{28}O_5$	360.4
11,17-dihydroxy-17-(2-hydroxyacetyl)-10,13-dimethyl-7,8,9,11,12,14,15,16-octahydro-6*H*-cyclopenta[a]-phenanthren-3-one	$C_{21}H_{28}O_5$	360.4
11-hydroxy-17-(2-hydroxyacetyl)-10-methyl-3-oxo-1,2,6,7,8,9,11,12,14,15,16,17-dodecahydrocyclopenta[a]-phenanthrene-13-carbaldehyde	$C_{21}H_{28}O_5$	360.4
18-hydroxy-2-(2-hydroxyacetyl)-14-methyl-17-oxapentacyclo[14.2.1.0^{1,5}.0^{6,15}0^{9,14}]-nonadec-9-en-11-one	$C_{21}H_{28}O_5$	360.4
[1-[3-(4-bromophenoxy)-propyl]-benzimidazol-2-yl]-methanol	$C_{17}H_{17}BrN_2O_2$	361.2

(2*S*, 3*S*)-2,3-dihydroxybutanedioic acid; 4-[(1*R*)1-hydroxy-2-(propan-2-yl)-amino]-ethyl]-benzene-1,2-diol	$C_{15}H_{23}NO_9$	361.3
[2-[[[5-(4-methylphenyl)-thieno[2,3-d]-pyrimidin-4-yl]-amino]-methyl]-phenyl]-methanol	$C_{21}H_{19}N_3OS$	361.5
6-[6-(3-hydroxyundeca-1,5-dienyl)-pyridin-2-yl]-hexane-1,5-diol	$C_{22}H_{35}NO_3$	361.5
[3-bromo-4-[(2,4-dichlorophenyl)-methoxy]-phenyl]-methanol	$C_{14}H_{11}BrCl_2O_2$	362.0
[4-[(4-bromophenyl)-methoxy]-3,5-dichlorophenyl]-methanol	$C_{14}H_{11}BrCl_2O_2$	362.0
2-[[5-bromo-2-ethoxy-4-(hydroxymethyl)-phenoxy]-methyl]-benzonitrile	$C_{17}H_{16}BrNO_3$	362.2
2-[4-bromo-2-(hydroxymethyl)-phenoxy]-1-(2,3-dihydroindol-1-yl)-ethanone	$C_{17}H_{16}BrNO_3$	362.2
2-[4-[6-(4-bromophenyl)-pyridazin-3-yl]-piperazin-1-yl]-ethanol	$C_{16}H_{19}BrN_4O$	362.3
7-[(4-fluorophenyl)-methoxy]-5-hydroxy-2-phenylchromen-4-one	$C_{22}H_{15}FO_4$	362.3
2-[(2,3-difluorophenyl)-methylsulfanyl]-3-(3-hydroxypropyl)-quinazolin-4-one	$C_{18}H_{16}F_2N_2O_2S$	362.4
N-(1-hydroxy-2-methylpropan-2-yl)-3-[(4-methylphenyl)-sulfonylamino]-benzamide	$C_{18}H_{22}N_2O_4S$	362.4
2,3-bis[(4-hydroxy-3-methoxyphenyl)-methyl]-butane-1,4-diol	$C_{20}H_{26}O_6$	362.4
11,17-dihydroxy-17-(2-hydroxyacetyl)-10,13-dimethyl-2,6,7,8,9,11,12,14,15,16-decahydro-1*H*-cyclopenta[a]-phenanthren-3-one	$C_{21}H_{30}O_5$	362.5
2-[4-(4-chloro-3-propoxyphenyl)-sulfonylpiperazin-1-yl]-ethanol	$C_{15}H_{23}ClN_2O_4S$	362.9
8-(2-hydroxyethyl)-2-(3-methoxyphenyl)-pyrido[4,3-b][1,6]-naphthyridine-1,9-dione	$C_{20}H_{17}N_3O_4$	363.4
ethyl 6-[[2-hydroxyethyl(methyl)-carbamoyl]-amino]-4-thia-2-azatricyclo[7.3.0.0^{3,7}]-dodeca-1,3(7),5,8-tetraene-5-carboxylate	$C_{17}H_{21}N_3O_4S$	363.4
4*a*,9-dihydroxy-3-prop-2-enyl-2,4,5,6,7*a*,13-hexahydro-1*H*-4,12-methanobenzofuro[3,2-e]-isoquinolin-7-one; hydrochloride	$C_{19}H_{22}ClNO_4$	363.8
2-[[4-[(2-chlorophenyl)-methoxy]-3-ethoxyphenyl]-methylamino]-butan-1-ol	$C_{20}H_{26}ClNO_3$	363.9
1-[3-[[4-[4-(hydroxymethyl)-triazol-1-yl]-anilino]-methyl]-2,4,6-trimethylphenyl]-ethanone	$C_{21}H_{24}N_4O_2$	364.4
2-hydroxy-5-[1-hydroxy-2-(4-phenylbutan-2-ylamino)-ethyl]-benzamide; hydrochloride	$C_{19}H_{25}ClN_2O_3$	364.9
2-hydroxy-4-[[2-(4-methylphenyl)-sulfonyloxyacetyl]-amino]-benzoic acid	$C_{16}H_{15}NO_7S$	365.4
2-[[4-(1,3-benzodioxol-5-ylmethoxy)-phenyl]-methylamino]-2-methylpropan-1-ol; hydrochloride	$C_{19}H_{24}ClNO_4$	365.8
5-bromo-2-hydroxy-*N*-[2-hydroxy-2-(4-methoxyphenyl)-ethyl]benzamide	$C_{16}H_{16}BrNO_4$	366.2
4,7-dichloro-3-hydroxy-3-[2-(4-methoxyphenyl)-2-oxoethyl]-1*H*-indol-2-one	$C_{17}H_{13}Cl_2NO_4$	366.2
(8,8-dimethyl-8-azoniabicyclo[3.2.1]octan-3-yl) 3-hydroxy-2-phenylpropanoate; nitrate	$C_{18}H_{26}N_2O_6$	366.4
ethyl 5-chloro-3-[[4-(2-hydroxyethyl)-piperazin-1-yl]-methyl]-1-benzofuran-2-carboxylate	$C_{18}H_{23}ClN_2O_4$	366.8

N-[2-[(2-fluorophenyl)-methyl]-3-hydroxypropyl]-5-methyl-1-phenylpyrazole-3-carboxamide	$C_{21}H_{22}FN_3O_2$	367.2
4-[hydroxy(phenyl)-methylidene]-1-(3-hydroxypropyl)-5-(4-methoxyphenyl)-pyrrolidine-2,3-dione	$C_{21}H_{21}NO_5$	367.4
1-[2-(hydroxymethyl)-1,3-dihydroinden-2-yl]-3-[3-(2-oxo-1,3-oxazolidin-3-yl)-phenyl]-urea	$C_{20}H_{21}N_3O_4$	367.4
2-[(12-ethyl-12-methyl-5-propylsulfanyl-11-oxa-8-thia-4,6-diazatricyclo[7.4.0.0^{2,7}]-trideca-1(9),2,4,6-tetraen-3-yl)-amino]-ethanol	$C_{17}H_{25}N_3O_2S_2$	367.5
4-[4-(4-fluorophenyl)-5-(2-methoxypyrimidin-4-yl)-imidazol-1-yl]-cyclohexan-1-ol	$C_{20}H_{21}FN_4O_2$	368.4
2-(2,3-dihydro-1,4-benzodioxin-6-yl)-*N*-[[2-(hydroxymethyl)-phenyl]-methyl]-pyrrolidine-1-carboxamide	$C_{21}H_{24}N_2O_4$	368.4
7-[2-(3,8-dihydroxyoct-1-enyl)-3-hydroxy-5-oxocyclopentyl]-hept-5-enoic acid	$C_{20}H_{32}O_6$	368.5
2-[[1-[[3-(4-chlorophenyl)-1,2-oxazol-5-yl]-methyl]-benzimidazol-2-yl]-amino]-ethanol	$C_{19}H_{17}ClN_4O_2$	368.8
2-[[4-[(2-fluorophenyl)-methoxy]-3-methoxyphenyl]-methylamino]-2-methylpropan-1-ol; hydrochloride	$C_{19}H_{25}ClFNO_3$	369.2
3-hydroxy-2-(octadec-9-enoylamino)-propanoic acid	$C_{21}H_{39}NO_4$	369.5
2-[4-(2-hydroxyethoxy)-3,5-dimethylphenyl]-5,7-dimethoxy-3H-quinazolin-4-one	$C_{20}H_{22}N_2O_5$	370.4
7-(4-fluorophenyl)-2-[4-(2-hydroxyethyl)-piperazin-1-yl]-7,8-dihydro-6*H*-quinazolin-5-one	$C_{20}H_{23}FN_4O_2$	370.4
2-(hydroxymethyl)-5-[6-(2-phenylethylamino)-purin-9-yl]-oxolane-3,4-diol	$C_{18}H_{21}N_5O_4$	371.4
2-(hydroxymethyl)-5-[6-[(2-methylphenyl)-methylamino]-purin-9-yl]-oxolane-3,4-diol	$C_{18}H_{21}N_5O_4$	371.4
5-(4-chlorophenyl)-4-[hydroxy(phenyl)-methylidene]-1-(3-hydroxypropyl)-pyrrolidine-2,3-dione	$C_{20}H_{18}ClNO_4$	371.8
2-[4-[(4-chlorophenoxy)-methyl]-11,12-dimethyl-3,5,6,8,10-pentazatricyclo[7.3.0.0^{2,6}]-dodeca-1(9),2,4,7,11-pentaen-10-yl]-ethanol	$C_{18}H_{18}ClN_5O_2$	371.8
1-(3,4-dichlorophenyl)-3-[4-(2-hydroxyethyl)-piperazin-1-yl]-pyrrolidine-2,5-dione	$C_{16}H_{19}Cl_2N_3O_3$	372.2
3-[2-[2-(3,4-dimethylphenoxy)-ethylsulfanyl]-benzimidazol-1-yl]-propane-1,2-diol	$C_{20}H_{24}N_2O_3S$	372.5
[3-(4-chlorophenyl)-1,2-oxazol-5-yl]-methyl 2-(2-hydroxyethylamino)-benzoate	$C_{19}H_{17}ClN_2O_4$	372.8
2-[4-[1-[(2-chlorophenyl)-methyl]-pyrazolo[3,4-d]-pyrimidin-4-yl]-piperazin-1-yl]-ethanol	$C_{18}H_{21}ClN_6O$	372.9
2-(6-bromoindol-1-yl)-*N*-[4-(2-hydroxyethyl)-phenyl]-acetamide	$C_{18}H_{17}BrN_2O_2$	373.2
[4-[2-[2-(5-chloroquinolin-8-yl)-oxyethoxy]-ethoxy]-phenyl]-methanol	$C_{20}H_{20}ClNO_4$	373.8
11,17-dihydroxy-17-(2-hydroxyacetyl)-6,10,13-trimethyl-7,8,9,11,12,14,15,16-octahydro-6*H*-cyclopenta[a]-phenanthren-3-one	$C_{22}H_{30}O_5$	374.5
2-[2-(3-hydroxypropylamino)-benzimidazol-1-yl]-*N*-(4-methylphenyl)-acetamide; hydrochloride	$C_{19}H_{23}ClN_4O_2$	374.9

2-(3-hydroxypropylamino)-5,5-dimethyl-3-phenyl-6*H*-benzo[h]-quinazolin-4-one	$C_{23}H_{25}N_3O_2$	375.5
N-[[2-(hydroxymethyl)-phenyl]-methyl]-1-phenyl-5,6,7,8-tetrahydro-4*H*-cyclohepta[c]-pyrazole-3-carboxamide	$C_{23}H_{25}N_3O_2$	375.5
4-[(1*R*,2*S*)-3-(4-benzylpiperidin-1-yl)-1-hydroxy-2-methylpropyl]-phenol; hydrochloride	$C_{22}H_{30}ClNO_2$	375.9
7,8-dimethyl-10-[(2*S*,3*S*,4*R*)-2,3,4,5-tetrahydroxypentyl)-2H,3H,4H,10H-benzo[g]-pteridine-2,4-dione	$C_{17}H_{20}N_4O_6$	376.4
2-amino-2-(hydroxymethyl)-propane-1,3-diol; 5-benzoyl-2,3-dihydro-1*H*-pyrrolizine-1-carboxylic acid	$C_{19}H_{24}N_2O_6$	376.4
2-[*N*-(2-hydroxyethyl)-anilino]-*N*-(4-phenylmethoxyphenyl)-acetamide	$C_{23}H_{24}N_2O_3$	376.4
N-(3-hydroxypropyl)-1-naphthalen-2-ylsulfonylpiperidine-3-carboxamide	$C_{19}H_{24}N_2O_4S$	376.5
9-fluoro-11-hydroxy-17-(2-hydroxyacetyl)-10,13,16-trimethyl-7,8,11,12,14,15,16,17-octahydro-6*H*-cyclopenta[a]-phenanthren-3-one	$C_{22}H_{29}FO_4$	376.5
2-chloro-*N*-(1-hydroxy-2-methylpropan-2-yl)-5-morpholin-4-ylsulfonylbenzamide	$C_{15}H_{21}ClN_2O_5S$	376.6
2-[5-hydroxy-2-(3-hydroxypropyl)-cyclohexyl]-5-(2-methyloctan-2-yl)-phenol	$C_{24}H_{40}O_3$	376.6
8-(2-hydroxyethyl)-2-[(4-methoxyphenyl)-methyl]-pyrido[4,3-b][1,6]-naphthyridine-1,9-dione	$C_{21}H_{19}N_3O_4$	377.4
2-[(1,5-dimethyl-2-oxo-3*H*-indol-3-yl)-methyl]-3-(3-hydroxypropyl)-quinazolin-4-one	$C_{22}H_{23}N_3O_3$	377.4
1-[4-(2-hydroxyethyl)-piperazin-1-yl]-3-(2-phenylindol-1-yl)-propan-1-one	$C_{23}H_{27}N_3O_2$	377.5
3-(cyclopropylmethyl)-4*a*,9-dihydroxy-2,4,5,6,7*a*,13-hexahydro-1*H*-4,12-methanobenzofuro[3,2-e]-isoquinolin-7-one; hydrochloride	$C_{20}H_{24}ClNO_4$	377.9
5-bromo-*N*-[1-(4-chloro-3-fluorophenyl)-2-hydroxyethyl]thiophene-2-carboxamide	$C_{13}H_{10}BrClFNO_2S$	378.6
2-[2-[[(1-hydroxy-2-methylpropan-2-yl)-amino]-methyl]-phenoxy]-*N*-(2-methylphenyl)-acetamide; hydrochloride	$C_{20}H_{27}ClN_2O_3$	378.9
N-[(3,4-dimethoxyphenyl)-methyl]-4-(2-hydroxyethylsulfonyl)-benzamide	$C_{18}H_{21}NO_6S$	379.4
2-[4-(2-methyl-3-phenyl-5-propylpyrazolo[1,5-a]-pyrimidin-7-yl)-piperazin-1-yl]-ethanol	$C_{22}H_{29}N_5O$	379.5
(9,9-dimethyl-3-oxa-9-azoniatricyclo[3.3.1.0^{2,4}]-nonan-7-yl) 3-hydroxy-2-phenylpropanoate; nitrate	$C_{18}H_{24}N_2O_7$	380.4
[1-(4-chloro-2-fluoroanilino)-1-oxopropan-2-yl] 2-(2-hydroxyethylamino)-benzoate	$C_{18}H_{18}ClFN_2O_4$	380.8
1-(3,5-dimethylphenoxy)-3-[4-(2-hydroxyethyl)-piperazin-1-yl]-propan-2-ol; dihydrochloride	$C_{17}H_{30}Cl_2N_2O_3$	381.3
2-[[4-(2-hydroxy-2-phenylethoxy)-3-methoxyphenyl]-methylamino]-butan-1-ol; hydrochloride	$C_{20}H_{28}ClNO_4$	381.9
2-[(4-*tert*-butylphenyl)-methylsulfanyl]-3-(3-hydroxypropyl)-quinazolin-4-one	$C_{22}H_{26}N_2O_2S$	382.2
2-[2-(2-hydroxyethylamino)-benzimidazol-1-yl]-1-thiophen-2-ylethanone; hydrobromide	$C_{15}H_{16}BrN_3O_2S$	382.3

3-[4-(5,6-dimethyl-2-phenylthieno[2,3-d]-pyrimidin-4-yl)-piperazin-1-yl]-propan-1-ol	$C_{21}H_{26}N_4OS$	382.5
[2-oxo-2-(4-phenylpiperazin-1-yl)-ethyl] 2-(2-hydroxyethylamino)-benzoate	$C_{21}H_{25}N_3O_4$	383.4
5-(2-chlorophenyl)-4-(3-hydroxypropylamino)-5*H*-chromeno[2,3-d]-pyrimidin-8-ol	$C_{20}H_{18}ClN_3O_3$	383.8
[(1*R*,2*R*,4*S*,5*S*)-9-methyl-3-oxa-9-azatricyclo[3.3.1.0^{2,4}]-nonan-7-yl] (2*S*)-3-hydroxy-2-phenylpropanoate; hydrobromide	$C_{17}H_{22}BrNO_4$	384.3
5-[1-hydroxy-2-[1-(4-hydroxyphenyl)-propan-2-ylamino]-ethyl]-benzene-1,3-diol; hydrobromide	$C_{17}H_{22}BrNO_4$	384.3
(9-methyl-3-oxa-9-azatricyclo[3.3.1.0^{2,4}]-nonan-7-yl) 3-hydroxy-2-phenylpropanoate; hydrobromide	$C_{17}H_{22}BrNO_4$	384.3
(8,8-dimethyl-8-azoniabicyclo[3.2.1]-octan-3-yl) 3-hydroxy-2-phenylpropanoate; bromide	$C_{18}H_{26}BrNO_3$	384.3
2-[*N*-[[2-(4-ethoxy-3-methoxyphenyl)-1,3-thiazol-4-yl]-methyl]-anilino]-ethanol	$C_{21}H_{24}N_2O_3S$	384.5
2-[4-bromo-2-(hydroxymethyl)phenoxy]-N-(3-chloro-2-methylphenyl)acetamide	$C_{16}H_{15}BrClNO_3$	384.6
2-[2-(4-bromophenyl)-ethenyl]-3-(3-hydroxypropyl)-quinazolin-4-one	$C_{19}H_{17}BrN_2O_2$	385.3
4-[[4-(2-hydroxyethylsulfonyl)-phenyl]-iminomethyl]-5-methyl-2-phenyl-1*H*-pyrazol-3-one	$C_{19}H_{19}N_3O_4S$	385.4
2-(hydroxymethyl)-5-[6-(1-phenylpropan-2-ylamino)-purin-9-yl]-oxolane-3,4-diol	$C_{19}H_{23}N_5O_4$	385.4
N'-hydroxy-*N*-[1-[2-(hydroxymethyl)-pyrrolidin-1-yl]-3-methyl-1-oxobutan-2-yl]-2-pentylbutanediamide	$C_{19}H_{35}N_3O_5$	385.5
2-[(5-bromo-2-fluorophenyl)methyl-[(5-tert-butyl-1,2,4-oxadiazol-3-yl)-methyl]-amino]-ethanol	$C_{16}H_{21}BrFN_3O_2$	386.3
2-[6-[2-(4-aminophenyl)-ethylamino]-purin-9-yl]-5-(hydroxymethyl)-oxolane-3,4-diol	$C_{18}H_{22}N_6O_4$	386.4
4-hydroxy-2-(hydroxymethyl)-*N*-[3-[(4-methoxyphenyl)-methoxymethyl]-phenyl]-pyrrolidine-1-carboxamide	$C_{21}H_{26}N_2O_5$	386.4
1-[2-(benzhydrylideneamino)-oxyethyl]-3,6-dihydro-2*H*-pyridine-5-carboxylic acid; hydrochloride	$C_{21}H_{23}ClN_2O_3$	386.9
2-[4-[2-[(4-chlorophenyl)-methyl]-5-methyl-[1,2,4]-triazolo[1,5-a]-pyrimidin-7-yl]-piperazin-1-yl]-ethanol	$C_{19}H_{23}ClN_6O$	386.9
2-[4-[[[5-(2-fluorophenyl)-thiophen-2-yl]-methylamino]-methyl]-2-methoxyphenoxy]-ethanol	$C_{21}H_{22}FNO_3S$	387.5
N-cyclohexyl-*N*-(1,1-dioxothiolan-3-yl)-2-[4-(2-hydroxyethyl)-piperazin-1-yl]-acetamide	$C_{18}H_{33}N_3O_4S$	387.5
2-[2-chloro-6-(cyclopentylamino)-purin-9-yl]-5-(hydroxymethyl)-oxolane-3,4-diol; hydrate	$C_{15}H_{22}ClN_5O_5$	387.8
1-[3-(hydroxymethyl)-2,5-dioxoimidazolidin-4-yl]-3-[[[3-(hydroxymethyl)-2,5-dioxoimidazolidin-4-yl]-carbamoylamino]-methyl]-urea	$C_{11}H_{16}N_8O_8$	388.3
2-[[6-(3-chloroanilino)-9-propan-2-ylpurin-2-yl]-amino]-3-methylbutan-1-ol	$C_{19}H_{25}ClN_6O$	388.9
2-(5,6-dimethyl-4-oxo-3-phenylthieno[2,3-d]pyrimidin-2-yl)sulfanyl-N-(2-hydroxyethyl)-acetamide	$C_{18}H_{19}N_3O_3S_2$	389.5

N-[5-[4-chloro-3-(2-hydroxyethylsulfamoyl)-phenyl]-4-methyl-1,3-thiazol-2-yl]-acetamide	$C_{14}H16ClN_3O_4S_2$	389.9
2-[3-[2-(4-tert-butylphenoxy)-ethyl]-2-iminobenzimidazol-1-yl]-ethanol; hydrochloride	$C_{21}H_{28}ClN_3O_2$	389.9
2-[3-hydroxy-5-[2-(4-hydroxyphenyl)-ethenyl]-phenoxy]-6-(hydroxymethyl)-oxane-3,4,5-triol	$C_{20}H_{22}O_8$	390.1
N-[2-[(2,5-difluorophenyl)-methyl]-3-hydroxypropyl]-2-[2-(furan-2-yl)-5-methyl-1,3-oxazol-4-yl]-acetamide	$C_{20}H_{20}F_2N_2O_4$	390.4
3-(5-bromo-2-hydroxyphenyl)-*N*-ethyl-*N*-(2-hydroxyethyl)-3-phenylpropanamide	$C_{19}H_{22}BrNO_3$	391.3
3-hydroxy-2-(icosa-5,8,11,14-tetraenoylamino)-propanoic acid	$C_{23}H_{37}NO_4$	391.5
[3-bromo-4-[(3,4-dichlorophenyl)-methoxy]-5-methoxyphenyl]-methanol	$C_{15}H_{13}BrCl_2O_3$	392.1
gold(1+); 3,4,5-trihydroxy-6-(hydroxymethyl)-oxane-2-thiolate	$C_6H_{11}AuO_5S$	392.2
3-(3-hydroxypropyl)-2-[(3-methylquinoxalin-2-yl)-methylsulfanyl]-quinazolin-4-one	$C_{21}H_{20}N_4O_2S$	392.5
9-fluoro-11,17-dihydroxy-17-(2-hydroxyacetyl)-10,13,16-trimethyl-6,7,8,11,12,14,15,16-octahydrocyclopenta[a]-phenanthren-3-one	$C_{22}H_{29}FO_5$	392.5
4-[4-[4-(2-hydroxyethyl)piperazine-1-carbonyl]-phenyl]-2-methylphthalazin-1-one	$C_{22}H_{24}N_4O_3$	392.5
2-[[3-chloro-4-[(2-chlorophenyl)-methoxy]-5-methoxyphenyl]-methylamino]-ethanol; hydrochloride	$C_{17}H_{20}Cl_3NO_3$	392.7
3-(cyclobutylmethyl)-1,2,4,5,6,7,7a,13-octahydro-4,12-methanobenzofuro[3,2-e]-isoquinoline-4a,7,9-triol; hydrochloride	$C_{21}H_{28}ClNO_4$	393.9
3-(3-hydroxypropyl)-2-[(3-phenyl-1,2,4-oxadiazol-5-yl)methylsulfanyl]-quinazolin-4-one	$C_{20}H_{18}N_4O_3S$	394.1
1-[4-(2-hydroxyethyl)-piperazin-1-yl]-3-(4-propan-2-ylphenoxy)-propan-2-ol; dihydrochloride	$C_{18}H_{32}Cl_2N_2O_3$	394.2
(8*S*,9*R*,10*S*,11*S*,13*S*,14*S*,16*R*,17*S*)-9-fluoro-11,16,17-trihydroxy-17-(2-hydroxyacetyl)-10,13-dimethyl-6,7,8,11,12,14,15,16-octahydrocyclopenta[a]phenanthren-3-one	$C_{21}H_{27}FO_6$	394.4
2-[[5-(2-hydroxyethyl)-4-methyl-6-oxo-1*H*-pyrimidin-2-yl]-sulfanyl]-*N*,*N*-diphenylacetamide	$C_{21}H_{21}N_3O_3S$	395.5
N-(2-hydroxyethyl)-7-[5-hydroxy-2-(3-hydroxyoct-1-enyl)-3-oxocyclopentyl]-hept-5-enamide	$C_{22}H_{37}NO_5$	395.5
N-(2-hydroxyethyl)-7-[3-hydroxy-2-(3-hydroxyoct-1-enyl)-5-oxocyclopentyl]-hept-5-enamide	$C_{22}H_{37}NO_5$	395.5
2-[2-bromo-4-(hydroxymethyl)-6-methoxyphenoxy]-*N*-(2-methoxyphenyl)-acetamide	$C_{17}H_{18}BrNO_5$	396.2
2-[4-(2-hydroxyethyl)-piperazin-1-yl]-7-(4-methoxyphenyl)-4-methyl-7,8-dihydro-6*H*-quinazolin-5-one	$C_{22}H_{28}N_4O_3$	396.2
[2-(*N*-cyclohexylanilino)-2-oxoethyl] 2-(2-hydroxyethylamino)-benzoate	$C_{23}H_{28}N_2O_4$	396.5
2-[(3-bromo-5-ethoxy-4-propan-2-yloxyphenyl)-methylamino]-2-methylpropan-1-ol; hydrochloride	$C_{16}H_{27}BrClNO_3$	396.7
N,*N*-diethyl-4-[4-[2-hydroxyethyl(methyl)-amino]-piperidin-1-yl]-sulfonylbenzamide	$C_{19}H_{31}N_3O_4S$	397.5

7-[3,5-dihydroxy-2-(3-hydroxyoct-1-enyl)cyclopentyl]-*N*-(2-hydroxyethyl)-hept-5-enamide	$C_{22}H_{39}NO_5$	397.5
N-(2-hydroxyethyl)-7-[3-hydroxy-2-(3-hydroxyoct-1-enyl)-5-oxocyclopentyl]-heptanamide	$C_{22}H_{39}NO_5$	397.5
N-(1,3-dihydroxyoctadec-4-en-2-yl)-hexanamide	$C_{24}H_{47}NO_3$	397.6
2-[2-bromo-4-[[(1-hydroxy-2-methylpropan-2-yl)amino]-methyl]-6-methoxyphenoxy]-acetamide; hydrochloride	$C_{14}H_{22}BrClN_2O_4$	397.7
(9,9-dimethyl-3-oxa-9-azoniatricyclo[3.3.1.0^{2,4}]-nonan-7-yl) 3-hydroxy-2-phenylpropanoate; bromide	$C_{18}H_{24}BrNO_4$	398.3
(1,1-dimethylpyrrolidin-1-ium-3-yl) 2-cyclopentyl-2-hydroxy-2-phenylacetate;bromide	$C_{19}H_{28}BrNO_3$	398.3
4-[6-(1-adamantyl)-7-hydroxynaphthalen-2-yl]-benzoic acid	$C_{27}H_{26}O_3$	398.5
6-[3-(1-adamantyl)-4-hydroxyphenyl]-naphthalene-2-carboxylic acid	$C_{27}H_{26}O_3$	398.5
3-(3,6-dibromocarbazol-9-yl)-propane-1,2-diol	$C_{15}H_{13}Br_2NO_2$	399.1
3-fluoro-4-[[2-hydroxy-2-(5,5,8,8-tetramethyl-6,7-dihydronaphthalen-2-yl)-acetyl]-amino]-benzoic acid	$C_{23}H_{26}FNO_4$	399.5
2-[[[5-(4-chlorophenyl)thieno[2,3-d]-pyrimidin-4-yl]-amino]-methyl]-3-(furan-2-yl)-propan-1-ol	$C_{20}H_{18}ClN_3O_2S$	399.9
(9-methyl-9-oxido-3-oxa-9-azoniatricyclo[3.3.1.0^{2,4}]-nonan-7-yl) 3-hydroxy-2-phenylpropanoate; hydrobromide	$C_{17}H_{22}BrNO_5$	400.3
N-cyclohexyl-2-[4-[[(1-hydroxy-2-methylpropan-2-yl)-amino]-methyl]-2-methoxyphenoxy]-acetamide; hydrochloride	$C_{20}H_{33}ClN_2O_4$	400.9
3,4-bis(2-hydroxyethylsulfanyl)-1-(4-phenylphenyl)-pyrrole-2,5-dione	$C_{20}H_{19}NO_4S_2$	401.5
3-benzyl-2-(2-hydroxyethylamino)-spiro[6*H*-benzo[h]-quinazoline-5,1'-cyclopentane]-4-one	$C_{25}H_{27}N_3O_2$	401.5
2-amino-3,4-dihydroxy-2-(hydroxymethyl)-14-oxoicos-6-enoic acid	$C_{21}H_{39}NO_6$	401.5
(9-methyl-3-oxa-9-azatricyclo[3.3.1.0^{2,4}]-nonan-7-yl) 3-hydroxy-2-phenylpropanoate; hydrate; hydrobromide	$C_{17}H_{24}BrNO_5$	402.3
2-[4-[3-(4-acetyl-3-hydroxy-2-propylphenoxy)-propoxy]-phenoxy]-acetic acid	$C_{22}H_{26}O_7$	402.4
2-[[2-amino-5-(1-phenylethylsulfanyl)-[1,3]-thiazolo[4,5-d]-pyrimidin-7-yl]-amino]-4-methylpentan-1-ol	$C_{19}H_{25}N_5OS_2$	403.6
2-[4-[3-(2-chlorophenothiazin-10-yl)-propyl]-piperazin-1-yl]-ethanol	$C_{21}H_{26}ClN_3OS$	404.0
5-[3-[1-(2-hydroxyethylamino)-2,3-dihydro-1*H*-inden-4-yl]-1,2,4-oxadiazol-5-yl]-2-propan-2-yloxybenzonitrile	$C_{23}H_{24}N_4O_3$	404.5
N-[4-(1,1,1,3,3,3-hexafluoro-2-hydroxypropan-2-yl)-phenyl]-thiophene-2-sulfonamide	$C_{13}H_9F_6NO_3S_2$	405.3
(2*R*,3*S*,5*R*)-2-[6-[[(1*S*,2*S*)-2-hydroxycyclopentyl]-amino]-purin-9-yl]-5-(hydroxymethyl)-oxolane-3,4-diol; hydrate; hydrochloride	$C_{15}H_{24}ClN_5O_6$	405.8
8-(1-hydroxyethyl)-2-methoxy-3-[(4-methoxyphenyl)-methoxy]-benzo[c]-chromen-6-one	$C_{24}H_{22}O_6$	406.4
2-[[5-bromo-2-[(2-chlorophenyl)-methoxy]-phenyl]-methylamino]-ethanol; hydrochloride	$C_{16}H_{18}BrCl_2NO_2$	407.1
2-[[3-bromo-5-methoxy-4-(thiophen-2-ylmethoxy)-phenyl]-methylamino]-ethanol; hydrochloride	$C_{15}H_{19}BrClNO_3S$	408.7

2-[4-[[(1-hydroxy-2-methylpropan-2-yl)-amino]-methyl]-2-methoxyphenoxy]-*N*-(4-methylphenyl)-acetamide; hydrochloride	$C_{21}H_{29}ClN_2O_4$	408.9
(8*S*,9*R*,11*S*,13*S*,14*S*,16*S*)-9-chloro-11,17-dihydroxy-17-(2-hydroxyacetyl)-1,13,16-trimethyl-7,8,10,11,12,14,15,16-octahydro-6*H*-cyclopenta[a]-phenanthren-3-one	C22H29ClO5	408.9
9-chloro-11,17-dihydroxy-17-(2-hydroxyacetyl)-10,13,16-trimethyl-6,7,8,11,12,14,15,16-octahydrocyclopenta[a]phenanthren-3-one	$C_{22}H_{29}ClO_5$	408.9
1-(4-fluoro-phenyl)-3-(3-(4-fluoro-phenyl)-3-hydroxy-propyl)-4-(4-hydroxy-phenyl)-azetidin-2-one	$C_{24}H_{21}F_2NO_3$	409.1
2,3-dihydroxypropyl 2-[(7-chloroquinolin-4-yl)-amino]-benzoate; hydrochloride	$C_{19}H_{18}Cl_2N_2O_4$	409.3
1-[4-(2-hydroxyethyl)-piperazin-1-yl]-3-(5-methyl-2-propan-2-ylphenoxy)-propan-2-ol; dihydrochloride	$C_{19}H_{34}Cl_2N_2O_3$	409.4
(3*R*,4*S*)-1-(4-fluorophenyl)-3-[(3*S*)-3-(4-fluorophenyl)-3-hydroxypropyl]-4-(4-hydroxyphenyl)-azetidin-2-one	$C_{24}H_{21}F_2NO_3$	409.4
6,9-difluoro-11,17-dihydroxy-17-(2-hydroxyacetyl)-10,13,16-trimethyl-6,7,8,11,12,14,15,16-octahydrocyclopenta[a]-phenanthren-3-one	$C_{22}H_{28}F_2O_5$	410.5
1-[10-[3-[4-(2-hydroxyethyl)-piperidin-1-yl]-propyl]-phenothiazin-2-yl]-ethanone	$C_{24}H_{30}N_2O_2S$	410.6
1-(4-chlorophenyl)-2-[2-(2-hydroxyethylamino)-benzimidazol-1-yl]-ethanone; hydrobromide	$C_{17}H_{17}BrClN_3O_2$	410.7
(8-methyl-8-propan-2-yl-8-azoniabicyclo[3.2.1]-octan-3-yl) 3-hydroxy-2-phenylpropanoate; bromide	$C_{20}H_{30}BrNO_3$	412.4
(2-hydroxy-1-phenylethyl) 8-methyl-8-propan-2-yl-8-azoniabicyclo[3.2.1]-octane-3-carboxylate; bromide	$C_{20}H_{30}BrNO_3$	412.4
[1-[[3-hydroxy-2-[(6-phenylpyridine-2-carbonyl)amino]-butanoyl]-amino]-3-methylbutyl]boronic acid	$C_{21}H_{28}BN_3O_5$	413.3
(5*R*,5*aR*,8*aR*,9*R*)-5-hydroxy-9-(3,4,5-trimethoxyphenyl)-5*a*,6,8*a*,9-tetrahydro-5*H*-[2]-benzofuro[5,6-f][1,3]-benzodioxol-8-one	$C_{22}H_{22}O_8$	414.4
2-(hydroxymethyl)-4-[1-hydroxy-2-[6-(4-phenylbutoxy)-hexylamino]-ethyl]-phenol	$C_{25}H_{37}NO_4$	415.6
4-[(4-bromophenyl)-hydroxymethylidene]-1-(3-hydroxypropyl)-5-phenylpyrrolidine-2,3-dione	$C_{20}H_{18}BrNO_4$	416.3
N-[1-(ethoxymethoxy)-5-(hydroxyamino)-4-methyl-5-oxopentan-2-yl]-4-phenoxybenzamide	$C_{22}H_{28}N_2O_6$	416.5
N-(2-hydroxyethyl)-3-[6-[4-(trifluoromethoxy)-anilino]-pyrimidin-4-yl]-benzamide	$C_{20}H_{17}F_3N_4O_3$	418.4
2-(hydroxymethyl)-5-[6-[(4-nitrophenyl)-methylsulfanyl]-purin-9-yl]-oxolane-3,4-diol	$C_{17}H_{17}N_5O_6S$	419.4
(1,1-dimethylpiperidin-1-ium-3-yl) 2-hydroxy-2,2-diphenylacetate; bromide	$C_{21}H_{26}BrNO_3$	420.3
2-[[3-bromo-4-[(2-fluorophenyl)methoxy]-5-methoxyphenyl]-methylamino]-ethanol; hydrochloride	$C_{17}H_{20}BrClFNO_3$	420.7
2-[4-chloro-3-[(4-ethoxyphenyl)-methyl]-phenyl]-6-methylsulfanyloxane-3,4,5-triol	$C_{21}H_{25}ClO_5S$	424.9
N-(1,3-dihydroxypropan-2-yl)-7-[5-hydroxy-2-(3-hydroxyoct-1-enyl)-3-oxocyclopentyl]-hept-5-enamide	$C_{23}H_{39}NO_6$	425.6

(*Z*)-*N*-(1,3-dihydroxypropan-2-yl)-7-[(1*R*,2*R*,3*R*)-3-hydroxy-2-[(*E*,3*S*)-3-hydroxyoct-1-enyl]-5-oxocyclopentyl]-hept-5-enamide	$C_{23}H_{39}NO_6$	425.6
N-(1-hydroxy-3-morpholin-4-yl-1-phenylpropan-2-yl)-decanamide; hydrochloride	$C_{23}H_{39}ClN_2O_3$	426.3
2,3-dihydroxypropyl 7-[3-hydroxy-2-(3-hydroxyoct-1-enyl)-5-oxocyclopentyl]-hept-5-enoate	$C_{23}H_{38}O_7$	426.5
2,3-dihydroxypropyl 7-[5-hydroxy-2-(3-hydroxyoct-1-enyl)-3-oxocyclopentyl]-hept-5-enoate	$C_{23}H_{38}O_7$	426.5
(*Z*)-7-[(1*R*,2*R*,3*R*,5*S*)-3,5-dihydroxy-2-[(*E*,3*S*)-3-hydroxyoct-1-enyl]-cyclopentyl]-*N*-(1,3-dihydroxypropan-2-yl)-hept-5-enamide	$C_{23}H_{41}NO_6$	427.6
2,3-dihydroxypropyl 7-[3,5-dihydroxy-2-(3-hydroxyoct-1-enyl)cyclopentyl]-hept-5-enoate	$C_{23}H_{40}O_7$	428.6
4-amino-*N*-[1-(4-chlorophenyl)-3-hydroxypropyl]-1-(7*H*-pyrrolo[2,3-d]-pyrimidin-4-yl)-piperidine-4-carboxamide	$C_{21}H_{25}ClN_6O_2$	428.9
11-hydroxy-8-(2-hydroxyacetyl)-9,13-dimethyl-6-propyl-5,7-dioxapentacyclo[10.8.0.0^{2,9}.0^{4,8}.0^{13,18}]-icosa-14,17-dien-16-one	C25H34O6	430.5
2-[[3-bromo-5-methoxy-4-(pyridin-3-ylmethoxy)-phenyl]-methylamino]-2-methylpropan-1-ol; hydrochloride	$C_{18}H_{24}BrClN_2O_3$	431.7
(1-methyl-1-azoniabicyclo[2.2.2]-octan-3-yl) 2-hydroxy-2,2-diphenylacetate; bromide	$C_{22}H_{26}BrNO_3$	432.3
5-hydroxy-2-(4-hydroxyphenyl)-7-[3,4,5-trihydroxy-6-(hydroxymethyl)-oxan-2-yl]-oxychromen-4-one	$C_{21}H_{20}O_{10}$	432.4
5,7-dihydroxy-2-(4-hydroxyphenyl)-8-[3,4,5-trihydroxy-6-(hydroxymethyl)-oxan-2-yl]-chromen-4-one	$C_{21}H_{20}O_{10}$	432.4
2-chloro-4-[[2-[(1-hydroxy-3-methylbutan-2-yl)amino]-9-propan-2-ylpurin-6-yl]-amino]-benzoic acid	$C_{20}H_{25}ClN_6O_3$	432.9
N-[1-(3-chlorophenyl)-2-hydroxyethyl]-4-[5-chloro-2-(propan-2-ylamino)-pyridin-4-yl]-1*H*-pyrrole-2-carboxamide	$C_{21}H_{22}Cl_2N_4O_2$	433.3
sodium; (*E*,3*S*,5*R*)-7-[3-(4-fluorophenyl)-1-propan-2-ylindol-2-yl]-3,5-dihydroxyhept-6-enoate	$C_{24}H_{25}FNNaO_4$	433.4
2-[2-amino-6-[(4-nitrophenyl)-methylsulfanyl]-purin-9-yl]-5-(hydroxymethyl)-oxolane-3,4-diol	$C_{17}H_{18}N_6O_6S$	434.4
7-[2-hydroxy-3-[2-hydroxyethyl(methyl)-amino]-propyl]-1,3-dimethylpurine-2,6-dione; pyridine-3-carboxylic acid	$C_{19}H_{26}N_6O_6$	434.4
7-[4-(3-ethynylanilino)-7-methoxyquinazolin-6-yl]-oxy-*N*-hydroxyheptanamide	$C_{24}H_{26}N_4O_4$	434.5
19-fluoro-11-hydroxy-8-(2-hydroxyacetyl)-6,6,9,13-tetramethyl-5,7-dioxapentacyclo[10.8.0.0^{2,9}.0^{4,8}.0^{13,18}]-icosa-14,17-dien-16-one	$C_{24}H_{31}FO_6$	434.5
N-[(4-amino-2-methylpyrimidin-5-yl)methyl]-*N*-[5-hydroxy-3-(oxolan-2-ylmethyldisulfanyl)-pent-2-en-2-yl]-formamide; hydrochloride	$C_{17}H_{27}ClN_4O_3S_2$	435.0
2-[4-(3-benzo[b][1]-benzazepin-11-ylpropyl)-piperazin-1-yl]-ethanol; dihydrochloride	$C_{23}H_{31}Cl_2N_3O$	436.4
1-[2,4-dihydroxy-6-[3,4,5-trihydroxy-6-(hydroxymethyl)-oxan-2-yl]-oxyphenyl]-3-(4-hydroxyphenyl)-propan-1-one	$C_{21}H_{24}O_{10}$	436.4
19-fluoro-11-hydroxy-8-(2-hydroxyacetyl)-6,6,9,13-tetramethyl-5,7-dioxapentacyclo[10.8.0.0^{2,9}.0^{4,8}.0^{13,18}]-icos-17-en-16-one	$C_{24}H_{33}FO_6$	436.5

2-[4-[3-(2-chlorothioxanthen-9-ylidene)-propyl]-piperazin-1-yl]-ethanol; hydrochloride	$C_{22}H_{26}Cl_2N_2OS$	437.4
2-[2-(3-hydroxypropylamino)-benzimidazol-1-yl]-1-naphthalen-1-ylethanone; hydrobromide	$C_{22}H_{22}BrN_3O_2$	440.3
(9-butyl-9-methyl-3-oxa-9-azoniatricyclo[3.3.1.0^{2,4}]-nonan-7-yl) 3-hydroxy-2-phenylpropanoate; bromide	$C_{21}H_{30}BrNO_4$	440.4
1-[1-(4-chloro-3-fluorophenyl)-2-hydroxyethyl]-4-[2-[(2-methylpyrazol-3-yl)-amino]-pyrimidin-4-yl]-pyridin-2-one	$C_{21}H_{18}ClFN_6O_2$	440.9
6-[2-[2-hydroxyethyl-[3-(4-nitrophenyl)-propyl]-amino]-ethylamino]-1,3-dimethylpyrimidine-2,4-dione; hydrochloride	$C_{19}H_{28}ClN_5O_5$	441.9
3’-[2-[1-(3,4-dimethylphenyl)-3-methyl-5-oxo-4,5-dihydro-1*H*-pyrazol-4-ylidene]-hydrazin-1-yl]-2‘-hydroxy-[1,1’-biphenyl]-3-cyrboxylic acid	$C_{25}H_{22}N_4O_4$	442.5
3*a*-(hydroxymethyl)-5*a*,5*b*,8,8,11*a*-pentamethyl-1-prop-1-en-2-yl-1,2,3,4,5,6,7,7*a*,9,10,11,11*b*,12,13,13*a*,13*b*-hexadecahydrocyclopenta[a]-chrysen-9-ol	$C_{30}H_{50}O_2$	442.7
2-chloro-6,9-difluoro-11,17-dihydroxy-17-(2-hydroxyacetyl)-10,13,16-trimethyl-6,7,8,11,12,14,15,16-octahydrocyclopenta[a]-phenanthren-3-one	$C_{22}H_{27}ClF_2O_5$	444.9
3-(3-hydroxy-4-methoxyphenyl)-7-[3,4,5-trihydroxy-6-(hydroxymethyl)-oxan-2-yl]-oxychromen-4-one	$C_{22}H_{22}O_{10}$	446.4
N-(4-ethoxyphenyl)-4-[hydroxy(diphenyl)-methyl]-piperidine-1-carbothioamide	$C_{27}H_{30}N_2O_2S$	446.6
2-[2-[4-[(4-chlorophenyl)-phenylmethyl]-piperazin-1-yl]-ethoxy]-ethanol; dihydrochloride	$C_{21}H_{29}Cl_3N_2O_2$	447.8
2-(3,4-dihydroxyphenyl)-5-hydroxy-7-[3,4,5-trihydroxy-6-(hydroxymethyl)-oxan-2-yl]-oxychromen-4-one	$C_{21}H_{20}O_{11}$	448.4
(3,6-diiodocarbazol-9-yl)-methanol	$C_{13}H_9I_2NO$	449.0
11-[4-(hydroxyiminomethyl)-phenyl]-17-methoxy-17-(methoxymethyl)-13-methyl-1,2,6,7,8,11,12,14,15,16-decahydrocyclopenta[a]-phenanthren-3-one	$C_{28}H_{35}NO_4$	449.6
1-(4-hydroxy-3-methoxyphenyl)-ethane-1,2-diol; piperazine	$C_{22}H_{34}N_2O_8$	454.5
but-2-enedioic acid; *N*-(1-hydroxybutan-2-yl)-7-methyl-6,6a,8,9-tetrahydro-4*H*-indolo[4,3-fg]-quinoline-9-carboxamide	$C_{24}H_{29}N_3O_6$	455.5
but-2-enedioic acid; *N*-(1-hydroxybutan-2-yl)-7-methyl-6,6a,8,9-tetrahydro-4*H*-indolo[4,3-fg]-quinoline-9-carboxamide	$C_{24}H_{29}N_3O_6$	455.5
6-(4-bromo-2-chloroanilino)-7-fluoro-*N*-(2-hydroxyethoxy)-3-methylbenzimidazole-5-carboxamide	$C_{17}H_{15}BrClFN_4O_3$	457.7
8-[(dimethylamino)-methyl]-19-ethyl-7,19-dihydroxy-17-oxa-3,13-diazapentacyclo[11.8.0.0^{2,11}.0^{4,9}.0^{15,20}]-henicosa-1(21),2,4(9),5,7,10,15(20)-heptaene-14,18-dione; hydrochloride	$C_{23}H_{24}ClN_3O_5$	457.9
1-(5-bromothiophen-2-yl)-2-[2-(2-hydroxyethylamino)-benzimidazol-1-yl]-ethanone; hydrobromide	$C_{15}H_{15}Br_2N_3O_2S$	431.2
N-(1,3-dihydroxypropan-2-yl)-7-[3-hydroxy-2-(3-hydroxy-5-phenylpent-1-enyl)-5-oxocyclopentyl]-hept-5-enamide	$C_{26}H_{37}NO_6$	459.3
potassium; [2-butyl-5-chloro-3-[[4-[2-(1,2,3-triaza-4-azanidacyclopenta-2,5-dien-5-yl)-phenyl]-phenyl]-methyl]-imidazol-4-yl]-methanol	$C_{22}H_{22}ClKN_6O$	461.0

potassium; [2-butyl-5-chloro-3-[[4-[2-(1,2,3-triaza-4-azanidacyclopenta-2,5-dien-5-yl)-phenyl]-phenyl]-methyl]-imidazol-4-yl]-methan	$C_{22}H_{22}ClKN_6O$	461.0
5-[[3-chloro-4-(2,3-dihydroxypropoxy)-phenyl]-methylidene]-3-(2-methylphenyl)-2-propylimino-1,3-thiazolidin-4-one	$C_{23}H_{25}ClN_2O_4S$	461.0
2-(2-fluoro-4-iodoanilino)-*N*-(2-hydroxyethoxy)-1,5-dimethyl-6-oxopyridine-3-carboxamide	$C_{16}H_{17}FIN_3O_4$	461.2
3,5-dihydroxy-2-[(*E*,3*S*)-3-hydroxy-5-phenylpent-1-enyl]-cyclopentyl]-*N*-(1,3-dihydroxypropan-2-yl)-hept-5-enamide	$C_{26}H_{39}NO_6$	461.6
2-(3,4-dihydroxyphenyl)-5,7-dihydroxy-3-[3,4,5-trihydroxy-6-(hydroxymethyl)-oxan-2-yl]-oxychromen-4-one	$C_{21}H_{20}O_{12}$	464.4
2-(3,4-dihydroxyphenyl)-5,7-dihydroxy-3-[3,4,5-trihydroxy-6-(hydroxymethyl)-oxan-2-yl]-oxychromen-4-one	$C_{17}H_{25}NO_{14}$	464.4
2-[4-chloro-3-[[4-(2-cyclopropyloxyethoxy)-phenyl]-methyl]-phenyl]-6-(hydroxymethyl)-oxane-3,4,5-triol	$C_{24}H_{29}ClO_7$	464.9
[5-[2-(2,6-dimethylmorpholin-4-yl)-4-morpholin-4-ylpyrido[2,3-d]pyrimidin-7-yl]-2-methoxyphenyl]methanol	$C_{25}H_{31}N_5O_4$	465.5
[5-[2,4-bis(3-methylmorpholin-4-yl)-pyrido[2,3-d]-pyrimidin-7-yl]-2-methoxyphenyl]-methanol	$C_{25}H_{31}N_5O_4$	465.6
1-(3,5-di*tert*-butyl-4-hydroxyphenyl)-2-[2-(3-hydroxypropylamino)-5,6-dimethyl-1,3-benzodiazol-1-yl]-ethanone	$C_{28}H_{39}N_3O_3$	465.6
4-amino-2-[4,6-diamino-3-[3-amino-6-(aminomethyl)-5-hydroxyoxan-2-yl]oxy-2-hydroxycyclohexyl]-oxy-6-(hydroxymethyl)-oxane-3,5-diol	$C_{18}H_{37}N_5O_9$	467.5
2-[2-hydroxyethyl-[2-[methyl-(2-methyl-1-phenylpropan-2-yl)-amino]-2-oxoethyl]-amino]-*N*-methyl-N-(2-methyl-1-phenylpropan-2-yl)-acetamide	$C_{28}H_{41}N_3O_3$	467.6
but-2-enedioic acid; *N*-(1-hydroxybutan-2-yl)-4,7-dimethyl-6,6*a*,8,9-tetrahydroindolo[4,3-fg]-quinoline-9-carboxamide	$C_{25}H_{31}N_3O_6$	469.5
2-[4-[[2-[3-fluoro-4-(trifluoromethyl)-phenyl]-4-methyl-1,3-thiazol-5-yl]-methylsulfanyl]-2-methylphenoxy]-acetic acid	$C_{21}H_{17}F_4NO_3S_2$	471.5
1-(4-tert-butylphenyl)-4-[4-[hydroxy(diphenyl)-methyl]-piperidin-1-yl]-butan-1-ol	$C_{32}H_{41}NO_2$	471.7
2-[2-ethoxy-4-(4-hydroxypiperidin-1-yl)-anilino]-5,11-dimethylpyrimido[4,5-b][1,4]-benzodiazepin-6-one	$C_{26}H_{30}N_6O_3$	474.6
N-[2-[(2,3-difluorophenyl)-methylsulfanyl]-6-(3,4-dihydroxybutan-2-ylamino)-pyrimidin-4-yl]-azetidine-1-sulfonamide	$C_{18}H_{23}F_2N_5O_4S_2$	475.5
4-[2-(4-benzylpiperidin-1-yl)-1-hydroxypropyl]-phenol;2,3-dihydroxybutanedioic acid	$C_{25}H_{33}NO_8$	475.5
2-amino-2-methoxypropane-1,3-diol; (*Z*)-7-[(1*R*,2*R*,3*R*,5*S*)-3,5-dihydroxy-2-[(*E*,3*S*)-3-hydroxyoct-1-enyl]-cyclopentyl]-hept-5-enoic acid	$C_{24}H_{45}NO_8$	475.6
2-amino-2-(hydroxymethyl)-propane-1,3-diol; 7-[3,5-dihydroxy-2-(3-hydroxyoct-1-enyl)-cyclopentyl]hept-5-enoic acid	$C_{24}H_{45}NO_8$	475.6
N-[5-[[4-(1,1,1,3,3,3-hexafluoro-2-hydroxypropan-2-yl)-phenyl]-sulfamoyl]-4-methyl-1,3-thiazol-2-yl]-acetamide	$C_{15}H_{13}F_6N_3O_4S_2$	477.4
(*E*)-but-2-enedioic acid; *N*-[2-hydroxy-5-[1-hydroxy-2-[1-(4-methoxyphenyl)propan-2-ylamino]-ethyl]-phenyl]-formamide; hydrate	$C_{23}H_{30}N_2O_9$	478.5

4-(dimethylamino)-1,5,10,11,12*a*-pentahydroxy-6-methylidene-3,12-dioxo-4,4*a*,5,5*a*-tetrahydrotetracene-2-carboxamide; hydrochloride	$C_{22}H_{23}ClN_2O_8$	478.9
4-fluoro-2-[4-[[4-(2-hydroxypropan-2-yl)-pyrrolidin-3-yl]-amino]-6,7-dimethoxyquinazolin-2-yl]-phenol; hydrochloride	$C_{23}H_{28}ClFN_4O_4$	478.9
2-[[2-(3,5-difluorophenyl)-2-hydroxyacetyl]-amino]-*N*-(5-methyl-6-oxo-7*H*-benzo[d][1]-benzazepin-7-yl)-propanamide	$C_{26}H_{23}F_2N_3O_4$	479.5
4-[[2-(3-chlorophenyl)-2-hydroxyethyl]-amino]-3-(4-methyl-6-morpholin-4-yl-1*H*-benzimidazol-2-yl)-1*H*-pyridin-2-one	$C_{25}H_{26}ClN_5O_3$	480.0
4-(dimethylamino)-1,5,10,11,12*a*-pentahydroxy-6-methyl-3,12-dioxo-4*a*,5,5*a*,6-tetrahydro-4*H*-tetracene-2-carboxamide; hydrochloride	$C_{22}H_{25}ClN_2O_8$	480.9
4-(dimethylamino)-1,6,10,11,12*a*-pentahydroxy-6-methyl-3,12-dioxo-4,4*a*,5,5*a*-tetrahydrotetracene-2-carboxamide; hydrochloride	$C_{22}H_{25}ClN_2O_8$	480.9
7-[4-(4-fluorophenyl)-2-[methyl(methylsulfonyl)-amino]-6-propan-2-ylpyrimidin-5-yl]-3,5-dihydroxyhept-6-enoic acid	$C_{22}H_{28}FN_3O_6S$	481.5
N-(2,3-dihydroxypropoxy)-3,4-difluoro-2-(2-fluoro-4-iodoanilino)-benzamide	$C_{16}H_{14}F3IN_2O_4$	482.2
2-hydroxy-4-[4-[5-(2-methyl-3-phenylprop-2-enylidene)-4-oxo-2-sulfanylidene-1,3-thiazolidin-3-yl]-butanoylamino]-benzoic acid	$C_{24}H_{22}N_2O_5S_2$	482.6
sodium; (2*R*)-*N*-[(6*R*,7*R*)-2-carboxy-3-[(1-methyltetrazol-5-yl)-sulfanylmethyl]-8-oxo-5-thia-1-azabicyclo[4.2.0]-oct-2-en-7-yl]-2-hydroxy-2-phenylethanimidate	$C_{18}H_{17}N_6NaO_5S_2$	484.5
N-(2-chloro-6-methylphenyl)-2-[[6-[4-(2-hydroxyethyl)-piperazin-1-yl]-2-methylpyrimidin-4-yl]-amino]-1,3-thiazole-5-carboxamide	$C_{22}H_{26}ClN_7O_2S$	488.0
2-[[9-propan-2-yl-6-(3-pyridin-2-ylanilino)-purin-2-yl]-amino]-butan-1-ol; dihydrochloride	$C_{23}H_{29}Cl_2N_7O$	490.4
7-(dimethylamino)-1,10,11,12*a*-tetrahydroxy-3,12-dioxo-4-propan-2-yl-4*a*,5,5a,6-tetrahydro-4*H*-tetracene-2-carboxamide; hydrochloride	$C_{24}H_{29}ClN_2O_7$	492.3
4-[2-(4-benzylpiperidin-1-yl)-1-hydroxypropyl]-phenol; 2,3-dihydroxybutanedioic acid; hydrate	$C_{25}H_{35}NO_9$	493.5
4,7-bis(dimethylamino)-1,10,11,12*a*-tetrahydroxy-3,12-dioxo-4a,5,5a,6-tetrahydro-4*H*-tetracene-2-carboxamide; hydrochloride	$C_{23}H_{28}ClN_3O_7$	493.9
[4*a*,5-dihydroxy-7-methyl-1-[3,4,5-trihydroxy-6-(hydroxymethyl)-oxan-2-yl]oxy-1,5,6,7*a*-tetrahydrocyclopenta[c]-pyran-7-yl] 3-phenylprop-2-enoate	$C_{24}H_{30}O_{11}$	494.5
calcium; 3-[(2,4-dihydroxy-3,3-dimethylbutanoyl)-amino]-propanoate; hydrate	$C_{18}H_{34}CaN_2O_{11}$	494.5
1-(3-hydroxypropyl)-5-[2-[2-[2-(2,2,2-trifluoroethoxy)-phenoxy]-ethylamino]-propyl]-2,3-dihydroindole-7-carboxamide	$C_{25}H_{32}F_3N_3O_4$	495.5
4-(dimethylamino)-1,5,6,10,11,12*a*-hexahydroxy-6-methyl-3,12-dioxo-4,4*a*,5,5*a*-tetrahydrotetracene-2-carboxamide; dihydrate	$C_{22}H_{28}N_2O_{11}$	496.5
9-acetyl-7-(4-amino-5-hydroxy-6-methyloxan-2-yl)-oxy-6,9,11-trihydroxy-8,10-dihydro-7*H*-tetracene-5,12-dione	$C_{26}H_{27}NO_9$	497.5
N-[[3-[3-[2-[4-(benzenesulfonamido)-phenyl]-ethylamino]-2-hydroxypropoxy]-phenyl]-methyl]-acetamide	$C_{26}H_{31}N_3O_5S$	497.6
2-[2-(4-benzo[b][1,4]-benzothiazepin-6-ylpiperazin-1-yl)-ethoxy]-ethanol; (*E*)-but-2-enedioic acid	$C_{25}H_{29}N_3O_6S$	499.6
4-[2-(2-chloro-4-fluoroanilino)-5-methylpyrimidin-4-yl]-*N*-[1-(3-chlorophenyl)-2-hydroxyethyl]-1*H*-pyrrole-2-carboxamide	$C_{24}H_{20}Cl_2FN_5O_2$	500.3

1-[6-(2-hydroxypropan-2-yl)-pyridin-2-yl]-6-[4-(4-methylpiperazin-1-yl)-anilino]-2-prop-2-enylpyrazolo[3,4-d]pyrimidin-3-one	$C_{27}H_{32}N_8O_2$	500.6
7-chloro-4-(dimethylamino)-1,6,10,11,12*a*-pentahydroxy-3,12-dioxo-4*a*,5,5*a*,6-tetrahydro-4*H*-tetracene-2-carboxamide; hydrochloride	$C_{21}H_{22}Cl_2N_2$O8	501.3
1-[5-chloro-6-(4-chlorophenyl)-1,3-benzoxazol-2-yl]-*N*-[3-(hydroxymethyl)-cyclohexyl]-piperidine-4-carboxamide	$C_{26}H_{29}Cl_2N_3$O3	502.4
2-[[2-[bis(2-hydroxyethyl)-amino]-4,8-di(piperidin-1-yl)-pyrimido[5,4-d]-pyrimidin-6-yl]-(2-hydroxyethyl)-amino]-ethanol	$C_{24}H_{40}N_8O_4$	504.6
2-[3-[[7-[3-[ethyl(2-hydroxyethyl)-amino]-propoxy]-quinazolin-4-yl]-amino]-1*H*-pyrazol-5-yl]-*N*-(3-fluorophenyl)-acetamide	$C_{26}H_{30}FN_7O_3$	507.6
7-chloro-4-(dimethylamino)-1,6,10,11,12*a*-pentahydroxy-6-methyl-3,12-dioxo-4,4*a*,5,5*a*-tetrahydrotetracene-2-carboxamide; hydrochloride	$C_{22}H_{24}Cl_2N_2O_8$	515.3
1,4-dihydroxy-5,8-bis[2-(2-hydroxyethylamino)-ethylamino]-anthracene-9,10-dione; dihydrochloride	$C_{22}H_{30}Cl_2N_4O_6$	517.4
5-[1-hydroxy-2-(propan-2-ylamino)-ethyl]-benzene-1,3-diol; sulfuric acid	$C_{22}H_{36}N_2O_{10}S$	520.6
1-(9H-carbazol-4-yloxy)-3-[2-(2-methoxyphenoxy)-ethylamino]-propan-2-ol; phosphoric acid; hydrate	$C_{24}H_{31}N_2O_9P$	522.5
3-[7-[[2-(3,4-difluorophenyl)-cyclopropyl]-amino]-5-propylsulfanyltriazolo[4,5-d]-pyrimidin-3-yl]-5-(2-hydroxyethoxy)-cyclopentane-1,2-diol	$C_{23}H_{28}F_2N_6O_4S$	522.6
4-(dimethylamino)-1,6,10,11,12*a*-pentahydroxy-6-methyl-3,12-dioxo-*N*-(pyrrolidin-1-ylmethyl)-4,4*a*,5,5*a*-tetrahydrotetracene-2-carboxamide	$C_{27}H_{33}N_3O_8$	527.6
[4,5-dihydroxy-6-(hydroxymethyl)-2-[2-[(1-hydroxy-6-oxocyclohex-2-ene-1-carbonyl)-oxymethyl]-phenoxy]-oxan-3-yl] benzoate	$C_{27}H_{28}O_{11}$	528.5
9-acetyl-7-(4-amino-5-hydroxy-6-methyloxan-2-yl)-oxy-6,9,11-trihydroxy-8,10-dihydro-7*H*-tetracene-5,12-dione; hydrochloride	$C_{26}H_{28}ClNO_9$	534.0
1-[3-methoxy-4-[[4-(2-propan-2-ylsulfonylanilino)-1*H*-pyrrolo[2,3-b]-pyridin-6-yl]-amino]-phenyl]-piperidin-4-ol	$C_{28}H_{33}N_5O_4S$	535.7
5-amino-2-[[7-amino-6-(4,6-diamino-2,3-dihydroxycyclohexyl)-oxy-4-hydroxy-3-(methylamino)-2,3,4,4*a*,6,7,8,8*a*-octahydropyrano[3,2-b]-pyran-2-yl]-oxy]-6-(hydroxymethyl)-oxane-3,4-diol	$C_{21}H_{41}N_5O_{11}$	539.6
2-[[9-propan-2-yl-6-[(4-pyridin-2-ylphenyl)-methylamino]-purin-2-yl]-amino]-butan-1-ol; trihydrochloride	$C_{24}H_{32}Cl_3N_7O$	540.9
2-amino-*N*-[5-[6-(dimethylamino)-purin-9-yl]-4-hydroxy-2-(hydroxymethyl)-oxolan-3-yl]-3-(4-methoxyphenyl)-propanamide; dihydrochloride	$C_{22}H_{31}Cl_2N_7O_5$	544.4
5-[2-(tert-butylamino)-1-hydroxyethyl]-benzene-1,3-diol; sulfuric acid	$C_{24}H_{40}N_2O_{10}S$	548.6
4-*tert*-butyl-*N*-[6-(2-hydroxyethoxy)-5-(2-methoxyphenoxy)-2-pyrimidin-2-ylpyrimidin-4-yl]-benzenesulfonamide	$C_{27}H_{29}N_5O_6S$	551.6
5-amino-2-(aminomethyl)-6-[4,6-diamino-2-[3,4-dihydroxy-5-(hydroxymethyl)-oxolan-2-yl]-oxy-3-hydroxycyclohexyl]-oxyoxane-3,4-diol; sulfuric acid	$C_{17}H_{36}N_4O_{14}S$	552.6
N-hydroxy-1-(2-methoxyethyl)-4-[4-[4-(trifluoromethoxy)-phenoxy]-phenyl]-sulfonylpiperidine-4-carboxamide; hydrochloride	$C_{22}H_{26}ClF_3N_2O_7S$	555.0
4-[[5-[6-amino-8-[(3,4-dichlorophenyl)-methylamino]-purin-9-yl]-3,4-dihydroxyoxolan-2-yl]methoxymethyl]-benzonitrile	$C_{25}H_{23}Cl_2N_7O_4$	556.4

4-[3-(4-bromophenyl)-5-(6-methyl-2-oxo-4-phenyl-1*H*-quinolin-3-yl)-3,4-dihydropyrazol-2-yl]-4-oxobutanoic acid	$C_{29}H_{24}BrN_3O_4$	558.4
N-[1,2-dimethoxy-10-methylsulfanyl-9-oxo-3-[3,4,5-trihydroxy-6-(hydroxymethyl)-oxan-2-yl]-oxy-6,7-dihydro-5*H*-benzo[a]-heptalen-7-yl]-acetamide	$C_{27}H_{33}NO_{10}S$	563.6
9-acetyl-7-(4-amino-5-hydroxy-6-methyloxan-2-yl)-oxy-6,9,11-trihydroxy-4-methoxy-8,10-dihydro-7*H*-tetracene-5,12-dione; hydrochloride	$C_{27}H_{30}ClNO_{10}$	564.0
7-(4-amino-5-hydroxy-6-methyloxan-2-yl)-oxy-6,9,11-trihydroxy-9-(2-hydroxyacetyl)-4-methoxy-8,10-dihydro-7*H*-tetracene-5,12-dione; hydrochloride	$C_{27}H_{30}ClNO_{11}$	580.0
2-(aminomethyl)-6-[4,6-diamino-3-[4-amino-3,5-dihydroxy-6-(hydroxymethyl)-oxan-2-yl]oxy-2-hydroxycyclohexyl]-oxyoxane-3,4,5-triol; sulfuric acid	$C_{18}H_{38}N_4O_{15}S$	582.6
3-[1,5,11,14-tetrahydroxy-10-(hydroxymethyl)-13-methyl-3-(3,4,5-trihydroxy-6-methyloxan-2-yl)oxy-2,3,4,6,7,8,9,11,12,15,16,17-dodecahydro-1*H*-cyclopenta[a]-phenanthren-17-yl]-2*H*-furan-5-one	$C_{29}H_{44}O_{12}$	584.7
1-hexyl-3-[4-[[4-[2-[[2-hydroxy-3-(4-hydroxyphenoxy)-propyl]-amino]-ethyl]-phenyl]-sulfamoyl]-phenyl]-urea	$C_{30}H_{40}N_4O_6S$	584.7
4-[1,5,11,14-tetrahydroxy-10-(hydroxymethyl)-13-methyl-3-(3,4,5-trihydroxy-6-methyloxan-2-yl)-oxy-2,3,4,6,7,8,9,11,12,15,16,17-dodecahydro-1*H*-cyclopenta[a]-phenanthren-17-yl]-oxolan-2-one	$C_{29}H_{46}O_{12}$	586.7
5-[(7,8-dihydroxy-2-methyl-4,4*a*,6,7,8,8*a*-hexahydropyrano[3,2-d][1,3]-dioxin-6-yl)-oxy]-9-(4-hydroxy-3,5-dimethoxyphenyl)-5*a*,6,8*a*,9-tetrahydro-5*H*-[2]-benzofuro[6,5-f][1,3]-benzodioxol-8-one	C29H32O13	588.6
(5*S*,5*aR*,8*aR*,9*R*)-5-[[(2*R*,4*aR*,6*R*,7*R*,8*aS*)-7,8*a*-dihydroxy-2-methyl-4*a*,6,7,8-tetrahydro-4*H*-pyrano[3,2-d][1,3]-dioxin-6-yl]-oxy]-9-(4-hydroxy-3,5-dimethoxyphenyl)-5*a*,6,8*a*,9-tetrahydro-5H-[2]-benzofuro[6,5-f][1,3]-benzodioxol-8-one	$C_{29}H_{32}O_{13}$	588.6
(5*R*,5*aR*,8*aR*,9*R*)-5-[[(2*R*,4*aR*,6*R*,7*R*,8*R*,8*aS*)-7,8-dihydroxy-2-methyl-4,4*a*,6,7,8,8*a*-hexahydropyrano[3,2-d][1,3]-dioxin-6-yl]-oxy]-9-(4-hydroxy-3,5-dimethoxyphenyl)-5*a*,6,8*a*,9-tetrahydro-5*H*-[2]benzofuro[6,5-f][1,3]-benzodioxol-8-one	$C_{29}H_{32}O_{13}$	588.6
4-[5-(4-carbamimidoylphenoxy)-pentoxy]-benzenecarboximidamide; 2-hydroxyethylsulfite	$C_{23}H_{34}N_4O_{10}S_2$	590.7
3-chloro-*N*-[1-[[2-(dimethylamino)-acetyl]-amino]-3-[4-[8-(1-hydroxyethyl)imidazo[1,2-a]-pyridin-2-yl]-phenyl]-propan-2-yl]-4-propan-2-yloxybenzamide	$C_{32}H_{38}ClN_5O_4$	592.1
4-[5-(4-carbamimidoylphenoxy)-pentoxy]-benzenecarboximidamide; 2-hydroxyethanesulfonic acid	$C_{23}H_{36}N_4O_{10}$S2	592.7
7-[4,5-dihydroxy-6-(hydroxymethyl)-3-(3,4,5-trihydroxy-6-methyloxan-2-yl)-oxyoxan-2-yl]-oxy-5-hydroxy-2-(4-hydroxyphenyl)-2,3-dihydrochromen-4-one; hydrate	$C_{27}H_{34}O_{15}$	598.5
6-amino-2-[[[4-(dimethylamino)-1,6,10,11,12*a*-pentahydroxy-6-methyl-3,12-dioxo-4,4*a*,5,5*a*-tetrahydrotetracene-2-carbonyl]-amino]-methylamino]-hexanoic acid	$C_{29}H_{38}N_4O_{10}$	602.6
2-(hydroxymethyl)-4-[1-hydroxy-2-[6-(4-phenylbutoxy)-hexylamino]-ethyl]-phenol; 1-hydroxynaphthalene-2-carboxylic acid	$C_{36}H_{45}NO_7$	603.7
6-cyclopropyl-8-fluoro-2-[2-(hydroxymethyl)-3-[1-methyl-5-[[5-(4-methylpiperazin-1-yl)-pyridin-2-yl]-amino]-6-oxopyridin-3-yl]phenyl]-isoquinolin-1-one	$C_{35}H_{35}FN_6O_3$	606.7

2-(3,4-dihydroxyphenyl)-5,7-dihydroxy-3-[3,4,5-trihydroxy-6-[(3,4,5-trihydroxy-6-methyloxan-2-yl)-oxymethyl]-oxan-2-yl]-oxychromen-4-one	$C_{27}H_{30}O_{16}$	610.5
5-hydroxy-2-(3-hydroxy-4-methoxyphenyl)-7-[3,4,5-trihydroxy-6-[(3,4,5-trihydroxy-6-methyloxan-2-yl)-oxymethyl]-oxan-2-yl]-oxy-2,3-dihydrochromen-4-one	$C_{28}H_{34}O_{15}$	610.6
sodium; 6-[7-[5-ethyl-5-(5-ethyl-5-hydroxy-6-methyloxan-2-yl)-3-methyloxolan-2-yl]-4-hydroxy-3,5-dimethyl-6-oxononyl]-2-hydroxy-3-methylbenzoate	$C_{34}H_{53}NaO_{8}$	612.8
[13-acetyloxy-1,6-dihydroxy-8-(hydroxymethyl)-4,12,12,15-tetramethyl-5-oxo-14-tetracyclo[8.5.0.0^{2,6}.0^{11,13}]-pentadeca-3,8-dienyl] tetradecanoate	$C_{36}H_{56}O_{8}$	616.8
8-[[2-[3,4-bis(hydroxymethyl)-3,4-dimethylpyrrolidin-1-yl]-5-chloropyridine-4-carbonyl]amino]-1-(4-fluorophenyl)-4,5-dihydrobenzo[g]-indazole-3-carboxamide	$C_{32}H_{32}ClFN_{6}O_{4}$	619.1
4-amino-*N*-[(1*S*,2*R*,3*R*,4*S*,5*R*)-5-amino-2-[(2*S*,3*R*,4*S*,5*S*,6*R*)-4-amino-3,5-dihydroxy-6-(hydroxymethyl)-oxan-2-yl]-oxy-4-[(2*R*,3*R*,4*S*,5*S*,6*R*)-6-(aminomethyl)-3,4,5-trihydroxyoxan-2-yl]-oxy-3-hydroxycyclohexyl]-2-hydroxybutanamide; dihydrate	$C_{22}H_{47}N_{5}O_{15}$	621.6
1-[5-chloro-1-(2,4-dimethoxyphenyl)-sulfonyl-3-(2-methoxyphenyl)-2-oxoindol-3-yl]-4-hydroxy-*N,N*-dimethylpyrrolidine-2-carboxamide	$C_{30}H_{32}ClN_{3}O_{8}S$	630.1
5-[5-[3,4-dihydroxy-6-methyl-5-[[4,5,6-trihydroxy-3-(hydroxymethyl)-cyclohex-2-en-1-yl]-amino]-oxan-2-yl]-oxy-3,4-dihydroxy-6-(hydroxymethyl)-oxan-2-yl]-oxy-6-(hydroxymethyl)-oxane-2,3,4-triol	$C_{25}H_{43}NO_{18}$	645.6
2-amino-3-[4-(4-hydroxy-3-iodophenoxy)-3,5-diiodophenyl]-propanoic acid	$C_{15}H_{12}I_{3}NO_{4}$	651.0
N-[1-[4-[2-[(2,4-dichlorophenyl)-sulfonylamino]-3-hydroxypropanoyl]-piperazin-1-yl]-4-methyl-1-oxopentan-2-yl]-1-benzothiophene-2-carboxamide	$C_{28}H_{32}Cl_{2}N_{4}O_{6}S_{2}$	655.6
5-[(7,8-dihydroxy-2-thiophen-2-yl-4,4*a*,6,7,8,8*a*-hexahydropyrano[3,2-d][1,3]-dioxin-6-yl)-oxy]-9-(4-hydroxy-3,5-dimethoxyphenyl)-5*a*,6,8*a*,9-tetrahydro-5*H*-[2]-benzofuro[6,5-f][1,3]-benzodioxol-8-one	$C_{32}H_{32}O_{13}S$	656.7
(3*S*,4*aS*,8*aS*)-*N*-*tert*-butyl-2-[(2*R*,3*R*)-2-hydroxy-3-[(3-hydroxy-2-methylbenzoyl)-amino]-4-phenylsulfanylbutyl]-3,4,4*a*,5,6,7,8,8*a*-octahydro-1*H*-isoquinoline-3-carboxamide; methanesulfonic acid	$C_{33}H_{49}N_{3}O_{7}S_{2}$	663.9
N-*tert*-butyl-2-[2-hydroxy-3-[(3-hydroxy-2-methylbenzoyl)-amino]-4-phenylsulfanylbutyl]-3,4,4*a*,5,6,7,8,8*a*-octahydro-1*H*-isoquinoline-3-carboxamide; methanesulfonic acid	$C_{33}H_{49}N_{3}O_{7}S_{2}$	663.9
tert-butyl *N*-[6-[[1-[(1-amino-1-oxo-3-phenylpropan-2-yl)-amino]-4-methyl-1-oxopentan-2-yl]-amino]-5-benzyl-3-hydroxy-6-oxo-1-phenylhexan-2-yl]-carbamate	$C_{39}H_{52}N_{4}O_{6}$	672.9
5-hydroxy-2-(4-methoxyphenyl)-8-(3-methylbut-2-enyl)-7-[3,4,5-trihydroxy-6-(hydroxymethyl)-oxan-2-yl]-oxy-3-(3,4,5-trihydroxy-6-methyloxan-2-yl)-oxychromen-4-one	$C_{33}H_{40}O_{15}$	676.7
(8-methyl-8-azabicyclo[3.2.1]-octan-3-yl) 3-hydroxy-2-phenylpropanoate; sulfuric acid	$C_{34}H_{48}N_{2}O_{10}S$	676.8
sodium; 4-[2-[5-ethyl-5-[5-[6-hydroxy-6-(hydroxymethyl)-3,5-dimethyloxan-2-yl]-3-methyloxolan-2-yl]-oxolan-2-yl]-7-hydroxy-2,8-	$C_{36}H_{61}NaO_{11}$	692.9

dimethyl-1,10-dioxaspiro[4.5]-decan-9-yl]-3-methoxy-2-methylpentanoate		
(8-methyl-8-azabicyclo[3.2.1]-octan-3-yl) 3-hydroxy-2-phenylpropanoate; sulfuric acid; hydrate	$C_{34}H_{50}N_2O_{11}S$	694.8
7-chloro-4-(dimethylamino)-1,5,10,11,12*a*-pentahydroxy-6-methylidene-3,12-dioxo-4,4*a*,5,5*a*-tetrahydrotetracene-2-carboxamide; 2-hydroxy-5-sulfobenzoic acid	$C_{29}H_{27}ClN_2O_{14}S$	695.0
5-amino-2-(aminomethyl)-6-[4,6-diamino-2-[4-[3-amino-6-(aminomethyl)-4,5-dihydroxyoxan-2-yl]-oxy-3-hydroxy-5-(hydroxymethyl)-oxolan-2-yl]-oxy-3-hydroxycyclohexyl]-oxyoxane-3,4-diol; sulfuric acid	$C_{23}H_{48}N_6O_{17}S$	712.7
5-amino-2-(aminomethyl)-6-[5-[3,5-diamino-2-[3-amino-4,5-dihydroxy-6-(hydroxymethyl)-oxan-2-yl]-oxy-6-hydroxycyclohexyl]-oxy-4-hydroxy-2-(hydroxymethyl)-oxolan-3-yl]-oxyoxane-3,4-diol; sulfuric acid	$C_{23}H_{47}N_5O_{18}S$	713.7
1,3-thiazol-5-ylmethyl *N*-[(2*S*,3*S*,5*S*)-3-hydroxy-5-[[(2*S*)-3-methyl-2-[[methyl-[(2-propan-2-yl-1,3-thiazol-4-yl)-methyl]-carbamoyl]-amino]-butanoyl]-amino]-1,6-diphenylhexan-2-yl]-carbamate	$C_{37}H_{48}N_6O_5S_2$	720.9
7-[(4*a*,9-dihydroxy-3-prop-2-enyl-2,4,5,6,7*a*,13-hexahydro-1*H*-4,12-methanobenzofuro[3,2-e]-isoquinolin-7-ylidene)-hydrazinylidene]-3-prop-2-enyl-2,4,5,6,7*a*,13-hexahydro-1*H*-4,12-methanobenzofuro[3,2-e]-isoquinoline-4a,9-diol; dihydrochloride	$C_{38}H_{44}Cl_2N_4O_6$	723.7
2-[6-[[2-[5-[5-[6-hydroxy-6-(hydroxymethyl)-3,5-dimethyloxan-2-yl]-3-methyloxolan-2-yl]-5-methyloxolan-2-yl]-7-methoxy-2,4,6-trimethyl-1,10-dioxaspiro[4.5]-decan-9-yl]-methyl]-3-methyloxan-2-yl]-propanoic acid	$C_{40}H_{68}O_{11}$	725.0
3-[(11R)-1,5,11,14-tetrahydroxy-10-(hydroxymethyl)-13-methyl-3-(3,4,5-trihydroxy-6-methyloxan-2-yl)-oxy-2,3,4,6,7,8,9,11,12,15,16,17-dodecahydro-1*H*-cyclopenta[a]-phenanthren-17-yl]-2*H*-furan-5-one; octahydrate	$C_{29}H_{60}O_{20}$	728.8
11,33-bis(cyclopropylmethyl)-19,25-dioxa-11,22,33-triazaundecacyclo[24.9.1.1^{8,14}.0^{1,24}.0^{2,32}.0^{4,23}.0^{5,21}.0^{7,12}.0^{8,20}.0^{30,36}.0^{18,37}]-heptatriaconta-4(23),5(21),14(37),15,17,26,28,30(36)-octaene-2,7,17,27-tetrol; dihydrochloride	$C_{40}H_{45}Cl_2N_3O_6$	734.7
(2*E*)-2-[(9*Z*,25*Z*,38*E*)-38-(2-hydroxyethylidene)-14,30-bis(prop-2-enyl)-8,24-diaza-14,30-diazoniaundecacyclo[25.5.2.2^{11,14}.1^{1,26}.1^{10,17}.0^{2,7}.0^{13,17}.0^{18,23}.0^{30,33}.0^{8,35}.0^{24,36}]-octatriaconta-2,4,6,9,18,20,22,25-octaen-28-ylidene]-ethanol; dichloride	$C_{44}H_{50}Cl_2N_4O_2$	737.8
4-amino-*N*-[5-amino-4-[3-amino-6-(aminomethyl)-4,5-dihydroxyoxan-2-yl]-oxy-3-[3,4-dihydroxy-5-(hydroxymethyl)-oxolan-2-yl]-oxy-2-hydroxycyclohexyl]-2-hydroxybutanamide; sulfuric acid	$C_{21}H_{45}N_5O_{20}S2$	751.7
1-*N*,3-*N*-bis(1,3-dihydroxypropan-2-yl)-5-(2-hydroxypropanoylamino)-2,4,6-triiodobenzene-1,3-dicarboxamide	$C_{17}H_{22}I_3N_3O_8$	777.1
3,6-diamino-*N*-[(6*Z*)-3-(2-amino-4-hydroxy-1,4,5,6-tetrahydropyrimidin-6-yl)-6-[(carbamoylamino)-methylidene]-9,12-bis(hydroxymethyl)-2,5,8,11,14-pentaoxo-1,4,7,10,13-pentazacyclohexadec-15-yl]-hexanamide; sulfuric acid	$C_{25}H_{45}N_{13}O_{14}S$	783.8
3-acetamido-5-[acetyl(methyl)-amino]-2,4,6-triiodo-*N*-[2,4,5-trihydroxy-6-(hydroxymethyl)-oxan-3-yl]-benzamide	$C_{18}H_{22}I_3N_3O_8$	789.1

1-*N*,3-*N*-bis(2,3-dihydroxypropyl)-2,4,6-triiodo-5-[(2-methoxyacetyl)-amino]-3-*N*-methylbenzene-1,3-dicarboxamide	$C_{18}H_{24}I_3N_3O_8$	790.9
but-2-enedioic acid; *N*-[2-[[2-hydroxy-3-(4-hydroxyphenoxy)-propyl]-amino]-ethyl]-morpholine-4-carboxamide	$C_{36}H_{54}N_6O_{14}$	794.8
4-[2-(4-benzylpiperidin-1-yl)-1-hydroxypropyl]-phenol; 2,3-dihydroxybutanedioic acid	$C_{46}H_{60}N_2O_{10}$	801.0
dipotassium; [2-[(5-carboxy-15-hydroxy-9-methyl-14-methylidene-7-tetracyclo[11.2.1.0^{1,10}.0^{4,9}]-hexadecanyl)-oxy]-6-(hydroxymethyl)-3-(3-methylbutanoyloxy)-5-sulfonatooxyoxan-4-yl] sulfate	$C_{30}H_{44}K_2O_{16}S2$	803.0
(*E*)-but-2-enedioic acid; 4-[(2*S*)-2-[[(2*S*)-2-hydroxy-2-[4-hydroxy-3-(hydroxymethylamino)-phenyl]-ethyl]-amino]-propyl]-benzaldehyde	$C_{42}H_{52}N_4O_{12}$	804.9
[3,4,5-trihydroxy-6-(hydroxymethyl)-oxan-2-yl] 13-[4,5-dihydroxy-6-(hydroxymethyl)-3-[3,4,5-trihydroxy-6-(hydroxymethyl)-oxan-2-yl]-oxyoxan-2-yl]-oxy-5,9-dimethyl-14-methylidenetetracyclo[11.2.1.0^{1,10}.0^{4,9}]-hexadecane-5-carboxylate	$C_{38}H_{60}O_{18}$	804.9
1-*N*,3-*N*-bis(2,3-dihydroxypropyl)-5-[(2-hydroxyacetyl)-(2-hydroxyethyl)-amino]-2,4,6-triiodobenzene-1,3-dicarboxamide	$C_{18}H_{24}I_3N_3O_9$	807.1
5-[acetyl(2,3-dihydroxypropyl)-amino]-1-*N*,3-*N*-bis(2,3-dihydroxypropyl)-2,4,6-triiodobenzene-1,3-dicarboxamide	$C_{19}H_{26}I_3N_3O_9$	821.1
(*E*)-but-2-enedioic acid; *N*-[2-hydroxy-5-[1-hydroxy-2-[1-(4-methoxyphenyl)-propan-2-ylamino]-ethyl]-phenyl]-formamide; dihydrate	$C_{42}H_{56}N_4O_{14}$	840.9
[4,12-diacetyloxy-15-(3-benzamido-2-hydroxy-3-phenylpropanoyl)oxy-1,9-dihydroxy-10,14,17,17-tetramethyl-11-oxo-6-oxatetracyclo[11.3.1.0^{3,10}.0^{4,7}]-heptadec-13-en-2-yl] benzoate	$C_{47}H_{51}NO_{14}$	853.9
2-[5-hydroxy-6-(hydroxymethyl)-2-[(10,14,16,20-tetramethyl-22-azahexacyclo[12.10.0.0^{2,11}.0^{5,10}.0^{15,23}.0^{17,22}]-tetracos-4-en-7-yl)-oxy]-4-[3,4,5-trihydroxy-6-(hydroxymethyl)-oxan-2-yl]-oxyoxan-3-yl]-oxy-6-methyloxane-3,4,5-triol	$C_{45}H_{73}NO_{15}$	868.1
[6-[[3,4-dihydroxy-6-(hydroxymethyl)-5-(3,4,5-trihydroxy-6-methyloxan-2-yl)-oxyoxan-2-yl]-oxymethyl]-3,4,5-trihydroxyoxan-2-yl] 10,11-dihydroxy-9-(hydroxymethyl)-1,2,6*a*,6*b*,9,12*a*-hexamethyl-2,3,4,5,6,6*a*,7,8,8*a*,10,11,12,13,14*b*-tetradecahydro-1*H*-picene-4*a*-carboxylate	$C_{48}H_{78}O_{19}$	959.1
2-[2-[[2-[[2-[2-(2-hydroxyethoxy)-ethoxymethoxy]-3-[[2-[2-(2-hydroxyethoxy)-ethoxymethoxy]-5-(2,4,4-trimethylpentan-2-yl)-phenyl]-methyl]-5-(2,4,4-trimethylpentan-2-yl)-phenyl]-methyl]-4-(2,4,4-trimethylpentan-2-yl)-phenoxy]-methoxy]-ethoxy]-ethanol	$C_{59}H_{96}O_{12}$	997.4
[(2*R*,3*R*,4*S*,6*S*)-6-[(2*R*,3*S*,4*S*,6*S*)-6-[(2*R*,3*S*,4*S*,6*R*)-6-[[(3*S*,5*R*,8*R*,9*R*,10*S*,12*R*,13*S*,14*S*,17*R*)-12,14-dihydroxy-8,9,10,13-tetramethyl-17-(5-oxo-2*H*-furan-3-yl)-1,2,3,4,5,6,7,11,12,15,16,17-dodecahydrocyclopenta[a]-phenanthren-3-yl]-oxy]-4-hydroxy-2-methyloxan-3-yl]-oxy-4-hydroxy-2-methyloxan-3-yl]-oxy-2-methyl-3-[(2*S*,3*R*,4*S*,5*S*,6*R*)-3,4,5-trihydroxy-6-(hydroxymethyl)oxan-2-yl]-oxyoxan-4-yl] acetate	$C_{51}H_{80}O_{20}$	1013.2
2-[2-[4,5-dihydroxy-2-(hydroxymethyl)-6-(5',7,9,13-tetramethylspiro[5-oxapentacyclo[10.8.0.0^{2,9}.0^{4,8}.0^{13,18}]-icosane-6,2'-piperidine]-16-yl)-oxyoxan-3-yl]-oxy-5-hydroxy-6-(hydroxymethyl)-4-(3,4,5-trihydroxyoxan-2-yl)-oxyoxan-3-yl]oxy-6-(hydroxymethyl)-oxane-3,4,5-triol	$C_{50}H_{83}NO_{21}$	1034.2
calcium; 7-[2-(4-fluorophenyl)-3-phenyl-4-(phenylcarbamoyl)-5-propan-2-ylpyrrol-1-yl]-3,5-dihydroxyheptanoate	$C_{66}H_{68}CaF_2N_4O_{10}$	1155.3

5-amino-*N*-(3-amino-2,2-dimethyl-3-oxopropyl)-4-hydroxy-7-[[4-methoxy-3-(3-methoxypropoxy)-phenyl]-methyl]-8-methyl-2-propan-2-ylnonanamide; but-2-enedioic acid	$C_{64}H_{110}N_6O_{16}$	1219.6
3-[[2-[[3-[acetyl(methyl)-amino]-2,4,6-triiodo-5-(methylcarbamoyl)-benzoyl]-amino]-acetyl]-amino]-5-(2-hydroxyethylcarbamoyl)-2,4,6-triiodobenzoic acid	$C_{24}H_{21}I_6N_5O_8$	1268.9
acetic acid; *N*-[1-[[1-[[1-[[1-[[1-[[1-[[1-[2-[(carbamoylamino)-carbamoyl]-pyrrolidin-1-yl]-5-(diaminomethylideneamino)-1-oxopentan-2-yl]-amino]-4-methyl-1-oxopentan-2-yl]amino]-3-[(2-methylpropan-2-yl)-oxy]-1-oxopropan-2-yl]-amino]-3-(4-hydroxyphenyl)-1-oxopropan-2-yl]-amino]-3-hydroxy-1-oxopropan-2-yl]-amino]-3-(1*H*-indol-3-yl)-1-oxopropan-2-yl]-amino]-3-(1*H*-imidazol-5-yl)-1-oxopropan-2-yl]-5-oxopyrrolidine-2-carboxamide	$C_{61}H_{88}N_{18}O_{16}$	1329.5
cobalt(3+); [5-(5,6-dimethylbenzimidazol-1-yl)-4-hydroxy-2-(hydroxymethyl)-oxolan-3-yl] 1-[3-[2,13,18-tris(2-amino-2-oxoethyl)-7,12,17-tris(3-amino-3-oxopropyl)-3,5,8,8,13,15,18,19-octamethyl-2,7,12,17-tetrahydro-1*H*-corrin-24-id-3-yl]-propanoylamino]-propan-2-yl phosphate; cyanide	$C_{63}H_{89}CoN_{14}O_{14}P$	1355.4
2-[3-(diaminomethylideneamino)-4-[3-[4,5-dihydroxy-6-(hydroxymethyl)-3-(methylamino)-oxan-2-yl]-oxy-4-formyl-4-hydroxy-5-methyloxolan-2-yl]-oxy-2,5,6-trihydroxycyclohexyl]-guanidine; sulfuric acid	$C_{42}H_{84}N_{14}O_{36}S_3$	1457.4
2-[3-(diaminomethylideneamino)-4-[3-[4,5-dihydroxy-6-(hydroxymethyl)-3-(methylamino)-oxan-2-yl]-oxy-4-hydroxy-4-(hydroxymethyl)-5-methyloxolan-2-yl]-oxy-2,5,6-trihydroxycyclohexyl]-guanidine; sulfuric acid	$C_{42}H_{88}N_{14}O_{36}S_3$	1461.4
48-[3-(4-amino-5-hydroxy-4,6-dimethyloxan-2-yl)oxy-4,5-dihydroxy-6-(hydroxymethyl)-oxan-2-yl]-oxy-22-(2-amino-2-oxoethyl)-5,15-dichloro-2,18,32,35,37-pentahydroxy-19-[[4-methyl-2-(methylamino)-pentanoyl]-amino]-20,23,26,42,44-pentaoxo-7,13-dioxa-21,24,27,41,43-pentazaoctacyclo[26.14.2.2^{3,6}.2^{14,17}.1^{8,12}.1^{29,33}.0^{10,25}.0^{34,39}]-pentaconta-3,5,8(48),9,11,14,16,29(45),30,32,34(39),35,37,46,49-pentadecaene-40-carboxylic acid; hydrochloride	$C_{66}H_{76}Cl_3N_9O_{24}$	1485.7
5-[acetyl-[3-[*N*-acetyl-3,5-bis(2,3-dihydroxypropylcarbamoyl)-2,4,6-triiodoanilino]-2-hydroxypropyl]-amino]-1-*N*,3-*N*-bis(2,3-dihydroxypropyl)-2,4,6-triiodobenzene-1,3-dicarboxamide	$C_{35}H_{44}I_6N_6O_{15}$	1550.2
